# Precise in vivo RNA base editing with a wobble-enhanced circular CLUSTER guide RNA

**DOI:** 10.1038/s41587-024-02313-0

**Published:** 2024-07-12

**Authors:** Philipp Reautschnig, Carolin Fruhner, Nicolai Wahn, Charlotte P. Wiegand, Sabrina Kragness, John F. Yung, Daniel T. Hofacker, Jenna Fisk, Michelle Eidelman, Nils Waffenschmidt, Maximilian Feige, Laura S. Pfeiffer, Annika E. Schulz, Yvonne Füll, Erez Y. Levanon, Gail Mandel, Thorsten Stafforst

**Affiliations:** 1 Interfaculty Institute of Biochemistry, Faculty of Science, University of Tübingen, Tübingen, Germany; 2 Vollum Institute, Oregon Health and Science University, Portland, OR USA; 3 Mina and Everard Goodman Faculty of Life Sciences, Bar-Ilan University, Ramat Gan, Israel; 4 The Institute of Nanotechnology and Advanced Materials, Bar-Ilan University, Ramat Gan, Israel; 5 Gene and RNA Therapy Center (GRTC), Faculty of Medicine, University of Tübingen, Tübingen, Germany; 6 iFIT Cluster of Excellence (EXC2180) Image-Guided and Functionally Instructed Tumor Therapies, University of Tübingen, Tübingen, Germany

**Keywords:** RNA, Preclinical research, Neurodevelopmental disorders, Biomedical engineering

## Abstract

Recruiting the endogenous editing enzyme adenosine deaminase acting on RNA (ADAR) with tailored guide RNAs for adenosine-to-inosine (A-to-I) RNA base editing is promising for safely manipulating genetic information at the RNA level. However, the precision and efficiency of editing are often compromised by bystander off-target editing. Here, we find that in 5′-UAN triplets, which dominate bystander editing, G•U wobble base pairs effectively mitigate off-target events while maintaining high on-target efficiency. This strategy is universally applicable to existing A-to-I RNA base-editing systems and complements other suppression methods such as G•A mismatches and uridine (U) depletion. Combining wobble base pairing with a circularized format of the CLUSTER approach achieves highly precise and efficient editing (up to 87%) of a disease-relevant mutation in the Mecp2 transcript in cell culture. Virus-mediated delivery of the guide RNA alone realizes functional MeCP2 protein restoration in the central nervous system of a murine Rett syndrome model with editing yields of up to 19% and excellent bystander control in vivo.

## Abstract (web summary)

Using G•U wobble base pairs at specific loci increases RNA base-editing precision and efficiency.

## Main

Site-directed adenosine-to-inosine (A-to-I) RNA base editing is a very promising technology with a clear path for clinical application^1,2^. Hydrolytic deamination of A by enzymes of the ADAR family (adenosine deaminases acting on RNA) produces an inosine, which is biochemically interpreted as G in many cellular processes such as splicing or translation and, consequently, functionally substitutes A with G on the RNA level^3,4^. There are three catalytically active human ADAR proteins: constitutively and ubiquitously expressed ADAR1 p110, interferon-inducible ADAR1 p150 and ADAR2. In the past, ADAR deaminase domains have been engineered into various artificial editing approaches that enable the efficient and highly programmable editing of any given target A in the transcriptome by applying customized guide RNAs and simple Watson–Crick base-pairing rules. Typical examples are the SNAP-ADAR^5,6^, the λN-ADAR^7–9^ and the Cas13-ADAR approaches^10–12^. However, even after several rounds of optimization, major limitations of such systems remain: (1) a guide RNA plus a protein component needs to be delivered; (2) nonhuman protein domains are included; and (3) global off-target editing hampers the clinical development. An elegant solution to all three limitations could be to harness ubiquitously expressed endogenous ADAR enzymes for RNA base editing. It was shown recently that endogenous ADAR1 can be recruited by either chemically modified antisense oligonucleotides (ASOs)^13,14^ or genetically encoded guide RNAs^15–18^. Genetically encoded guide RNAs are particularly desired for the long-lasting reversal of disease-causing G>A point mutations in vivo by viral delivery of the guide RNA component.

Genetically encoded guide RNAs currently suffer from massive bystander editing in the guide RNA–target RNA duplex. This is, on one hand, caused by the large size of the duplex (70–200 bp) and, on the other hand, by the ubiquitous presence of highly editable A bases all over the duplex. Both ADAR1 and ADAR2 prefer similar nearest neighboring bases (for example, U > A > C > G at the 5′ position relative to the target A and G > C ≈ A > U or G > C > U ≈ A at the 3′ position, respectively)^19^. Consequently, bystander editing is dominated by a handful of preferred triplets, particularly all four 5′-UAN triplets (N = A, U, G or C), 5′-AAG and 5′-CAG. Bystander editing can lead to unwanted recoding events in the target and might even cause ribosome stalling^20^. Thus, the avoidance of bystander editing represents a major engineering problem for encodable RNA base-editing systems. Today, several strategies have been suggested. In the LEAPER approach^16,17^, bystander editing is usually suppressed by mismatching some or even all bystander-prone A bases with G. The rationale behind this is the preference of the ADAR deaminase for a specific counter base (C > U > A or G) opposite the targeted A^21^. A more recent approach is U depletion of the guide RNA by keeping off-target prone A bases unpaired, thus producing single-nucleotide bulges within the guide RNA–mRNA duplex^20^. However, both strategies do not always work optimally as they sometimes fail to suppress bystander editing and often reduce editing efficiency, sometimes even dramatically (for example, in A-rich sequence contexts). Consequently, using these strategies to improve editing precision can cost notable editing efficiency. An alternative solution is presented in the CLUSTER approach^15^. Here, the presence of editable triplets in the guide RNA–mRNA duplex is minimized by subdividing the guide RNA into several functional segments. Each segment is designed to bind the target transcript in areas selected for the absence of editable A bases. Because the individual segments of the CLUSTER guide RNAs, which we refer to as recruitment sequences (RSs), can be arranged closely within a window of a few hundred nucleotides around the target site, the approach leads to a very good control of bystander editing and high editing yields. However, in target transcripts that are very rich in highly editable A bases, the RSs currently have to be placed at large distances, which sometimes compromises editing efficiency. In a somewhat related solution, the guide RNA–mRNA duplex is regularly interrupted by bulged-out nucleotides, which leads to a reduction in bystander editing^18^. While the latter guide RNAs are very simple to design, the CLUSTER approach allows the assembly of guide RNAs from a much larger sequence space and was shown to enable a boost of editing efficiency by computationally selecting CLUSTER guide RNAs that avoid inhibitory self-folding.

In the human transcriptome, several highly efficient and precise editing events are known, which are guided by *cis*-acting intronic editing complementary sequences (ECS) that fold back to the target site inside an exon^22–28^. Notably, such natural editing sites are typically not found in perfect RNA helices but rather contain bulges, mismatches and wobble base pairs, which may serve to suppress bystander editing while preserving high on-target editing yields. The exact structural layer that makes an RNA a good or poor substrate for ADAR is still underexplored, although several recent studies have started to address this issue^29–31^.

Here, we systematically explore the rational use of G•U wobble base pairs to affect editing precision and efficiency. The G•U wobble base pair is the most abundant type of non-Watson–Crick base pair in the transcriptome^32,33^. Surrounding the G•U wobble base, the RNA helix structure is perturbed, affecting the groove width, base stacking and electrostatic profile^33^. Specific structural effects induced by G•U wobble bases have been shown to be important for the interaction of double-stranded RNA (dsRNA)-binding proteins with dsRNA substrates, including ADAR^34^. In this study, we systematically deduce rules on how to place G•U wobble base pairs to improve site-directed RNA base editing in terms of editing precision and efficiency. We apply G•U wobble base pairs in various RNA base-editing approaches including the CLUSTER approach, where it greatly improves the guide RNA design process, giving raise to highly precise and efficient circular CLUSTER guide RNAs that recode a Rett syndrome causing mutation in vivo. The disease in the model animals is caused by a loss-of-function mutation (W104>amber) in the transcription factor methyl CpG-binding protein 2 (MeCP2) that impairs its expression and binding to methylated DNA, thus deregulating >2,500 downstream genes, including many that are relevant for neuronal function^35^. This model system allowed us to assess key factors of successful in vivo transcript repair with endogenous Adars in the central nervous system (CNS).

## Results and discussion

### Wobble bases modulate editing depending on their orientation

**Fig. 1:**
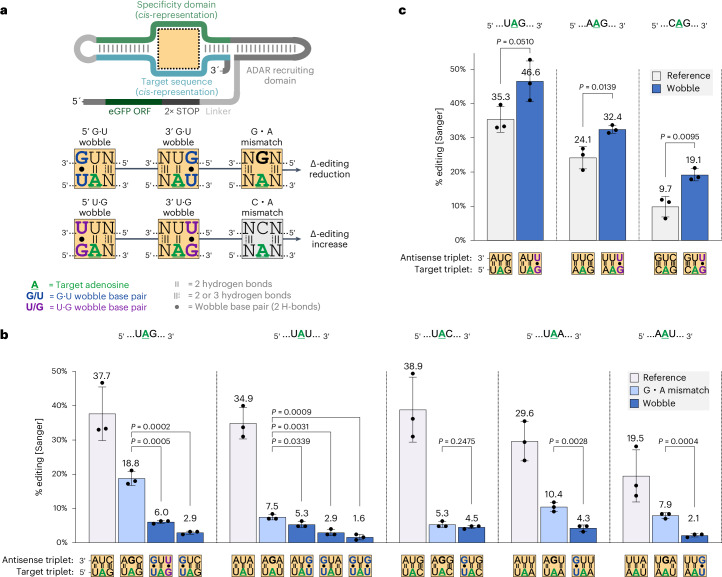
Wobble base pairs modulate RNA editing in an orientation-dependent manner. **a**, Design of the *cis*-acting editing reporter and illustration of the applied triplet base-pairing motifs. The orientation of wobble base pairs at nearest neighbor positions or the presence of G or C counter bases modulate Δ-editing at the central A of a triplet in comparison to its fully Watson–Crick base-paired counterpart. All triplet base-pairing motifs with ocher background color were installed at the empty dotted outline within the *cis*-acting editing construct to generate the results in **b**,**c**. **b**, Suppression of RNA editing in different target triplets using G•U wobble base pairs or G•A mismatches. **c**, Enhancing RNA editing in different target triplets using U•G wobble base pairs. The Sanger sequence analysis in **b** and **c** was performed after transfection of editing reporter plasmids into ADAR1 p110 Flp-In T-REx cells. Data in **b** and **c** are shown as the mean ± s.d. of *n* = 3 biological replicates. For statistical analysis, a Student’s *t*-test (two-tailed, parametric) was applied.

To characterize the effect of wobble base pairs in the nearest neighbor context, we first studied all 12 possible triplets that contained either a U or a G either 5′ and/or 3′ next to the target A. While the target A was always placed opposite of a U base, similar to a regular bystander site, the nearest neighboring nucleotides were either conventionally Watson–Crick base-paired or wobble base-paired (Fig. 1a). Under these circumstances, four types of wobble base pairs can occur. When the U base in a 5′-UAN or 5′-NAU triplet (N = any base) is base-paired with a G, we refer to this as a 5′-G•U or 3′-G•U wobble, respectively; when the G base in a 5′-GAN or 5′-NAG triplet is base-paired with a U, we refer to this as a 5′-U•G or 3′-U•G wobble, respectively. As a benchmark to previous studies^21^, we also included experiments where the target A was mismatched with a G base (G•A mismatch), to suppress editing.

The experiment used an editing reporter construct, based on the earlier R/G-guide RNA approach^36,37^. While the *trans*-acting guide RNA comprised only a double-stranded ADAR-recruiting domain and a single-stranded 20-nt specificity domain (SD), the *cis*-acting reporter additionally contained its own 20-nt target sequence (TS) and was located in the 3′ untranslated region (UTR) of an *eGFP* transcript to enable convenient Sanger sequencing readout (Fig. 1a). Editing was performed by transfecting the plasmid-borne editing reporter into Flp-In T-REx cells, overexpressing ADAR1 p110.

Importantly, we found that both, 5′-G•U and 3′-G•U wobble base pairs strongly suppress editing in the five triplets, 5′-UAN and 5′-AAU (Fig. 1b and Supplementary Fig. 1), which are highly editable under normal Watson–Crick base-pairing conditions and are, thus, a major source of bystander off-target events for *trans*-acting guide RNAs. In the 5′-UAU triplet, both G•U wobbles seemed to cooperate. For four of the five triplet contexts, the suppressive effect of the G•U wobble on editing significantly outcompeted the suppressive effect of the G•A mismatch (Fig. 1b). Interestingly, we found the opposite effect for the U•G wobble base pair. In particular, for the three triplets 5′-UAG, 5′-AAG and 5′-CAG, a clear editing-enhancing effect on the A directly adjacent to the 3′-U•G wobble was apparent (Fig. 1c). Because of the inability of ADARs to achieve sufficient editing at 5′-GAN triplets, the effect of 5′-U•G wobbles could not be verified (Supplementary Fig. 1).

The 5′-UAG triplet can simultaneously accommodate both a suppressive 5′-G•U and an enhancing 3′-U•G wobble base pair and, thus, allows studying their interplay. Our data suggest that the suppressive effect of the G•U wobble entirely dominates the activating effect of the U•G wobble (Fig. 1b).

It is intriguing to speculate that the enhancing effect of the 3′-U•G wobble could be combined with or replace the activating effect of the commonly used C•A mismatch at an on-target site such as 5′-UAG. We tested this idea using the CLUSTER guide RNA system on three endogenous targets (Supplementary Fig. 2a,b), with a mixed outcome. Only in one example (*ACTB*) was the U•G wobble a promising alternative to the C•A mismatch, although it could be useful in certain other sequence contexts for guide RNA structure optimization.

A guide RNA might find and bind to near-cognate sequences within the transcriptome to induce off-target editing. In this context, we aimed to understand whether a randomly occurring G•U wobble suppresses editing only in the context of a U-paired A or also in the rare context of a C-mismatched A, which is known to be more prone to editing. We evaluated the potential of 5′-G•U wobbles for suppressing editing at 5′-UAG sites where the A was mismatched with C. In two of three examples (*GUSB* and *NUP43*), the suppressive effect of the 5′-G•U wobble was strong enough to suppress editing even at the mismatched A (Supplementary Fig. 2c).

**Extended Data Fig. 1:**
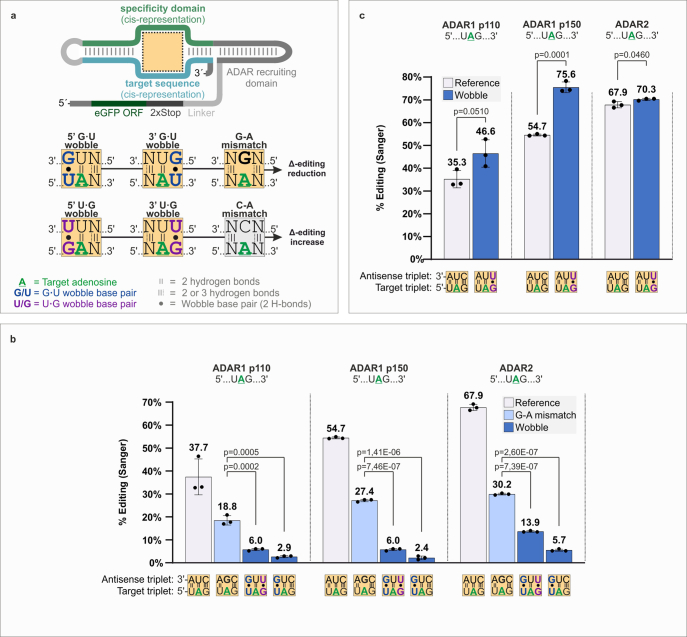
The enhancing and suppressing effects of G·U and U·G wobble base pairs on RNA editing apply to all catalytically active ADAR isoforms. **(a)** Schematic of the utilized cis-acting editing reporter construct. **(b)** Editing yields when applying G·A mismatches or G·U wobbles for editing suppression at the 5’-UAG triplet in either ADAR1 p110-, ADAR p150- or ADAR2-Flp-In T-REx cells. The exact position of each mismatch or wobble is indicated below each bar. The target adenosine is highlighted in green, bold and underlined. Bases that induce wobbles or mismatches are highlighted in dark blue and bold. The 5’ and 3’ orientation of the triplet and the used ADAR isoform are shown above the grouped bars. **(c)** As b) but regarding editing yields when applying enhancing U·G wobbles. Data for b) and c) are shown as the mean ± s.d. of N = 3 biological replicates per bar. For statistical analysis, a student’s *t*-test (two-tailed, parametric) was applied.

To see whether the enhancing and suppressing effects of wobble base pairs also apply for ADAR1 p150 and ADAR2, we selected the 5′-UAG triplet, which is a particularly frequent site of bystander editing, and transfected the corresponding plasmid-borne editing reporters into Flp-In T-REx cells, overexpressing ADAR1 p150 or ADAR2, respectively. As expected, the underlying mechanisms were not ADAR isoform dependent and worked equally well (Extended Data Fig. 1).

### G•U wobbles improve precision and efficiency of LEAPER guides

**Fig. 2:**
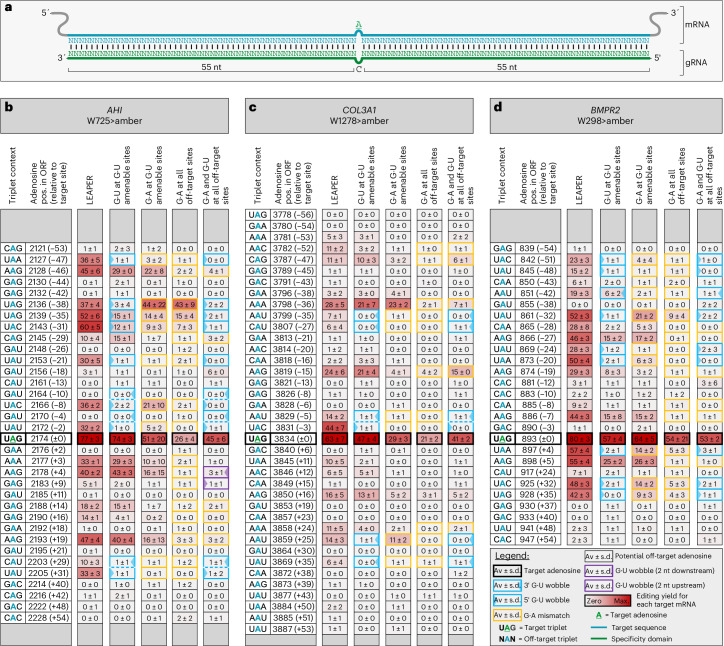
G•U wobbles improve efficiency and precision of *trans*-acting LEAPER guide RNAs for exogenous targets. **a**, Schematic of 111-nt-long unstructured linear LEAPER guide RNA. **b**–**d**, Editing heat maps of the LEAPER guide RNA-binding sites within the indicated transcripts: *AHI1* (**b**), *COL3A1* (**c**) and *BMPR2* (**d**). The basic design column, LEAPER, lacks G•A mismatches and wobble base pairs. The other guide RNAs contain either G•A mismatches or G•U wobbles at G•U-amenable sites or a combination of both solutions at all bystander sites. In the latter case, G•A mismatches are placed at sites not amenable to G•U wobbles. The triplet context for each listed editing event is given with the target A highlighted in green and all off-target A bases in blue. The position of each site is given relative to the transcript and the target A (±0 position). Editing was performed with plasmid-borne guide RNA and target in HeLa cells (endogenous ADAR). Data are shown as the mean editing percentage ± s.d. of *n* = 3 (*AHI1* and *COL3A1*) or *n* = 5 (*BMPR2*) biological replicates.

**Extended Data Fig. 2:**
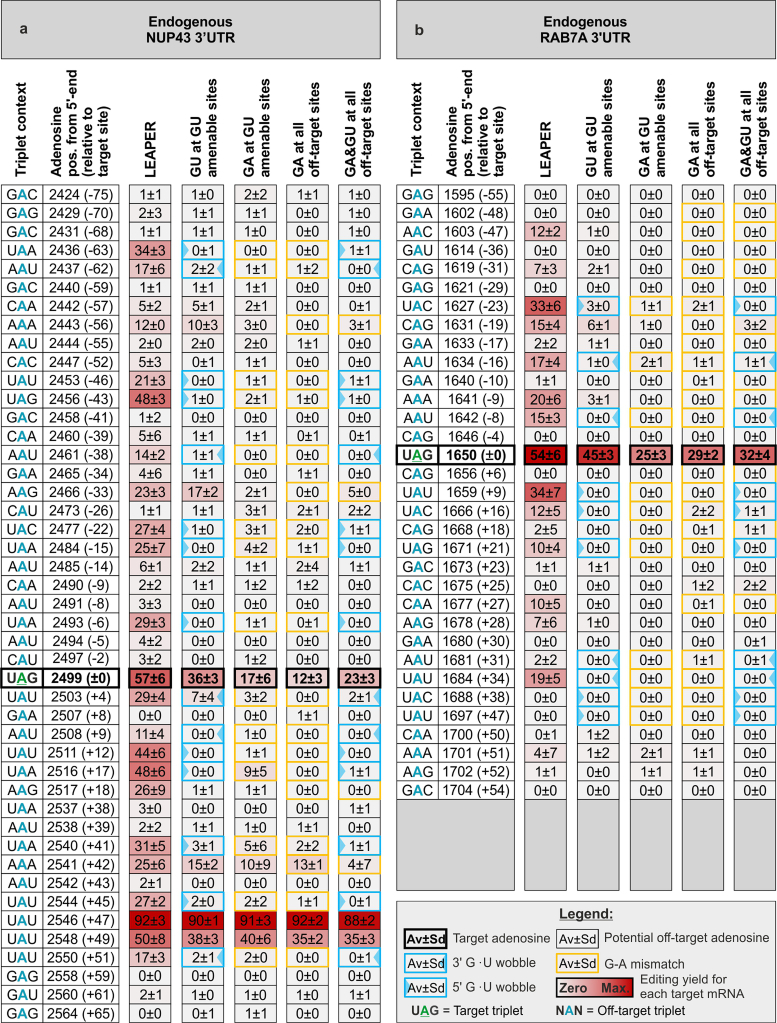
G·U wobbles improve efficiency and precision of trans-acting LEAPER guide RNAs for endogenous targets. **(a-b)** Editing heat-map of the LEAPER guide RNA binding site within the indicated transcripts. The basic design column, LEAPER, lacks G·A mismatches and wobble base pairs. The other guide RNAs contain either G·A mismatches or G·U wobbles at G·U amenable sites or a combination of both solutions at all bystander sites. In the latter case, G·A mismatches are placed at sites not amenable to G·U wobbles. The triplet context for each listed editing event is given with the target adenosine highlighted in green and all off-target adenosines in blue. The position of each site is given relative to the transcript and the target adenosine (±0 position). The editing events detected at position 2546 ( + 47) and 2548 ( + 49) of the NUP43 transcript were natural events and were thus skipped in regard to bystander suppression. Editing was performed with plasmid-borne guide RNA and endogenous target transcripts in HEK293FT cells (endogenous ADAR). Data are shown as the mean editing percentage ± s.d. of N = 3 biological replicates.

We next applied G•U wobble base pairs in the context of unstructured 111-nt-long LEAPER guide RNAs^16^ (Fig. 2a and Supplementary Fig. 3), which are highly prone to bystander editing. Our data (Fig. 1b) indicated that G•U wobble base pairs would be convenient to suppress bystander editing in the five highly editable triplets 5′-UAN (N = A, U, G or C) and 5′-AAU and could be combined with the G•A mismatch at all other editable triplets, such as 5′-AAG and 5′-CAG. To test this concept, *trans*-acting LEAPER guide RNAs (Fig. 2a) encoded on plasmids were cotransfected into HeLa cells with plasmids carrying the full-length complementary DNA (cDNA) of one of three different target genes (*AHI1* (ref. ^38^), *COL3A1* (ref. ^39^) and *BMPR2* (ref. ^40^)) each carrying a disease-relevant W>amber STOP mutation (5′-UAG) (Fig. 2b–d). To also evaluate endogenous targets, LEAPER guide RNAs targeting a 5′-UAG within the 3′ UTR of *NUP43* and *RAB7A* were transfected into HEK293FT cells expressing these genes (Extended Data Fig. 2a,b). As seen in both settings, LEAPER guide RNAs recruited endogenous ADAR to induce significant on-target editing in each of the five targets (54–80%); however, this was contaminated with massive bystander editing (>10 sites per target), as previously reported^15^.

First, we tested for all five targets whether G•U wobble base pairs outcompete G•A mismatches to suppress bystander editing at such sites, where G•U wobbles are amenable (G•U at G•U-amenable sites) and compared such guide RNAs with guide RNAs that apply G•A mismatches at the same sites (G•A at G•U-amenable sites) (Fig. 2b–d and Extended Data Fig. 2a,b). Notably, we found that the G•U wobble strategy was very potent to suppress bystander editing, often but not always outcompeting the G•A mismatch approach. Moreover, the on-target editing yield was higher for four of the five targets (*AHI1*, 74% versus 51%, and *COL3A1*, 47% versus 29%, Fig. 2; endogenous *NUP43*, 36% versus 17%, and endogenous *RAB7A*, 45% versus 25%, Extended Data Fig. 2).

Second, we aimed to suppress bystander editing entirely by either fully relying on the prior-art G•A mismatch approach (G•A all off-target sites) or by combining G•A mismatches with G•U wobble base pairs (G•A and G•U at all off-target sites). For *AHI1* (Fig. 2b), only the combination of G•A mismatches with G•U wobble base pairs achieved the entire suppression of bystander editing and a good on-target editing yield (45%), while the guide RNA using only G•A mismatches suffered from residual bystander editing (position −38, 43%; position −35, 15%) and a reduced on-target efficiency (26%). These results highlight the power of G•U wobbles to improve the precision of LEAPER guide RNAs. For *COL3A1* (Fig. 2c) and *NUP43* (Extended Data Fig. 2a), the on-target yields were again clearly better when complementing G•A mismatches with wobble base pairs (41% versus 21% and 23% versus 12%, respectively). However, because of the lower yields at all edited sites, the G•A mismatch approach appeared to give slightly better precision. The *BMPR2* target transcript (Fig. 2d) gave equal on-target yields for both strategies but better precision when G•U wobble base pairs were included. In the case of *RAB7A* (Extended Data Fig. 2b), both designs performed similarly.

According to the literature, LEAPER guide RNAs that carry numerous G•A mismatches can suffer from a loss of editing efficiency^16^. The reason for this might be that G•A mismatches have the lowest duplex stability among all known nucleotide mismatches^41^. In contrast, the thermodynamic stability of the G•U wobble is comparable to the A-U base pair^32^. This might partly explain why on-target efficiency often benefitted when G•A mismatches were complemented with G•U wobble base pairs. Notably, the number of suppressive G•U wobble base pairs required to improve precision can be smaller than that of G•A mismatches because one G•U wobble acts simultaneously at its 5′ and 3′ nearest neighbor positions. Examples can be found in Fig. 2b,c (*AHI1*, positions +29 and +31; *COL3A1*, positions −5 and −3). Furthermore, we found several cases where G•A mismatching failed to fully suppress bystander editing, while the G•U wobble strategy succeeded (for example, *AHI1* at positions −38, −35 and −31 and *BMPR2* at positions −32, +32 and +35; Figure 2b,d).

After targeting both exogenous and endogenous transcripts ranging from high expression levels (*AHI1*, *BMPR2* and *COL3A1* all as cDNAs), over medium (*RAB7A*, normalized transcripts per million (nTPM) = 105) to low (*NUP43*, nTPM = 36) expression levels, our data suggest that the G•U wobble strategy is unaffected by target transcript abundance and, thus, widely applicable. Overall, the wobble strategy complements the prior-art G•A mismatch strategy very well and regularly improves both editing efficiency and precision.

### G•U wobbles are widely applicable to RNA base-editing tools

**Fig. 3:**
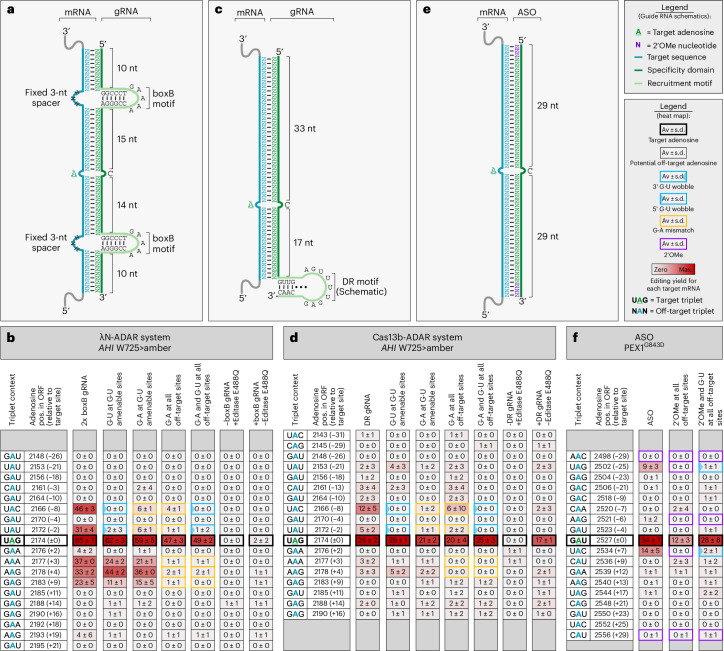
G•U wobble base pairs are broadly applicable to numerous side-directed RNA base-editing systems. **a**, Schematic of the λN-ADAR editing system. The 2× boxB motif-containing guide RNA (84 nt) binds its target mRNA (through 49 bp) to recruit the engineered hyperactive λN-ADAR2Q editase. **b**, Editing heat map of exogenous AHI1 W725>amber mRNA targeted by the λN-ADAR system. **c**, Schematic of the Cas13b-ADAR system. The DR motif-containing guide RNA (87 nt) binds its target mRNA (through 51 bp) to recruit the engineered PspCas13b-ADAR editase carrying a hyperactive ADAR2 E488Q deaminase domain. **d**, Editing heat map of exogenous AHI1 W725>amber mRNA targeted by the Cas13b-ADAR system. **e**, Schematic of a symmetric ASO bound to its target mRNA (through 58 nt). The ASO is end-blocked by 2′-OMe, contains phosphorothioate linkages and recruits endogenous ADARs. **f**, Editing heat map of the ASO binding site within exogenous *PEX1*^G843D^ transcript. In **b**,**d**,**f**, the basic designs (columns 2× boxB, DR guide RNA and ASO) do not contain G•A mismatches, wobble base pairs or 2′-OMe modifications beyond the end-blocks. The other guide RNAs contain additional 2′-OMe modifications, G•A mismatches, G•U wobbles at G•U-amenable sites or a combination of these solutions at all bystander sites. In the case of a combined solution, G•A mismatches or 2′-OMe modifications are placed at sites not amenable to G•U wobbles. The triplet context for each listed editing event is given with the target A highlighted in green and all off-target A bases in blue. The position of each site is given relative to the transcript and the target A (±0 position). Editing was performed in HeLa cells using plasmid-borne guide RNAs (2× boxB and DR guide RNA) and editase (λN-ADAR2Q and Cas13b-ADAR2Q) or ASOs recruiting endogenous ADAR. Data are shown as the mean editing percentage ± s.d. of *n* = 3 biological replicates.

Bystander editing is a common problem of all RNA base-editing systems, particularly those such as the λN-ADAR^8^ and Cas13-ADAR^10^ approaches (Fig. 3a–d and Supplementary Fig. 3) that also use genetically encoded guide RNAs. Thus, we applied the G•U wobble strategy with boxB guide RNAs (Fig. 3a) and direct repeat (DR) guide RNAs (Fig. 3c) to see whether it improves the precision of the λN-ADAR and the Cas13-ADAR approaches, respectively. For this, triple-plasmid protocols were used where plasmids encoding the guide RNA, the editase (λN-ADAR2Q or Cas13-ADAR2Q) and the target (*AHI1* W725X) were cotransfected into HeLa cells. In contrast to the LEAPER approach, the λN-ADAR and Cas13-ADAR approaches apply a hyperactive ADAR mutant and use guide RNAs with comparably short antisense part (boxB, 49 nt; DR, 59 nt). Because of the shorter duplex, the number of bystander sites is overall smaller. Nevertheless, the λN-ADAR approach induced significant bystander editing (Fig. 3b). Notably, amenable sites were readily controlled by G•U wobbles but not by G•A mismatches alone. A combination of G•U wobble and G•A mismatches was able to fully suppress bystander editing at a minor cost of editing efficiency (65% ± 7% to 49% ± 2%; Fig. 3b). For Cas13-ADAR, the combination of G•U wobble and G•A mismatches gave the best results in terms of editing efficiency (25% ± 3% versus 20% ± 4%) and allowed for complete bystander suppression (Fig. 3d). However, the Cas13-ADAR system itself gave dramatically lower editing yields compared to the λN-ADAR system (26% ± 2% versus 65% ± 7%) and showed little specificity for the Cas13b protein, as the editing yield of the DR guide RNA with and without overexpression of the editase differed only by ~9% (Fig. 3d).

In ADAR-recruiting ASOs, the strategic placement of chemical modifications allows to control bystander events^13,14^. However, dense chemical modification, for example with 2′-*O*-methylated ribose (2′-OMe), can interfere strongly with editing. Thus, we evaluated G•U wobble base pairs in a case where additional chemical modifications diminished the on-target efficiency. Using a chemically modified (phosphorothioate linkage and 2′-OMe end-blocked) 59-nt-long symmetric ASO (Fig. 3e), we targeted the *PEX1* transcript, specifically the G843D substitution causative for the peroxisome biogenesis disorder Zellweger syndrome^42^. While placement of additional 2′-OMe modifications at the −25, −6 and +7 positions controlled bystander editing, they also reduced the on-target yield drastically (34% ± 7% to 12% ± 3%; Fig. 3f). By contrast, a combination of G•U wobble base pairs and 2′-OMe modifications enabled the control of bystander editing while preserving the on-target yield (28% ± 8%; Fig. 3f). The latter example shows that ASO-based approaches can also potentially benefit from wobble base pairs to maintain high on-target yields.

### Superior off-target control in A-rich target sites

**Extended Data Fig. 3:**
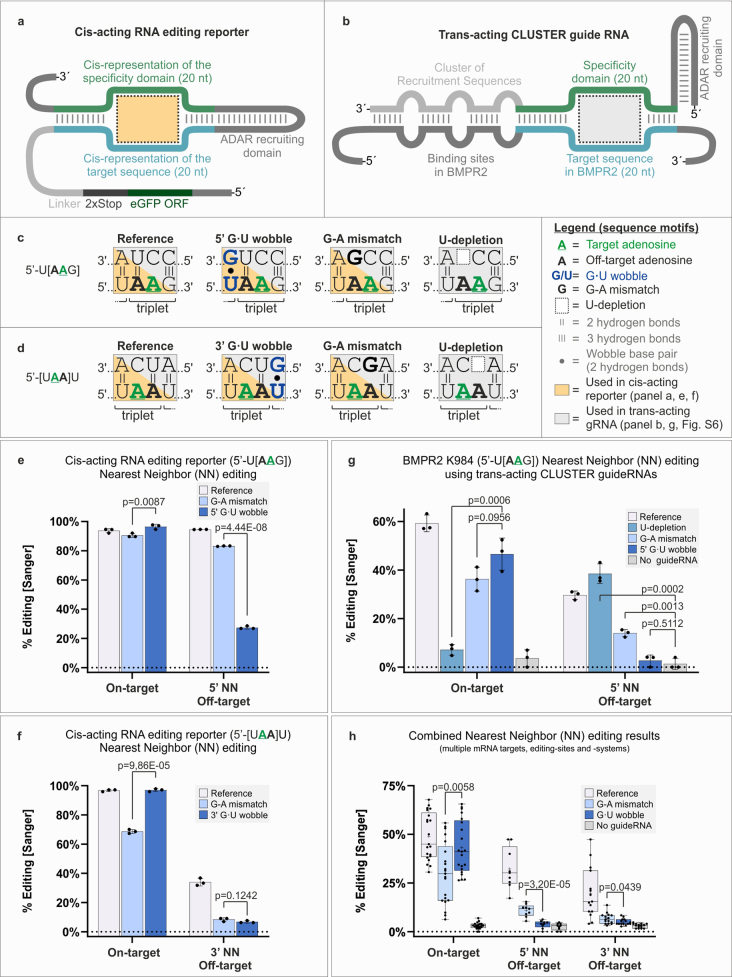
G·U wobbles significantly outperform previous solutions in bystander suppression at nearest neighbor (NN) sites. **(a)** Design of the cis-acting RNA editing reporter constructs used in e) and f). **(b)** Design of the linear trans-acting CLUSTER guide RNAs used in g). **(c)** Illustration of the base-pairing motifs applied to the constructs in a) (ocher) and b) (grey) that were used to suppress bystander editing at the 5´-U[**AA**G] sequence motif in e) and g). **(d)** As c) but for the 5´-[U**AA**]U sequence motif and the results in f). **(e)** Suppression of bystander editing at the 5´-NN **A** and concurrent effect on the target **A** editing yield in a 5’-U[**AA**G] sequence motif. **(f)** Similar to e) but targeting the **A** in a 5’-[U**AA**]U sequence motif and suppressing bystander editing at a 3´-NN **A**. Editing in e) and f) was performed in ADAR1 p110 Flp-In T-REx cells using plasmid-borne reporters as displayed in a). **(g)** Suppression of bystander editing at a 5´-NN **A** when editing a 5’-U[**AA**G] site (K984) of the human BMPR2 transcript using trans-acting CLUSTER guide RNAs. **(h)** Meta-analysis of bystander suppression at 5´- and 3´-NN sites over three different target transcripts (AHI1, BMPR2 and COL3A1), three different A-rich target triplets flanked either 5’ and/or 3’ by uridines (5’-[U**AA**]U, 5’-U[**AA**G], 5’-U[**AAA**]U), and applying two trans-acting site-directed RNA editing systems (CLUSTER/endogenous ADAR, boxB/λN-ADAR2Q). The full data set of the meta-analysis is shown in Supplementary Fig. S5. Editing in g) and h) was performed in HeLa cells using plasmid-borne editase (λN-ADAR2Q) and/or guide RNA (2x boxB, CLUSTER). Data in e) – g) are shown as the mean editing percentage ± s.d. of N = 3 biological replicates. Data in h) are shown as the mean editing percentage ± s.d. of three 5’-NN off-target sites, seven on-target sites, and five 3’-NN off-target sites containing N = 3 biological replicates per site. The center line represents the median, the plus-sign the mean. The whiskers extend to the minimum and maximum values. The box limits the 25^th^ and 75^th^ percentile. For statistical analysis, a student’s *t*-test (two-tailed, parametric) was applied.

The suppression of bystander editing in closest proximity to an on-target A is a common problem for all fully encoded RNA base-editing systems. Strategies such as G•A mismatching^16^ or U depletion^17^ often lead to a substantial loss of editing yield when they are applied too close to the on-target site. To assess the G•U wobble strategy for such a setting, we systematically tested how far the suppressive effect of a single G•U wobble base pair extends in the 5′ and 3′ directions. For this purpose, we again used *cis*-acting constructs that placed an editable duplex in direct extension of an ADAR-recruiting domain (R/G helix; Extended Data Fig. 3a and Supplementary Fig. 4) into the 3′ UTR of an *eGFP* reporter. We then evaluated the editing yields after transfection of these constructs into ADAR1 p110-expressing Flp-In T-Rex 293 cells (Supplementary Fig. 4). To study the suppressive effect of the 3′-G•U wobble in the 5′ direction, we studied a series of three 5′-UA(A)_i_U base-paring motifs (i = 1–3 A bases) with increasing distance between the 3′-G•U wobble and the on-target A (Supplementary Fig. 4a–c). Accordingly, we also designed a series of three 5′-U(A)_i_AG base-paring motifs (i = 1–3 A bases) to test the effect of a 5′-G•U wobble in the 3′ direction (Supplementary Fig. 4d–f). The target triplet was either 5′-UAA or 5′-AAG, hereinafter indicated by square brackets. In both series, we also benchmarked the effect of the G•A mismatch for the same bystander off-target A site. Neither the 5′-G•U nor the 3′-G•U wobble affected the on-target editing yield negatively at any distance tested. Instead, the suppressive effect was almost entirely focused on the direct 3′ and 5′ neighboring base. This was in clear contrast to the G•A mismatch where not only the mismatched base but also the first and sometimes even the second neighboring base in both directions were negatively affected (Supplementary Fig. 4). Thus, the G•U wobble strategy should be particularly strong to precisely suppress bystander editing close to an on-target A. We show exemplary data on how the 5′-G•U wobble controls editing precision in a 5′-U[AAG] base-pairing motif (Extended Data Fig. 3a,c,e) and how the 3′-G•U wobble acts in the 5′-[UAA]U base-pairing motif (Extended Data Fig. 3a,d,f), always in comparison to the G•A mismatch. In both cases, the G•U wobble clearly gave a better balance of editing efficiency over editing precision.

Next, we transferred the concept to *trans*-acting CLUSTER guide RNAs (linear design), which targeted a 5′-U[AAG] site in the *BMPR2* transcript (K984, on cDNA) by harnessing endogenous ADAR in HeLa cells (Extended Data Fig. 3b,c,g). As expected, the reference guide RNA showed good on-target yields (A, 59%; Extended Data Fig. 3g) but also a strong bystander editing at the 5′ neighboring A (30%). The strategically placed 5′-G•U wobble base pair was able to fully suppress this bystander editing. This was not the case with the G•A mismatch where only a partial suppression of bystander editing was achieved. Notably, the U depletion strategy^17^ even increased bystander editing to 38% (Extended Data Fig. 3g). The 5′-G•U wobble also gave the best on-target efficiency of the compared bystander solutions with 47% yield, whereas a G•A mismatch reduced the yield and U depletion almost fully blocked editing, highlighting the power of the G•U wobble strategy to achieve high efficiency and high precision in very A-rich triplet contexts where G•A mismatch and U depletion fail (Extended Data Fig. 3g and Supplementary Fig. 5a,b).

To show that this finding is highly generalizable, we performed a meta-analysis over three different target transcripts (*AHI1*, *BMPR2* and *COL3A1*) representing three different A-rich target triplets (5′-[UAA]U, 5′-U[AAG] and 5′-U[AAA]U) while using two different editing approaches, the CLUSTER guide RNA with endogenous ADAR and the boxB/λN-ADAR system with engineered ADAR. Notably, not only bystander control but also on-target efficiency was significantly better with G•U wobble base pairs, demonstrating the strength of the strategy to suppress bystander editing precisely within A-rich triplets (Extended Data Fig. 3h and Supplementary Fig. 5).

### Wobble bases improve the engineering of CLUSTER guide RNAs

**Extended Data Fig. 4:**
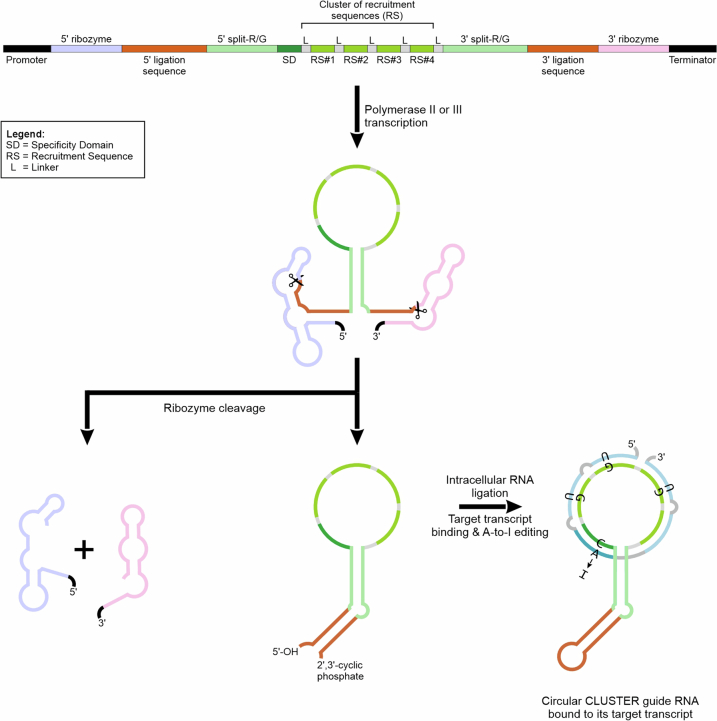
Circularization of a split-R/G CLUSTER guide RNA using the Tornado expression system. The guide RNA is expressed from either a polymerase III (for example U6) or a polymerase II (for example CAG) promoter with a corresponding terminator. 5’ and 3’ ribozymes auto-cleave the primary transcript resulting in a 5’-OH and a 2’,3’-cyclic phosphate overhang, respectively, which are subsequently ligated by the endogenous RTCB ligase. In the final guide RNA, the ligation stem (orange) forms a nearly continuous duplex with the split-R/G motif (light green), only interrupted by a five-nucleotide bulge. The circular guide RNA binds to its target transcript. Strategic placement of wobble base pairs within the recruitment sequences (RS) and/or the specificity domain (dark green) suppresses off-target editing, while a C-A mismatch within the specificity domain (SD) promotes on-target A-to-I RNA editing.

CLUSTER guide RNAs represent a recent strategy to harness endogenous ADAR for precise and efficient RNA base editing^15^. The basic concept combines an ADAR recruitment motif, a 20-nt SD that binds the target site and three or more additional RSs 15–20 nt in length that bind to the target mRNA over a larger stretch of sequence space in a multivalent fashion (Supplementary Fig. 3). In silico optimization of the guide RNA sequence is applied to choose RSs in such a way that highly editable A bases are avoided, enabling high control over bystander editing. Furthermore, guide RNAs with a high tendency to form inhibitory secondary structure are sorted out automatically, which helps to improve editing efficiency. Similar to the report for the LEAPER system^17^, we herein established the ribozyme-based Tornado expression system^43^ for circularization and, thus, stabilization of guide RNAs (Extended Data Fig. 4). Starting with a simple LEAPER design, we could verify the formation of cleanly circularized guide RNAs (Supplementary Fig. 6), which gave an improved editing efficiency on the endogenous *RAB7A* transcript (Supplementary Fig. 7). Particularly notable was the positive effect of circularization on the editing yield after stable integration of the LEAPER guide RNA cassette by the PiggyBac transposase into the genome of HeLa cells, which gave moderate and stable editing yields over several weeks even with the weaker Pol2 promoter, elongation factor 1α (EF1α) (Supplementary Fig. 8).

Compared to simple LEAPER guide RNAs, the flexible design of CLUSTER guide RNAs represented a considerable engineering challenge for circularization. The order of elements within the CLUSTER guide RNA, such as the SD, the RSs, the ADAR recruitment motif and the target mRNA exit points, can all be placed individually and relative to each other. On the guide RNA side, RSs can be placed 5′ and/or 3′ to the SD, while the ADAR recruitment motif can be flexibly placed anywhere in between. On the mRNA side, binding sites that correspond to the RS within the guide RNA can be located 5′ and/or 3′ of the TS, which corresponds to the SD within the guide RNA. Binding-site placement ultimately defines whether the exit points of the mRNA are close or distant relative to the ligation stem. Furthermore, the number and length of all antisense elements and linkers can be varied, which may individually affect the torsion within the guide RNA circle and, thus, its interaction with the target transcript.

To identify general design rules for circular CLUSTER guide RNAs, we tested various constructs on a luciferase reporter construct (Supplementary Figs. 9 and 10). We found that the mRNA exits should be placed approximately opposite of the SD and that the ADAR recruitment motif can be combined with the ligation stem into a larger RNA structure placed adjacent to the SD. Notably, a 5-nt bulge that separates ADAR recruitment motif and ligation stem achieved particularly high editing efficiency (Supplementary Fig. 10a–c, design L13). This ADAR recruitment motif was called the split-R/G motif and was used in subsequent designs.

After identifying general design principles for circular CLUSTER guide RNAs, we aimed to design an optimal guide RNA for a proof-of-concept in vivo study. Specifically, we aimed to target a premature STOP codon in the murine *Mecp2* transcript (W104>amber), which causes severe Rett syndrome-like symptoms in mice carrying this patient mutation^44^.

**Fig. 4:**
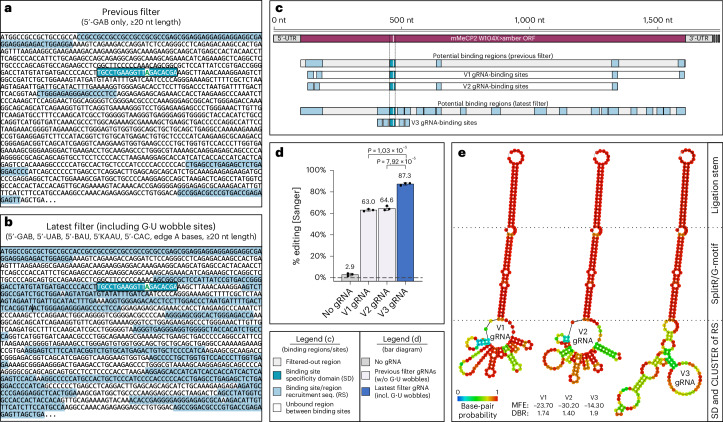
Wobble base pairing improves the design of CLUSTER guide RNAs. **a**, Results from an in silico search for RS-binding sites within the murine *Mecp2* W104>amber ORF when applying the previous filter settings to the GuideRNA-Forge tool. The binding site for the SD is displayed in turquoise and outlined in black. It contains the target A in green and underlined. All potential RS-binding regions ≥ 20 nt length, in which binding sites for RSs can be selected, are displayed in light blue. The previous filter excludes all A bases except for those within a 5′-GAB (B = U, C or G) triplet context. **b**, Results for the same in silico search with the latest filter that included A bases in a 5′-GAB, 5′-UAB, 5′-BAU, 5′-KAAU and 5′-CAC triplet context, in addition to allowing A bases independent of their sequence context at the edges of a detected binding region. **c**, Illustration of binding regions in which specific binding sites of RSs of three circular CLUSTER guide RNAs are located within the *Mecp2* ORF, generated using the previous filter (V1 and V2) or the latest filter (V3). **d**, Editing was performed with plasmid-borne guide RNA and murine *Mecp2* transcript in HeLa cells (endogenous ADAR). **e**, Secondary structure prediction of guide RNA V1–V3 generated using the ViennaRNA Package 2.0 (ref. ^54^). The mean free energy (MFE) of the antisense part (SD and CLUSTER of RS) is given in kcal per mol. The dot–bracket ratio (DBR) indicates the number of dots (unpaired bases) per bracket (paired bases) within the dot–bracket annotation of the antisense part. Data are shown as the mean editing percentage ± s.d. of *n* = 3 biological replicates. For statistical analysis, a Student’s *t*-test (two-tailed, parametric) was applied.

**Fig. 5:**
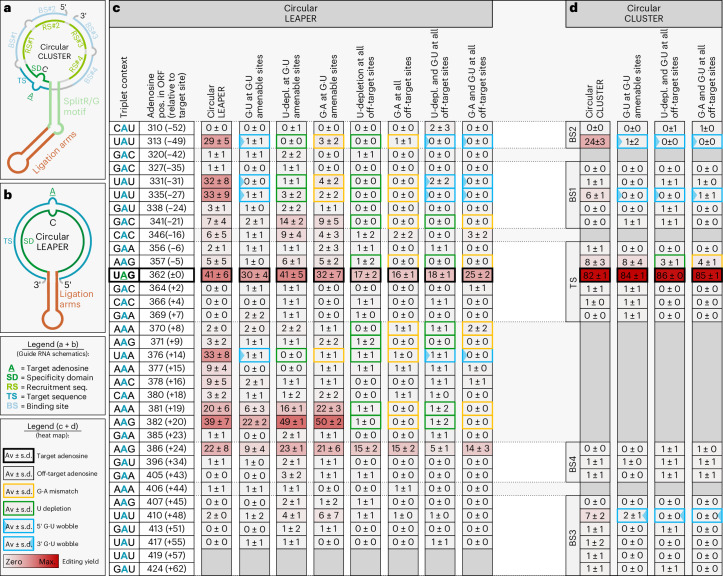
Circular CLUSTER versus circular LEAPER *Mecp2* guide RNAs. Editing heat map of guide RNA-binding sites within the murine *Mecp2* W104>amber transcript. The triplet context for each listed editing event is given with the target A highlighted in green and all off-target A bases in blue. The position of each site is given relative to the transcript and the target A (±0 position). Editing was performed with plasmid-borne guide RNA and murine *Mecp2* transcript in HeLa cells (endogenous ADAR). **a**, Circular CLUSTER guide RNA design. **b**, Circular LEAPER guide RNA design. **c**, Yields achieved using circular unstructured LEAPER guide RNAs containing a 111-nt-long antisense part. The basic design (column circular LEAPER) does not contain bystander solutions. The other guide RNAs either contain G•A mismatches or apply U depletion at all bystander sites, G•U wobbles at G•U-amenable sites or a combination of G•A mismatching or U depletion with G•U wobbles at amenable sites. **d**, Yields achieved using circular CLUSTER guide RNAs containing a 100-nt-long antisense part split into a targeting sequence (20 nt) and four RSs (each 20 nt). The basic design (column circular CLUSTER) does not contain bystander solutions. The other guide RNAs contain G•U wobbles at positions −49, −27 and +48, as well as no bystander solution, G•A mismatch or depleted U at position −5. Data in **a**,**b** are shown as the mean editing percentage ± s.d. of *n* = 3 biological replicates.

CLUSTER guide RNAs avoid bystander editing by choosing RSs that minimize the presence of editable A bases in the guide RNA–target RNA duplex. However, in highly A-rich target RNAs, such as *Mecp2*, individual binding regions for RSs can be separated by long distances within the target transcript (for example, spread over several exons) and the available sequence space for secondary structure optimization can be limited. Both effects can negatively impact the editing efficiency of CLUSTER guide RNAs^15^. However, by applying the wobble base-pairing strategy to suppress bystander editing, the highly limiting filter set that defined eligible A bases within the RS-binding regions could now be considerably expanded; 5′-UAB, 5′-BAU triplets (B = C, G or U) and 5′-KAAU (K = G or U) sequence motifs are now included, while only 5′-GAB triplets were previously allowed^15^. Furthermore, 5′-CAC triplets and all A bases located at either end of a binding region (edge A bases), which we identified as being resistant to off-target editing, could be used to expand the sequence space. With the old filter settings (Fig. 4a), a circular CLUSTER guide RNA with four RSs and a split-R/G ADAR recruitment motif needs substantial space on the *Mecp2* target RNA—specifically, 1,470 nt for guide RNA V1 and 1,208 nt for guide RNA V2 (Fig. 4c). In contrast, with the new filter settings (Fig. 4b), the entire guide RNA (V3) covers only 127 nt on the murine *Mecp2* transcript (Fig. 4c). This guide RNA V3 gave significantly better on-target editing than V1 and V2 (87% versus 63% and 65%), which used the old filter settings (Fig. 4d). Partially, this might also be attributed to the larger available sequence space, which allows selecting guide RNAs with a much lower level of inhibitory secondary structure (Fig. 4e). As expected, the expansion of eligible A bases led to the appearance of bystander editing at binding sites of the RSs, which were albeit well suppressed with the G•U wobble strategy (Fig. 5).

**Extended Data Fig. 5:**
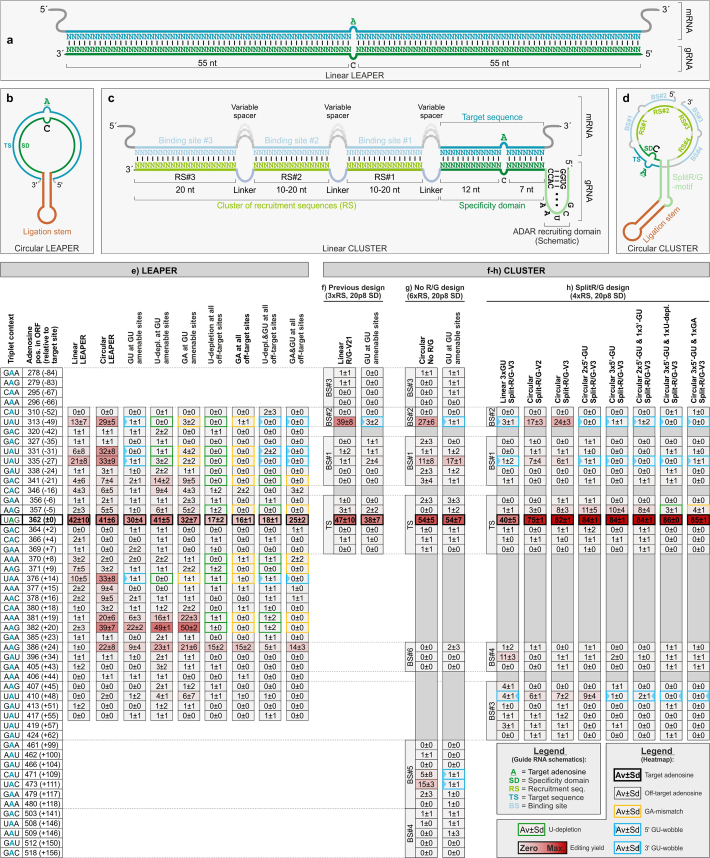
Extended data set for editing Mecp2 W104amber with LEAPER and CLUSTER guide RNAs. Panels **(a)** to **(d)** give schematic representations of the used linear and circular LEAPER and CLUSTER guide RNAs and their binding to the target mRNA. LEAPER guide RNAs were 111 nt long, centered with a C-A mismatch on the target adenosine. CLUSTER guide RNAs combined an ADAR-recruiting domain (R/G motif), a 20 nt long specificity domain containing the C-A mismatch with the target adenosine at position 8, and a cluster of three recruitment sequences of 20 nt length each. The circular CLUSTER guide RNA combines the ligation stem for circularization with the ADAR-recruiting motif into a split-R/G motif. (**e)** Editing heat-map using linear and circular LEAPER guide RNAs as seen in a) and b). (**f-h)** Editing heat map using linear and circular CLUSTER guide RNAs as seen in c) and d). Notably, the linear R/G version 21 guide RNA achieved already highly precise editing with an impressive 38 ± 7% on-target editing yield, clearly better than the best circular LEAPER guide RNA design (25 ± 2% on-target yield) with similar precision. A circular CLUSTER guide RNA with six recruitment sequences, but lacking the ADAR recruitment motif, achieved 54 ± 7% on-target editing with very high precision after applying the GU wobble strategy. However, adding a split-R/G recruiting motif achieved even better on-target editing yields of 75 ± 1% to 82 ± 1%, even though only 4 recruitment sequences were applied. GU wobbling was required to eliminate bystander editing at the binding sites of recruitment sequences. The triplet context for each listed editing event is given with the edited adenosine highlighted in bold green. The position of each bystander site (bold blue) is given relative to the target adenosine (±0 position). Editing was performed with plasmid-borne guide RNA and murine Mecp2 in HeLa cells (endogenous ADAR). Data are shown as the mean editing percentage ± s.d. of N = 3 biological replicates.

Very recently, circular LEAPER guide RNAs were demonstrated to recruit endogenous ADARs with moderate editing efficiency and precision in cell culture and in vivo^17,18^. We benchmarked our best circular CLUSTER guide RNAs (Fig. 5a) for the *Mecp2* W104>amber transcript against 111-nt-long symmetric LEAPER guide RNAs (Fig. 5b) and assessed various means of bystander editing suppression (Fig. 5 and Extended Data Fig. 5). Linear (Extended Data Fig. 5) and circularized LEAPER guide RNAs (Fig. 5b,c), using the Tornado expression system were tested by transfection into HeLa cells. As expected, the circular LEAPER-based design gave massive bystander editing at >10 sites, with yields of 20–39% at seven such sites (Fig. 5c, circular LEAPER). Again we first compared G•A mismatching^16^, U depletion^17^ and G•U wobbles to suppress bystander editing at the four bystander sites, which are amenable to G•U wobble base pairing. Given the low number of amenable sites (Fig. 5c) the effects on on-target editing efficiency and bystander editing were comparably low. However, when we aimed to suppress off-target editing at the major ten bystander sites, the on-target yield of a pure G•A mismatch (17% ± 2%) or pure U depletion (16% ± 1%) solution dropped considerably, while a combination of G•U wobble and G•A mismatch (25% ± 2%) outperformed the combination of G•U wobble and U depletion (18% ± 1%) for the best-performing circular LEAPER guide RNA (Fig. 5c). In contrast, a linear CLUSTER guide RNA applying the G•U wobble strategy already achieved bystander-free 38% ± 7% on-target editing (Extended Data Fig. 5f). Furthermore, all optimized circular CLUSTER guide RNAs gave an exceptionally good editing efficiency of >84% on target with very good precision (Fig. 5d), clearly outcompeting all tested LEAPER guide RNAs (Extended Data Fig. 5). G•U wobbles entirely suppressed bystander editing at all three bystander sites where RSs bound the *Mecp2* mRNA. It might be possible that G•A mismatches or U depletion would work similarly well to suppress bystander editing at such sites but this was not tested. There was one remaining site (5′-AAG) at position −5 with a moderate editing yield of 8% that was not amenable for wobble base pairing. Editing at this site would not change the amino acid sequence of MeCP2 yet its editing was reduced by U depletion or G•A mismatching (Fig. 5d).

### In vivo proof-of-concept in a murine Rett syndrome model

**Extended Data Fig. 6:**
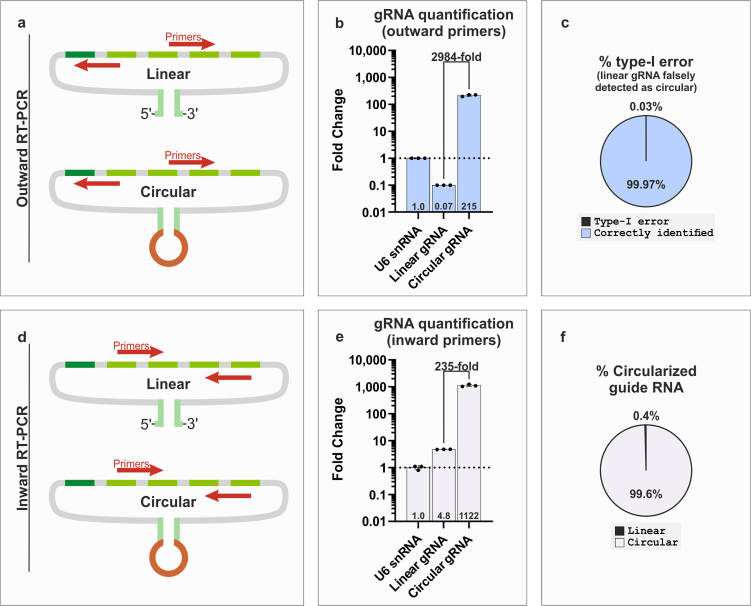
Verifying guide RNA circularization via quantitative RT-qPCR. The circular state of the split-R/G CLUSTER guide RNA (V27.2.4, pTS2108) targeting mMecp2 W104Amber was verified by using quantitative RT-PCR on in vitro samples from transfected HeLa cells. As reference a linear version of the same guide RNA was used. The dataset was normalized to human *RNU6* snRNA. The amplicon of the outward primer pair in a) is 187 bp long and requires reverse transcription and PCR through a strong hairpin structure. It can thus not be directly compared to the inward primer pair in d) which is smaller (75 bp) and does not contain inhibitory secondary structures. **(a)** Binding sites of the outward primer pair, which can only amplify circular guide RNAs. **(b)** Fold change of linear and circular split-R/G CLUSTER guide RNAs (V27.2.4, pTS2108, pTS2228) relative to endogenous human *RNU6* as detected by using the outward primer pair. The 2984-fold difference between linear and circular shows that the endogenous circularization of the guide RNAs though the RTCB ligase (Extended Data Fig. 4) was successful and that the outward primer pair can identify circular guide RNAs with high confidence (low type-I error rate). **(c)** Type-I error rate calculated from b) amounts to only 0.03%, thus 99.97% of circular guide RNAs are correctly identified. **(d)** Binding sites of the inward primer pair, which can amplify both linear and circular guide RNAs. **(e)** Fold change of linear and circular split-R/G CLUSTER guide RNAs (V27.2.4, pTS2108) relative to endogenous human *RNU6* snRNA as detected by using the inward primer pair. The 235-fold difference between linear and circular shows the high level of total guide RNA enrichment achieved after circularization. **(f)** Percentage of successfully circularized guide RNAs calculated from e). Under the assumption that the 235-fold increase detected for circular guide RNAs compared to linear ones solely depends on circularization, 99.6% of all guide RNAs were successfully processed by the ribozymes of The tornado expression system and then ligated by RTCB. Data in b) and e) are shown as the mean editing percentage ± s.d. of N = 3 biological replicates.

Before applying the optimized circular CLUSTER guide RNA in vivo, we verified its successful circularization in cell culture with two sets of reverse transcription (RT)–qPCR primer pairs (Supplementary Fig. 11). The use of an outward primer pair (Extended Data Fig. 6a) allowed us to verify circularization with high confidence (Extended Data Fig. 6b,c), while the use of an inward primer pair (Extended Data Fig. 6d) allowed us to quantify the strong effect (235-fold increase) of circularization on the total guide RNA abundance (Extended Data Fig. 6e), suggesting that the majority of guide RNAs are fully processed (Extended Data Fig. 6f). We chose the PHP.eB serotype for adeno-associated virus (AAV) encapsulation as it allows cargo delivery to the mouse brain after systemic administration^45^. Indeed, this serotype was successfully used by us before to deliver the boxB/λ-ADAR tool into the same Rett syndrome mouse model for mutation correction^44^. The targeting virus encoded the circular CLUSTER guide RNA as displayed in Fig. 5a,d (G•U at G•U-amenable sites). For the nontargeting virus control, the guide RNA’s antisense parts were scrambled.

**Fig. 6:**
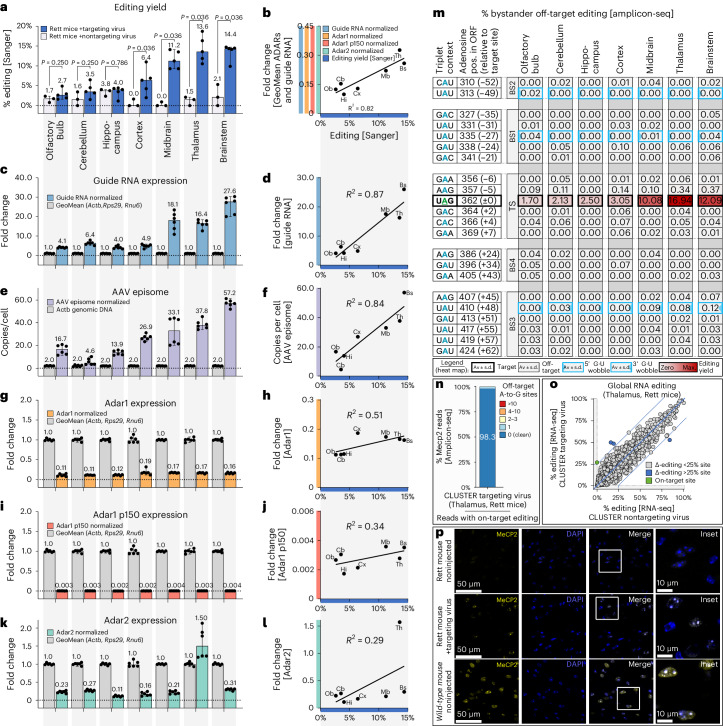
Transcript repair in a mouse model of Rett syndrome using circular wobble-optimized CLUSTER guide RNAs and endogenous murine Adars. **a**, Editing yields in different brain regions, after delivery of the AAV-PHP.eB-encoded guide RNA through retro-orbital injection into Rett mice carrying the *Mecp2* W104>amber mutation and quantification by Sanger sequencing 4 weeks later. **b**, Correlation between the median editing in **a** and the geometric mean of the RT–qPCR targets in **c**,**g**,**i**,**k**. Olfactory bulb, Ob; cerebellum, Cb; hippocampus, Hi; cortex, Cx; midbrain, Mb; thalamus, Th; brainstem, Bs. **c**, Guide RNA expression quantified by RT–qPCR (normalized to the geometric mean of *Actb*, *Rps29* and *Rnu6*. **d**, As in **b** but correlating **a**,**c**. **e**, Absolute quantification of AAV episomes per cell by standard curve qPCR (normalized to *Actb*). **f**, As in **b** but correlating **a**,**e**. **g**, As in **c** but for Adar1. **h**, As in **b** but correlating **a**,**g**. **i**, As in **c** but for Adar1 p150. **j**, As in **b** but correlating **a**,**i**. **k**, As in **c** but for Adar2. **l**, As in **b** but correlating **a**,**k**. **m**, Bystander off-target events at the guide RNA-binding sites and the TS. **n**, All amplicon reads with on-target editing binned according to their number of bystander events (based on Supplementary Fig. 14). **o**, Global RNA editing at 2,533 endogenous sites (coverage *≥* 50 reads, REDIportal^55^). **p**, Thalamus sections stained for MeCP2 and DAPI (nuclei). For **a**, data are shown as the median editing percentage ± 95% CI determined in *n* = 3 mice (nontargeting virus) and *n* = 5 mice (targeting virus). For statistical analysis, a Mann–Whitney *U*-test (two*-t*ailed, nonparametric) was applied. For **c**,**e**,**g**,**i**,**k**,**m**,**n**, data are shown as the median fold change ± 95% CI or median number of copies per cell ± 95% CI determined in *n* = 2 mice (three technical replicates each). The NGS analysis in **o** is based on results from *n* = 1 (nontargeting virus) or *n* = 2 (targeting virus) mice. The results in **p** are derived from *n* = 1 mouse per group. For **b**,**d**,**f**,**h**,**j**,**l**, the *R²* values were determined by simple linear regression.

Mice were treated with 4 × 10^12^ viral genomes by retro-orbital injection and killed 4 weeks later; brain regions were analyzed separately for editing efficiency by Sanger sequencing. Editing levels differed among the seven brain regions, with clearly detectable editing in the midbrain, brainstem and thalamus, with an editing efficiency up to 19% (Fig. 6a). To better understand the key factors for successful editing, we analyzed all seven brain regions for the expression of the guide RNA (Fig. 6c), the AAV episome abundance (Fig. 6e) and the expression levels of all catalytically active murine Adar isoforms: total Adar1 (Fig. 6g), Adar1 p150 (Fig. 6i) and Adar2 (Fig. 6k). The relative expression of guide RNA and Adars were directly compared through RT–qPCR by normalization to the geometric mean of the same three housekeeping genes *Actb*, *Rps29* and *Rnu6* (Supplementary Figs. 12 and 13), while the AAV episome abundance was determined as the number of copies per cell. Unexpectedly, editing yield correlated the least with the Adar levels (Fig. 6h,j,l; *R*^*2*^ = 0.29–0.51), even though Adar expression differed among brain regions, particularly for Adar2 (for example, thalamus versus other brain regions). This indicates that Adar abundance did not limit the editing outcome. The strongest correlation was found between guide RNA expression level and editing yield (Fig. 6d; *R*^*2*^ = 0.87), suggesting that, even under circularization, guide RNA levels still limit on-target editing. Editing also correlated well with AAV abundance (Fig. 6f; *R*^*2*^ = 0.84) and, in most brain regions, guide RNA expression seemed to also correlate well with AAV abundance. However, there were a few exceptions, such as in the cortex, where guide RNA expression was low even though AAV abundance was comparably high. This indicates, in agreement with our previous findings^44^, that the strength of the guide RNA promoter (for example, U6 promoter) or guide RNA stability differs among brain regions, thereby limiting editing even after successful viral delivery. In summary, these data show that the guide RNA’s abundance (determined by its delivery, expression and stability) is the most important factor to achieve high on-target editing in the brain, while Adar levels seem less important. This may instruct future designs of expression cassettes for CNS applications.

**Extended Data Fig. 7:**
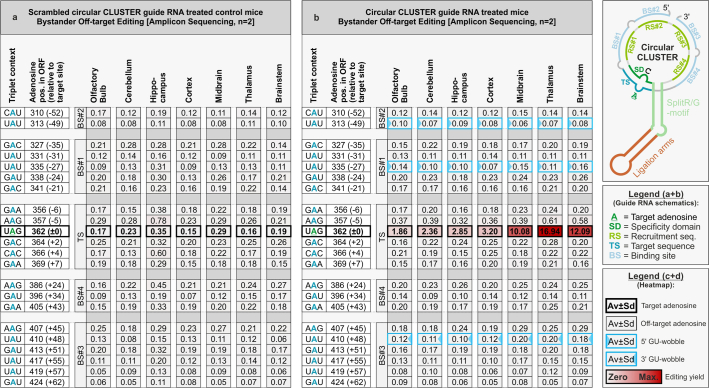
Amplicon sequencing results before background correction. **(a)** Editing heat map of the indicated brain tissue of Rett syndrome mice treated with either the scrambled circular CLUSTER guide RNA virus (nontargeting virus) or **(b)** the circular CLUSTER guide RNA virus (targeting virus). The triplet context for the target-site row is boxed, and the target adenosine underlined and green. All other triplet contexts contain an off-target adenosine in blue. The position of each site is given relative to the transcript and the target adenosine (±0 position). The dataset covers the complete guide RNA binding region including all binding sites (BS) and the target sequence (TS) containing the target adenosine. Data are shown as the mean editing percentage of N = 2 mice.

Particularly in a clinical setting, editing must be efficient and precise, represented by cleanly edited transcripts devoid of unintended recoding events. To evaluate bystander off-target events in the Rett mouse model, we performed deep amplicon sequencing (average read depth of 47,009 and average coverage of 45,991) of the *Mecp2* target transcript in all seven brain tissues. To ensure extensive detection of bystander events, we selected the two mice from the targeting virus group that had given the highest on-target editing yield in the thalamus. The on-target editing results matched very well with the Sanger sequencing (Fig. 6a,m and Extended Data Fig. 7). As controls, two nontargeting virus-treated mice were used. They showed considerably lower background (~0.2%) compared to Sanger sequencing (~5%) (Fig. 6a,m and Extended Data Fig. 7). Consequently, this now enabled us to measure on-target editing yields of 1.7–3.0% with high confidence in the olfactory bulb, cerebellum, hippocampus and cortex (Fig. 6m). Notably, bystander editing was hardly detected in any brain region. At three positions (−49, −27 and +48), G•U wobble base pairs were applied to suppress bystander editing. At positions −49 and −27, this was very successful; no bystander editing was detectable. At position +48, there may have been up to 0.12% bystander editing in the brainstem but 100-fold below the on-target editing yield in that tissue. Only one bystander was detected with high confidence. This was the unresolved, silent bystander site at position −5 that was already discovered in cell culture (Fig. 5d; 8% ± 4%). Here we detected up to 0.37% bystander editing in vivo in the brainstem (Fig. 6m). If they appear at many sites, even bystander events with low editing yield may sum up to interfere with on-target editing. To address this potential issue, we studied how often an on-target edited read is damaged by an additional bystander edit. We found that 98.3% of the ~10,000 detected on-target edited reads were completely bystander free (Fig. 6n and Supplementary Fig. 14), highlighting the impressive degree of editing precision achieved in the in vivo proof of concept.

**Extended Data Fig. 8:**
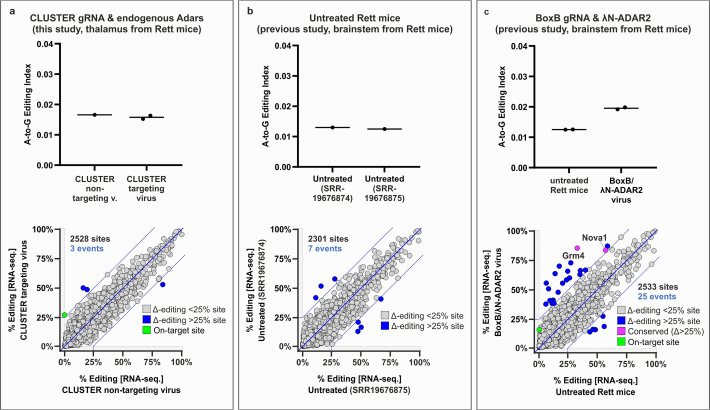
Analysis of global off-target events by RNA-seq. **(a)** Rett mice injected with wobble-optimized circular CLUSTER guide RNA (targeting virus) versus scrambled guide RNA (nontargeting virus). **(a) (top)** A-to-G RNA editing index in coding sequences. An index of 1 means 1% of RNA nucleotides mapped to genomic adenosines are guanosines. The index was stable (no change in global editing activity due to the targeting guide RNA). **(a) (bottom)** Global RNA editing at 2,528 endogenous sites (coverage ≥ 50, REDIportal^55^). With only 3 sites showing > 25% Δ-editing (beyond blue dotted lines), no clear off-target editing was detectable in comparison to panel b. **(b)** Reanalyzed RNA-seq data (previous study^44^). With respect to the tissue selected in panel c), data from the brainstems of two untreated Rett mice are compared. **(b) (top)** Both untreated mice show a stable editing index. **(b) (bottom)** Global RNA editing at 2,301 endogenous sites (coverage ≥ 50). These sites are also displayed in panel a) (bottom) and c) (bottom). The 7 sites showing > 25% Δ-editing are considered normal mouse to mouse and technical variability. **(c)** Reanalyzed RNA-seq data (previous study^44^). Rett mice injected with boxB/λN-ADAR2 virus were compared to untreated ones. The ADAR2 catalytic domain in the virus is from the native ADAR2 protein. **(c) (top)** The A-to-G index increase by 0.007 in the coding space reflects a substantial and somewhat concerning increase in global editing, probably due to the overexpressed ADAR2 catalytic subunit. **(c) (bottom)** Global RNA editing at 2,533 endogenous sites that include the 2,528 sites shown in panel a) (coverage ≥ 50 reads). 25 sites showing > 25% Δ-editing after injection of the λN-ADAR2 targeting virus. This is in clear contrast to the very high precision seen for CLUSTER guide RNAs recruiting endogenous ADARs in a) (bottom) or the untreated mice in b) (bottom). Importantly, the potential off-target sites included two ADAR2-specific, highly evolutionary conserved sites in GRM4 (glutamate transmission) and NOVA1 (splicing)^49^. Data in a), b) and c) are based on results from N = 1-2 mice per group. Thalamus and brainstem were selected for showing the highest editing yields in their respective study.

Next, we evaluated the global editing precision by interrogating transcriptome-wide RNA sequencing (RNA-seq) data collected from the thalamus of targeting and nontargeting virus-treated Rett mice (Fig. 6o and Extended Data Fig. 8a). As before, we selected the two mice from the targeting virus group that gave the highest on-target editing yield in the thalamus with Sanger sequencing. First, we applied the RNA-editing index method that monitors changes in global RNA-editing levels in a highly unbiased manner and focused the analysis on the particularly critical coding sequence space^46^. The A-to-G RNA-editing indices were nearly identical in both groups (Extended Data Fig. 8a), indicating that the global editing activity was overall not affected by the presence of the targeting guide RNA. Second, we tried to detect differentially edited sites between the two conditions (Fig. 6o), similar to a previous study^15^. However, compared to the same analysis performed with untreated Rett mice (Extended Data Fig. 8b), we detected no clear off-target sites. Only three sites fell slightly outside of the ±25% Δ-editing margin but this was likely because of normal variability among mice. None of the three sites were located in the coding region and none of them were complementary to the guide RNA. Third, we tried to detect off-target events in a candidate approach and searched in silico throughout the whole murine genome for transcripts with 20-nt similarity (with up to one mismatch) to the guide RNA-binding regions. We identified 65 potential sites; however, only four of these sites were sufficiently expressed (≥20 reads of coverage) to be evaluated and no off-target editing was detected at any of them. Overall, we were unable to identify any global off-target events, excluding mouse-to-mouse variability, which suggests a very high precision of our approach on the transcriptome-wide level.

RNA base-editing approaches that apply the overexpression of an engineered editase, such as λN-ADAR2, typically suffer from substantial, editase-dependent global off-target events^46–48^. In our recent study—from which the noninjected mice data were derived (Extended Data Fig. 8a), we also detected numerous off-target events even though the native ADAR2 deaminase domain was fused to the λN peptide. We revisited this published RNA-seq data^44^ to compare with the CLUSTER generated data. The boxB/λN-ADAR2 data were generated using the same experimental setup (mouse model, AAV serotype and application route) as in this CLUSTER study. We ran the data in parallel through the same next-generation sequencing (NGS) analysis pipelines. First, we evaluated the same 2,533 endogenous RNA-editing sites (with coverage ≥ 50 reads) that we evaluated for the CLUSTER approach, as shown in Fig. 6o. Again, we selected the tissue with the highest on-target editing yield, which was the brainstem in this study^44^. We found a notable number (25) of potential off-target sites exhibiting a Δ-editing margin above 25% (Extended Data Fig. 8c). Importantly, the identified events included two evolutionary highly conserved, Adar2-specific editing sites in glutamate metabotropic receptor 4 (GRM4) and neuro-oncological ventral antigen 1 (NOVA1)^49^, for which the change in editing level could have functional impact. Second, we analyzed the editing index in the coding sequence space and found a considerable increase in the index in the presence of the λN-ADAR2 deaminase (Extended Data Fig. 8c). Together, this shows, in accordance with the literature^47^, that the harnessing of endogenous ADAR is more precise on the transcriptome-wide level than the ectopic expression of engineered ADAR effectors, whether hyperactive or native.

**Extended Data Fig. 9:**
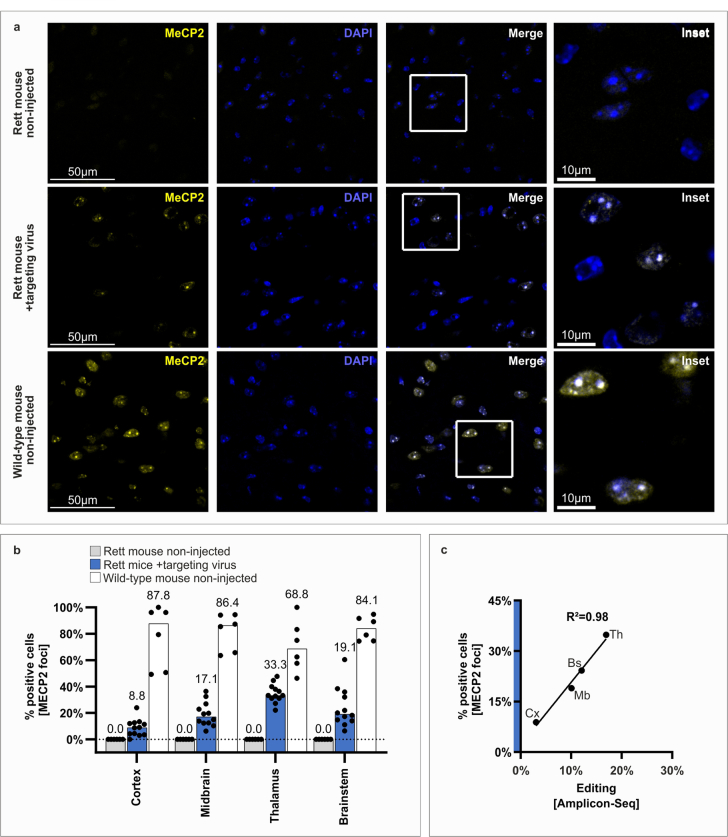
Immunohistochemical evidence supporting repair of MeCP2 protein and function after treatment of Rett mice with the circular CLUSTER guide RNA. **(a)** Confocal images of sections of thalamus stained for MeCP2 protein and DAPI to indicate nuclei. MeCP2 puncta staining, co-localizing with DAPI, only seen in middle and lower panels indicating restoration of endogenous protein expression. Nuclei (DAPI) were pseudo-colored to highlight colocalization puncta. **(b)** Each bar represents the total percentage of MeCP2 protein foci positive cells relative to DAPI positive cells in each mouse group. Each datapoint represents one microscopic field of view. Non-injected Rett mice show background levels of immunofluorescence^44^. The distribution of positive nuclei in non-injected wild-type mice reflects the cellular heterogeneity within the brain. Analysis was performed by confocal imaging in three sections from each brain region indicated in the figure. We selected the four brain regions with the highest editing yields. Data is shown as the median percentage of cells showing MeCP2 protein foci ± c.i. (confidence interval 95%) from N = 2 mice per bar, as counted in 3 (non-injected Rett mice) or 6 (injected Rett mice) fields of view per animal. **(c)** High correlation between the median editing yields (amplicon sequencing) and the percentage of cells with MeCP2 protein foci in the four brain regions in a). The abbreviations stand for Cortex (Cx), midbrain (Mb), thalamus (Th), and brainstem (Bs). Data is shown as one datapoint per brain region. Each datapoint represents the median result of the respective tissue from N = 2 mice injected with targeting virus per readout. For technical reasons the correlated editing yields and protein foci percentages are from different animals.

Lastly, we studied the restoration of MeCP2 protein expression and function upon treatment using the circular CLUSTER guide RNA (targeting virus). In the noninjected Rett mice, MeCP2 expression was not detectable by single-cell immunohistochemistry^44^ in the thalamus, as the mutation resulted in an unstable protein (Fig. 6p and Extended Data Fig. 9). In clear contrast, MeCP2 was restored in ~33.3% ± 4% (median ± 95% confidence interval (CI)) of the cells in the thalamus of the treated brain. Importantly, the restored MeCP2 protein was localized in heterochromatic foci inside the nucleus, which is an accepted proxy for its in vivo binding ability to methylated DNA^50,51^ and very similar to the positive control mouse where up to 100% of the cells showed MeCP2 protein associated with heterochromatin (Fig. 6p and Extended Data Fig. 9a,b). The abundance of foci increased with increasing editing yields over the evaluated brain tissues (Extended Data Fig. 9c). The results suggest that MeCP2 protein and its function are restored in cells where guide RNA is delivered and expressed.

## Conclusions and outlook

The strategic placement of G•U wobble base pairs is a novel approach to improve editing precision and is widely applicable to RNA base-editing tools, including the LEAPER and CLUSTER approaches, λN-ADAR, Cas13-ADAR and chemically modified ASOs. The 5′-G•U wobble is particularly powerful to suppress editing at all four 5′-UAN triplets, which account for the largest burden of bystander editing. The suppressive effect of the G•U wobble is very strong and, at the same time, restricted to the direct 5′ and 3′ neighboring bases. In this regard, the G•U wobble differs from alternative solutions such as G•A mismatch or U depletion and makes the wobble strategy particularly powerful to suppress bystander editing close to an on-target site. As not all bystander-prone triplets are amenable to the G•U wobble strategy, we suggest to combine them with other strategies of bystander suppression, particularly with G•A mismatches at sites not amenable for the wobble strategy. Typically, but not always, a combination of wobble base pairing with G•A mismatching resulted in a better balance of editing efficiency over editing precision than the use of G•A mismatching only.

**Extended Data Fig. 10:**
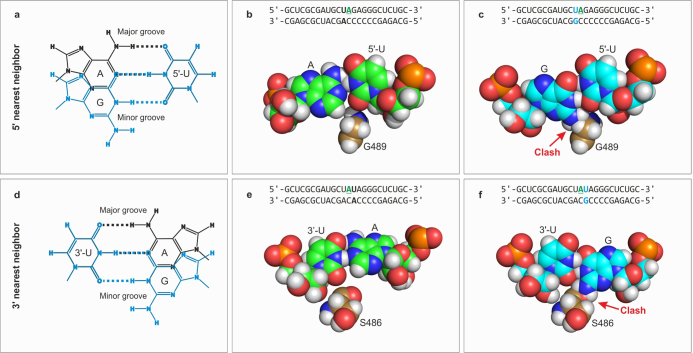
Proposed mechanisms for the suppressive effect of G·U wobble base pairs on RNA editing when placed at nearest neighbor sites. **(a)** Superposition of a 5´ G U wobble base pair (blue) on top of a Watson-Crick AU base pair (black) arranged according to panel b) and c). The shifted Guanine (blue) results in the projection of an exocyclic amino group into the minor groove, which is a distinctive feature of the GU wobble base pair^32^. **(b)** hADAR2d/Bdf2 crystal structure indicating the close proximity of the 5’ nearest neighbor AU base pair and Glycine 489. The position of the observed base pair (black, bold) and the target adenosine (green, bold, underlined) is highlighted in the Bdf2 duplex sequence. **(c)** A 5´ G·U wobble base pair fitted into the hADAR2d/Bdf2 crystal structure at the 5’ nearest neighbor position predicts a steric clash between the exocyclic amino group of the Guanine and the Glycine 489. The clash would require extensive accommodation of the complex and thus cause structural perturbations, that might negatively affect the deamination rate. The position of the observed wobble base pair (blue, bold) and the target adenosine (green, bold, underlined) is highlighted in the Bdf2 duplex sequence. **(d)** As a) but arranged according to panel e) and f). **(e)** As b) but showing the 3’ nearest neighbor AU base pairs proximity to Serine 486. The AU base pair was fitted into the position of the original CG base pair of the hADAR2d/Bdf2 crystal structure. **(f)** As c) but showing the Guanosine of the 3’ G·U wobble base pairs clashing with Serine 486. The 3´ G·U wobble base pair was fitted into the position of the original CG base pair of the hADAR2d/Bdf2 crystal structure.

It is intriguing to speculate about the mechanism that underlies the suppressive effect of wobble base pairs. The ADAR deaminase domain strongly favors 5′-UAN triplets over 5′-CAN and 5′-GAN. A recent crystal structure analysis of the ADAR2 deaminase in complex with a dsRNA substrate shed light on the molecular basis of the nearest neighbor preference^52^ and identified a specific steric clash between the backbone of G489 and the exocyclic amino group of the guanine of the 5′ neighboring G≡C base pair (Extended Data Fig. 10a–c). Thus, replacing a 5′-A=U base pair with a 5′-G•U wobble base pair would again introduce a sterically demanding exocyclic amino group in the minor groove of the dsRNA substrate. Furthermore, the guanine base is likely further shifted toward the minor groove given the noncanonical hydrogen bond pattern in a G•U wobble base pair^32^. This might explain the very strong and located suppressive effect of the 5′ G•U wobble base pair (Extended Data Fig. 10a–c). A similar clash of the exocyclic amino group with S486 in the minor groove might explain the suppressive effect of the G•U wobble base pair at the 3′ nearest neighbor position (Extended Data Fig. 10d–f).

Overall, wobble base pairs create structural perturbations within the RNA substrate that have consequences for the activity of human ADAR. Moreover, regarding natural substrates, wobble base pairs seem to be part of a structural layer of control that may not yet be fully recognized. Nevertheless, we could deduce simple rules for the rational design of *trans*-acting guide RNAs for targeted RNA base editing, which achieved very high editing efficiency with very good editing precision. While broadly applicable in many approaches, the G•U wobble strategy proved helpful to design CLUSTER guide RNAs by means of expanding the sequence space for the computation of in silico optimized circular CLUSTER guide RNAs. In a direct comparison with LEAPER guide RNAs of similar length, we could show that CLUSTER guide RNAs can be designed with clearly advantageous efficiency and precision. It remains open whether G•A mismatching or U depletion could serve similarly well to optimize CLUSTER guide RNAs. However, given the short length of an individual RS (15–20 nt), we speculate that wobble base pairs might work better in highly A-rich binding regions where multiple G•A mismatches or bulged A bases might interfere with RS binding.

The AAV-mediated delivery of the most advanced circular CLUSTER guide RNA demonstrated effective (up to 19%) transcript repair of the *Mecp2* W104>amber mutation in a mouse model of Rett syndrome and represents a successful in vivo recruitment of endogenous Adars for site-directed RNA editing in the CNS. With a median editing yield of ~14.4%, it also represents a high in vivo editing yield for restoring a disease-causing mutation in a murine model of human disease using only endogenous Adars. Furthermore, deep amplicon sequencing showed that on-target editing was achieved with very high precision regarding bystander editing. More than 98% of the detected on-target-edited reads were free from bystander edits. Only one (silent) bystander edit was detected with high confidence, albeit with a yield of only 0.1–0.37%. Transcriptome-wide RNA-seq confirmed a stable A-to-G RNA-editing index and no off-target sites in the transcriptome could be identified. This shows that editing can be achieved with very high precision and under the control of bystander editing in vivo. Notably, it is highly promising to see that endogenous Adar can be harnessed to obtain similar editing levels to those achieved with the exogenous boxB/λN-ADAR tool in the same mouse model before^44^. Interestingly, the trends regarding the varying editing efficiencies in different brain regions were also comparable between endogenous and exogenous ADAR approaches. A side-by-side analysis of on-target editing, AAV episome abundance as well as guide RNA and Adar isoform expression data^53^ suggests that not endogenous Ader expression but rather the virus-mediated guide RNA expression limited editing efficiency in the CNS of the murine Rett model. Thus, future improvements of AAV capsid engineering, application routes, guide RNA expression cassettes and/or guide RNA stability promise to enhance RNA base editing even further, breaking ground for clinical applications. Overall, harnessing endogenous ADAR with permanent AAV-driven CLUSTER guide RNAs in the CNS is an important next step toward the development of novel drug modalities that fight neurological diseases.

## Methods

### The GuideRNA-Forge tool

The GuideRNA-Forge tool was programmed in Python (version 3.9). It is based on our previously published ‘recruitment cluster finder’ tool and, thus, follows the core principles summarized previously^15^. GuideRNA-Forge uses a JavaScript Object Notation file (.json) to allow for the modular input of guide RNA parts and, thus, makes the power of in silico optimization available to any conceivable multivalent design, including circular guide RNAs. An example .json file including all relevant input values or strings for the generation of a circular CLUSTER guide RNA is provided in the GitHub archive.

The filters that identify binding regions for RS placement, while excluding RSs that contain unwanted sequences (for example, immunogenic, cytotoxic or guide RNA-destabilizing motifs) are now customizable. A combination of regular expressions (regex) and modular filter building blocks allows for quick assembly of user defined filters. The filters used in this study to generate G•U wobble-containing circular CLUSTER guide RNAs are provided in the GitHub archive. Note that the filters do not apply G•U wobbles to your guide RNA. Instead, they allow binding regions that contain A bases in a 5′-GAB, 5′-UAB, 5′-BAU (B = C, G or U), 5′-KAAU (K = G or U) and 5′-CAC sequence context, as well as A bases at the edges of binding regions. As not all A bases that allow for the G•U wobble solution (5′-UAB, 5′-BAU and 5′-KAAU) are necessarily edited, we recommend determining actual bystander off-target sites experimentally. G•U wobbles can then be strategically placed adjacent to problematic A bases.

In contrast to the previous version, the GuideRNA-Forge tool can identify multiple RSs within one potential binding region, as well as search in both the 5′ and the 3′ directions starting from the SD-binding site. Furthermore, the processing speed of recombination and folding (ViennaRNA package^54^) was drastically increased by implementing multithreading.

The resulting guide RNAs are scored for proximity of their binding sites andminimal secondary structure. CLUSTER guide RNAs with a high median score consist of RSs that bind in close proximity to each other on the target transcript and are less likely to engage in unproductive folding of their antisense part or misfolding of the ADAR-recruiting domain.

### Vector design (guide RNA and cDNA)

The guide RNA and *cis*-acting reporter inserts including the necessary overhangs were created by hybridization and phosphorylation of oligonucleotides. The *cis*-acting editing reporters were created by cloning hybrid oligonucleotide inserts into our pcDNA3.1-based *eGFP* expression plasmid (pTS58) using ApaI and AgeI as described in Supplementary Note 1. Linear CLUSTER (using HindIII and BbsI into pTS1033), LEAPER (using HindIII and BamHI into pTS1033) and boxB (using HindIII and BamHI into pTS1033) guide RNAs were created as previously described^15^. DR guide RNAs were created by cloning hybrid oligonucleotide inserts encoding the antisense part into the PspCas13b crRNA backbone (Addgene plasmid 103854) using BbsI. The DR hairpin is already present in the backbone and must not be a part of the insert.

As the first step to create circular CLUSTER or LEAPER guide RNAs expressed by the U6 promoter, a modified Tornado expression cassette was created by gene synthesis (Thermo Fisher, GeneArt gene synthesis) and cloned into the backbone of our in-house editing vector ‘pEdit1.2’ (ref. ^37^) using NheI and AgeI, thereby replacing the ADAR2 expression cassette. The resulting Tornado OHA vector U6 (pTS1541) was used as the backbone for subsequent circular guide RNA cloning. The hybrid oligonucleotide guide RNA inserts were cloned into the latter backbone using BbsI. The cloning strategy is described in Supplementary Note 2. As a first step for cloning of circular LEAPER guide RNAs with flexible poly(AC) RNA linkers (AC50), the latter were synthesized as a gene block and cloned into pTS1541 using BbsI, resulting in the CircLEAPER AC50 cloning vector (pTS2508). Subsequent cloning of gene blocks containing circular LEAPER guide RNAs destined for the AC50 context was performed using BbsI into pTS2508. For the expression of circular guide RNAs by the EF1α promoter, the Tornado OHA vector EF1α (pTS1593) was used for subsequent circular guide RNA cloning analogous to pTS1541. For direct benchmarks between EF1α and U6 constructs, the hybrid oligonucleotide guide RNA inserts were cloned into pTS1593 (EF1α) and pTS1790 (U6) using BbsI. For stable genomic integration of linear or circular guide RNAs by the PiggyBac system, a transposase expression plasmid and the plasmid pTS1896 (transposon, U6, circular LEAPER guide RNA *RAB7A*), pTS1897 (transposon, U6, linear LEAPER guide RNA *RAB7A*), pTS1899 (transposon, EF1α, circular LEAPER guide RNA *RAB7A*) or pTS1900 (Transposon, EF1α, linear LEAPER guide RNA *RAB7A*) were used.

For AAV production, the lead circular CLUSTER guide RNA targeting the *Mecp2* W104>amber transcript was cloned into the pAAV-GFP backbone (Cell Biolabs). The cloning strategy is described in Supplementary Note 3.

The open reading frame (ORF) sequence of murine *Mecp2* W104>amber was supplied by the Mandel Lab (Vollum Institute, Oregon Health and Science University) and cloned into the pEGFP-N3 backbone (Clonetech) using EcoRI and KpnI under the control of a cytomegalovirus (CMV) promoter and terminated by an SV40 poly(A) signal. The human *PEX1*^G843D^ plasmid was supplied by the Dodt Lab (Interfaculty Institute of Biochemistry and Cell Biochemistry, University of Tübingen). The cassette is under the control of an EF1α promoter and terminated by an EF1α poly(A) signal. The dual-luciferase reporters and the disease-relevant cDNA constructs *AHI1* W725>amber, *BMPR2* W298>amber and *COL3A1* W1278>amber were created as previously described^15^. The sequences of all cloned products were verified by Sanger sequencing. The ORFs and amino acid sequences of *Mecp2*, *PEX1* and the engineered editases are given in Supplementary Note 4. Plasmid maps of all mentioned pTS plasmids are given in Supplementary Note 5 or can be found in the literature^37^. Further details can be found in the Supplementary Information.

### Analysis of RNA editing

A-to-I editing yields were quantified from Sanger sequence traces using SNAP-Gene (version 4.2.11). The relative height of the signal of G was compared to the sum of G+A, as described earlier^37^. If a reverse primer was used for sequencing, C and T peaks were treated accordingly. For better comparability, on-target editing yields of the same target with different guide RNAs were quantified using the same sequencing primer. Off-target editing had to be evaluated with different sequencing primers in most cases because of large distances between the guide RNA-binding sites. Only the cleanest reads were used for off-target evaluations, whereby G peaks below background were counted as 0% off target. Editing events with yields below 10% were background-corrected with the negative control.

### Structural analysis

A-U base pair structures and ADAR amino acid residue structures were obtained from the substrate-bound ADAR2 dimer crystal structure (Protein Data Bank (PDB) 5HP2)^56^, while G•U and U•G wobble base pairs were obtained from the NMR structure of the Gria2 stem loop RNA (PDB 2L2J)^57^. Structures were fitted and imaged with the open-source PyMOL Molecular Graphics System (version 2.5.0)^58^. The results are reported in Extended Data Fig. 10.

### Cell culture

#### General

The used cell cultures were grown in DMEM (Thermo Fisher, 41965062) supplemented with 10% FBS (Thermo Fisher, 10270106) and kept in an incubator at 37 °C and 5% CO_2_.

#### Cell line choice

In our laboratory, HeLa cells have undergone comprehensive characterization, including assessment of ADAR1 expression, knockdown efficiency, response to interferon treatment and optimization of transfection conditions. Consequently, HeLa cells were used for most RNA base-editing experiments, particularly those involving editing of overexpressed exogenous transcripts. However, when targeting endogenous transcripts, achieving a high percentage of positively transfected cells is crucial for robust results. Each untransfected cell harbors the unedited transcript, which can adversely impact the overall readout. HEK293FT cells offer superior transfection efficiency and were, thus, used for targeting endogenous transcripts. By integrating a single genomic copy of ADAR1 p110, p150 or ADAR2 under the control of an CMV Tet-On promoter into Flp-In T-REx cells, we generated a set of cell lines that can be used to characterize how well dsRNA substrates such as bound guide RNAs or editing reporters are accepted by specific ADAR isoforms. Notably, the ADAR expression levels in these Flp-In cell lines exceeded typical endogenous levels, which increased bystander editing and facilitated the discovery of suppressive effects of wobble base pairs on bystander off-target edits.

#### ADAR Flp-In T-REx cells

A total of 2.5 × 10^5^ ADAR1 p110 or p150 Flp-In T-REx cells or 3 × 10^5^ ADAR2 Flp-In T-REx cells were seeded on poly(dLys)-coated 24-well plates in 500 µl of DMEM + 10% FBS + 10 ng ml^−1^ doxycycline. After 24 h, cells were transfected with 1,300 ng of *cis*-acting editing reporter plasmid (NucleoSpin Plasmid Transfection-grade, Macherey Nagel, 740490) using a 1:3 ratio of Lipofectamine 2000 (Thermo Fisher, 11668019). Then, 72 h after transfection, the cells were harvested. As the readout method, Sanger sequencing was used. Results are reported in Fig. 1, Extended Data Figs. 1 and 3 and Supplementary Figs. 1 and 4.

#### HEK293FT cells

HEK293FT cells (6 × 10^4^) were seeded in 24-well plates in 450 µl of DMEM + 10% FBS. After 24 h, cells were transfected with 1,200 ng of guide RNA plasmid (NucleoSpin Plasmid Transfection-grade, Macherey Nagel, 740490) using a 1:3 ratio of FuGene 6 (Promega, E2691). Then, 48 h after transfection, cells were harvested. As the readout method, Sanger sequencing was used. Results are reported in Extended Data Fig. 2 and Supplementary Figs. 2, 6 and 7.

#### HeLa cells (experiments using encodable guide RNAs)

A total of 0.8 × 10^5^ HeLa cells were seeded in 24-well plates in 500 µl of DMEM + 10% FBS. Then, 24 h after seeding, cells were transfected with a total of 1,000 ng (800 ng of LEAPER or CLUSTER guide RNA plasmid and 200 ng of target-encoding plasmid) or a total of 1,200 ng (800 ng of boxB or DR guide RNA plasmid, 200 ng of target-encoding plasmid and 200 ng of λN-ADAR or Cas13-ADAR editase encoding plasmid) per well using a 1:1.5 ratio of plasmid to Lipofectamine 3000 ratio. Then, 72 h after transfection, cells were harvested. As the readout method, Sanger sequencing was used. Results are reported in Figs. 2–5, Extended Data Figs. 3, 5 and 6 and Supplementary Figs. 5 and 11.

#### HeLa cells (experiments using an ASO guide RNA targeting the *PEX1*^G843D^ transcript)

A total of 1 × 10^5^ HeLa cells were seeded in 24-well plates in 500 µl of DMEM + 10% FBS. Then, 24 h after seeding, cells were transfected with 300 ng of target *PEX1*^G843D^-encoding plasmid per well using a 1:3 ratio of plasmid to FuGene 6. Then, 48 h after seeding, the chemically modified guide RNAs were forward-transfected with 25 pmol of guide RNA and 1.5 µl of Lipofectamine RNAiMAX reagent (Thermo Fisher, 13778150) per well. Then, 24 h after guide RNA transfection, cells were harvested. The one-step RT–PCR (*PEX1* primer set A) was followed by a nested PCR (*PEX1* primer set B) for this specific target. As the readout method, Sanger sequencing was used. Results are reported in Fig. 3f.

#### HeLa cells (stable integration of guide RNAs)

A total of 1.5 × 10^5^ HeLa cells were seeded in 12-well plates in 1 ml of DMEM + 10% FBS. Then, 24 h after seeding, cells were transfected with a total of 2,000 ng (1,600 ng of PiggyBac transposon vector containing the guide RNA expression cassette and 400 ng of pTS687 PiggyBac transposase vector) using a 1:3 ratio of plasmid to FuGene 6 (Promega, E2691). Then, 24 h after transfection, cells were transferred to 10-cm dishes and, after another 48 h, selection was started by addition of 5 µg ml^−1^ puromycin. Puromycin was refreshed on day 6 after transfection and selection was stopped 8 days after transfection. Each time when the cells were split, an aliquot was taken aside and used for RNA extraction and editing analysis. Results are reported in Supplementary Fig. 8.

#### HeLa cells (experiments using the dual-luciferase reporter system)

A total of 2.4 × 10^4^ HeLa cells were seeded in 96-well plates. Cells were transfected 24 h after seeding with 160 ng of guide RNA plasmid and 40 ng of dual-luciferase reporter per well using a 1:1.5 ratio of plasmid to Lipofectamine 3000 and a 1:2 ratio of plasmid to P3000 reagent. For equimolar comparisons among differently sized guide RNA plasmids, their amount was adjusted accordingly and filled to a total of 160 ng with an empty pcDNA3.1 plasmid. The luciferase assay was performed 48 h after transfection. Results are reported in Supplementary Figs. 9 and 10.

#### Editing readout using dual-luciferase activity

Dual-luciferase activity was measured with the Dual-Luciferase Reporter Assay System (Promega) according to the manual in 96-well plates. Cells were washed with PBS and then lysed in 1× passive lysis buffer (35 µl per well) while shaking for 15 min at 700 r.p.m. at room temperature. Cell lysate (30 µl per well) was transferred to a LumiNunc 96-well plate (VWR, 732-2696) and measured in a Spark 10M plate reader (Tecan) using the dual-luciferase reporter assay reagents (35 µl per well) with an autoinjector. The attenuation standard settings (OD-none) of the Tecan reader were used. Each measurement was performed for 10 s, starting 5 s after injection. Per treatment, five biological replicates were analyzed, each measured in one technical replicate. For data processing, measured blank values (background) were subtracted from samples and controls and then all firefly values were divided by the corresponding *Renilla* values. The resulting normalized firefly activity of all samples was then set in ratio to the positive control to obtain the restored normalized firefly activity as a percentage.

#### Editing readout using Sanger sequencing (total RNA from cell lines)

Cells were harvested in RLT buffer (Qiagen, 79216), followed by RNA isolation using either the Monarch RNA cleanup kit (NEB, T2030L) or the RNeasy Mini RNA isolation kit (Qiagen, 74104). DNase I digestion was performed according to the manual using NEB DNase I (NEB, M0303S). One-step RT–PCR was performed using the Biotechrabbit one-step RT–PCR kit (Biotechrabbit, BR0400102) for regular substrates. For difficult substrates (for example, the murine *Mecp2* W104>amber transcript), the OneTaq one-step RT–PCR kit (NEB, E5315S) was used. All samples were mixed with nuclease-free water (10 µl) and heated to 90 °C for 2 min immediately before RT–PCR. If necessary, a sense-oligo (1 µl, 10 µM) corresponding to the used guide RNA (Supplementary Table 2) was included in the volume. For the murine *Mecp2* W104>amber transcript, the heating step was performed at 95 °C for 3 min under 12.5% DMSO (5% final concentration in RT–PCR mix). After TAE buffer–agarose gel electrophoresis and PCR cleanup (NucleoSpin Gel and PCR cleanup kit, Macherey Nagel, 740609), Sanger sequencing (Microsynth AG) was performed.

#### RT–qPCR experiments

RNA isolation was performed using either the Monarch RNA cleanup kit (NEB, T2030L) or the RNeasy Mini RNA isolation kit (Qiagen, 74104) according to the manufacturer’s protocol. DNase digestion was performed according to the manufacturer′s protocol (rigorous two-step incubation treatment) using the Turbo DNase Kit (Thermo Fisher, AM1907). RT with 500 ng of RNA per sample was performed using the high-capacity cDNA RT kit (Thermo Fisher, 4368814), followed by PCR cleanup using the NucleoSpin Gel and PCR cleanup kit (Macherey Nagel, 740609). RT–qPCR was executed in an Applied Biosystems QuantStudio 7 Pro real-time PCR system (96-well or 384-well qPCR plate, 20 ng or 4 ng of cDNA (10 or 2 ng μl^–1^) per well). The Fast SYBR-Green mastermix (Applied Biosystems, 4385612) was used according to the manufacturer’s protocol (10 µl or 5 μl of SYBR-Green mix, 7.2 µl or 2.6 μl of nuclease-free water and 0.4 μl or 0.2 µl of each primer). Samples and TE buffer negative controls were measured in three technical replicates. ΔΔ*C*_*t*_ calculations were performed as previously described^15^. Amplification efficiency and melting curves were analyzed for each new primer pair using a cDNA dilution series (Supplementary Fig. 11). For normalization, one or more housekeeping genes from the following list were used: human U6 small nuclear RNA (snRNA; *RNU6*), murine U6 snRNA (*Rnu6*), murine β-actin (*Actb*) and murine ribosomal protein 29 (*Rps29*). Results are reported in Fig. 6 and Supplementary Figs. 11–13.

#### qPCR experiments (AAV episome copy number)

Genomic DNA isolation from murine brain tissue was performed using the QIAamp DNA Micro Kit (Qiagen, 56304) according to the manufacturer’s protocol. qPCR was executed in an Applied Biosystems QuantStudio 7 Pro real-time PCR system (384-well qPCR plate, 4 ng of genomic DNA (gDNA; 2 ng μl^–1^) per well). The Fast SYBR-Green mastermix (Applied Biosystems, 4385612) was used according to the manufacturer’s protocol (5 μl of SYBR-Green-Mix, 2.6 μl of nuclease-free water and 0.2 μl of each primer). Samples and AE buffer negative controls were measured in three technical replicates. ΔΔ*C*_*t*_ calculations were performed as previously described^15^. For normalization, the murine *Actb* housekeeping gene was used. The vector copy number per μg of DNA was determined by absolute quantification using a standard curve. The templates for the standard curve were prepared according to the manufacturer’s protocol (Takara, 6233). For the conversion of vector copy number per μg of DNA into copies per cell, 6.030 pg of gDNA was assumed per male C57BL/6 nucleus according to a previous study^59^. Results are reported in Fig. 6.

### Animal experiments

#### Animal studies

All animal procedures were approved by the Institutional Animal Care and Use Committees of Oregon Health and Science University. A total of ten *Mecp2*^G311A^-carrying male mice (W104X) were killed during this study. Three mice were injected with the nontargeting virus and seven were injected with the targeting virus. From these seven animals, five were used for RT–qPCR and Sanger, amplicon and RNA-seq. The remaining two mice were used for MeCP2 immunohistochemistry.

#### *Mecp2* 311G>A husbandry and genotyping

All mice were housed in conventional laboratory housing under controlled humidity (target value, 44%; minimum, 24%; maximum, 64%), temperature (target value, 21 °C; minimum, 18 °C; maximum, 24 °C) and lighting (12-h light, 12-h dark period) with free access to food (regular mouse chow) and water. The *Mecp2*^G311A/+^ mice were maintained by crossing to pure wild-type C57BL/6J mice. Genotyping was performed using primers specific for the *Mecp2*^G311A^ allele. Separately, sex was determined using PCR primers specific for the X and Y chromosomes (Supplementary Table 2).

#### Design of AAV-encoded guide RNAs

The targeting virus encoded the circular *Mecp2-*targeting CLUSTER guide RNA V27.2.4 (Supplementary Table 1). The latter consisted of a TS and four RSs of 20 nt length each, a split-R/G V3 ADAR-recruiting domain, a ligation stem and two ribozymes required for its circularization (Extended Data Fig. 4). For the nontargeting guide RNA virus, the sequences of all four RSs and the targeting sequence were scrambled, while A linkers, the ADAR recruiting motif and ribozymes required for circularization were unchanged (Supplementary Table 1).

#### Viral vector preparation

The AAV-PHP.eB vectors were produced by the Penn Vector Core Facility (Perelman School of Medicine, University of Pennsylvania) and titered using droplet digital PCR. Aliquots were stored at −80 °C before use.

#### Viral injections

P30–P34 male mice were deeply anesthetized with 3% isofluorane (v/v) and placed on a prewarmed surface. For each animal, a 100-μl volume containing 4E12 viral genomes was injected into the retro-orbital sinus. Following injections, mice were monitored for pain and distress while recovering on a heated pad before being returned to their home cage.

#### RNA isolation and Sanger sequencing (in vivo samples)

Four weeks after injection, brains were dissected in 5 mM HEPES in Hanks’ balanced salt solution and total RNA was isolated from individual brain regions using QIAzol reagent (Qiagen, 79306) and the Qiagen miRNeasy Kit reagent (Qiagen, 217004) according to the manufacturer’s instructions. RNA was reverse-transcribed using the SuperScript III first-strand synthesis system (Invitrogen, 18080051) and primed using oligo dT. Endogenous *Mecp2* cDNA was amplified and analyzed by Sanger sequencing (Supplementary Table 2).

#### RT–qPCR experiments (in vivo samples)

Total RNA was isolated from individual brain regions using QIAzol reagent (Qiagen, 79306) and the Qiagen miRNeasy Kit (Qiagen, 217004) according to the manufacturer’s instructions. DNase digestion was performed on 1 μg of RNA using Turbo DNase Kit (Thermo Fisher, AM1907) according to the manufacturer’s instructions. DNase-treated RNA was then reverse-transcribed using the SuperScript III first-strand synthesis system (Invitrogen, 18080051) and was primed using oligo dT. RT–qPCR experiments were performed on an Applied Biosystems QuantStudio 6 Flex real-time PCR system. All samples were run in triplicate with a standard curve using SYBR select master mix reagent (Thermo Fisher, 4472918) for inward and outward primer sets to quantify CLUSTER guide concentrations across brain regions and circularization, respectively. CLUSTER guide expression was calculated relative to the geometric mean of three housekeeping genes (*Rnu6*, *Rps29* and *Actb)*. Primers sequences are listed in Supplementary Table 2.

#### Immunostaining

Immunostaining was performed as previously described^60^. Briefly, mice were anesthetized using an intraperitoneal injection of 2,2,2-tribromoethanol (Sigma Aldrich, T48402) and killed by transcardial perfusion of PBS pH 7.4, followed by 4% depolymerized paraformaldehyde. Brains were cryoprotected with sucrose, embedded in freezing medium and stored at −80 °C. Sagittal whole-brain sections were cut at 25 μm using a cryostat and stored at −20 °C until staining. Sections underwent heat-mediated antigen retrieval before blocking in PBST (0.1% Triton X-100 in PBS, pH 7.4) and 10% BSA (Sigma, A2153-500G) for 1 h at room temperature. Sections were incubated overnight at 4 °C with rabbit anti-MeCP2 antibody (rabbit monoclonal antibody D4F3, RRID: AB_2143849, Cell Signaling, 1:500) diluted in blocking buffer. Sections were washed with PBST and incubated for 1 h at room temperature with Alexa Fluor 488 donkey anti-rabbit IgG (Invitrogen, A21206 1:750) diluted in blocking buffer. Sections were washed and incubated with 300 nM DAPI (Invitrogen, D1306) in PBS for 5 min. After a final wash in PBS, sections were mounted using Fluoromount G (Invitrogen, 00-4958-02). Images were acquired using ZEN 2.3 SPI FPS Black (version 14.0.0.0) on a Zeiss LSM 710 confocal microscope with a C-Apochromat ×40 (1.2 numerical aperture) objective and LSM T-PMT detector. Images were processed in ImageJ (version 1.54f). Murine MeCP2 protein and nuclei (DAPI) were pseudo-colored in ImageJ to highlight colocation puncta. Results are reported in Fig. 6p and Extended Data Fig. 9.

### Deep amplicon NGS experiment

Retro-orbital injections and RNA isolation were performed as explained above. Two settings were carried out, each with an independent duplicate: (1) nontargeting circular CLUSTER guide RNA and (2) *Mecp2* W104>amber transcript-targeting circular CLUSTER guide RNA. For the latter we selected the two thalamus samples that showed the highest *Mecp2* on-target editing yield during Sanger sequencing. Purified RNA was delivered to the Massively Parallel Sequencing Shared Resource Core (MPSSR) at the Oregon Health and Science University for Illumina library preparation and then transferred to the Molecular Technologies Core (MTC) at the Oregon National Primate Research Center for Illumina amplicon sequencing. The library was prepared with the TruSeq DNA nano library prep kit and sequenced with a MiSeq instrument (1 million reads, 500 cycles, 2 × 500 bp of paired-end reads; Illumina). Furthermore, 2 nM pooled libraries were spiked with 5% PhiX before the sequencing run. The sequencing reads were demultiplexed using BCL Convert (version 2.4.0). Adaptors were trimmed with FastQ (version 1.0.0). Raw FASTQ files were processed using Seqtk (version 1.3-r106). The command ‘seqtk trimfq in.fastq > out.fastq’ was used to trim low-quality bases. The command ‘seqtk seq -q30 -n N in.fastq > out.fastq’ was used to mask base calls with a quality value < 30 as N. The base-call accuracy of the remaining bases was, thus, 99.9%. FASTQ file quality was assessed with FastQC (version 0.11.9)^61^.

#### Mapping of deep amplicon-seq reads

Reads were aligned to the GRCm38/mm10 reference genome using BWA-MEM (version 0.7.17-r1188). The alignments were analyzed using the Integrative Genomics Viewer (version 2.16.2). The editing yields were exported from IGV (version 2.16.2) and then further processed in Excel 2016.

### Next-generation RNA-seq experiment

Retro-orbital injections and RNA isolation were performed as explained above. Two settings were carried out, each with an independent duplicate: (1) nontargeting circular CLUSTER guide RNA and (2) *Mecp2* W104>amber transcript-targeting circular CLUSTER guide RNA. For the latter, we selected the two thalamus samples that showed the highest *Mecp2* transcript on-target editing yield during Sanger sequencing. Purified RNA was delivered to CeGaT for total RNA-seq. The library was prepared from 100 ng of RNA with the KAPA RNA HyperPrep library prep kit with RiboErase (HMR) and KAPA globin depletion hybridization oligos (Roche) and sequenced with a NovaSeq 6000 (50 million reads, 2 × 100 bp of paired-end reads; Illumina). The sequencing reads were demultiplexed using Illumina bcl2fastq (version 2.20). Adaptors were trimmed with Skewer (version 0.2.2)^62^. No quality trimming of the reads was performed. Raw FASTQ file quality was assessed with FastQC (version 0.11.8)^61^. One of the nontargeting CLUSTER guide RNA samples failed quality control (RNA integrity number of 1) and, thus, had to be removed from the analysis. Results are reported in Fig. 6o and Extended Data Fig. 8. In addition, we reanalyzed brainstem samples from a similar previous study that applied the boxB/λN-ADAR system^44^. From this existing dataset, we randomly selected samples from the untreated Rett mouse negative control group and the two samples from the boxB/λN-ADAR targeting virus-treated group that that showed the highest *Mecp2* transcript on-target editing yield. Results are reported in Extended Data Fig. 8.

#### Mapping of RNA-seq reads

Reads were uniquely aligned to the GRCm38/mm10 reference genome using STAR (version 2.7.3a) (‘STAR-2.7.3a --alignSJoverhangMin 8 --alignIntronMax 1000000 --alignMatesGapMax 600000 --outFilterMismatchNoverLmax 0.3 --outFilterMismatchNoverReadLmax 1 --outFilterMatchNminOverLread 0.66 --outFilterMultimapNmax 1 --outReadsUnmapped Fastx --outSAMattributes All --outSAMtype BAM Unsorted --quantMode GeneCounts --genomeLoad LoadAndKeep --limitBAMsortRAM 37580963840 --outBAMsortingThreadN 10 --runThreadN 40 --genomeDir mm10.STAR.7.ReadsLn100.gencodeM18 --readFilesCommand cat --readFilesIn file-name_R1.fastq file-name_R2.fastq --outFileNamePrefix file-name’)^63^.

#### RNA-editing index

The RNA-editing index tool^46^ calculates the average editing level across all A bases in a set of regions. The editing index is defined as the ratio of the number of A•G mismatches to the total coverage of A bases. Genomic sites overlapping common single-nucleotide polymorphisms (dbSNP142) were excluded. The RNA-editing index tool was used to assess the overall editing for all the coding sequence regions of the mouse genome.

#### Global RNA editing

To assess global RNA editing, we used the known editing sites from the REDIPortal^55^ and from a very small set of evolutionarily conserved A-to-I editing sites^64^. We calculated the editing levels in those sites using REDITools^65^ with a coverage *≥* 50 reads for every site. For every group of each dataset (this study and the previous study^44^), we combined the editing levels of the replicates for each site. We then selected the 2,532 sites that were common in both datasets.

### Data analysis

Data were analyzed using Excel 2016 and GraphPad Prism 8. Figures were created with CorelDraw 2017. The manuscript was written using Word 2016. The custom GuideRNA-Forge tool was written in Python (version 3.9). Guide RNA folds were created using the ViennaRNA Package (version 2.0, http://rna.tbi.univie.ac.at//cgi-bin/RNAWebSuite/RNAfold.cgi). qPCR analysis was performed using the Applied Biosystems 7500 data analysis software version 1.1 (animal experiment) and version 2.3 (remainder of the study).

### Reporting summary

Further information on research design is available in the Nature Portfolio Reporting Summary linked to this article.

## Online content

Any methods, additional references, Nature Portfolio reporting summaries, source data, extended data, supplementary information, acknowledgements, peer review information; details of author contributions and competing interests; and statements of data and code availability are available at 10.1038/s41587-024-02313-0.

## Supplementary information

Supplementary Information Supplementary Figs. 1–14, Tables 1–4 and Notes 1–5.

Reporting Summary

Supplementary Table 1 A-to-G RNA-editing index and global off-target analysis based on NGS data.

## Data Availability

Transcriptome-wide RNA-seq data are accessible from the National Center for Biotechnology Information (NCBI) Gene Expression Omnibus database with accession code GSE265898. Deep amplicon NGS data are accessible from the NCBI Sequence Read Archive SRA database with accession code PRJNA1100948. Transcriptome-wide RNA-seq data from Sinnamon et al. are accessible from the NCBI SRA database with accession code PRJNA849938. Structural data from Thuy-Boun et al. and Stefl et al. are available from PDB 5HP2 and 2L2J, respectively.

## Code availability

The full code of the GuideRNA-Forge tool is available upon request. The compiled tool can be obtained from GitHub (https://github.com/recruitment-cluster-finder/GuideRNA-FORGE)^66^.
