# Supplementary material for: Precise in vivo RNA base editing with a wobble-enhanced circular CLUSTER guide RNA
Source: Nat Biotechnol. 2024 Jul 12;43(4):545–57. doi: 10.1038/s41587-024-02313-0 (PMC11994451; doi:10.1038/s41587-024-02313-0)
Supplement: Supplementary file 1 — Supplementary Figs. 1–14, Tables 1–4 and Notes 1–5. [file 41587_2024_2313_MOESM1_ESM.pdf]

nature biotechnology

Supplementary information https://doi.org/10.1038/s41587-024-02313-0

Precise in vivo RNA base editing with a wobble-enhanced circular CLUSTER guide RNA

In the format provided by the authors and unedited

Supplementary Information

Inventory of Supplementary Information

- Supplementary Figures S1-S14
- Supplementary Tables S1-S4
- Supplementary Notes S1-S5

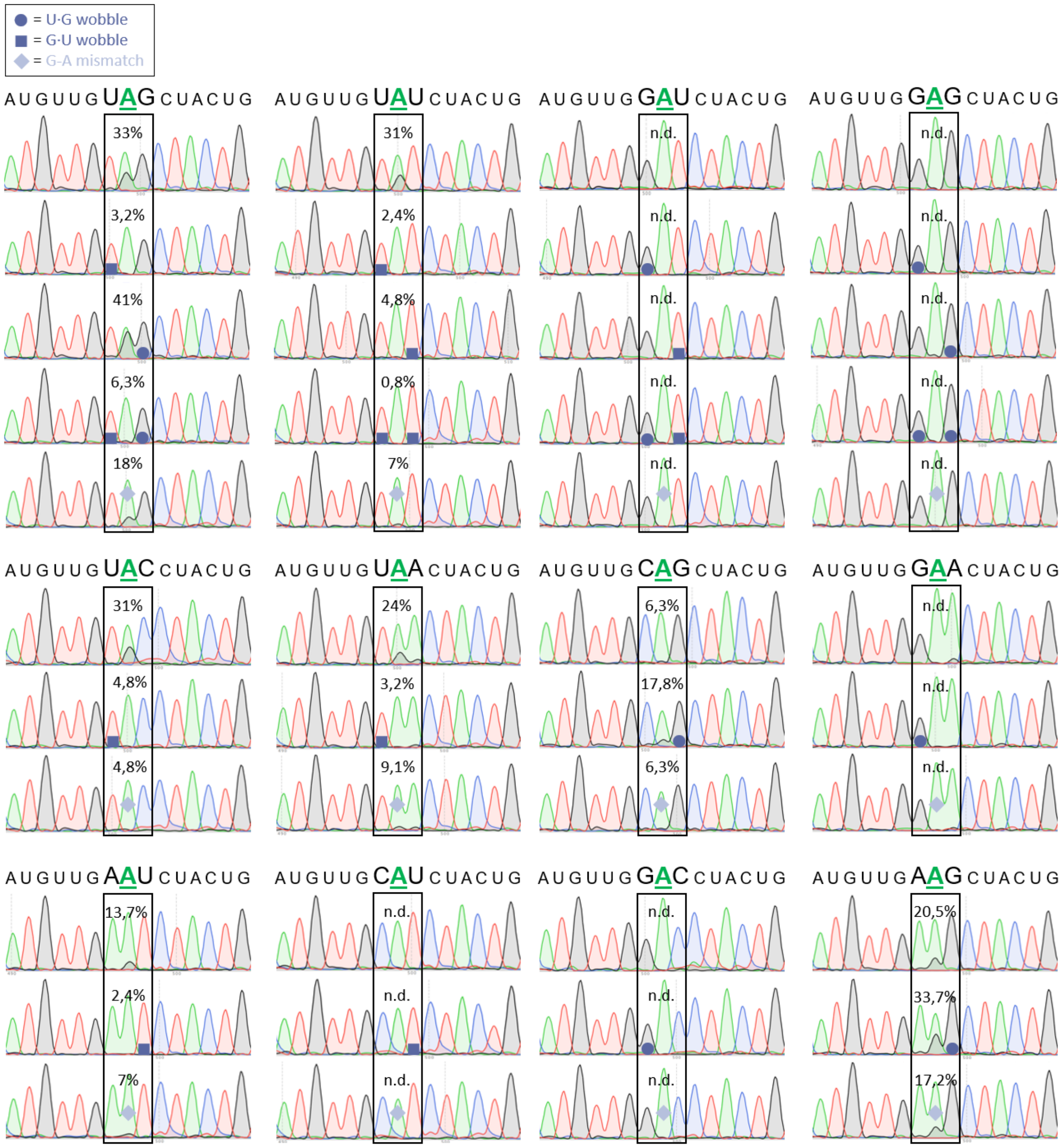

Supplementary Figure S1. Exemplary sequencing reads related to the dataset from main text Figure 1. *Editing of cis-acting reporters in ADAR1 p110 Flp-In T-REx cells, showing the effect of wobble base pairs on target triplets containing Uracil and/or Guanine. The target adenosine is highlighted in green bold and underlined. The sort and position of mismatch/wobble is indicated in the sequencing trace and explained in the legend.*

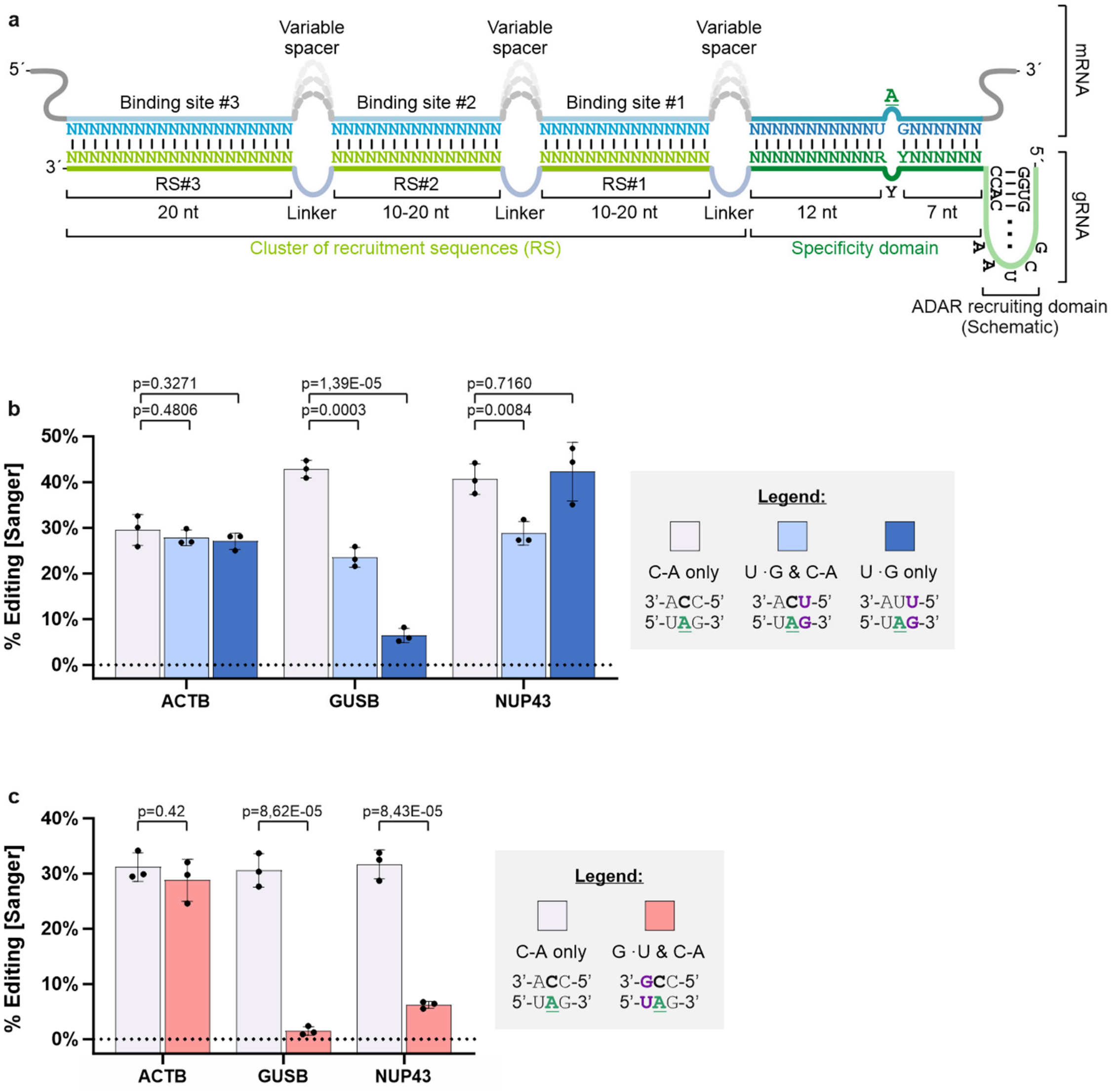

Supplementary Figure S2. Enhancing U·G- and suppressing G·U wobble base pairs in the context of a C-A mismatched target adenosine. a) *Design of a linear CLUSTER gRNA consisting of three recruitment sequences (RS) connected by triple adenosine linkers, a 20 nt long specificity domain and an ADAR recruitment motif based on the natural R/G motif of the glutamate receptor 2. The guide RNA is displayed bound to its target transcript. The target adenosine is highlighted in green, bold and underlined. The counter base of the target adenosine is a Y (Y = C or U). The counter base of the Guanine at the 3' nearest neighbor position is also a Y, the counterbase at the 5' nearest neighbor position is a R (R = A or G).* ***b)*** *Editing yields achieved using linear CLUSTER guide RNAs targeting the endogenous housekeeping genes ACTB, GUSB and NUP43. The designs differ only in the counter bases present at the target adenosine and its 3' nearest neighbor Guanine.* ***c)*** *Similar to b), but the designs differ in the counter base present at the 5' nearest neighbor Uracil. Editing in b) and c) was performed in HEK293FT cells using plasmid-borne guide RNAs and endogenous ADAR1. Data in b) and c) are shown as the mean editing percentage ± s.d. of N = 3 biological replicates. For statistical analysis, a student t-test (two-tailed, parametric) was applied.*

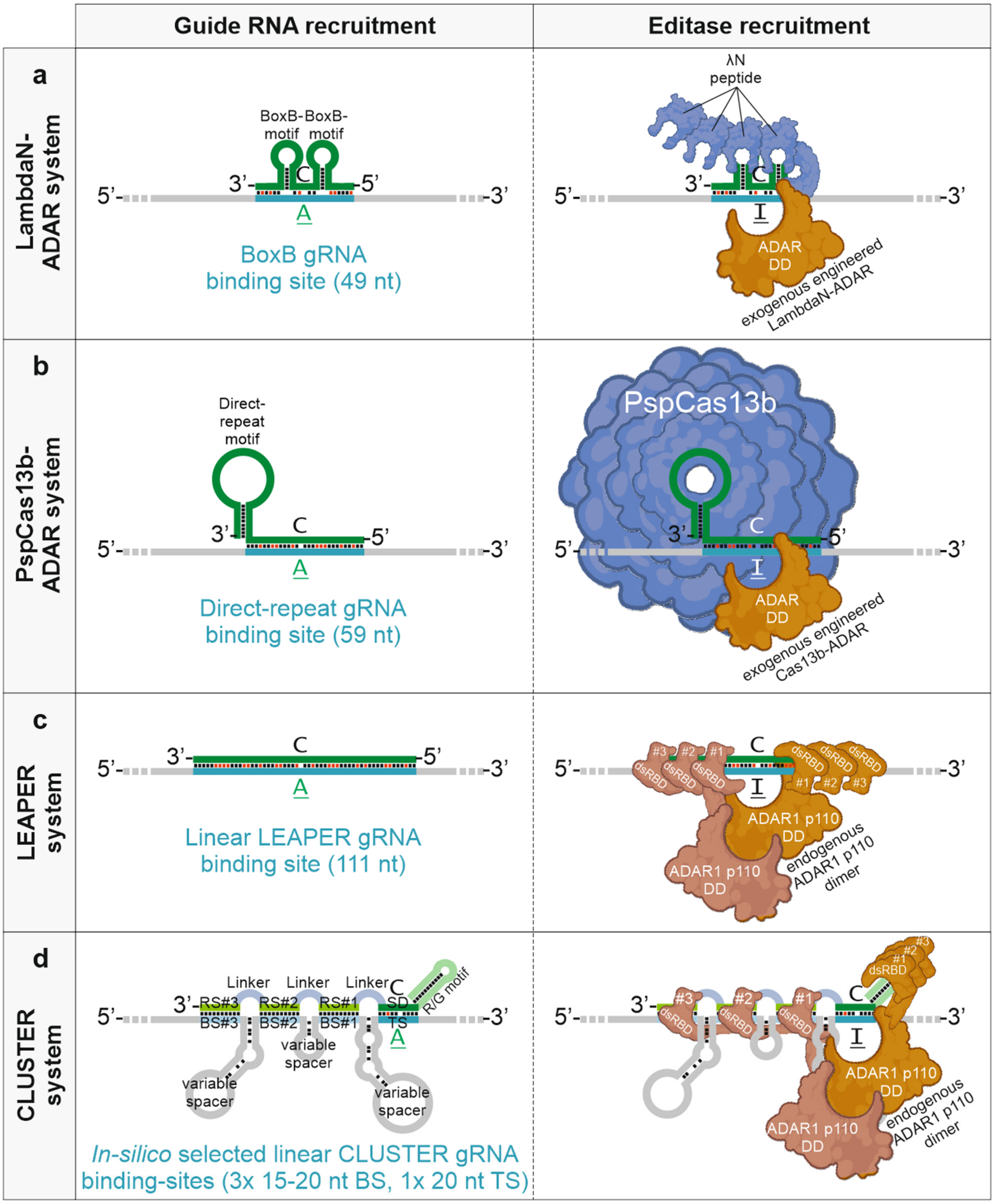

Supplementary Figure S3. Overview of the targeting strategies of the encodable side-directed RNA editing systems that are applied in this study. *In all panels the target mRNA is displayed in grey and the guide RNA binding site(s) on it in different shades of blue. Guide RNA elements are displayed in different shades of green. The target adenosine within the target mRNA is displayed bold, underlined, and in green, while its Cytosine counterbase in the guide RNA is displayed in bold and black. Hydrogen bonds are displayed as black or red lines.*

***Supplementary Figure S3 continued. Overview of the targeting strategies of the encodable side-directed RNA editing systems that are applied in this study.** The abundance of red lines (not their specific positions) illustrates the likelihood of bystander off-target events for the respective system and antisense element. Non-human protein domains/peptides are displayed in dark blue. Human protein domains are displayed in orange. To distinguish two independent human proteins within a homodimer the domains of the first protein are displayed in orange and the domains of the second one in red. **(a)** The targeting strategy of the λN-ADAR system utilizes phage-derived elements. The nanomolar affinity of the λN peptide for boxB RNA-hairpin motifs enables efficient recruitment of the editase fusion protein. The latter consists of an ADAR deaminase domain (e.g. hADAR1d, hADAR2d, or hyperactive mutants) and four λN peptides, while the guide RNA consists of a 49 nt antisense part that is interrupted by two boxB motifs. **(b)** The targeting strategy of the λN-ADAR system utilizes bacteria-derived elements and is a variation of the λN-ADAR system. The affinity of the PspCas13b protein for direct-repeat motifs enables recruitment of the editase fusion protein. The latter consists again of an ADAR deaminase domain (e.g. hADAR1d, hADAR2d, or hyperactive mutants) and the PspCas13b protein, while the guide RNA consists of a 59 nt antisense part and a 3'-terminal direct repeat motif. **(c)** The targeting strategy of the LEAPER system utilizes the double-stranded RNA binding domains of endogenous human ADARs. In contrast to the previous two systems the overexpression of an engineered editase is not required. Instead, the 111 nt long LEAPER guide RNA forms a dsRNA substrate with the target mRNA allowing for recruitment of endogenous ADAR enzymes. Due to the length of the duplex this could potentially allow for ADAR homo- and/or heterodimer formation. Exemplary an ADAR1 p110 homodimers is displayed. The unstructured duplex makes the design prone to off-target editing events, if no further off-target solutions like e.g. GA-mismatches are applied. There is also a version of this system that utilizes the Tornado expression system to achieve circularization of the guide RNA. The latter increases its stability and thus guide RNA abundance and consequently improves the editing yields. **(d)** The targeting strategy of the CLUSTER system does also utilize the double-stranded RNA binding domains of endogenous human ADARs. Thus, the overexpression of an engineered editase is again not required. The guide RNA design is however not unstructured but instead highly sophisticated. It consists of several functional elements. The R/G motif is a dsRNA-hairpin based on the natural R/G site of the GRIA2 transcript, that has evolved for efficient ADAR2 binding and was engineered for human ADAR1 p110 recruitment via rational design. It might potentially enable ADAR recruitment even before the antisense parts of the CLUSTER guide RNA have bound their target transcript. In addition, it increases the guide RNA's binding surface for ADAR enzymes without extending its antisense part. The length of the latter is an important factor for the abundance of bystander off-target events. The specificity domain (SD) of the guide RNA binds to the target sequence (TS), which represents the sequence on the target transcript that contains the target adenosine. The SD is the only part of the CLUSTER guide RNA's antisense part that is not fully modular, as its sequence depends on the sequence context around the target adenosine in the TS. In contrast the antisense parts of all other site-directed RNA editing systems are completely defined by their target sequence and do not contain any modular elements. The length of the SD, although flexible, is in most instances set to 20 nt, according to the footprint of the ADAR deaminase domain. A shorter sequence might negatively affect editing, while a longer sequence increases the chance of bystander off-target events caused by this element of the guide RNA. The Cluster of recruitment sequences (RS) is the last and most modular element of the CLUSTER guide RNA design. It consists of between 3 and 9 RS each being 15 to 20 nt long.*

Supplementary Figure S3 continued. Overview of the targeting strategies of the encodable side-directed RNA editing systems that are applied in this study. *Their corresponding binding sites (BS) on the target transcript can be located thousands of nucleotides distal from each other, although closer proximity is correlated with higher editing yields. The optimal RS number and length was experimentally determined. However, the rational for the RS number was to provide at least one RS per ADAR dsRBD. The rational for the RS length was their ability to bind their target transcript independent from the binding power of the remaining RS's at 37°C and at a reasonable GC-content. Each RS binding site is identified by an algorithm that searches for uninterrupted >15 nt sequences that contain adenosines only in the very hard to edit 5'-GAB (B = G, C; U) triplet context or that contain no adenosines at all. This selection process makes sure that bystander off-target events within the binding sites of the cluster of recruitment sequences are virtually impossible. The individual RS are connected to each other via adenosine linkers of 1-5 nt length. The corresponding spacer within the target transcript is variable and can range from zero to thousands of nucleotides, depending on the placement of each RS. The RS selection process depends on the secondary structure within the antisense part of all potential guide RNAs for a certain target transcript. All potential RS that are identified by the algorithm are recombined by our gRNA-Forge tool, folded using the ViennaRNA package, and scored for low secondary structure within the antisense part. The scoring is performed via the DBR (dot-bracket ratio) metric, which gives a higher value the more bases are unpaired in the MFE (mean free energy) structure of the guide RNA. A high DBR score thus means that most of the guide RNAs antisense part is readily available for target transcript binding, instead of engaging in unproductive folding in cis. CLUSTER guide RNAs can be circularized using the Tornado expression system. Circularization increases their stability and thus the guide RNA abundance. Consequently, this improves the editing yields. The engineering of circular CLUSTER guide RNAs is described in further detail in Extended Data Figure 4, S9, and S10. This figure contains elements from BioRender.com.*

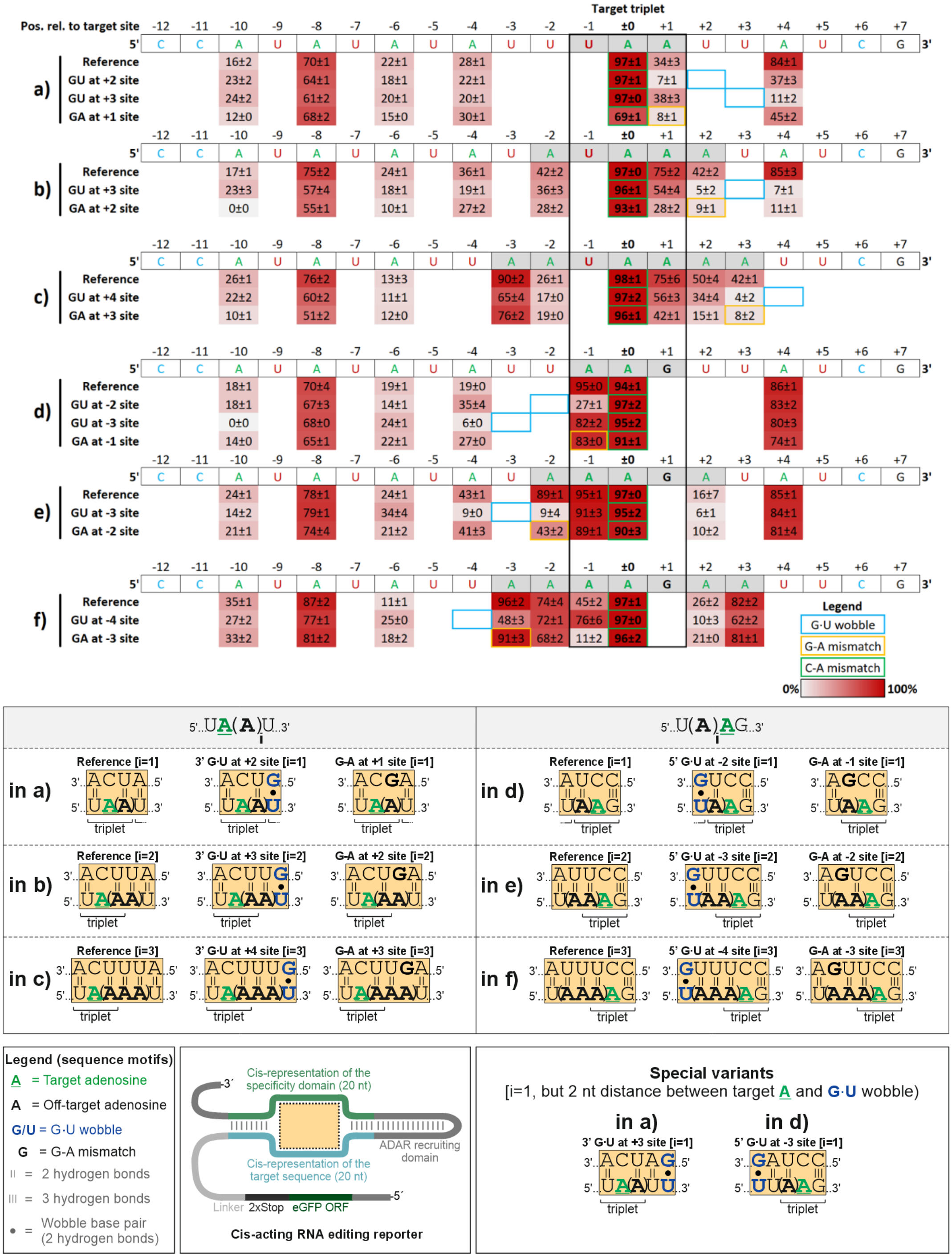

Supplementary Figure S4. Characterizing the up- and downstream distance of editing suppression after protecting the first (-1, +1), second (-2, +2) or third (-3, +3) nearest neighbor sites by G·U wobble. *In panel* ***a)*** *to* ***c)****, we studied a series of three 5´-[U*__*A*__*(*__*A*__*)*$_i$*]U sequence motifs (i= 1-3) with increasing distance between the 3´-G·U wobble and the on-target* __*A*__ *in cis-acting constructs. In panel* ***d)*** *to* ***f)****, we assessed a series of three 5´-U[(*__*A*__*)*$_i$__*A*__*G] sequence motifs (i = 1-3) to test the effect of a 5´-G·U wobble into the 3´ direction. In all panels, we also benchmarked the effect of the G-A mismatch.*

Supplementary Figure S4 continued. Characterizing the up- and downstream distance of editing suppression after protecting the first (-1, +1), second (-2, +2) or third (-3, +3) nearest neighbor sites by G·U wobble. *Editing efficiencies on-target (±0) and at bystander sites (±1, ±2, ±3) are displayed in a heatmap fashion. The exact positioning of the respective wobble or G-A mismatch is indicated by blue and yellow frames, respectively. The design of the cis-acting RNA editing reporter and a detailed description of the evaluated sequence motifs are given below the datasets. The G·U wobble base pairs (5´ and 3´) enable a more precise control of bystander editing by giving stronger and more focused suppression compared to the G-A mismatches. Thus, the wobble strategy is particularly powerful to suppress bystander editing close to an on-target adenosine in A-rich codons. Data in a) – f) are shown as the mean ± s.d. of N = 3 biological replicates for each position in the heatmap.*

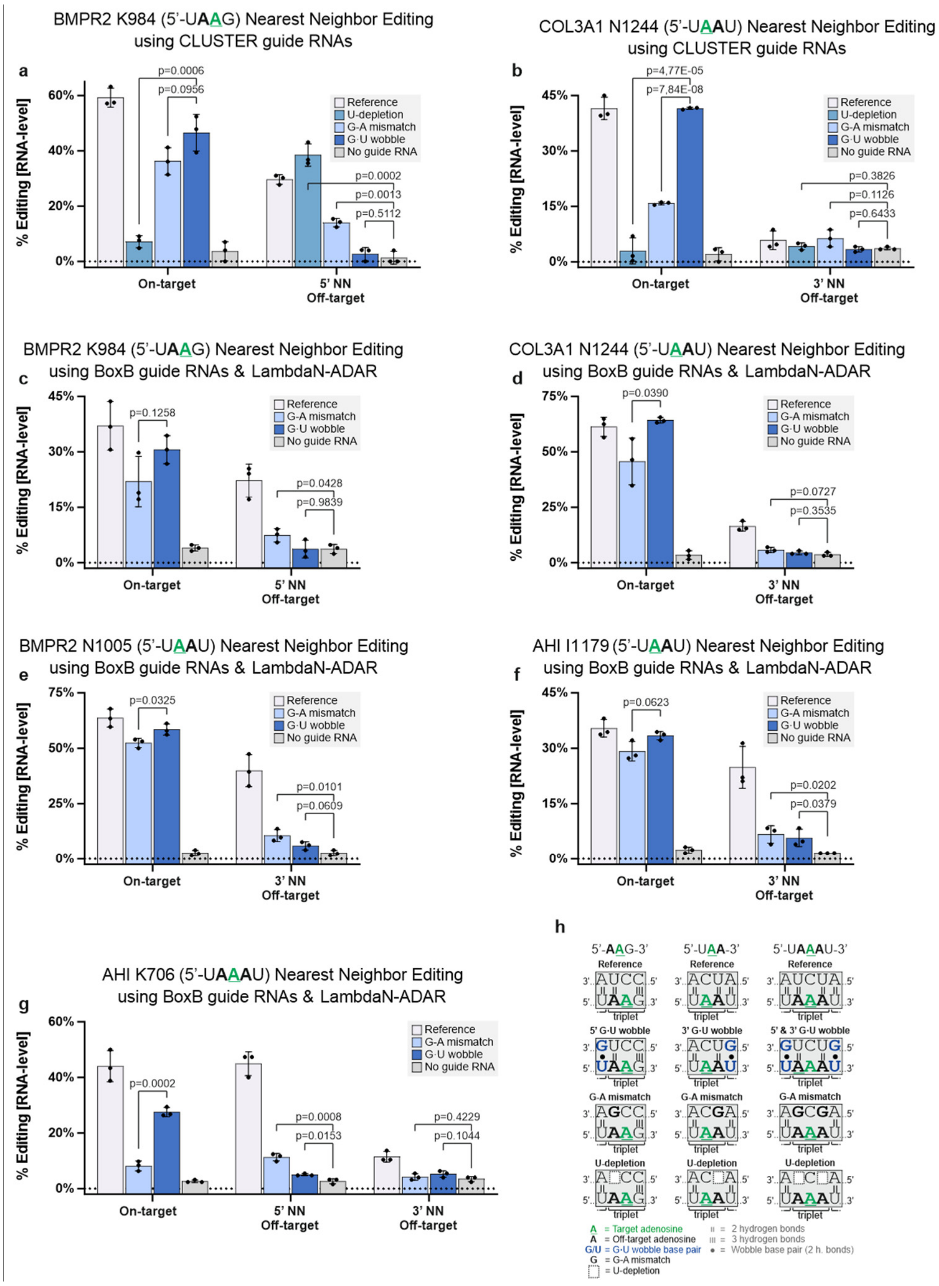

Supplementary Figure S5. Individual datasets used for the meta-analysis shown in Extended Data Figure 3e. *Suppression of bystander editing at nearest neighbor adenosines via G·U wobbles, G-A mismatches, or uridine-depletion using either trans-acting CLUSTER guide RNAs in* ***a)*** *and* ***b)*** *or the λN-ADAR approach in* ***c) – g)****. The target transcript, -amino-acid and sequence context are indicated at each bar chart. The target adenosine is green, underlined and bold. The off-target adenosine(s) is/are black and bold.* ***h)*** *Detailed notation of the different base-pairing motifs to target specific sequence motifs. Editing was performed in HeLa cells using plasmid-borne editase (λN-ADAR) and/or guide RNA (2x boxB, CLUSTER). Data in a) - g) are shown as the mean editing percentage ± s.d. of N = 3 biological replicates. For statistical analysis, a student t-test (two-tailed, parametric) was applied.*

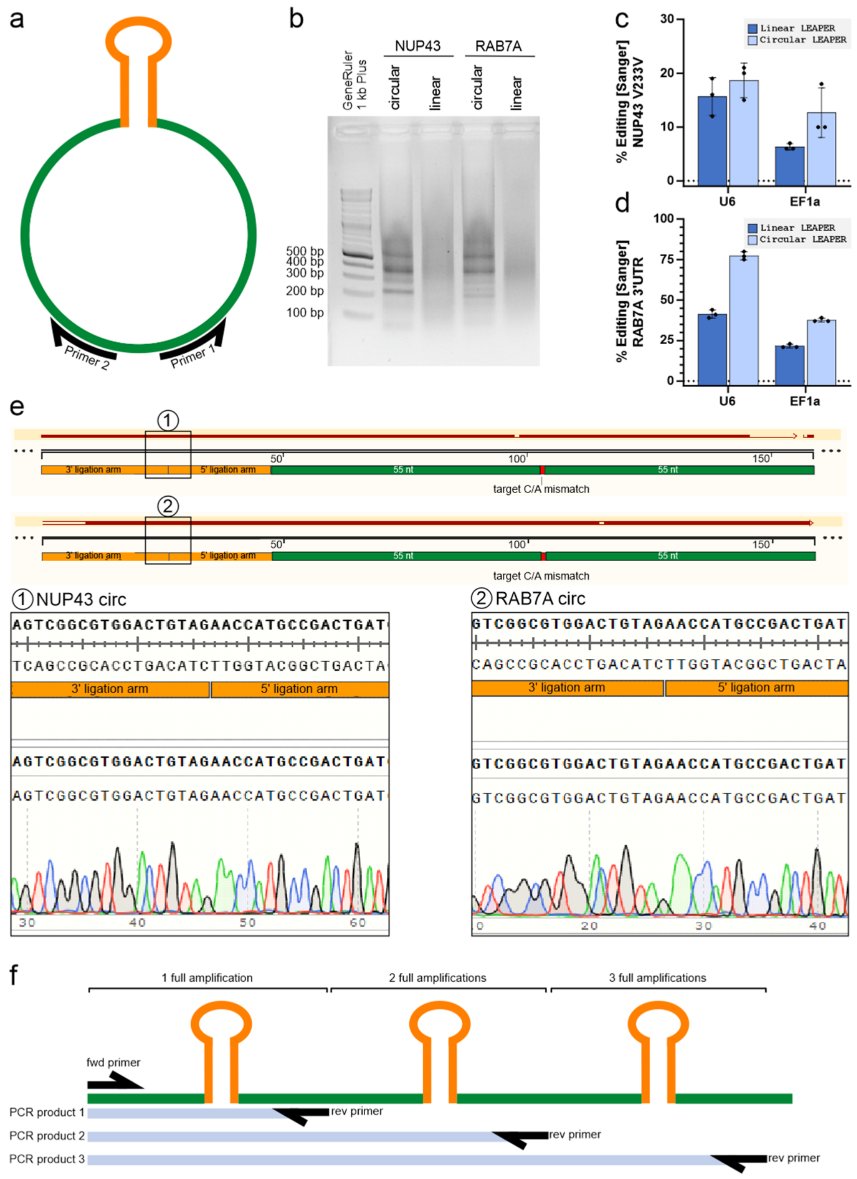

Supplementary Figure S6. Initial characterization of circular LEAPER guide RNAs. a) *Schematic view of primer binding sites for verification of guide RNA circularization* ***b)*** *Circularization of 111 nt antisense guide RNAs targeting NUP43 and RAB7A was verified by RT-PCR and subsequent agarose gel electrophoresis. Primers were facing outwards from the central cytosine base opposite the target adenosine. Clear bands were only obtained for the circular guide RNAs but not their linear analogs. Multiple bands are visible due to rolling circle amplification during reverse transcription.* ***c)-d)*** *Circularization of guide RNAs improved NUP43 (panel c) and RAB7A (panel d) editing compared to their linear analogs up to 2-fold and was achieved both with pol-II (EF1α) and pol-III (U6) promoters.* ***e)*** *Precise circularization between 3' and 5' ligation arm was verified by Sanger sequencing of PCR products from panel b) for both guide RNAs (NUP43 and RAB7A). Editing was performed with plasmid-borne guide RNAs and endogenous NUP43 or RAB7A in HEK293FT cells (endogenous ADAR). Data in c) and d) are shown as the mean editing percentage ± s.d. of N = 3 biological replicates.*

Endogenous RAB7A 3' UTR

Triplet context	Adenosine pos. from 5'-end (relative to target site)	U6 Linear LEAPER	U6 Circular LEAPER	Ef1α Linear LEAPER	Ef1α Circular LEAPER
GAG	1595 (-55)	2±1	1±0	2±0	1±0
GAA	1602 (-48)	2±1	6±1	1±0	1±0
AAC	1603 (-47)	3±1	25±2	1±0	7±0
GAU	1614 (-36)	1±0	1±0	1±0	1±0
CAG	1619 (-31)	4±1	15±0	2±0	7±1
GAG	1621 (-29)	1±0	2±0	1±0	1±0
UAC	1627 (-23)	26±1	45±2	12±0	17±0
CAG	1631 (-19)	6±1	16±1	2±1	4±0
GAA	1633 (-17)	1±0	4±1	1±0	1±0
AAU	1634 (-16)	7±0	19±2	4±2	5±1
GAA	1640 (-10)	2±0	5±0	1±0	1±0
AAA	1641 (-9)	10±2	34±3	5±1	10±1
AAU	1642 (-8)	6±0	26±1	2±0	8±1
CAG	1646 (-4)	1±0	3±0	2±0	2±0
**UAG**	**1650 (±0)**	**54±0**	**68±3**	**23±2**	**33±1**
CAG	1656 (+6)	2±0	7±1	2±0	3±0
UAU	1659 (+9)	21±1	43±2	11±2	14±1
UAC	1666 (+16)	3±1	42±2	2±0	15±1
CAG	1668 (+18)	1±0	2±0	2±0	2±0
UAG	1671 (+21)	2±0	45±3	2±0	13±1
GAC	1673 (+23)	1±1	0±0	2±0	0±0
CAC	1675 (+25)	1±0	3±1	2±0	2±0
CAA	1677 (+27)	3±1	13±1	3±1	4±0
AAG	1678 (+28)	4±0	25±3	3±1	6±0
GAA	1680 (+30)	1±0	0±0	1±0	0±0
AAU	1681 (+31)	2±1	4±0	2±1	1±0
UAU	1684 (+34)	9±1	31±2	8±0	4±1
UAC	1688 (+38)	3±0	4±2	4±0	4±0
UAU	1697 (+47)	1±0	26±1	1±0	2±0
CAA	1700 (+50)	1±1	1±1	1±1	1±0
AAA	1701 (+51)	2±0	2±1	3±1	3±1
AAG	1702 (+52)	1±0	2±0	1±0	1±0
GAC	1704 (+54)	1±0	0±0	0±0	1±0

Legend:

Av±Sd Target adenosine row

Av±Sd Off-target adenosine row

Zero Max. Editing yield for each target mRNA

U<u>A</u>G = Target triplet

NAN = Off-target triplet

Supplementary Figure S7. Circularization of LEAPER guide RNAs increases both on-target and bystander editing. *Editing heat-maps of LEAPER guide RNA binding sites within RAB7A. LEAPER guide RNAs are an unstructured, 111 nt long antisense sequence to the target mRNA with a central C-A mismatch. The triplet context for each listed bystander editing event is given with the edited adenosine highlighted in bold blue. The position of each site is given relative to the target adenosine (±0 position, in bold green and underlined). Editing was performed with plasmid-borne guide RNAs and endogenous RAB7A in HEK293FT cells (endogenous ADAR) after guide RNA expression from U6 (pol-III) or EF1α (pol-II) promoter. Data are shown as the mean editing percentage ± s.d. of N = 3 biological replicates.*

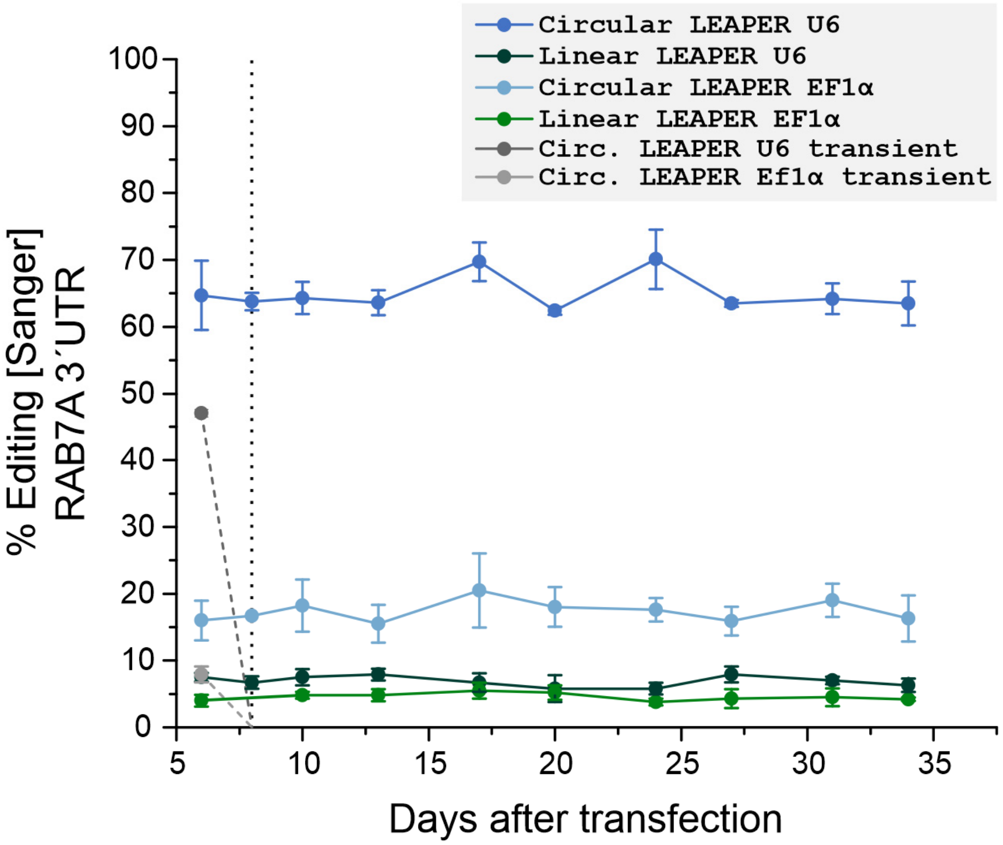

Supplementary Figure S8. Circularization strongly increases editing yields of stably integrated guide RNAs. *Circular or linear 111 nt LEAPER guide RNAs targeting the 3'-UTR of RAB7A were stably integrated into the genome of HeLa cells via the PiggyBac transposon system and puromycin selection for 8 days (spotted vertical line). Stable integration of circular guide RNAs increased the editing yield from both promoters, U6 and EF1α, by 8-fold and 3-fold, respectively. Editing yields stayed constant over ten passages (= 28 days) for the stably integrated circular guide RNA under both promoters. In contrast, editing yields for the linear embodiment and the transiently transfected circular guide RNAs was at the Sanger detection limit after selection. Data are shown as the mean editing percentage ± s.d. of N = 3 biological replicates per data point.*

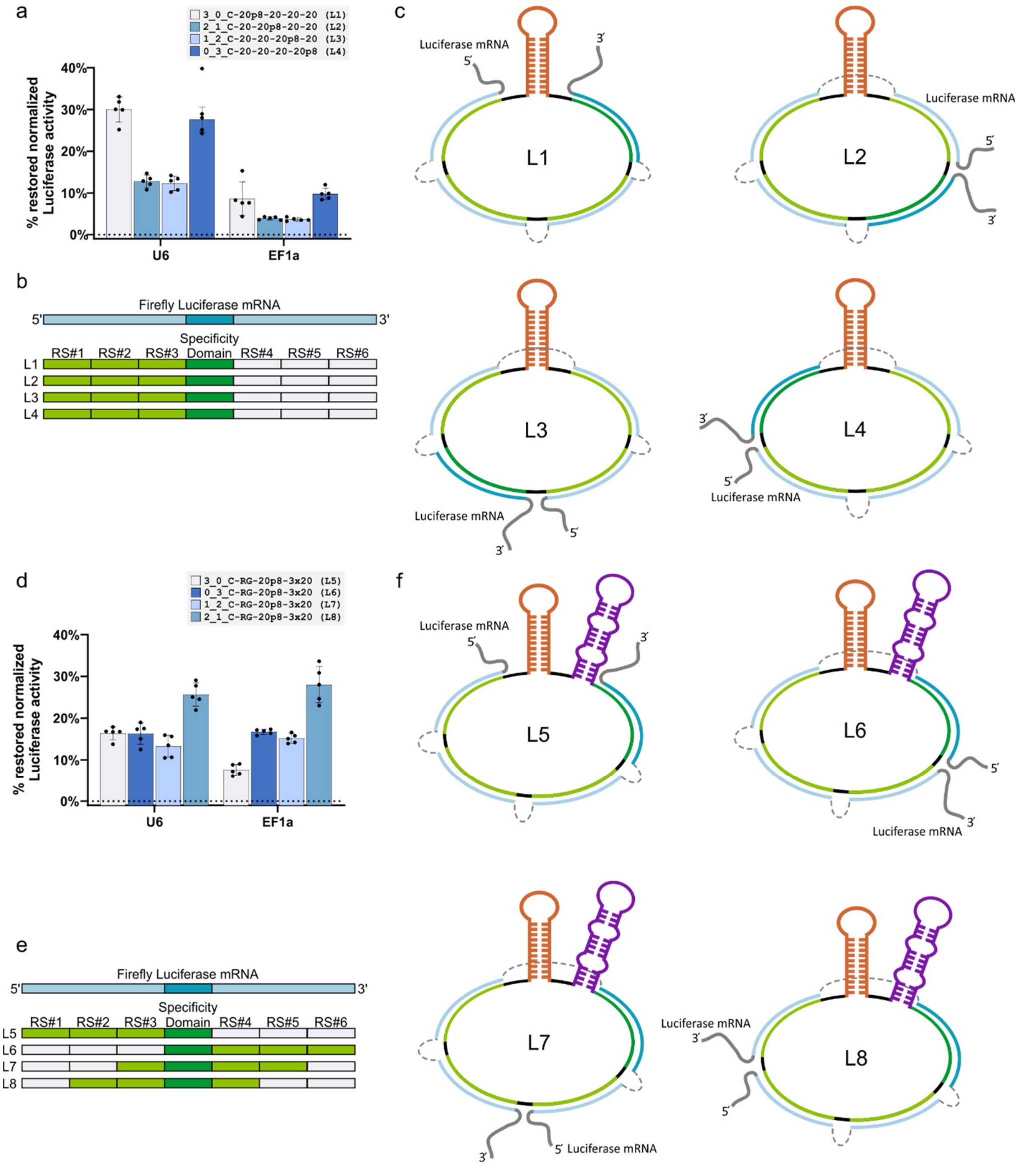

Supplementary Figure S9. First round engineering circular CLUSTER guide RNAs. a) *Percentage of restored firefly luciferase activity normalized to renilla luciferase activity for circular guide RNAs expressed via U6 or EF1α promoter in HeLa cells. Placement of the specificity domain (see panel* ***b*** *and* ***c****) adjacent to the ligation stem of the Tornado expression system improves restoration of normalized Luciferase activity (L1 and L4).* ***d)*** *Introduction of an R/G-motif (see panel* ***e*** *and* ***f****) improves editing for EF1α expressed guide RNAs and can compensate for potential lower expression level compared to U6 expression. Placement of the mRNA exits approximately opposite of the specificity domain resulted in the highest restoration (L8). Panel* ***b*** *and* ***e*** *give a schematic view of recruitment sequences (light green) and their binding sites (light blue) relative to the target site (dark blue) on the mRNA. Panel* ***c*** *and* ***f*** *give 2-dimensional representations of guide RNAs with green = specificity domain, light green = recruitment sequence, dark blue = specificity domain binding site, light blue = recruitment sequence binding site, orange = ligation stem, dark purple = R/G-motif. All Dual Luciferase Assays were performed with N = 5 replicates, shown is the mean ± s.d.*

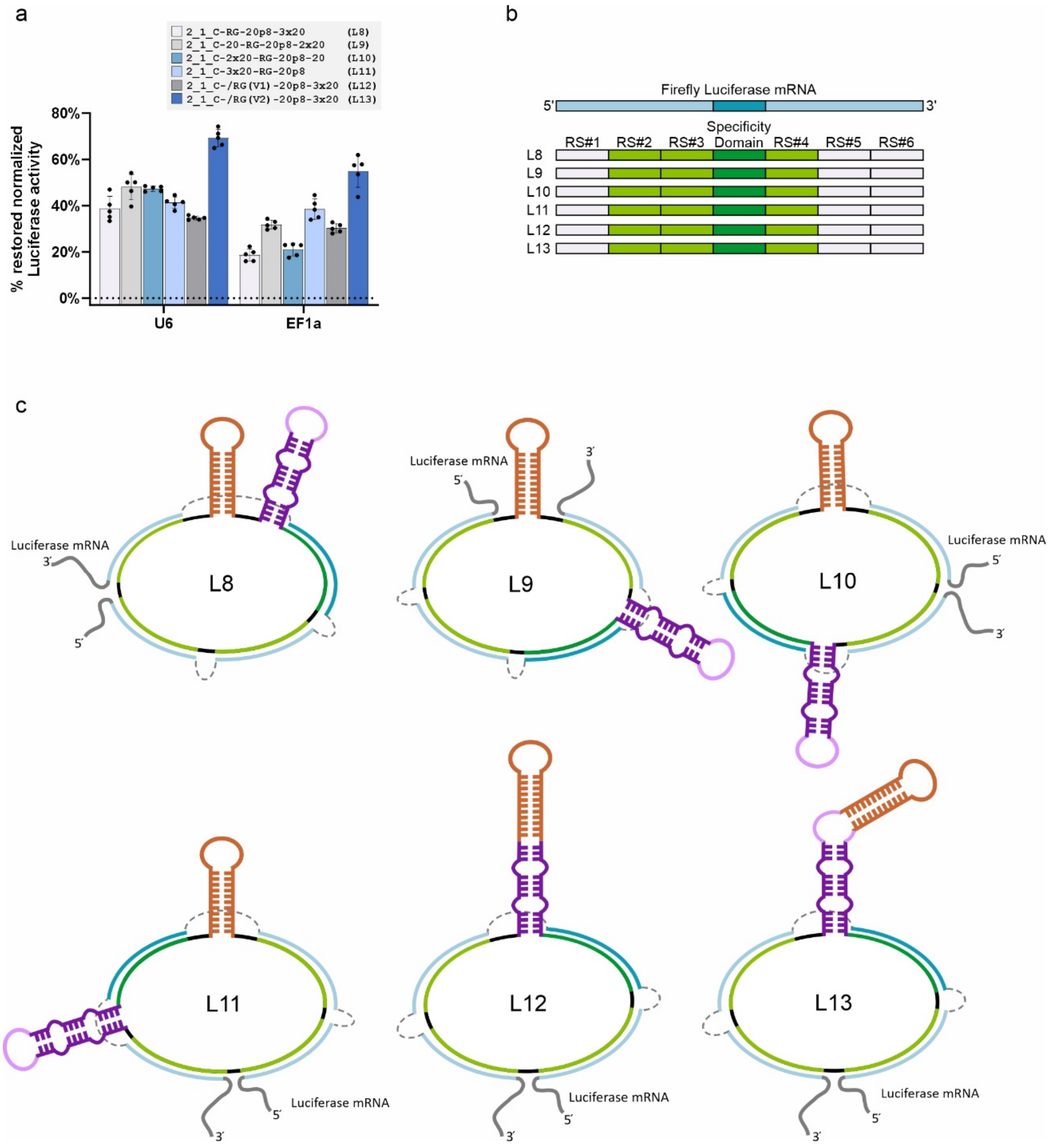

Supplementary Figure S10. Second round engineering circular CLUSTER guide RNAs. a) *Percentage of restored Firefly luciferase activity normalized to Renilla luciferase activity for circular gRNAs expressed via U6 or EF1α promoter in HeLa cells. Position of the R/G-motif and the specificity domain in relation to the ligation stem had only minor influence (L5, L9-L12). In contrast, a flexible split-R/G-motif adjacent to the ligation stem (construct L13) boosted luciferase restoration.* ***b)*** *Schematic view of recruitment sequences and their position relative to the target site for the guide RNAs shown in panel a) on the mRNA.* ***c)*** *2-Dimensional representation of guide RNAs from panel a) bound to the target transcript; green = specificity domain, light green = recruitment sequence, dark blue = specificity domain binding site, light blue = recruitment sequence binding site, orange = ligation stem, dark purple = R/G-motif (ADAR recruitment motif), light purple = penta-loop (5 nt bulge). All dual luciferase assays were*

performed with N = 5 replicates, shown is the mean ± s.d.

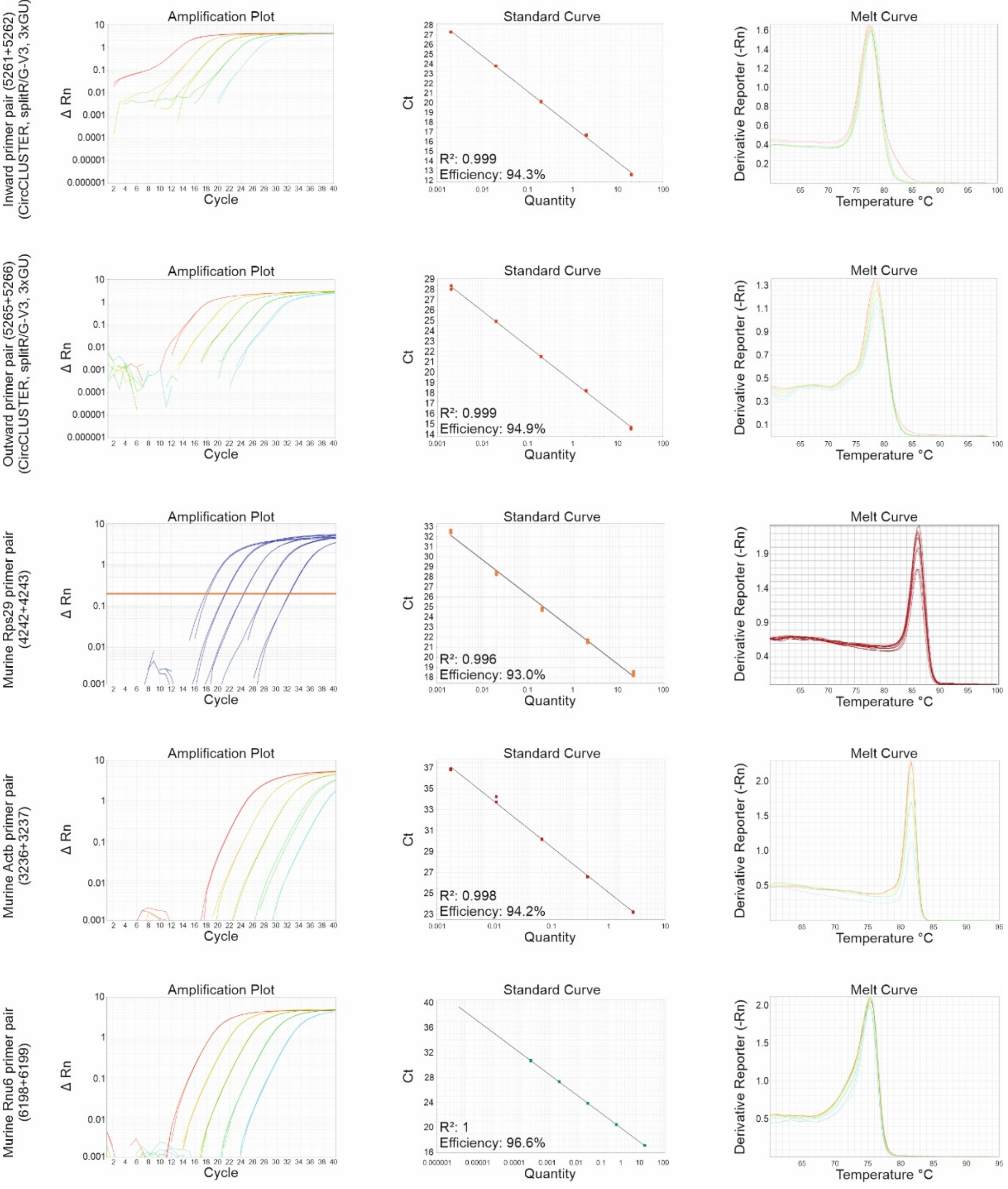

Supplementary Figure S11: Characterization of RT-qPCR primer pairs. *All RT-qPCR primer pairs used (see Supplementary Table S2 & S3) were characterized here or previously [1] for their efficiency and were in the acceptable range between 80% and 110%, with good standard curve fits represented by R2-values >0.99. The dilution series (20 ng, 2 ng, 0.2 ng, 0.02 ng, 0.002 ng) resulted in a linear dynamic range up to 0.002 ng cDNA template in the amplification plots, and clean single-transition melting curves. The cDNA template used for the guide RNA primer pair characterization came from HeLa cell total RNA. 0.8x10^5 HeLa cells were transfected with 1000 ng circular CLUSTER gRNA plasmid (pTS2108) and 200 ng mMecp2 W104Amber encoding plasmid per well using a plasmid to Lipofectamine-3000 ratio of 1:1.5. The template for the characterization of the three housekeeping genes murine Actb, Rps29 and Rnu6 came from primary murine brain tissue (cerebellum) of a targeting virus treated animal.*

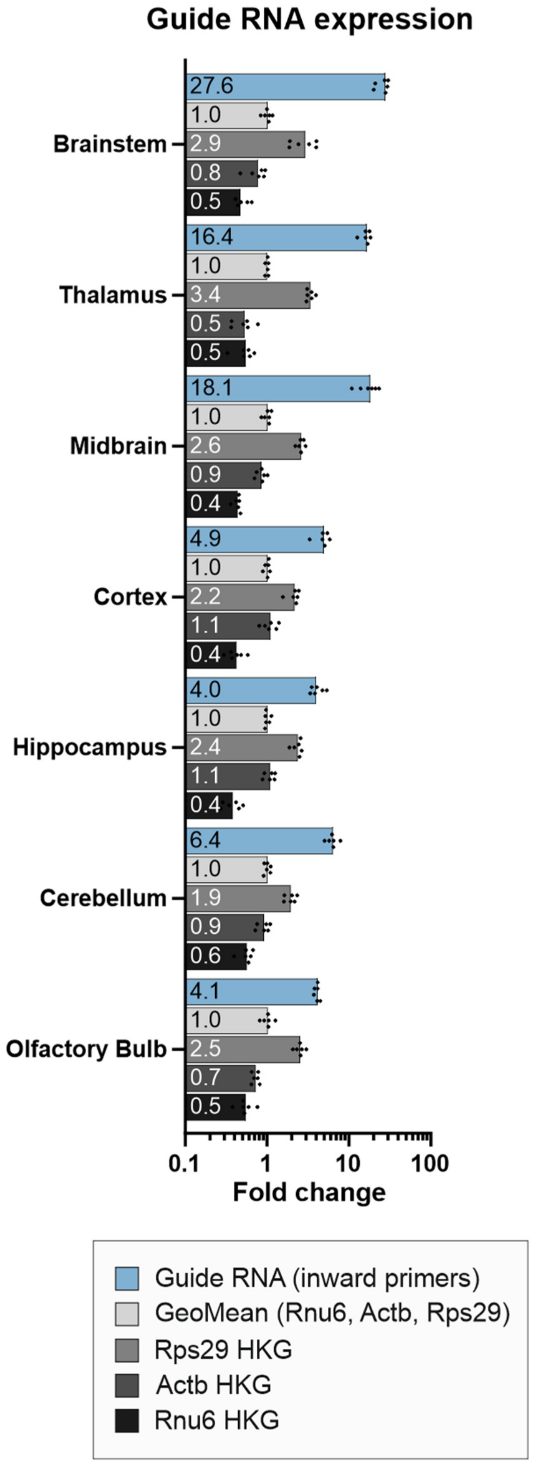

Supplementary Figure S12. Extended in-vivo guide RNA RT-qPCR dataset. *In-vivo guide RNA expression in all analysed brain tissues including all used housekeeping genes. Data from N = 2 targeting guide RNA treated Rett syndrome animals are shown as the median of three technical replicates each. All fold changes are normalized to the geometric mean of the three murine housekeeping genes Rnu6, Actb, and Rps29.*

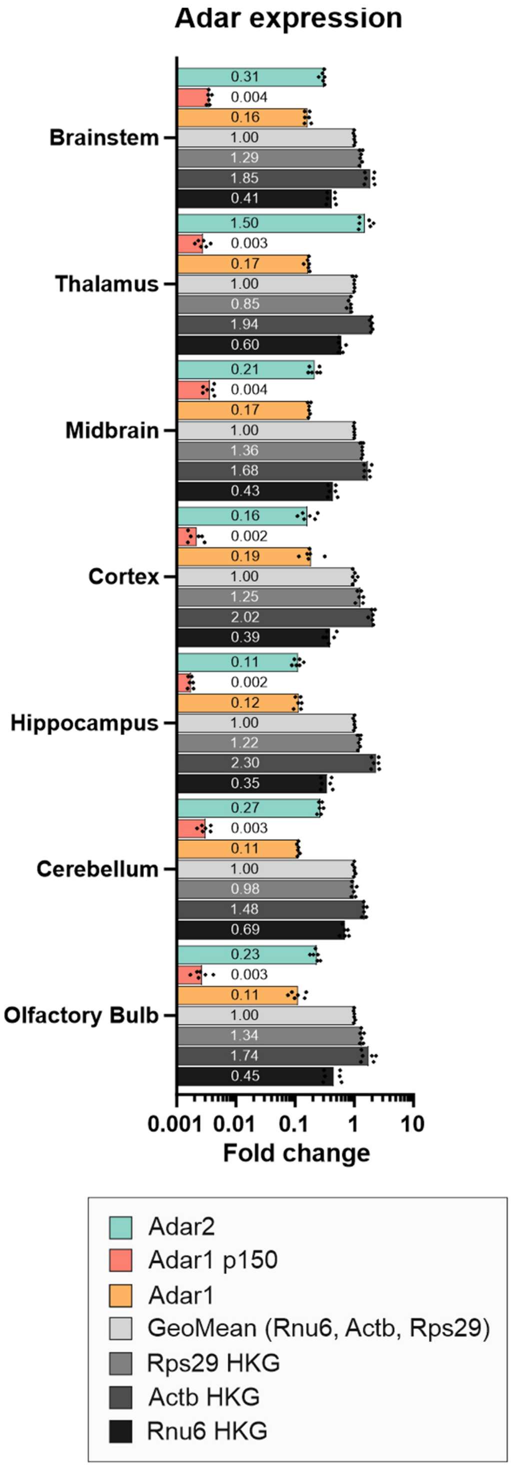

Supplementary Figure S13. Extended in-vivo Adar RT-qPCR dataset. *In-vivo Adar1, Adar1 p150 and Adar2 expression in all analysed brain tissues including all used housekeeping genes. Data from N = 2 targeting guide RNA treated Rett syndrome animals are shown as the median of three technical replicates each. All fold changes are normalized to the geometric mean of the three murine housekeeping genes Rnu6, Actb, and Rps29.*

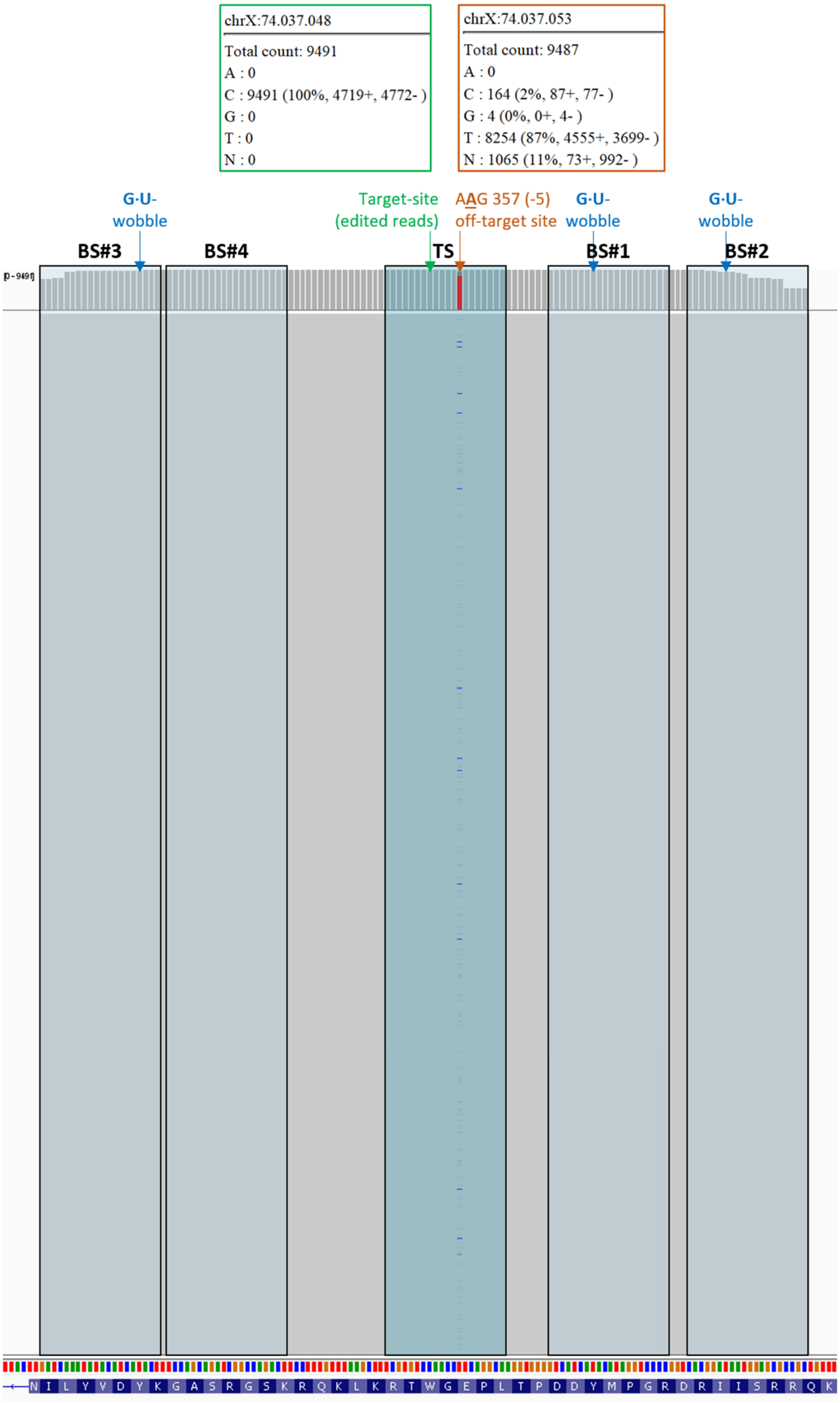

Supplementary Figure S14. Bystander off-target evaluation via amplicon sequencing. *Excerpt of amplicon sequencing results measured in the thalamus of N = 2 circCLUSTER guideRNA-treated Mecp2 W104Amber mice.*

Supplementary Figure S14 continued. Bystander off-target evaluation via amplicon sequencing. *The fastq files were pre-processed using Seqtk trimfq. Base-calls with QV<30 were masked as N. The base-call accuracy of the remaining bases was thus 99.9%. The alignment was performed using BWA-mem against the GRCm38/mm10 reference genome. In two successive trimming steps first soft-clippings and then all sequences outside the guide RNA binding region were removed from all reads. Trimming was performed using a custom Python script. In the next step all reads with successful on-target editing were selected by writing reads with a cytosine at index chrX:74.037.048 into a new file individually for each animal. Finally, these two files were merged using Samtools 1.9. The merged file was sorted and indexed using IGV 2.16.2. The reads are displayed in Quick Consensus Mode with the coverage allele-fraction threshold being set to 0.5%. Only one bystander off-target site was detected at the 5'-AAG-triplet at position 357 in the Mecp2 ORF (-5 position relative to the on-target site). Only 1.73% of the on-target edited reads showed bystander off-target events, almost all at the -5 site. The sites protected by G·U-wobbles showed no detectable bystander off-target editing.*

Supplementary Table S1: List of applied encoded guide RNAs and ASOs. For the ease of use, the encoded guide RNAs are given as coding DNA sequences including all functional elements like e.g. split-R/G-, boxB- or direct repeat-motifs. Circular guide RNAs are given after ribozyme processing. ASOs are given as modified RNA sequences.

Sequence annotation:

Targeting sequence / Specificity domain
Target sequence (in cis-acting reporters)
Cluster of recruitment sequences
R/G-motif
Split-R/G-motif V1
Split-R/G-motif V2
Split-R/G-motif V3
BoxB-motif
DR-motif
BoxB gRNA, DR gRNA and LEAPER targeting sequence
Ligation stems after ribozyme processing
AC50-linker
mN = 2'O-methyl
* = Phosphorothioate linkage

pTS #	Guide RNA Name	Sequence 5'->3'	Used in Figure
**pTS1444**	Cis-acting_systematic_GU-evaluation_AAT-TTA	GGTGTCGAGAAGAGGAGAACAATATGCTAAATGTTGTTCTCGTCTCCTCGACACCCAGTAGATTCAACATGCTGTAAAAAACAGCATGTTGAATCTACTG	1, S1
**pTS1445**	Cis-acting_systematic_GU-evaluation_AAT-TTG	GGTGTCGAGAAGAGGAGAACAATATGCTAAATGTTGTTCTCGTCTCCTCGACACCCAGTAGGTTCAACATGCTGTAAAAAACAGCATGTTGAATCTACTG	1, S1
**pTS1446**	Cis-acting_systematic_GU-evaluation_AAG-TTC	GGTGTCGAGAAGAGGAGAACAATATGCTAAATGTTGTTCTCGTCTCCTCGACACCCAGTAGCTTCAACATGCTGTAAAAAACAGCATGTTGAAGCTACTG	1, S1
**pTS1447**	Cis-acting_systematic_GU-evaluation_AAG-TTT	GGTGTCGAGAAGAGGAGAACAATATGCTAAATGTTGTTCTCGTCTCCTCGACACCCAGTAGTTTCAACATGCTGTAAAAAACAGCATGTTGAAGCTACTG	1, S1
**pTS1448**	Cis-acting_systematic_GU-evaluation_TAA-ATT	GGTGTCGAGAAGAGGAGAACAATATGCTAAATGTTGTTCTCGTCTCCTCGACACCCAGTAGTTACAACATGCTGTAAAAAACAGCATGTTGTAACTACTG	1, S1
**pTS1449**	Cis-acting_systematic_GU-evaluation_TAA-GTT	GGTGTCGAGAAGAGGAGAACAATATGCTAAATGTTGTTCTCGTCTCCTCGACACCCAGTAGTTGCAACATGCTGTAAAAAACAGCATGTTGTAACTACTG	1, S1
**pTS1450**	Cis-acting_systematic_GU-evaluation_TAT-ATA	GGTGTCGAGAAGAGGAGAACAATATGCTAAATGTTGTTCTCGTCTCCTCGACACCCAGTAGATACAACATGCTGTAAAAAACAGCATGTTGTATCTACTG	1, S1
**pTS1451**	Cis-acting_systematic_GU-evaluation_TAT-GTA	GGTGTCGAGAAGAGGAGAACAATATGCTAAATGTTGTTCTCGTCTCCTCGACACCCAGTAGATGCAACATGCTGTAAAAAACAGCATGTTGTATCTACTG	1, S1
**pTS1452**	Cis-acting_systematic_GU-evaluation_TAT-ATG	GGTGTCGAGAAGAGGAGAACAATATGCTAAATGTTGTTCTCGTCTCCTCGACACCCAGTAGGTACAACATGCTGTAAAAAACAGCATGTTGTATCTACTG	1, S1

**pTS1453**	Cis-acting_systematic_GU-evaluation_TAT-GTG	GGTGTCGAGAAGAGGAGAACAATATGCTAAATGTTGTTCTCGTCTCCTCGACACCCAGTAGGTGCAACATGCTGTAAAAAACAGCATGTTGTATCTACTG	1, S1
**pTS1454**	Cis-acting_systematic_GU-evaluation_TAG-ATC	GGTGTCGAGAAGAGGAGAACAATATGCTAAATGTTGTTCTCGTCTCCTCGACACCCAGTAGCTACAACATGCTGTAAAAAACAGCATGTTGTAGCTACTG	1, EDF1, S1
**pTS1455**	Cis-acting_systematic_GU-evaluation_TAG-GTC	GGTGTCGAGAAGAGGAGAACAATATGCTAAATGTTGTTCTCGTCTCCTCGACACCCAGTAGCTGCAACATGCTGTAAAAAACAGCATGTTGTAGCTACTG	1, EDF1, S1
**pTS1456**	Cis-acting_systematic_GU-evaluation_TAG-ATT	GGTGTCGAGAAGAGGAGAACAATATGCTAAATGTTGTTCTCGTCTCCTCGACACCCAGTAGTTACAACATGCTGTAAAAAACAGCATGTTGTAGCTACTG	1, EDF1, S1
**pTS1457**	Cis-acting_systematic_GU-evaluation_TAG-GTT	GGTGTCGAGAAGAGGAGAACAATATGCTAAATGTTGTTCTCGTCTCCTCGACACCCAGTAGTTGCAACATGCTGTAAAAAACAGCATGTTGTAGCTACTG	1, EDF1, S1
**pTS1458**	Cis-acting_systematic_GU-evaluation_TAC-ATG	GGTGTCGAGAAGAGGAGAACAATATGCTAAATGTTGTTCTCGTCTCCTCGACACCCAGTAGGTACAACATGCTGTAAAAAACAGCATGTTGTACCTACTG	1, S1
**pTS1459**	Cis-acting_systematic_GU-evaluation_TAC-GTG	GGTGTCGAGAAGAGGAGAACAATATGCTAAATGTTGTTCTCGTCTCCTCGACACCCAGTAGGTGCAACATGCTGTAAAAAACAGCATGTTGTACCTACTG	1, S1
**pTS1460**	Cis-acting_systematic_GU-evaluation_GAA-CTT	GGTGTCGAGAAGAGGAGAACAATATGCTAAATGTTGTTCTCGTCTCCTCGACACCCAGTAGTTCCAACATGCTGTAAAAAACAGCATGTTGGAACTACTG	S1
**pTS1461**	Cis-acting_systematic_GU-evaluation_GAA-TTT	GGTGTCGAGAAGAGGAGAACAATATGCTAAATGTTGTTCTCGTCTCCTCGACACCCAGTAGTTTCAACATGCTGTAAAAAACAGCATGTTGGAACTACTG	S1
**pTS1462**	Cis-acting_systematic_GU-evaluation_GAT-CTA	GGTGTCGAGAAGAGGAGAACAATATGCTAAATGTTGTTCTCGTCTCCTCGACACCCAGTAGATCCAACATGCTGTAAAAAACAGCATGTTGGATCTACTG	S1
**pTS1463**	Cis-acting_systematic_GU-evaluation_GAT-TTA	GGTGTCGAGAAGAGGAGAACAATATGCTAAATGTTGTTCTCGTCTCCTCGACACCCAGTAGATTCAACATGCTGTAAAAAACAGCATGTTGGATCTACTG	S1
**pTS1464**	Cis-acting_systematic_GU-evaluation_GAT-CTG	GGTGTCGAGAAGAGGAGAACAATATGCTAAATGTTGTTCTCGTCTCCTCGACACCCAGTAGGTCCAACATGCTGTAAAAAACAGCATGTTGGATCTACTG	S1
**pTS1465**	Cis-acting_systematic_GU-evaluation_GAT-TTG	GGTGTCGAGAAGAGGAGAACAATATGCTAAATGTTGTTCTCGTCTCCTCGACACCCAGTAGGTTCAACATGCTGTAAAAAACAGCATGTTGGATCTACTG	S1
**pTS1466**	Cis-acting_systematic_GU-evaluation_GAG-CTC	GGTGTCGAGAAGAGGAGAACAATATGCTAAATGTTGTTCTCGTCTCCTCGACACCCAGTAGCTCCAACATGCTGTAAAAAACAGCATGTTGGAGCTACTG	S1
**pTS1467**	Cis-acting_systematic_GU-evaluation_GAG-TTC	GGTGTCGAGAAGAGGAGAACAATATGCTAAATGTTGTTCTCGTCTCCTCGACACCCAGTAGCTTCAACATGCTGTAAAAAACAGCATGTTGGAGCTACTG	S1
**pTS1468**	Cis-acting_systematic_GU-evaluation_GAG-CTT	GGTGTCGAGAAGAGGAGAACAATATGCTAAATGTTGTTCTCGTCTCCTCGACACCCAGTAGTTCCAACATGCTGTAAAAAACAGCATGTTGGAGCTACTG	S1
**pTS1469**	Cis-acting_systematic_GU-evaluation_GAG-TTT	GGTGTCGAGAAGAGGAGAACAATATGCTAAATGTTGTTCTCGTCTCCTCGACACCCAGTAGTTTCAACATGCTGTAAAAAACAGCATGTTGGAGCTACTG	S1
**pTS1470**	Cis-acting_systematic_GU-evaluation_GAC-CTG	GGTGTCGAGAAGAGGAGAACAATATGCTAAATGTTGTTCTCGTCTCCTCGACACCCAGTAGGTCCAACATGCTGTAAAAAACAGCATGTTGGACCTACTG	S1
**pTS1471**	Cis-acting_systematic_GU-evaluation_GAC-TTG	GGTGTCGAGAAGAGGAGAACAATATGCTAAATGTTGTTCTCGTCTCCTCGACACCCAGTAGGTTCAACATGCTGTAAAAAACAGCATGTTGGACCTACTG	S1
**pTS1472**	Cis-acting_systematic_GU-evaluation_CAT-GTA	GGTGTCGAGAAGAGGAGAACAATATGCTAAATGTTGTTCTCGTCTCCTCGACACCCAGTAGATGCAACATGCTGTAAAAAACAGCATGTTGCATCTACTG	S1
**pTS1473**	Cis-acting_systematic_GU-evaluation_CAT-GTG	GGTGTCGAGAAGAGGAGAACAATATGCTAAATGTTGTTCTCGTCTCCTCGACACCCAGTAGGTGCAACATGCTGTAAAAAACAGCATGTTGCATCTACTG	S1
**pTS1474**	Cis-acting_systematic_GU-evaluation_CAG-GTC	GGTGTCGAGAAGAGGAGAACAATATGCTAAATGTTGTTCTCGTCTCCTCGACACCCAGTAGCTGCAACATGCTGTAAAAAACAGCATGTTGCAGCTACTG	1, S1
**pTS1475**	Cis-acting_systematic_GU-evaluation_CAG-GTT	GGTGTCGAGAAGAGGAGAACAATATGCTAAATGTTGTTCTCGTCTCCTCGACACCCAGTAGTTGCAACATGCTGTAAAAAACAGCATGTTGCAGCTACTG	1, S1

**pTS1484**	Cis-acting_systematic_GU-evaluation_AAT-TGA	GGTGTCGAGAAGAGGAGAACAATATGCTAAATGTTGTTCTCGTCTCCTCGACACCCAGTAGAGTCAACATGCTGTAAAAAACAGCATGTTGAATCTACTG	1, S1
**pTS1485**	Cis-acting_systematic_GU-evaluation_AAG-TGC	GGTGTCGAGAAGAGGAGAACAATATGCTAAATGTTGTTCTCGTCTCCTCGACACCCAGTAGCGTCAACATGCTGTAAAAAACAGCATGTTGAAGCTACTG	S1
**pTS1486**	Cis-acting_systematic_GU-evaluation_TAA-AGT	GGTGTCGAGAAGAGGAGAACAATATGCTAAATGTTGTTCTCGTCTCCTCGACACCCAGTAGTGACAACATGCTGTAAAAAACAGCATGTTGTAACTACTG	1, S1
**pTS1487**	Cis-acting_systematic_GU-evaluation_TAT-AGA	GGTGTCGAGAAGAGGAGAACAATATGCTAAATGTTGTTCTCGTCTCCTCGACACCCAGTAGAGACAACATGCTGTAAAAAACAGCATGTTGTATCTACTG	1, S1
**pTS1488**	Cis-acting_systematic_GU-evaluation_TAG-AGC	GGTGTCGAGAAGAGGAGAACAATATGCTAAATGTTGTTCTCGTCTCCTCGACACCCAGTAGCGACAACATGCTGTAAAAAACAGCATGTTGTAGCTACTG	1, EDF1, S1
**pTS1489**	Cis-acting_systematic_GU-evaluation_TAC-AGG	GGTGTCGAGAAGAGGAGAACAATATGCTAAATGTTGTTCTCGTCTCCTCGACACCCAGTAGGGACAACATGCTGTAAAAAACAGCATGTTGTACCTACTG	1, S1
**pTS1490**	Cis-acting_systematic_GU-evaluation_GAA-CGT	GGTGTCGAGAAGAGGAGAACAATATGCTAAATGTTGTTCTCGTCTCCTCGACACCCAGTAGTGCCAACATGCTGTAAAAAACAGCATGTTGGAACTACTG	S1
**pTS1491**	Cis-acting_systematic_GU-evaluation_GAT-CGA	GGTGTCGAGAAGAGGAGAACAATATGCTAAATGTTGTTCTCGTCTCCTCGACACCCAGTAGAGCCAACATGCTGTAAAAAACAGCATGTTGGATCTACTG	S1
**pTS1492**	Cis-acting_systematic_GU-evaluation_GAG-CGC	GGTGTCGAGAAGAGGAGAACAATATGCTAAATGTTGTTCTCGTCTCCTCGACACCCAGTAGCGCCAACATGCTGTAAAAAACAGCATGTTGGAGCTACTG	S1
**pTS1493**	Cis-acting_systematic_GU-evaluation_GAC-CGG	GGTGTCGAGAAGAGGAGAACAATATGCTAAATGTTGTTCTCGTCTCCTCGACACCCAGTAGGGCCAACATGCTGTAAAAAACAGCATGTTGGACCTACTG	S1
**pTS1494**	Cis-acting_systematic_GU-evaluation_CAT-GGA	GGTGTCGAGAAGAGGAGAACAATATGCTAAATGTTGTTCTCGTCTCCTCGACACCCAGTAGAGGCAACATGCTGTAAAAAACAGCATGTTGCATCTACTG	S1
**pTS1495**	Cis-acting_systematic_GU-evaluation_CAG-GGC	GGTGTCGAGAAGAGGAGAACAATATGCTAAATGTTGTTCTCGTCTCCTCGACACCCAGTAGCGGCAACATGCTGTAAAAAACAGCATGTTGCAGCTACTG	S1
**pTS1909**	Cis-acting_NN_ttaAgtt-aatCcaa	CCATATATATTAAGTTATCGGGTGTCGAGAAGAGGAGAACAATATGCTAAATGTTGTTCTCGTCTCCTCGACACCCGATAACCTAATATATATGG	EDF3, S4
**pTS1910**	Cis-acting_NN_ttaAgtt-aGtCcaa	CCATATATATTAAGTTATCGGGTGTCGAGAAGAGGAGAACAATATGCTAAATGTTGTTCTCGTCTCCTCGACACCCGATAACCTGATATATATGG	EDF3, S4
**pTS1911**	Cis-acting_NN_ttaAgtt-GatCcaa	CCATATATATTAAGTTATCGGGTGTCGAGAAGAGGAGAACAATATGCTAAATGTTGTTCTCGTCTCCTCGACACCCGATAACCTAGTATATATGG	S4
**pTS1912**	Cis-acting_NN_ttaAgtt-aaGCcaa	CCATATATATTAAGTTATCGGGTGTCGAGAAGAGGAGAACAATATGCTAAATGTTGTTCTCGTCTCCTCGACACCCGATAACCGAATATATATGG	EDF3, S4
**pTS1913**	Cis-acting_NN_tttAatt-aaaCtaa	CCATATATATTTAATTATCGGGTGTCGAGAAGAGGAGAACAATATGCTAAATGTTGTTCTCGTCTCCTCGACACCCGATAATCAAATATATATGG	EDF3, S4
**pTS1914**	Cis-acting_NN_tttAatt-aaaCtGa	CCATATATATTTAATTATCGGGTGTCGAGAAGAGGAGAACAATATGCTAAATGTTGTTCTCGTCTCCTCGACACCCGATAGTCAAATATATATGG	EDF3, S4
**pTS1915**	Cis-acting_NN_tttAatt-aaaCtaG	CCATATATATTTAATTATCGGGTGTCGAGAAGAGGAGAACAATATGCTAAATGTTGTTCTCGTCTCCTCGACACCCGATGATCAAATATATATGG	S4
**pTS1916**	Cis-acting_NN_tttAatt-aaaCGaa	CCATATATATTTAATTATCGGGTGTCGAGAAGAGGAGAACAATATGCTAAATGTTGTTCTCGTCTCCTCGACACCCGATAAGCAAATATATATGG	EDF3, S4
**pTS1917**	Cis-acting_2nd-NN_tatAaat-ataCtta	CCATATATATATAAATATCGGGTGTCGAGAAGAGGAGAACAATATGCTAAATGTTGTTCTCGTCTCCTCGACACCCGATATTCATATATATATGG	S4
**pTS1918**	Cis-acting_2nd-NN_tatAaat-ataCttG	CCATATATATATAAATATCGGGTGTCGAGAAGAGGAGAACAATATGCTAAATGTTGTTCTCGTCTCCTCGACACCCGATGTTCATATATATATGG	S4
**pTS1919**	Cis-acting_2nd-NN_tatAaat-ataCtGa	CCATATATATATAAATATCGGGTGTCGAGAAGAGGAGAACAATATGCTAAATGTTGTTCTCGTCTCCTCGACACCCGATAGTCATATATATATGG	S4

**pTS1920**	Cis-acting_2nd-NN_taaAgat-attCcta	CCATATATATAAAGATATCGGGTGTCGAGAAGAGGAGAACAATATGCTAAATGTTGTTCTCGTCTCCTCGACACCCGATATCCTTATATATATGG	S4
**pTS1921**	Cis-acting_2nd-NN_taaAgat-GttCcta	CCATATATATAAAGATATCGGGTGTCGAGAAGAGGAGAACAATATGCTAAATGTTGTTCTCGTCTCCTCGACACCCGATATCCTTGTATATATGG	S4
**pTS1922**	Cis-acting_2nd-NN_taaAgat-aGtCcta	CCATATATATAAAGATATCGGGTGTCGAGAAGAGGAGAACAATATGCTAAATGTTGTTCTCGTCTCCTCGACACCCGATATCCTGATATATATGG	S4
**pTS1923**	Cis-acting_3nd-NN_taatAaaat-attaCttta	CCATATATTAATAAAATTCGGGTGTCGAGAAGAGGAGAACAATATGCTAAATGTTGTTCTCGTCTCCTCGACACCCGAATTTCATTAATATATGG	S4
**pTS1924**	Cis-acting_3nd-NN_taatAaaat-attaCtttG	CCATATATTAATAAAATTCGGGTGTCGAGAAGAGGAGAACAATATGCTAAATGTTGTTCTCGTCTCCTCGACACCCGAGTTTCATTAATATATGG	S4
**pTS1925**	Cis-acting_3nd-NN_taatAaaat-attaCttGa	CCATATATTAATAAAATTCGGGTGTCGAGAAGAGGAGAACAATATGCTAAATGTTGTTCTCGTCTCCTCGACACCCGAAGTTCATTAATATATGG	S4
**pTS1926**	Cis-acting_3nd-NN_taaaAgaat-atttCctta	CCATATATTAAAAGAATTCGGGTGTCGAGAAGAGGAGAACAATATGCTAAATGTTGTTCTCGTCTCCTCGACACCCGAATTCCTTTAATATATGG	S4
**pTS1927**	Cis-acting_3nd-NN_taaaAgaat-GtttCctta	CCATATATTAAAAGAATTCGGGTGTCGAGAAGAGGAGAACAATATGCTAAATGTTGTTCTCGTCTCCTCGACACCCGAATTCCTTTGATATATGG	S4
**pTS1928**	Cis-acting_3nd-NN_taaaAgaat-aGttCctta	CCATATATTAAAAGAATTCGGGTGTCGAGAAGAGGAGAACAATATGCTAAATGTTGTTCTCGTCTCCTCGACACCCGAATTCCTTGAATATATGG	S4
**pTS1717**	AHI1_LEAPER_gRNA	GTGAACGTCAAACTGTCGGACCAATATGGCAGAATCTTCTCTCATCTCAACTTTCCATATCCGTATCATGGAATCATAGCATCCTGTAACTACTAGCTCTCTTACAGCTGG	2
**pTS1758**	AHI1_LEAPER_GU_at_GU_amenable_sites_gRNA	GTGAACGTCAAACTGTCGGACCAATGTGGCAGAATCTTCTCTCATCTCAACTTTCCATGTCCGTGTCATGGAATCATGGCATCCTGTGACTGCTGGCTCTCTTGCAGCTGG	2
**pTS1791**	AHI1_LEAPER_GA_at_GU_amenable_sites_gRNA	GTGAACGTCAAACTGTCGGACCAAGAGGGCAGAATCTTCTCTCATCTCAACTTTCCAGATCCGGATCATGGAATCAGAGCATCCTGGAACGACGAGCTCTCTGACAGCTGG	2
**pTS1757**	AHI1_LEAPER_GA_at_all_off-target_sites_gRNA	GTGAACGTCAAACTGTCGGACCAAGAGGGCAGAATCGTCGCGCATCGCAACGGTCCAGATCCGGATCATGGAATCAGAGCATCCGGGAACGACGAGCTCTCGGACAGCTGG	2
**pTS1818**	AHI1_LEAPER_GU&GA_at_all_off-target_sites_gRNA	GTGAACGTCAAACTGTCGGACCAATGTGGCAGAATCGTCGCGCATCTCAGCTTTCCATGTCCGTGTCATGGAATCATGGCATCCGGTGACTGCTGGCTCTCGTGCAGCTGG	2
**pTS1718**	BMPR2_LEAPER_gRNA	GTGAAGATAAGCCAGTCCTCTAGTAACAGAATGAGCAAGACGGCAAGAGCTTACCCAGTCACTTGTGTGGAGACTTAAATACTTGCATAAAGATCCATTGGGATAGTACTC	2
**pTS1752**	BMPR2_LEAPER_GU_at_GU_amenable_sites_gRNA	GTGAAGATAAGCCAGTCCTCTGGTGACAGAATGAGCAAGACGGCAAGAGCTTGCCCAGTCACTTGTGTGGAGACTTGAATGCTTGCATGAAGATCCGTTGGGATGGTGCTC	2
**pTS1753**	BMPR2_LEAPER_GA_at_GU_amenable_sites_gRNA	GTGAAGATAAGCCAGTCCTCGAGGAACAGAATGAGCAAGACGGCAAGAGCTGACCCAGTCACTTGTGTGGAGACTGAAAGACTTGCAGAAAGATCCAGTGGGAGAGGACTC	2
**pTS1751**	BMPR2_LEAPER_GA_at_all_off-target_sites_gRNA	GTGAAGATAAGCCAGTCCTCGAGGAACAGAATGAGCAAGACGGCAAGAGCGGACCCAGTCACGGGTGTGGAGACGGAAAGACGGGCAGAAAGATCCAGTGGGAGAGGACTC	2
**pTS1827**	BMPR2_LEAPER_GU&GA_at_all_off-target_sites_gRNA	GTGAAGATAAGCCAGTCCTCTGGTGACAGAATGAGCAAGACGGCAAGAGCGGACCCAGTCACGGGTGTGGAGACGTGAATGCGGGCATGAAGATCCGTTGGGATGGTGCTC	2
**pTS1760**	COL3A1_LEAPER_gRNA	CATATTACAGAATACCTTGATAGCATCCAATTTGCATCCTTGGTTAGGGTCAACCCAGTATTCTCCACTCTTGAGTTCAGGATGGCAGAATTTCAGGTCTCTGCAGTTTCT	2
**pTS1821**	COL3A1_LEAPER_GU_at_GU_amenable_sites_gRNA	CATATTACAGAATACCTTGGTAGCATCCAGTTTGCATCCTTGGTTAGGGTCAACCCAGTGTTCTCCACTCTTGAGTTCAGGGTGGCAGAGTTTCAGGTCTCTGCAGTTTCT	2
**pTS1822**	COL3A1_LEAPER_GA_at_GU_amenable_sites_gRNA	CATATTACAGAATACCTTGAGAGCATCCAAGTTGCATCCTTGGTTAGGGTCAACCCAGGAGTCTCCACTCTTGAGTTCAGGAGGGCAGAAGTTCAGGTCTCTGCAGTTTCT	2
**pTS1823**	COL3A1_LEAPER_GA_at_all_off-target_sites_gRNA	CATATTACAGAATACCTTGAGAGCATCCAAGGTGCATCCTTGGTTAGGGTCAACCCAGGAGTCTCCACTCGTGAGTTCAGGAGGGCAGAAGGTCAGGTCTCGGCAGGTTCT	2

**pTS1824**	COL3A1_LEAPER_GU&GA_at_all_off-target_sites_gRNA	CATATTACAGAATACCTTGGTAGCATCCAGTGTGCATCCTTGGTTAGGGTCAACCCAGTGTTCTCCACTCGTGAGTTCAGGGTGGCAGAGTGTCAGGTCTCTGCAGGTTCT	2
**pTS1193**	RAB7A_LEAPER_gRNA	GTCTTTGATAAAAGGCGTACATAATTCTTGTGTCTACTGTACAGAATACTGCCGCCAGCTGGATTTCCCAATTCTGAGTAACACTCTGCAATCCAAACAGGGTTCAACCCT	EDF2
**pTS1801**	RAB7A_LEAPER_GU_at_GU_amenable_sites_gRNA	GTCTTTGATGAAAGGCGTGCGTAGTTCTTGTGTCTGCTGTGCAGAATGCTGCCGCCAGCTGGGTTTCCCAGTTCTGAGTGACACTCTGCAATCCAAACAGGGTTCAACCCT	EDF2
**pTS1802**	RAB7A_LEAPER_GA_at_GU_amenable_sites_gRNA	GTCTTTGAGAAAAGGCGGACAGAAGTCTTGTGTCGACTGGACAGAAGACTGCCGCCAGCTGGAGTTCCCAAGTCTGAGGAACACTCTGCAATCCAAACAGGGTTCAACCCT	EDF2
**pTS1216**	RAB7A_LEAPER_GA_at_all_off-target_sites_gRNA	GTCTTTGAGAAAAGGCGGACAGAAGTCTGGTGTCGACGGGACAGAAGACGGCCGCCAGCTGGAGGGCCCAAGGCGGAGGAACACTCGGCAATCCAAACAGGGGGCAACCCT	EDF2
**pTS1803**	RAB7A_LEAPER_GU&GA_at_all_off-target_sites_gRNA	GTCTTTGATGAAAGGCGTGCGTAGTTCGGGTGTCTGCTGTGCAGAATGCGGCCGCCAGCTGGGTGGCCCAGTGCGGAGTGACACGCGGCAATCCAAACAGGGGGCAACCCT	EDF2
**pTS1964**	mMecp2_LEAPER_Linear_gRNA	TACATCATACTTTCCAGCAGATCGGCCAGACTTCCTTTGTTTAAGCTTTCGTGTCCAACCTTCAGGCAAGGTGGGGTCATCATACATAGGTCCCCGGTCACGGATAATGGA	EDF5
**pTS2031**	mMecp2_LEAPER_Circular_gRNA	AACCATGCCGACTGATGGCAGTACATCATACTTTCCAGCAGATCGGCCAGACTTCCTTTGTTTAAGCTTTCGTGTCCAACCTTCAGGCAAGGTGGGGTCATCATACATAGGTCCCCGGTCACGGATAATGGACTGCCATCAGTCGGCGTGGACTGTAG	5, EDF5
**pTS2529**	mMecp2_LEAPER_Circular_gRNA_GA_at_GU_amenable_sites	AACCATGCCGACTGATGGCAGTACATCATACTTTCCAGCAGATCGGCCAGACTTCCTTTGTTgAAGCTTTCGTGTCcAACCTTCAGGCAAGGTGGGGTCATCAgACAgAGGTCCCCGGTCACGGAgAATGGACTGCCATCAGTCGGCGTGGACTGTAG	5, EDF5
**pTS2530**	mMecp2_LEAPER_Circular_gRNA_Uridine-depletion_at_GU_amenable_sites	AACCATGCCGACTGATGGCAGTACATCATACTTTCCAGCAGATCGGCCAGACTTCCTTTGTTAAGCTTTCGTGTCcAACCTTCAGGCAAGGTGGGGTCATCAACAAGGTCCCCGGTCACGGAAATGGACTGCCATCAGTCGGCGTGGACTGTAG	5, EDF5
**pTS2033**	mMecp2_LEAPER_Circular_GA_at_all_off-target_sites_gRNA	AACCATGCCGACTGATGGCAGTACATCATACTTTCCAGCAGATCGGCCAGACTTCCGGTGTTGAAGCGGTCGTGTCCAACCGTCAGGCAAGGTGGGGGCATCAGACAGAGGTCCCCGGTCACGGAGAATGGACTGCCATCAGTCGGCGTGGACTGTAG	5, EDF5
**pTS2034**	mMecp2_LEAPER_Circular_U-depletion_gRNA	AACCATGCCGACTGATGGCAGTACATCATACTTTCCAGCAGATCGGCCAGACTTCCTGTTAAGCTCGTGTCcAACCTCAGGCAAGGTGGGGCATCAACAAGGTCCCCGGTCACGGAAATGGACTGCCATCAGTCGGCGTGGACTGTAG	5, EDF5
**pTS2035**	mMecp2_LEAPER_Circular_GU-only_gRNA	AACCATGCCGACTGATGGCAGTACATCATACTTTCCAGCAGATCGGCCAGACTTCCTTTGTTTgAGCTTTCGTGTCcAACCTTCAGGCAAGGTGGGGTCATCATgCATgGGTCCCCGGTCACGGATgATGGACTGCCATCAGTCGGCGTGGACTGTAG	5, EDF5
**pTS2037**	mMecp2_LEAPER_Circular_GU&U-depletion_gRNA	AACCATGCCGACTGATGGCAGTACATCATACTTTCCAGCAGATCGGCCAGACTTCCTGTTTgAGCTCGTGTCcAACCTCAGGCAAGGTGGGGCATCATgCATgGGTCCCCGGTCACGGATgATGGACTGCCATCAGTCGGCGTGGACTGTAG	5, EDF5
**pTS2036**	mMecp2_LEAPER_Circular_GU&GA_at_all_off-target_sites_gRNA	AACCATGCCGACTGATGGCAGTACATCATACTTTCCAGCAGATCGGCCAGACTTCCGGTGTTTGAGCGGTCGTGTCCAACCGTCAGGCAAGGTGGGGGCATCATGCATGGGTCCCCGGTCACGGATGATGGACTGCCATCAGTCGGCGTGGACTGTAG	5, EDF5
**pTS2043**	mMecp2_W104Amber_Linear_3x20-20p8_RG-V21 (V45 mMecp2 gRNA)	GGTGTCGAGAAGAGGAGAACAATATCTTTCATGTTGTTCTCCTCTCCTCGACACCTCGTGTCCAACCTTCAGGCAAAAGTCATCATACATAGGTCCCCAAACACGGATAATGGAGCGCCGCAAATTGGGGGAAGCCGAGGCTTC	EDF5
**pTS2058**	mMecp2_W104Amber_Linear_3x20-20p8_RG-V21_GU-wobbles (V45.2 mMecp2 gRNA)	GGTGTCGAGAAGAGGAGAACAATATGCTAAATGTTGTTCTCGTCTCCTCGACACCTCGTGTCCAACCTTCAGGCAAAAGTCATCATACATAGGTCCCCAAACACGGATGATGGAGCGCCGCAAATTGGGGGAAGCCGAGGCTTC	EDF5
**pTS2023**	mMecp2_W104Amber_Circular_6x20-20p8_NoRG (V37 mMecp2 gRNA)	AACCATGCCGACTGATGGCAGAAAAATCAAAATCATTAGGGTCCAAAAATTCAAAGTATGCAATCAATTAAATCCAGCAGATCGGCCAGACTAAATCGTGTCCAACCTTCAGGCAAAAGTCATCATACATAGGTCCCCAAACACGGATAATGGAGCGCCGCAAATTGGGGGAAGCCGAGGCTTCAAAAACTGCCATCAGTCGGCGTGGACTGTAG	EDF5
**pTS2055**	mMecp2_W104Amber_Circular_6x20-	AACCATGCCGACTGATGGCAGAAAAATCAAAATCATTAGGGTCCAAAAATTCAAAGTGTGCAATCAATTAAATCCAGCAGATCGG	EDF5

	20p8_NoRG_GU-wobbles (V37.2 mMecp2 gRNA)	CCAGACTAAATCGTGTCCAACCTTCAGGCAAAAGTCATCATAC<br>ATAGGTCCCCAAACACGGATGATGGAGCGCCGCAAATTGGG<br>GGAAGCCGAGGCTTCAAAAACTGCCATCAGTCGGCGTGGAC<br>TGTAG	
**pTS2228**	mMecp2_W104Amber_Linear_4x20-20p8_Split-RG-V3_GU-wobbles (Linear V27.2.4 mMecp2 gRNA)	GCATGTTGTTCTCGTCTCCTCGACACCTCGTGTCCAACCTTCA<br>GGCAAAAAGTCATCATGCATAGGTCCCCAAACACGGATGATG<br>GAGCGCCGCAAAGATCAAATATACATCGTACTAAATCCAGCA<br>GATCGGCCAGACTAAAAAGGTGTCGAGAAGAGGAGAACAATA<br>TCTTT	EDF5-6
**pTS2013**	mMecp2_W104Amber_Circular_4x20-20p8_Split-RG-V2 (V27 mMecp2 gRNA)	AACCATGCCGACTGATGGCAGAATGTTGTTCTCGTCTCCTCG<br>ACACCTCGTGTCCAACCTTCAGGCAAAAAGTCATCATACATAG<br>GTCCCCAAACACGGATAATGGAGCGCCGCAAAGATCAAATAT<br>ACATCATACTAAATCCAGCAGATCGGCCAGACTAAAAAGGTG<br>TCGAGAAGAGGAGAACAATATGCTACTGCCATCAGTCGGCGT<br>GGACTGTAG	EDF5
**pTS2336**	mMecp2_W104Amber_Circular_4x20-20p8_Split-RG-V3_gRNA-V1 (Previous Filter)	AACCATGCCGACTGATGGCAGCATGTTGTTCTCGTCTCCTCG<br>ACACCTCGTGTCCAACCTTCAGGCAAAATCCTCCAGTCTCTC<br>CTCCTCAAACCTCCGCTCGGCGCGGCGGCAAAAACTCTCTCG<br>GTCACGGGCGAAATCCTCAGAGCTCTCAGGCTCAAAAAGGTG<br>TCGAGAAGAGGAGAACAATATCTTTCTGCCATCAGTCGGCGT<br>GGACTGTAG	4
**pTS2337**	mMecp2_W104Amber_Circular_4x20-20p8_Split-RG-V3_gRNA-V2 (Previous Filter)	AACCATGCCGACTGATGGCAGCATGTTGTTCTCGTCTCCTCG<br>ACACCTCGTGTCCAACCTTCAGGCAAAACTCTCCTCCTCGCC<br>TCCTCCAAACCTCCGCTCGGCGCGGCGGCAAATCCTCAGAG<br>CTCTCAGGCTCAAAGGAGGGGCTCCCTCTCCCAGAAAAAGGT<br>GTCGAGAAGAGGAGAACAATATCTTTCTGCCATCAGTCGGCG<br>TGGACTGTAG	4
**pTS2051**	mMecp2_W104Amber_Circular_4x20-20p8_Split-RG-V3_gRNA-V3 (Latest Filter)	AACCATGCCGACTGATGGCAGCATGTTGTTCTCGTCTCCTCG<br>ACACCTCGTGTCCAACCTTCAGGCAAAAAGTCATCATACATAG<br>GTCCCCAAACACGGATAATGGAGCGCCGCAAAGATCAAATAT<br>ACATCATACTAAATCCAGCAGATCGGCCAGACTAAAAAGGTG<br>TCGAGAAGAGGAGAACAATATCTTTCTGCCATCAGTCGGCGT<br>GGACTGTAG	4, 5, EDF5
**pTS2082**	mMecp2_W104Amber_Circular_4x20-20p8_Split-RG-V3_2xGU-wobbles (V27.2.2 mMecp2 gRNA)	AACCATGCCGACTGATGGCAGCATGTTGTTCTCGTCTCCTCG<br>ACACCTCGTGTCcAACCTTCAGGCAAAAAGTCATCATgCATAG<br>GTCCCCAAACACGGATgATGGAGCGCCGCAAAGATCAAATAT<br>ACATCATACTAAATCCAGCAGATCGGCCAGACTAAAAAGGTG<br>TCGAGAAGAGGAGAACAATATCTTTCTGCCATCAGTCGGCGT<br>GGACTGTAG	EDF5
**pTS2108 / Targeting virus**	mMecp2_W104Amber_Circular_4x20-20p8_Split-RG-V3_3xGU-wobbles (V27.2.4 mMecp2 gRNA)	AACCATGCCGACTGATGGCAGCATGTTGTTCTCGTCTCCTCG<br>ACACCTCGTGTCCAACCTTCAGGCAAAAAGTCATCATGCATA<br>GGTCCCCAAACACGGATGATGGAGCGCCGCAAAGATCAAATA<br>TACATCGTACTAAATCCAGCAGATCGGCCAGACTAAAAAGGT<br>GTCGAGAAGAGGAGAACAATATCTTTCTGCCATCAGTCGGCG<br>TGGACTGTAG	5, 6, EDF5-9,S11-14
pTS2191 / **Non-targeting virus**	Scrambled_mMecp2_W104Amber_Circular_4x20-20p8_Split-RG-V3_(Scrambled V27.2.4 mMecp2 gRNA)	AACCATGCCGACTGATGGCAGCATGTTGTTCTCGTCTCCTCG<br>ACACCGGGCTTATACCCGACCTACTAAAAGGACTGTCTCGAC<br>CTCTACAAAAGGGCGACCCCGATGACAGGTAAAGTTAACATA<br>CTCCTAGATAAAAAGCGACACCGCTGCTGACATAAAAAAGGT<br>GTCGAGAAGAGGAGAACAATATCTTTCTGCCATCAGTCGGCG<br>TGGACTGTAG	6, EDF7-8
**pTS2109**	mMecp2_W104Amber_Circular_4x20-20p8_Split-RG-V3_3xGU-wobbles (V27.2.5 mMecp2 gRNA)	AACCATGCCGACTGATGGCAGCATGTTGTTCTCGTCTCCTCG<br>ACACCTCGTGTCcAACCTTCAGGCAAAAAGTCATCATgCATAG<br>GTCCCCAAACACGGATgATGGAGCGCCGCAAAGATCAAATAT<br>ACATCATgCTAAATCCAGCAGATCGGCCAGACTAAAAAGGTGT<br>CGAGAAGAGGAGAACAATATCTTTCTGCCATCAGTCGGCGTG<br>GACTGTAG	EDF5
**pTS2521**	mMecp2_W104Amber_Circular_4x20-20p8_Split-RG-V3_3xGU-wobbles_1xGA-mismatch (V27.2.6 mMecp2 gRNA)	AACCATGCCGACTGATGGCAGCATGTTGTTCTCGTCTCCTCG<br>ACACCTCGTGTCCAACCGTCAGGCAAAAAGTCATCATGCATA<br>GGTCCCCAAACACGGATGATGGAGCGCCGCAAAGATCAAATA<br>TACATCGTACTAAATCCAGCAGATCGGCCAGACTAAAAAGGT<br>GTCGAGAAGAGGAGAACAATATCTTTCTGCCATCAGTCGGCG<br>TGGACTGTAG	5, EDF5
**pTS2522**	mMecp2_W104Amber_Circular_4x20-20p8_Split-RG-V3_3xGU-wobbles_1xUridine-	AACCATGCCGACTGATGGCAGCATGTTGTTCTCGTCTCCTCG<br>ACACCTCGTGTCCAACCTCAGGCAAAAAGTCATCATGCATAG<br>GTCCCCAAACACGGATGATGGAGCGCCGCAAAGATCAAATAT<br>ACATCGTACTAAATCCAGCAGATCGGCCAGACTAAAAAGGTG	5, EDF5

	depletion (V27.2.7 mMecp2 gRNA)	TCGAGAAGAGGAGAACAATATCTTTCTGCCATCAGTCGGCGTGGACTGTAG	
**pTS1885**	AHI1_2xBoxB_10(3)-29p15-(3)10_gRNA	GCAGAATCTTGGCCCTGAAAAAGGGCCTCATCTCAACTTTCCATATCCGTATCATGGGCCCTGAAAAAGGGCCTCATAGCATC	3
**pTS1969**	AHI1_2xBoxB_10(3)-29p15-(3)10_GU_at_GU_amenable_sites_gRNA	GCAGAATCTTGGCCCTGAAAAAGGGCCTCATCTCAACTTTCCATGTCCGTGTCATGGGCCCTGAAAAAGGGCCTCATAGCATC	3
**pTS1968**	AHI1_2xBoxB_10(3)-29p15-(3)10_GA_at_GU_amenable_sites_gRNA	GCAGAATCTTGGCCCTGAAAAAGGGCCTCATCTCAACTTTCCAGATCCGGATCATGGGCCCTGAAAAAGGGCCTCATAGCATC	3
**pTS1967**	AHI1_2xBoxB_10(3)-29p15-(3)10_GA_at_all_off-target_sites_gRNA	GCAGAATCTTGGCCCTGAAAAAGGGCCTCATCGCAACGGTCCAGATCCGGATCATGGGCCCTGAAAAAGGGCCTCATAGCATC	3
**pTS1970**	AHI1_2xBoxB_10(3)-29p15-(3)10_GU&GA_at_all_off-target_sites_gRNA	GCAGAATCTTGGCCCTGAAAAAGGGCCTCATCGCAACGGTCCATGTCCGTGTCATGGGCCCTGAAAAAGGGCCTCATAGCATC	3
**pTS1973**	AHI1_DR_gRNA	CTCTCATCTCAACTTTCCATATCCGTATCATGGAATCATAGCATCCTGTAAGTTGTGGAAGGTCCAGTTTTGAGGGGCTATTACAAC	3
**pTS1976**	AHI1_DR_GU_at_GU_amenable_sites_gRNA	CTCTCATCTCAACTTTCCATGTCCGTGTCATGGAATCATAGCATCCTGTAAGTTGTGGAAGGTCCAGTTTTGAGGGGCTATTACAAC	3
**pTS1975**	AHI1_DR_GA_at_GU_amenable_sites_gRNA	CTCTCATCTCAACTTTCCAGATCCGGATCATGGAATCATAGCATCCTGTAAGTTGTGGAAGGTCCAGTTTTGAGGGGCTATTACAAC	3
**pTS1974**	AHI1_DR_GA_at_all_off-target_sites_gRNA	CTCTCATCTCAACGGTCCAGATCCGGATCATGGAATCATAGCATCCTGTAAGTTGTGGAAGGTCCAGTTTTGAGGGGCTATTACAAC	3
**pTS1977**	AHI1_DR_GU&GA_at_all_off-target_sites_gRNA	CTCTCATCTCAACGGTCCATGTCCGTGTCATGGAATCATAGCATCCTGTAAGTTGTGGAAGGTCCAGTTTTGAGGGGCTATTACAAC	3
**pTS1997**	BMPR2_K983_3x20-20p8_RG-V21_Reference	GGTGTCGAGAAGAGGAGAACAATATGCTAAATGTTGTTCTCGTCTCCTCGACACCCCACCGCCTAAGAGAATAGGAAATGCTGCCATCCAGGACATTTAAACTGAAAGATCCAGAGAATTAAAAGAACACCCTGTGCAAGAACA	EDF3, S5
**pTS2000**	BMPR2_K983_3x20-20p8_RG-V21_GU	GGTGTCGAGAAGAGGAGAACAATATGCTAAATGTTGTTCTCGTCTCCTCGACACCCCACCGCCTGAGAGAATAGGAAATGCTGCCATCCAGGACATTTAAACTGAAAGATCCAGAGAATTAAAAGAACACCCTGTGCAAGAACA	EDF3, S5
**pTS1998**	BMPR2_K983_3x20-20p8_RG-V21_GA	GGTGTCGAGAAGAGGAGAACAATATGCTAAATGTTGTTCTCGTCTCCTCGACACCCCACCGCCGAAGAGAATAGGAAATGCTGCCATCCAGGACATTTAAACTGAAAGATCCAGAGAATTAAAAGAACACCCTGTGCAAGAACA	EDF3, S5
**pTS1999**	BMPR2_K983_3x20-19p8_RG-V21_U-depletion	GGTGTCGAGAAGAGGAGAACAATATGCTAAATGTTGTTCTCGTCTCCTCGACACCCCACCGCCAAGAGAATAGGAAATGCTGCCATCCAGGACATTTAAACTGAAAGATCCAGAGAATTAAAAGAACACCCTGTGCAAGAACA	EDF3, S5
**pTS2002**	COL3A1_N1244_3x20-20p8_RG-V21_Reference	GGTGTCGAGAAGAGGAGAACAATATGCTAAATGTTGTTCTCGTCTCCTCGACACCTGTCCATCAACAGACTTGAGAAAACGGGGCAAAACCGCCAGCTAAATCACCTCCAATCCCAGCAATAAACTCCAACACCACCACAGCAA	EDF3, S5
**pTS2005**	COL3A1_N1244_3x20-20p8_RG-V21_GU	GGTGTCGAGAAGAGGAGAACAATATGCTAAATGTTGTTCTCGTCTCCTCGACACCTGTCCGTCAACAGACTTGAGAAAACGGGGCAAAACCGCCAGCTAAATCACCTCCAATCCCAGCAATAAACTCCAACACCACCACAGCAA	EDF3, S5
**pTS2003**	COL3A1_N1244_3x20-20p8_RG-V21_GA	GGTGTCGAGAAGAGGAGAACAATATGCTAAATGTTGTTCTCGTCTCCTCGACACCTGTCCAGCAACAGACTTGAGAAAACGGGGCAAAACCGCCAGCTAAATCACCTCCAATCCCAGCAATAAACTCCAACACCACCACAGCAA	EDF3, S5
**pTS2004**	COL3A1_N1244_3x20-20p8_RG-V21_ U-depletion	GGTGTCGAGAAGAGGAGAACAATATGCTAAATGTTGTTCTCGTCTCCTCGACACCTGTCCACAACAGACTTGAGAAAACGGGGCAAAACCGCCAGCTAAATCACCTCCAATCCCAGCAATAAACTCCAACACCACCACAGCAA	EDF3, S5
**pTS1764**	NUP43_V233V_3x20-20p8_RG-V25_CA-only	GGTGTCGAGAAGAGGAGAACAATATCTTTCATGTTGTTCTCCTCTCCTCGACACCCAGTAGCCACAACATGCTGTAAAGGCACTC	S2

		GGTCACCAGTCAGAAAAAACCCTCCATCAGAGTCCAAAAAGG<br>CAGCGGTCGCCAGCGGG	
**pTS1766**	NUP43_V233V_3x20-20p8_RG-V25_UG&CA	GGTGTCGAGAAGAGGAGAACAATATCTTTCATGTTGTTCTCCT<br>CTCCTCGACACCCAGTAGTCACAACATGCTGTAAAGGCACTC<br>GGTCACCAGTCAGAAAAAACCCTCCATCAGAGTCCAAAAAGG<br>CAGCGGTCGCCAGCGGG	S2
**pTS1805**	NUP43_V233V_3x20-20p8_RG-V25_UG-only	GGTGTCGAGAAGAGGAGAACAATATCTTTCATGTTGTTCTCCT<br>CTCCTCGACACCCAGTAGTTACAACATGCTGTAAAGGCACTC<br>GGTCACCAGTCAGAAAAAACCCTCCATCAGAGTCCAAAAAGG<br>CAGCGGTCGCCAGCGGG	S2
**pTS2531**	NUP43_V233V_3x20-20p8_RG-V25_CA&GU	GGTGTCGAGAAGAGGAGAACAATATCTTTCATGTTGTTCTCCT<br>CTCCTCGACACCCAGTAGCcGCAACATGCTGTAAAGGCACTC<br>GGTCACCAGTCAGAAAAAACCCTCCATCAGAGTCCAAAAAGG<br>CAGCGGTCGCCAGCGGG	S2
**pTS1702**	GUSB_L456L_3x20-20p8_RG-V25_CA-only	GGTGTCGAGAAGAGGAGAACAATATCTTTCATGTTGTTCTCCT<br>CTCCTCGACACCCAGATTCCAGGTGGGACGCAAAAGCCACA<br>GACCACATCACGACAAACACGCCGGGACACTCATCGAAAAGC<br>ACCAAGCCAGCGAAGCAG	S2
**pTS1770**	GUSB_L456L_3x20-20p8_RG-V25_UG&CA	GGTGTCGAGAAGAGGAGAACAATATCTTTCATGTTGTTCTCCT<br>CTCCTCGACACCCAGATTTCAGGTGGGACGCAAAAGCCACAG<br>ACCACATCACGACAAACACGCCGGGACACTCATCGAAAAGCA<br>CCAAGCCAGCGAAGCAG	S2
**pTS1769**	GUSB_L456L_3x20-20p8_RG-V25_UG-only	GGTGTCGAGAAGAGGAGAACAATATCTTTCATGTTGTTCTCCT<br>CTCCTCGACACCCAGATTTTAGGTGGGACGCAAAAGCCACAG<br>ACCACATCACGACAAACACGCCGGGACACTCATCGAAAAGCA<br>CCAAGCCAGCGAAGCAG	S2
**pTS2532**	GUSB_L456L_3x20-20p8_RG-V25_CA&GU	GGTGTCGAGAAGAGGAGAACAATATCTTTCATGTTGTTCTCCT<br>CTCCTCGACACCCAGATTCcGGGTGGGACGCAAAAGCCACAG<br>ACCACATCACGACAAACACGCCGGGACACTCATCGAAAAGCA<br>CCAAGCCAGCGAAGCAG	S2
**pTS1705**	ACTB_3'-UTR_3x20-20p8_RG-V25_CA-only	GGTGTCGAGAAGAGGAGAACAATATCTTTCATGTTGTTCTCCT<br>CTCCTCGACACCACGCAACCAAGTCATAGTCCAAAAGCCGCC<br>GATCCACACGGAGAAACCTCAGGGCAGCGGAACCGCAAAGC<br>TCGAAGTCCAGGGCGACG	S2
**pTS1774**	ACTB_3'-UTR_3x20-20p8_RG-V25_UG&CA	GGTGTCGAGAAGAGGAGAACAATATCTTTCATGTTGTTCTCCT<br>CTCCTCGACACCACGCAATCAAGTCATAGTCCAAAAGCCGCC<br>GATCCACACGGAGAAACCTCAGGGCAGCGGAACCGCAAAGC<br>TCGAAGTCCAGGGCGACG	S2
**pTS1773**	ACTB_3'-UTR_3x20-20p8_RG-V25_UG-only	GGTGTCGAGAAGAGGAGAACAATATCTTTCATGTTGTTCTCCT<br>CTCCTCGACACCACGCAATTAAGTCATAGTCCAAAAGCCGCC<br>GATCCACACGGAGAAACCTCAGGGCAGCGGAACCGCAAAGC<br>TCGAAGTCCAGGGCGACG	S2
**pTS2533**	ACTB_3'-UTR_3x20-20p8_RG-V25_CA&GU	GGTGTCGAGAAGAGGAGAACAATATCTTTCATGTTGTTCTCCT<br>CTCCTCGACACCACGCAACcGAGTCATAGTCCAAAAGCCGCC<br>GATCCACACGGAGAAACCTCAGGGCAGCGGAACCGCAAAGC<br>TCGAAGTCCAGGGCGACG	S2
**pTS2087**	AHI1_K706_2xBoxB_10(3)-29p15-(3)10_5'-_and_3' NN_reference	ACTAGCTCTCGGCCCTGAAAAAGGGCCCAGCTGGATGGAATC<br>TAGCCGTGTAAACAGGCCCTGAAAAAGGGCCGAAGGATGAG	EDF3, S5
**pTS2111**	AHI1_K706_2xBoxB_10(3)-29p15-(3)10_5'-_and_3' NN_GA-mismatch	ACTAGCTCTCGGCCCTGAAAAAGGGCCCAGCTGGATGGAAG<br>CGAGCCGTGTAAACAGGCCCTGAAAAAGGGCCGAAGGATGA<br>G	EDF3, S5
**pTS2117**	AHI1_K706_2xBoxB_10(3)-29p15-(3)10_5'-_and_3' NN_GU-wobble	ACTAGCTCTCGGCCCTGAAAAAGGGCCCAGCTGGATGGAGT<br>CTGGCCGTGTAAACAGGCCCTGAAAAAGGGCCGAAGGATGA<br>G	EDF3, S5
**pTS2088**	BMPR2_K984_2xBoxB_10(3)-29p15-(3)10_5' NN_reference	ATGACCCAGGGGCCCTGAAAAAGGGCCAGGGGCGCCACCG<br>CCTAAGAGAATAGGGAGGCCCTGAAAAAGGGCCTTCACACGT<br>T	EDF3, S5
**pTS2114**	BMPR2_K984_2xBoxB_10(3)-29p15-(3)10_5' NN_GA-mismatch	ATGACCCAGGGGCCCTGAAAAAGGGCCAGGGGCGCCACCG<br>CCGAAGAGAATAGGGAGGCCCTGAAAAAGGGCCTTCACACG<br>TT	EDF3, S5
**pTS2120**	BMPR2_K984_2xBoxB_10(3)-29p15-(3)10_5' NN_ GU-wobble	ATGACCCAGGGGCCCTGAAAAAGGGCCAGGGGCGCCACCG<br>CCTGAGAGAATAGGGAGGCCCTGAAAAAGGGCCTTCACACG<br>TT	EDF3, S5
**pTS2086**	AHI1_I1179_2xBoxB_10(3)-29p15-(3)10_3' NN_reference	GCTTGTTCTTCGGCCCTGAAAAAGGGCCATCCGTGTATCCAT<br>CATGTGTCCTTGGTCGGCCCTGAAAAAGGGCCATGGCTCTGT	EDF3, S5

**pTS2110**	AHI1_I1179_2xBoxB_10(3)-29p15-(3)10_3'NN_GA-mismatch	GCTTGTTCTTCGGCCCTGAAAAAGGGCCATCCGTGTATCCAG<br>CATGTGTCCTTGGTCGGCCCTGAAAAAGGGCCATGGCTCTGT	EDF3, S5
**pTS2116**	AHI1_I1179_2xBoxB_10(3)-29p15-(3)10_3'NN_GU-wobble	GCTTGTTCTTCGGCCCTGAAAAAGGGCCATCCGTGTATCCGT<br>CATGTGTCCTTGGTCGGCCCTGAAAAAGGGCCATGGCTCTGT	EDF3, S5
**pTS2089**	BMPR2_N1005_2xBoxB_10(3)-29p15-(3)10_3'NN_reference	GTGGAATGAACGGCCCTGAAAAAGGGCCCCTGTTACTGCCAT<br>CATTGTTGACTTCACGGCCCTGAAAAAGGGCCCCAGCGATTC	EDF3, S5
**pTS2112**	BMPR2_N1005_2xBoxB_10(3)-29p15-(3)10_3'NN_GA-mismatch	GTGGAATGAACGGCCCTGAAAAAGGGCCCCTGTTACTGCCA<br>GCATTGTTGACTTCACGGCCCTGAAAAAGGGCCCCAGCGATT<br>C	EDF3, S5
**pTS2118**	BMPR2_N1005_2xBoxB_10(3)-29p15-(3)10_3'NN_GU-wobble	GTGGAATGAACGGCCCTGAAAAAGGGCCCCTGTTACTGCCGT<br>CATTGTTGACTTCACGGCCCTGAAAAAGGGCCCCAGCGATTC	EDF3, S5
**pTS2091**	COL3A1_N1244_2xBoxB_10(3)-29p15-(3)10_3'NN_reference	GACTAATGAGGGCCCTGAAAAAGGGCCTTCTATTTGTCCATC<br>AACAGACTTGAGTGGGCCCTGAAAAAGGGCCTCATAATCTC	EDF3, S5
**pTS2113**	COL3A1_N1244_2xBoxB_10(3)-29p15-(3)10_3'NN_GA-mismatch	GACTAATGAGGGCCCTGAAAAAGGGCCTTCTATTTGTCCAGC<br>AACAGACTTGAGTGGGCCCTGAAAAAGGGCCTCATAATCTC	EDF3, S5
**pTS2119**	COL3A1_N1244_2xBoxB_10(3)-29p15-(3)10_3'NN_GU-wobble	GACTAATGAGGGCCCTGAAAAAGGGCCTTCTATTTGTCCGTC<br>AACAGACTTGAGTGGGCCCTGAAAAAGGGCCTCATAATCTC	EDF3, S5
**ASO V117.24**	PEX1 G843D_A:C+G:G	mU*mG*mA*G*U*A*U*C*U*G*C*C*U*A*A*C*U*U*C*A*U*G*U*A*<br>A*CCCACGAAU*C*U*U*G*U*C*C*C*A*A*C*C*C*A*G*G*U*C*U*<br>C*U*A*mG*mG*mU	3
**ASO V117.27**	PEX1 G843D_A:C+G:G_off-trgt_OMe	mU*mG*mA*G*U*A*U*C*U*G*C*C*U*A*A*C*U*U*C*A*U*mG*mU<br>*mA*A*CCCACGAAU*mC*mU*mU*G*U*C*C*C*A*A*C*C*C*A*G*<br>G*U*C*U*mC*mU*mA*mG*mG*mU	3
**ASO V117.28**	PEX1 G843D_A:C+G:G_off-trgt_G:U wobble	mU*mG*mA*G*U*A*U*C*U*G*C*C*U*A*A*C*U*U*C*A*U*G*U*G*<br>A*CCCACGAAU*mC*mU*mU*G*U*C*C*C*A*A*C*C*C*A*G*G*U*<br>C*U*C*U*G*mG*mG*mU	3
**pTS1862**	3_0 20p8-3x20 Luci circ + RG U6	AACCATGCCGACTGATGGCAGAAAAAGGTGTCGAGAAGAGG<br>AGAACAATATGCTAAATGTTGTTCTCGTCTCCTCGACACCGTG<br>CAGCCAGCCGTCCTTGTAAACGTAGCCGCTCATGATCATGAA<br>ACACGGACGCACAGCTCGCCGAAACCGGTGTCCAAGTCCAC<br>CACAAAAACTGCCATCAGTCGGCGTGGACTGTAG	S9
**pTS1863**	2_1 20p8-3x20 Luci circ + RG U6	AACCATGCCGACTGATGGCAGAAAAAGGTGTCGAGAAGAGG<br>AGAACAATATGCTAAATGTTGTTCTCGTCTCCTCGACACCGTG<br>CAGCCAGCCGTCCTTGTAAACGTAGCCGCTCATGATCATGAA<br>ACACGGACGCACAGCTCGCCGAAAGTGCTCGTCCTCGTCCC<br>AGTAAAAACTGCCATCAGTCGGCGTGGACTGTAG	S9-10
**pTS1864**	1_2 20p8-3x20 Luci circ + RG U6	AACCATGCCGACTGATGGCAGAAAAAGGTGTCGAGAAGAGG<br>AGAACAATATGCTAAATGTTGTTCTCGTCTCCTCGACACCGTG<br>CAGCCAGCCGTCCTTGTAAACGTAGCCGCTCATGATCATGAA<br>AACCCCGGCGTCGAAGATGTTAAAGTGCTCGTCCTCGTCCCA<br>GTAAAAACTGCCATCAGTCGGCGTGGACTGTAG	S9
**pTS1865**	0_3 20p8-3x20 Luci circ + RG U6	AACCATGCCGACTGATGGCAGAAAAAGGTGTCGAGAAGAGG<br>AGAACAATATGCTAAATGTTGTTCTCGTCTCCTCGACACCGTG<br>CAGCCAGCCGTCCTTGTAAAAGCTCGCCGGCATCGTCGTCAA<br>ACCCCGGCGTCGAAGATGTTAAAGTGCTCGTCCTCGTCCCAG<br>TAAAAACTGCCATCAGTCGGCGTGGACTGTAG	S9
**pTS1867**	3_0 20p8-3x20 Luci circ + RG EF1α	AACCATGCCGACTGATGGCAGAAAAAGGTGTCGAGAAGAGG<br>AGAACAATATGCTAAATGTTGTTCTCGTCTCCTCGACACCGTG<br>CAGCCAGCCGTCCTTGTAAACGTAGCCGCTCATGATCATGAA<br>ACACGGACGCACAGCTCGCCGAAACCGGTGTCCAAGTCCAC<br>CACAAAAACTGCCATCAGTCGGCGTGGACTGTAG	S9
**pTS1868**	2_1 20p8-3x20 Luci circ + RG EF1α	AACCATGCCGACTGATGGCAGAAAAAGGTGTCGAGAAGAGG<br>AGAACAATATGCTAAATGTTGTTCTCGTCTCCTCGACACCGTG<br>CAGCCAGCCGTCCTTGTAAACGTAGCCGCTCATGATCATGAA<br>ACACGGACGCACAGCTCGCCGAAAGTGCTCGTCCTCGTCCC<br>AGTAAAAACTGCCATCAGTCGGCGTGGACTGTAG	S9-10
**pTS1869**	1_2 20p8-3x20 Luci circ + RG EF1α	AACCATGCCGACTGATGGCAGAAAAAGGTGTCGAGAAGAGG<br>AGAACAATATGCTAAATGTTGTTCTCGTCTCCTCGACACCGTG<br>CAGCCAGCCGTCCTTGTAAACGTAGCCGCTCATGATCATGAA<br>AACCCCGGCGTCGAAGATGTTAAAGTGCTCGTCCTCGTCCCA<br>GTAAAAACTGCCATCAGTCGGCGTGGACTGTAG	S9

pTS1870	0_3 20p8-3x20 Luci circ + RG EF1α	AACCATGCCGACTGATGGCAGAAAAAGGTGTCGAGAAGAGGAGAACAATATGCTAAATGTTGTTCTCGTCTCCTCGACACCGTGCAGCCAGCCGTCCTTGTAAAAGCTCGCCGGCATCGTCGTCAAACCCCGGCGTCGAAGATGTTAAAGTGCTCGTCCTCGTCCCAGTAAAAACTGCCATCAGTCGGCGTGGACTGTAG	S9
pTS1872	Z-20p8-20-20-20 Luci circ U6	AACCATGCCGACTGATGGCAGAAAAAGTGCAGCCAGCCGTCCTTGTAAACGTAGCCGCTCATGATCATGAAACACGGACGCACAGCTCGCCGAAACCGGTGTCCAAGTCCACCACAAAAACTGCCATCAGTCGGCGTGGACTGTAG	S9
pTS1873	Z-20-20p8-20-20 Luci circ U6	AACCATGCCGACTGATGGCAGAAAAACCGGTGTCCAAGTCCACCACAAAGTGCAGCCAGCCGTCCTTGTAAACGTAGCCGCTCATGATCATGAAACACGGACGCACAGCTCGCCGCTGCCATCAGTCGGCGTGGACTGTAG	S9
pTS1874	Z-20-20-20p8-20 Luci circ U6	AACCATGCCGACTGATGGCAGAAAAACACGGACGCACAGCTCGCCGAAACCGGTGTCCAAGTCCACCACAAAGTGCAGCCAGCCGTCCTTGTAAACGTAGCCGCTCATGATCATGAAAAACTGCCATCAGTCGGCGTGGACTGTAG	S9
pTS1875	Z-20-20-20-20p8 Luci circ U6	AACCATGCCGACTGATGGCAGAAAAACGTAGCCGCTCATGATCATGAAACACGGACGCACAGCTCGCCGAAACCGGTGTCCAAGTCCACCACAAAGTGCAGCCAGCCGTCCTTGTAAAAACTGCCATCAGTCGGCGTGGACTGTAG	S9
pTS1877	Z-20p8-20-20-20 Luci circ EF1α	AACCATGCCGACTGATGGCAGAAAAAGTGCAGCCAGCCGTCCTTGTAAACGTAGCCGCTCATGATCATGAAACACGGACGCACAGCTCGCCGAAACCGGTGTCCAAGTCCACCACAAAAACTGCCATCAGTCGGCGTGGACTGTAG	S9
pTS1878	Z-20-20p8-20-20 Luci circ EF1α	AACCATGCCGACTGATGGCAGAAAAACCGGTGTCCAAGTCCACCACAAAGTGCAGCCAGCCGTCCTTGTAAACGTAGCCGCTCATGATCATGAAACACGGACGCACAGCTCGCCGCTGCCATCAGTCGGCGTGGACTGTAG	S9
pTS1879	Z-20-20-20p8-20 Luci circ EF1α	AACCATGCCGACTGATGGCAGAAAAACACGGACGCACAGCTCGCCGAAACCGGTGTCCAAGTCCACCACAAAGTGCAGCCAGCCGTCCTTGTAAACGTAGCCGCTCATGATCATGAAAAACTGCCATCAGTCGGCGTGGACTGTAG	S9
pTS1880	Z-20-20-20-20p8 Luci circ EF1α	AACCATGCCGACTGATGGCAGAAAAACGTAGCCGCTCATGATCATGAAACACGGACGCACAGCTCGCCGAAACCGGTGTCCAAGTCCACCACAAAGTGCAGCCAGCCGTCCTTGTAAAAACTGCCATCAGTCGGCGTGGACTGTAG	S9
pTS1931	2-1-circ_3x_RC_20-20_20p8-RG-20_Luci_gRNA EF1α	AACCATGCCGACTGATGGCAGAAAAAGTGCTCGTCCTCGTCCCAGTAAAGGTGTCGAGAAGAGGAGAACAATATGCTAAATGTTGTTCTCGTCTCCTCGACACCGTGCAGCCAGCCGTCCTTGTAAACGTAGCCGCTCATGATCATGAAACACGGACGCACAGCTCGCCGAAAAACTGCCATCAGTCGGCGTGGACTGTAG	S10
pTS1932	2-1-circ_3x_RC_20_20p8-RG-20-20_Luci_gRNA EF1α	AACCATGCCGACTGATGGCAGAAAAACACGGACGCACAGCTCGCCGAAAGTGCTCGTCCTCGTCCCAGTAAAGGTGTCGAGAAGAGGAGAACAATATGCTAAATGTTGTTCTCGTCTCCTCGACACCGTGCAGCCAGCCGTCCTTGTAAACGTAGCCGCTCATGATCATGAAAAACTGCCATCAGTCGGCGTGGACTGTAG	S10
pTS1933	2-1-circ_3x_RC_20p8-RG-20-20_Luci_gRNA EF1α	AACCATGCCGACTGATGGCAGAAAAACGTAGCCGCTCATGATCATGAAACACGGACGCACAGCTCGCCGAAAGTGCTCGTCCTCGTCCCAGTAAAGGTGTCGAGAAGAGGAGAACAATATGCTAAATGTTGTTCTCGTCTCCTCGACACCGTGCAGCCAGCCGTCCTTGTAAAAACTGCCATCAGTCGGCGTGGACTGTAG	S10
pTS1938	2-1-circ_3x_RC_20-20_20p8-RG-20_Luci_gRNA U6	AACCATGCCGACTGATGGCAGAAAAAGTGCTCGTCCTCGTCCCAGTAAAGGTGTCGAGAAGAGGAGAACAATATGCTAAATGTTGTTCTCGTCTCCTCGACACCGTGCAGCCAGCCGTCCTTGTAAACGTAGCCGCTCATGATCATGAAACACGGACGCACAGCTCGCCGAAAAACTGCCATCAGTCGGCGTGGACTGTAG	S10
pTS1939	2-1-circ_3x_RC_20_20p8-RG-20-20_Luci_gRNA U6	AACCATGCCGACTGATGGCAGAAAAACACGGACGCACAGCTCGCCGAAAGTGCTCGTCCTCGTCCCAGTAAAGGTGTCGAGAAGAGGAGAACAATATGCTAAATGTTGTTCTCGTCTCCTCGACACCGTGCAGCCAGCCGTCCTTGTAAACGTAGCCGCTCATGATCATGAAAAACTGCCATCAGTCGGCGTGGACTGTAG	S10
pTS1940	2-1-circ_3x_RC_20p8-RG-20-20_Luci_gRNA U6	AACCATGCCGACTGATGGCAGAAAAACGTAGCCGCTCATGATCATGAAACACGGACGCACAGCTCGCCGAAAGTGCTCGTCCTCGTCCCAGTAAAGGTGTCGAGAAGAGGAGAACAATATGCTAAATGTTGTTCTCGTCTCCTCGACACCGTGCAGCCAGCCGTCCTTGTAAAAACTGCCATCAGTCGGCGTGGACTGTAG	S10

**pTS1934**	2-1-circ_3x_RC_split-RG(V2) Luci_gRNA EF1α	AACCATGCCGACTGATGGCAGAATGTTGTTCTCGTCTCCTCG<br>ACACCGTGCAGCCAGCCGTCCTTGTAAACGTAGCCGCTCATG<br>ATCATGAAACACGGACGCACAGCTCGCCGAAAGTGCTCGTCC<br>TCGTCCCAGTAAAAAGGTGTCGAGAAGAGGAGAACAATATGC<br>TACTGCCATCAGTCGGCGTGGACTGTAG	S10
**pTS1941**	2-1-circ_3x_RC_split-RG(V2)_Luci_gRNA U6	AACCATGCCGACTGATGGCAGAATGTTGTTCTCGTCTCCTCG<br>ACACCGTGCAGCCAGCCGTCCTTGTAAACGTAGCCGCTCATG<br>ATCATGAAACACGGACGCACAGCTCGCCGAAAGTGCTCGTCC<br>TCGTCCCAGTAAAAAGGTGTCGAGAAGAGGAGAACAATATGC<br>TACTGCCATCAGTCGGCGTGGACTGTAG	S10
**pTS1989**	2-1-circ_3x_RC_split-RG(V1) Luci_gRNA EF1α	AACCATGCCGACTGATGGCAGATGTTGTTCTCGTCTCCTCGA<br>CACCGTGCAGCCAGCCGTCCTTGTAAACGTAGCCGCTCATGA<br>TCATGAAACACGGACGCACAGCTCGCCGAAAGTGCTCGTCCT<br>CGTCCCAGTAAAAAGGTGTCGAGAAGAGGAGAACAATATCTG<br>CCATCAGTCGGCGTGGACTGTAG	S10
**pTS1992**	2-1-circ_3x_RC_split-RG(V1)_Luci_gRNA U6	AACCATGCCGACTGATGGCAGATGTTGTTCTCGTCTCCTCGA<br>CACCGTGCAGCCAGCCGTCCTTGTAAACGTAGCCGCTCATGA<br>TCATGAAACACGGACGCACAGCTCGCCGAAAGTGCTCGTCCT<br>CGTCCCAGTAAAAAGGTGTCGAGAAGAGGAGAACAATATCTG<br>CCATCAGTCGGCGTGGACTGTAG	S10
**pTS1594**	NUP43 V233V 111 nt Circular EF1α	AACCATGCCGACTGATGGCAGTAGTACCTTGTCTAACATCCC<br>AAATACTCAACATTCCATCTTGGCCACCAGTAGCCACAACATG<br>CTGTTGGTTGGGATGTCTATCAACACAGTGGAGTGGCACTCG<br>GTCACCTGCCATCAGTCGGCGTGGACTGTAG	S6
**pTS1722**	NUP43 V233V 111 nt Linear EF1α	TAGTACCTTGTCTAACATCCCAAATACTCAACATTCCATCTTG<br>GCCACCAGTAGCCACAACATGCTGTTGGTTGGGATGTCTATC<br>AACACAGTGGAGTGGCACTCGGTCAC	S6
**pTS1643**	RAB7A 3'UTR 111 nt Circular EF1α	AACCATGCCGACTGATGGCAGGTCTTTGATAAAAGGCGTACA<br>TAATTCTTGTGTCTACTGTACAGAATACTGCCGCCAGCTGGAT<br>TTCCCAATTCTGAGTAACACTCTGCAATCCAAACAGGGTTCAA<br>CCCTCTGCCATCAGTCGGCGTGGACTGTAG	S6-8
**pTS1724**	RAB7A 3'UTR 111 nt Linear EF1α	GTCTTTGATAAAAGGCGTACATAATTCTTGTGTCTACTGTACA<br>GAATACTGCCGCCAGCTGGATTTCCCAATTCTGAGTAACACT<br>CTGCAATCCAAACAGGGTTCAACCCT	S6-7
**pTS1810**	NUP43 V233V 111 nt Circular U6	AACCATGCCGACTGATGGCAGTAGTACCTTGTCTAACATCCC<br>AAATACTCAACATTCCATCTTGGCCACCAGTAGCCACAACATG<br>CTGTTGGTTGGGATGTCTATCAACACAGTGGAGTGGCACTCG<br>GTCACCTGCCATCAGTCGGCGTGGACTGTAG	S6
**pTS1811**	NUP43 V233V LEAPER U6	TAGTACCTTGTCTAACATCCCAAATACTCAACATTCCATCTTG<br>GCCACCAGTAGCCACAACATGCTGTTGGTTGGGATGTCTATC<br>AACACAGTGGAGTGGCACTCGGTCAC	S6
**pTS1812**	RAB7A 3'UTR 111 nt Circular U6	AACCATGCCGACTGATGGCAGGTCTTTGATAAAAGGCGTACA<br>TAATTCTTGTGTCTACTGTACAGAATACTGCCGCCAGCTGGAT<br>TTCCCAATTCTGAGTAACACTCTGCAATCCAAACAGGGTTCAA<br>CCCTCTGCCATCAGTCGGCGTGGACTGTAG	S6-8
**pTS1813**	RAB7A 3'UTR LEAPER U6	GTCTTTGATAAAAGGCGTACATAATTCTTGTGTCTACTGTACA<br>GAATACTGCCGCCAGCTGGATTTCCCAATTCTGAGTAACACT<br>CTGCAATCCAAACAGGGTTCAACCCT	S6-7
**pTS1896**	RAB7A 3'UTR 111 nt Circular U6 stable integration	AACCATGCCGACTGATGGCAGGTCTTTGATAAAAGGCGTACA<br>TAATTCTTGTGTCTACTGTACAGAATACTGCCGCCAGCTGGAT<br>TTCCCAATTCTGAGTAACACTCTGCAATCCAAACAGGGTTCAA<br>CCCTCTGCCATCAGTCGGCGTGGACTGTAG	S8
**pTS1897**	RAB7A 3'UTR 111 nt Linear U6 stable integration	GTCTTTGATAAAAGGCGTACATAATTCTTGTGTCTACTGTACA<br>GAATACTGCCGCCAGCTGGATTTCCCAATTCTGAGTAACACT<br>CTGCAATCCAAACAGGGTTCAACCCT	S8
**pTS1899**	RAB7A 3'UTR 111 nt Circular EF1α stable integration	AACCATGCCGACTGATGGCAGGTCTTTGATAAAAGGCGTACA<br>TAATTCTTGTGTCTACTGTACAGAATACTGCCGCCAGCTGGAT<br>TTCCCAATTCTGAGTAACACTCTGCAATCCAAACAGGGTTCAA<br>CCCTCTGCCATCAGTCGGCGTGGACTGTAG	S8
**pTS1900**	RAB7A 3'UTR 111 nt Linear EF1α stable integration	GTCTTTGATAAAAGGCGTACATAATTCTTGTGTCTACTGTACA<br>GAATACTGCCGCCAGCTGGATTTCCCAATTCTGAGTAACACT<br>CTGCAATCCAAACAGGGTTCAACCCT	S8
**pTS2515**	NUP43_UTR_CircLEAPER_151p76_AC50	AACCATGCCGACTGATGGCAGAAAAACAAAAAACAAAAAAAA<br>CAAAAAAAAAACCAAAAAAAACAAAACACAAGAGCAAAACTCCA<br>TCTCAAAAAATATATATATTTATTAGGACAGAAAAGCAAAACCT<br>TACCATAATTCCATAACCATGATTATTGGAATTACAAAGTAAAT<br>GAACACTTCAATTGTCTAATAGCAGTGATTTGTCATTAAGGTC<br>TCAGGTAAAAACAAAAAACAAAAAAAACAAAAAAAAAAACCAAA<br>AAAACAAAACACACTGCCATCAGTCGGCGTGGACTGTAG	EDF2

**pTS2560**	NUP43_UTR_CircLEAPER_151p76_AC50_GU_at_GU-amenable-sites	AACCATGCCGACTGATGGCAGAAAAACAAAAAACAAAAAAAA<br>CAAAAAAAAAACCAAAAAAAACAAAACACAAGAGCAAAACTCCA<br>TCTCAAAAAgTATATATgTTTgTTAGGACAGAAAAGCAAAACCT<br>TgCCATggTTCCgTAACCATGATTgTTGGAATTgCAAAGTgAATG<br>AACACTTCAgTTGTCTgATgGCAGTGATTTGTCgTTgAGGTCTC<br>AGGTAAAAACAAAAAACAAAAAAAACAAAAAAAAAACCAAAAA<br>AACAAAACACACTGCCATCAGTCGGCGTGGACTGTAG	EDF2
**pTS2561**	NUP43_UTR_CircLEAPER_151p76_AC50_GA_at_GU-amenable-sites	AACCATGCCGACTGATGGCAGAAAAACAAAAAACAAAAAAAA<br>CAAAAAAAAAACCAAAAAAAACAAAACACAAGAGCAAAACTCCA<br>TCTCAAAAAAgATATAgATTgATTAGGACAGAAAAGCAAAACCT<br>gACCAgAAgTCCAgAACCATGATgATTGGAATgACAAAGgAAAT<br>GAACACTTCAAgTGTCgAAgAGCAGTGATTTGTCAggAAGGTCT<br>CAGGTAAAAACAAAAAACAAAAAAAACAAAAAAAAAACCAAAA<br>AAACAAAACACACTGCCATCAGTCGGCGTGGACTGTAG	EDF2
**pTS2562**	NUP43_UTR_CircLEAPER_151p76_AC50_GA_at_all_off-target_sites	AACCATGCCGACTGATGGCAGAAAAACAAAAAACAAAAAAAA<br>CAAAAAAAAAACCAAAAAAAACAAAACACAAGAGCAAAACTCCA<br>TCTCAAAAAAgATATAgATggATTAGGACAGAAAAGCAAAACCg<br>gACCAgAAgTCCAgAACCATGATgATTGGAATgACAAAGgAAAT<br>GAACACgTCAAgTGTCgAAgAGCAGTGATgTGTCAggAAGGTCT<br>CAGGTAAAAACAAAAAACAAAAAAAACAAAAAAAAAACCAAAA<br>AAACAAAACACACTGCCATCAGTCGGCGTGGACTGTAG	EDF2
**pTS2563**	NUP43_UTR_CircLEAPER_151p76_AC50_GU+GA	AACCATGCCGACTGATGGCAGAAAAACAAAAAACAAAAAAAA<br>CAAAAAAAAAACCAAAAAAAACAAAACACAAGAGCAAAACTCCA<br>TCTCAAAAAgTATATATgTgTgTTAGGACAGAAAAGCAAAACCgT<br>gCCATggTTCCgTAACCATGATTgTTGGAATTgCAAAGTgAATGA<br>ACACgTCAgTTGTCTgATgGCAGTGATgTGTCgTTgAGGTCTCA<br>GGTAAAAACAAAAAACAAAAAAAACAAAAAAAAAACCAAAAAA<br>ACAAAACACACTGCCATCAGTCGGCGTGGACTGTAG	EDF2

Supplementary Table S2: Primer list

Primer #	Primer Name	Sequence 5‘->3‘
	+91 Mecp2 E1 Rev	ggaagctttgtcagagccctacc
	-14 Mecp2 E1 Fwd	aacccgtccggaaaatggcc
	Mecp2 E1 743 Rev	ccaggcgatgcttggaaagg
	Mecp2G311A Fwd (genotyping)	cccaccttgcctgaagggta
	Mecp2G311A Rev (genotyping)	cctagccttcctacccacctg
	Sex Fwd	accttaagaacaagccaataca
	Sex Rev	ggcttgtcctgaaaacatttgg
	TM766 Rev	tgtacaagaaagctgggtcg
	TM764 Fwd	ctttgcgaagtgtcaacct
	TM768 Rev	ttccaggcggaccatacaac
	TM764 Fwd	ctttgcgaagtgtcaacct
	AAV2_ITR_fw	ggaacccctagtgatggagtt
	AAV2_ITR_bw	cggcctcagtgagcga
121	GFP_fw	gcggatccaccatggctagcaaaggagaagaactc
144	BGH_bw	ctagaaggcacagtcgaggc
551	BGH_long_bw	gctggcaactagaaggcacagtcgaggc
566	GFP_seq_fw	gacacgtgctgaagtcaagtttgaaggtg
1032	ACTB_fw	cagcagatgtggatcagcaagcaggag
1033	ACTB_bw	ggaaggggggcacgaaggctcatc
2443	BsaI_SV40_PolyA_bw	tatggtctctggcctaagatacattgatgagtttggacaaac
2901	AHI1_W725Amber_fw	gccgtatcccaaacaaacacc
2902	AHI1_W725Amber_bw	ccaaacaacaatcacccctgt
2903	BMPR2_W298Amber_fw	ccttggatgagcgtccagtt
2905	COL3A1_W1278Amber_fw	tgctgggattggaggtgaaaa
2919	GUSB_L456L_fw	caacaagcatgaggatgcgg
2920	GUSB_L456L_bw	gtgcccgtagtcgtgatacc
2933	NUP43_V233V_fw	tgtgtgcaacaacccagaaat
2934	NUP43_V233V_bw	gtactgcttcttcctccttggtg
2939	RAB7A_3'UTR_fw	gccccattacaggctcacac
2940	RAB7A_3'UTR_bw	ttgaagtgtggagcaggggg
3041	RAB7A_Exon5_fw	ccagacgattgcacggaatg
3107	Murine_Adar1_fw	tcccgccattaccctgtctt
3108	Murine_Adar1_bw	catggtacggagtctttcccc
3109	Murine_Adar1p150_fw	cttgccggcactatgtctca
3110	Murine_Adar1p150_bw	ctgcgggtatctccacttgc
3134	RAB7A_3'UTR_Sense-Oligo	ggttgaaccctgtttggattgcagagtgttactcagaattgggaaatccagctggcggcagtattct gtacagtagacacaagaattatgtacgccttttatcaaagtga
3236	Murine_Actb fw	gagcgcaagtactctgtgtg
3237	Murine_Actb_bw	aaacgcagctcagtaacagtc
3241	mMecp2_fw	aaccttcagcccaccattct
3243	GUSB_L456L_Meta-Sense-Oligo	gggcttcgactggccgctgctggtgtttttcctgcttcgctggcttggtgcctttttcgatgagtgtccc ggcgtgggcctggcgctgccgctttttccccgcggtcgtgatgtggtctgtggcctttttgcgtccca cctcgaatgcc
3244	NUP43_V233V_Meta-Sense-Oligo	gttggagaggatggtcgatttttctcctgagattcttttttctgactggtgaccgagtgccttttacagc atgttgtcgctagcc

3454	NUP43_5'UTR_fw	ctgctgcggccgctttcg
3569	ACTB_3'UTR_Sense-Oligo	cgtcgccctggacttcgagctttgcggttccgctgccctgaggtttctccgtgtggatcggcggctttt ggactatgacttcgttggca
4242	Murine_Rps29_fw	tgaaggcaagatgggtcac
4243	Murine_Rps29_bw	gcacatgttcagcccgtatt
4247	Murine_Adar2_fw	ttgccctgaaggagttttg
4248	Murine_Adar2_bw	gagggcttcttgactggc
4964	mMecp2_65°C_bw	acaacaagtttcccagggctcttctcc
4965	mMecp2_65°C_fw	ctagcgctaccggactcagatctcg
5058	MluI_U6_fw	gggcacgcgtgagggcctatttcccatgattcc
5059	BglII_BamHI_KpnI_SV40_bw	aaaagatctggatccaaaggtacctaagatacattgatgagtttggacaaacc
5062	KpnI_Circ-guideRNA_fw	aaaggtaccaccgtgctcgcttcggcagc
5122	MluI_Universal_Stuffer_fw	agaacgcgtgctaccatggaccatcctg
5123	MluI_2827nt_Stuffer_bw	aaaacgcgtgtatactgctgccatccag
5261	V27.2.4_inwards_qPCR_fw	cataggtccccaaacacgga
5262	V27.2.4_inwards_qPCR_bw	ggccgatctgctggatttagt
5265	V27.2.4_outwards_qPCR_fw	ggagcgccgcaaagatcaa
5266	V27.2.4_outwards_qPCR_bw	tttttgcctgaaggttggacac
6109	NUP43_3‘ UTR_fw	agctgaaagttgccaaactgg
6110	NUP43_3‘ UTR_bw	cctgcaacccttgaaagacac
6198	Murine_Rnu6_fw	gcttcggcagcacatatactaaaat
6199	Murine_Rnu6_bw	cgcttcacgaatttgcgtgtcat

Supplementary Table S3: Primer pair list

Target	Primer Pair	Sense Oligo	Sequencing Primer
**ACTB 3'UTR**	1032+1033	3569	1032
**AHI1 K706, I1179 & W725Amber**	2901+144	None	2901 (K706),<br>2902 (W725Amber),<br>or 144 (I1179)
**BMPR2 K983 & W298Amber**	2903+144	None	144
**circCLUSTER gRNA V27.2.4 inward (qPCR)**	5261+5262	None	None
**circCLUSTER gRNA V27.2.4 outward (qPCR)**	5265+5266	None	None
**COL3A1 N1244**	2905+144	None	2905
**COL3A1 W1278Amber**	2905+144	None	144
**eGFP cis-acting guide RNA reporter**	551+121	None	566
**GUSB L456L**	2919+2920	3243	2919
**Murine Mecp2 W104Amber (in vitro)**	4964+4965	None	3241
**Murine Mecp2 W104Amber (in vivo, editing)**	-14 Mecp2 E1 Fwd &<br>+91 Mecp2 E1 Rev	None	Mecp2 E1 743 Rev
**Murine Mecp2 W104Amber (in vivo, genotyping)**	Mecp2G311A Fwd &<br>Rev2934+3454	3244	2933
**NUP43 V233V**	2934+3454	3244	2933
**PEX1 G843D set A**	TM766 Rev &<br>TM764 Fwd	None	None
**PEX1 G843D set B (nested PCR)**	TM768 Rev & TM764 Fwd	None	TM768 Rev
**RAB7A 3'UTR**	3041+2940	3134	2939
**Rett mice genotyping (MeCPp2 W104Amber)**	Mecp2G311A Fwd &<br>Mecp2G311A Rev	None	None
**Murine Atcb qPCR**	3236+3237	None	None
**Murine Rnu6 qPCR**	6198+6199	None	None
**Murine Rps29 qPCR**	4242+4243	None	None
**Murine Adar1 qPCR**	3107+3108	None	None
**Murine Adar1p150 qPCR**	3109+3110	None	None
**Murine Adar2 qPCR**	4247+4248	None	None
**NUP43 3' UTR**	6109+6110	None	6109
**AAV ITR**	fw+bw	None	None

Supplementary Table S4: Antibody list

Antibody	Target Protein	Produced in	Immuno-globulin Class	Dilution used	Supplier	Order #	Against	Validation
**MeCP2 (D4F3) XP Rabbit mAb**	α-MeCP2	Rabbit	Monoclonal IgG	1:500	Cell Signaling Technology	3456	Carboxyl end of human MeCP2	Commonly used commercial antibody validated in KO mouse tissue sections previously in our lab. RRID: AB_2143849 PMID: 35939700
**Donkey α Rabbit AlexaFluor 488**	α-rabbit IgG	Donkey	Polyclonal IgG	1:750	Invitrogen	A21206	Whole molecule rabbit IgG	Commonly used commercial antibody with 135 IHC-IF citations as of Jan. 2024. RRID: AB_2535792 Recent publications: PMID: 36865524 PMID: 36321664 PMID: 38017073

Supplementary Notes:

Table of supplementary notes

1. Cloning strategy for cis-acting editing reporters
2. Cloning strategy of circular CLUSTER or LEAPER guide RNAs in the pEdit1.2 backbone
3. Cloning strategy for circular CLUSTER guide RNAs in the pAAV-GFP backbone
4. ORF & amino acid sequences of:
    - Murine Mecp2 W104Amber
    - Human PEX1 G843D
    - dPspCas13b_ADAR2DD_E488Q
    - 4λN_ADAR2DD_E488Q
5. Plasmid-maps & sequences of:
    - pEGFP-N3 vector (Clontech)
    - Tornado OHA vector U6 (pTS1541)
    - pAAV-GFP (Cell Biolabs)
    - circ-mMecp2-V27.2.4_gRNA_pEdit_no-stuffer (pTS2108)
    - circ-mMecp2-V27.2.4_gRNA_pAAV_no-stuffer (pTS2123)
    - BMPR2_W298X_cDNA_pcDNA3.1 (pTS1160)
    - circ-mMecp2-V27.2.4_gRNA_pAAV_stuffer (pTS2154)
    - pcDNA3.1 eGFP (pTS58)
    - Tornado OHA vector EF1α (pTS1593)
    - Tornado OHA vector U6 (pTS1790)
    - Transposon U6 circ RAB7A LEAPER gRNA vector (pTS1896):
    - Transposon U6 linear RAB7A LEAPER gRNA vector (pTS1897):
    - Transposon EF1α circ RAB7A LEAPER gRNA vector (pTS1899):
    - Transposon EF1α linear RAB7A LEAPER gRNA vector (pTS1900):
    - Plasmid-map and sequence of the CircLEAPER AC50 cloning vector (pTS2508):

1. Cloning strategy for cis-acting editing reporters

Two types of cis-acting editing reporters were generated. In both versions the hybrid oligonucleotide inserts were cloned into the 3’ UTR of an eGFP construct (pTS58) via ApaI and AgeI. To make sure that the reporter would not be unfolded due to ribosomal activity an additional Ochre (5’-TAA) stop codon and an additional linker (20 nt in version #1 and 21 nt in version #2) were placed between the primary Amber (5’-TAG) stop codon and the start of the cis-acting guide RNA. The first version is shown in main Figure 1b. In this design, the ADAR recruiting domain and the cis-acting specificity domain are two separate duplexes, separated by a 5x Adenosine linker. In Supplementary Table S1 the cis-acting guide RNAs of design version #1 are displayed from the 5’-end of the R/G motif V21 to the 3’-end of the cis-representation of the target sequence. The second version is shown in Extended Data Figure 3. In this design the ADAR recruiting domain and the cis-acting specificity domain are part of one continuous duplex. In Supplementary Table S1 the cis-acting guide RNAs of design version #2 are displayed from the 5’-end of the cis-representation of the target sequence to the 3’-end of the cis-representation of the specificity domain. In both cases a bGH poly(A) signal serves as termination signal.

Version #1:

1 **ATG**GCTAGCA AAGGAGAAGA ACTCTTCACT GGAGTTGTCC CAATTCTTGT TGAATTAGAT

61 GGTGATGTTA ACGGCCACAA GTTCTCTGTC AGTGGAGAGG GTGAAGGTGA TGCAACATAC

121 GGAAAACTTA CCCTGAAGTT CATCTGCACT ACTGGCAAAC TGCCTGTTCC GTGGCCGACA

181 CTAGTGACGA CGCTCTGCTA TGGCGTCCAG TGCTTTTCAA GATACCCGGA TCACATGAAA

241 CGGCATGACT TTTTCAAGAG TGCCATGCCC GAAGGTTATG TACAGGAAAG GACCATCTTC

301 TTCAAAGATG ACGGCAACTA CAAGACACGT GCTGAAGTCA AGTTTGAAGG TGATACCCTT

361 GTTAATAGAA TCGAGTTAAA AGGTATTGAC TTCAAGGAAG ATGGCAACAT TCTGGGACAC

421 AAATTGGAAT ACAACTATAA CTCACACAAT GTATACATCA TGGCAGACAA ACAAAAGAAT

481 GGAATCAAAG TGAACTTCAA GACCCGCCAC AACATTGAAG ATGGAAGCGT TCAACTAGCA

541 GACCATTATC AACAAAATAC TCCAATTGGC GATGGCCCTG TCCTTTTACC AGACAACCAT

601 TACCTGTCCA CACAATCTGC CCTTTCGAAA GATCCCAACG AAAAGAGAGA CCACATGGTC

661 CTTCTTGAGT TTGTAACAGC TGCTGGGATT ACACATGGCA TGGATGAACT ATACAAATCC

721 GGCTCTAGAG GGCCC**TAGTA A**GTGGAAAGG ACGCGGGATC CGGTGTCGAG AAGAGGAGAA

781 CAATATGCTA AATGTTGTTC TCGTCTCCTC GACACCCAGT AG**NKN**CAACA TGCTGTAAAA

841 AACAGCATGT TG**NAN**CTACT GTTTACCGGT CATCATCACC ATCACCATTG AGTTTAAACC

901 CGCTGATCAG CCTCGACTGT GCCTTCTAGT TGCCAGCCAT CTGTTGTTTG CCCCTCCCCC

961 GTGCCTTCCT TGACCCTGGA AGGTGCCACT CCCACTGTCC TTTCCTAATA AAATGAGGAA

1021 ATTGCATCGC ATTGTCTGAG TAGGTGTCAT TCTATTCTGG GGGGTGGGGT GGGGCAGGAC

1081 AGCAAGGGGG AGGATTGGGA AGACAATAGC AGGCATGCTG GGGATGCGGT GGGCTCTATG

1141 G

Version #2:

1 **ATG**GCTAGCA AAGGAGAAGA ACTCTTCACT GGAGTTGTCC CAATTCTTGT TGAATTAGAT

61 GGTGATGTTA ACGGCCACAA GTTCTCTGTC AGTGGAGAGG GTGAAGGTGA TGCAACATAC

121 GGAAAACTTA CCCTGAAGTT CATCTGCACT ACTGGCAAAC TGCCTGTTCC GTGGCCGACA

181 CTAGTGACGA CGCTCTGCTA TGGCGTCCAG TGCTTTTCAA GATACCCGGA TCACATGAAA

241 CGGCATGACT TTTTCAAGAG TGCCATGCCC GAAGGTTATG TACAGGAAAG GACCATCTTC

301 TTCAAAGATG ACGGCAACTA CAAGACACGT GCTGAAGTCA AGTTTGAAGG TGATACCCTT

361 GTTAATAGAA TCGAGTTAAA AGGTATTGAC TTCAAGGAAG ATGGCAACAT TCTGGGACAC

421 AAATTGGAAT ACAACTATAA CTCACACAAT GTATACATCA TGGCAGACAA ACAAAAGAAT

481 GGAATCAAAG TGAACTTCAA GACCCGCCAC AACATTGAAG ATGGAAGCGT TCAACTAGCA

541 GACCATTATC AACAAAATAC TCCAATTGGC GATGGCCCTG TCCTTTTACC AGACAACCAT

601 TACCTGTCCA CACAATCTGC CCTTTCGAAA GATCCCAACG AAAAGAGAGA CCACATGGTC

661 CTTCTTGAGT TTGTAACAGC TGCTGGGATT ACACATGGCA TGGATGAACT ATACAAATCC

721 GGCTCTAGAG GGCCC**TAGTA** **A**GTGGAAAGG ACGCGGGATC CGCCATATAT WWW**WAR**WWWT

781 CGGGTGTCGA GAAGAGGAGA ACAATATGCT AAATGTTGTT CTCGTCTCCT CGACACCCGW

841 WWW**YCW**WWWA TATATGGCGG CCGGTCATCA TCACCATCAC CATTGAGTTT AAACCCGCTG

901 ATCAGCCTCG ACTGTGCCTT CTAGTTGCCA GCCATCTGTT GTTTGCCCCT CCCCCGTGCC

961 TTCCTTGACC CTGGAAGGTG CCACTCCCAC TGTCCTTTCC TAATAAAATG AGGAAATTGC

1021 ATCGCATTGT CTGAGTAGGT GTCATTCTAT TCTGGGGGGT GGGGTGGGGC AGGACAGCAA

1081 GGGGGAGGAT TGGGAAGACA ATAGCAGGCA TGCTGGGGAT GCGGTGGGCT CTATGG

2. Cloning strategy for circular CLUSTER or LEAPER guide RNAs in the pEdit1.2 backbone

Sequence of the complete expression cassette in our **circular gRNA cloning vector (pTS1541, Tornado OHA vector U6)** that is based on our in-house pEdit1.2 backbone (see editing vector in [2]). The construct contains two twister ribozymes (P3 twister U2A ribozyme, P1 twister ribozyme) and adjacent ligation sequences to enable autocatalytic cleavage followed by processing by the endogenous ligase RtcB resulting in circularization. By using two Bbs-I sites the guide RNA sequence can be seamlessly added between the ligation sequences. The inserted sequence can either be an unstructured LEAPER guide RNA or a CLUSTER guide RNA containing a split-R/G motif, a specificity domain and a cluster of recruitment sequences. The circular guide RNA sequences listed in Supplementary Table S1 show the guide RNA ligation sequences after ribozyme processing. The guide RNAs are under control of an U6 promoter. A 7xT-stretch within the SV40 Poly(A) signal serves as pol3 termination signal.

```
1      GAGGGCCTAT TTCCCATGAT TCCTTCATAT TTGCATATAC GATACAAGGC TGTTAGAGAG
61     ATAATTAGAA TTAATTTGAC TGTAAACACA AAGATATTAG TACAAAATAC GTGACGTAGA
121    AAGTAATAAT TTCTTGGGTA GTTTGCAGTT TTAAAATTAT GTTTTAAAAT GGACTATCAT
181    ATGCTTACCG TAACTTGAAA GTATTTCGAT TTCTTGGCTT TATATATCTT GTGGAAAGGA
241    CGAAACACCG TGCTCGCTTC GGCAGCACAT ATACTAGTCG ACGCCATCAG TCGCCGGTCC
301    CAAGCCCGGA TAAAATGGGA GGGGGCGGGA AACCGCCTAA CCATGCCGAC TGATGGCAGT
361    AGTCTTCACT GGTACCGAGC TCGGATCCAC TGGAATTCGC CATGCATCTA GGGCCCTAGA
421    AGACTACTGC CATCAGTCGG CGTGGACTGT AGAACACTGC CAATGCCGGT CCCAAGCCCG
481    GATAAAAGTG GAGGGTACAG TCCACGCTCT AGAGCGGACT TCGGTCCGCT TTTTACTAGG
541    ACCTGCAGGC ATGCAAGCTT GACGTCGGTT ACCGATATCC ATATGGCGGC CGCATCGATC
601    TCGAGCCGCG GACTAGTAAC TTGTTTATTG CAGCTTATAA TGGTTACAAA TAAAGCAATA
661    GCATCACAAA TTTCACAAAT AAAGCATTTT TTTCACTGCA TTCTAGTTGT GGTTTGTCCA
721    AACTCATCAA TGTATCTTA
```

3. Cloning strategy for circular CLUSTER guide RNAs in the pAAV-GFP backbone

For AAV production the circular V27.2.4 splitR/G CLUSTER guide RNA was PCR-cloned into the pAAV-GFP backbone (Cell Biolabs) using the primer pair 5058+5059 on pTS2108 and the MluI and BglII restriction sites. The primers 5122-5123, the PCR template pTS1160 and the restriction site MluI were used to insert stuffer sequences adjusting the distance from the start of the first ITR to the end of the second ITR of each construct to ~4500 bp including the ITRs.

4. ORF & amino acid sequences:

ORF & amino acid sequence of the **murine_Mecp2_W104Amber-eGFP (pTS1034)** construct in the context of the pEGFP-N3 vector (Clonetech), under control of the CMV promoter and the SV40 polyA signal:

```
                   10        20        30        40        50        60
1 gaattccaccATGGCCGCCGCTGCCGCCACCGCCGCCGCCGCCGCCGCGCCGAGCGGAGGAGGAGGAGGA
1 EcoRI       M  A  A  A  A  A  T  A  A  A  A  A  A  P  S  G  G  G  G  G

                   70        80        90       100       110       120
61        GGCGAGGAGGAGAGACTGGAGGAAAAGTCAGAAGACCAGGATCTCCAGGGCCTCAGAGAC
21         G  E  E  E  R  L  E  E  K  S  E  D  Q  D  L  Q  G  L  R  D

                  130       140       150       160       170       180
121       AAGCCACTGAAGTTTAAGAAGGCGAAGAAAGACAAGAAGGAGGACAAAGAAGGCAAGCAT
41         K  P  L  K  F  K  K  A  K  K  D  K  K  E  D  K  E  G  K  H

                  190       200       210       220       230       240
181       GAGCCACTACAACCTTCAGCCCACCATTCTGCAGAGCCAGCAGAGGCAGGCAAAGCAGAA
61         E  P  L  Q  P  S  A  H  H  S  A  E  P  A  E  A  G  K  A  E

                  250       260       270       280       290       300
241       ACATCAGAAAGCTCAGGCTCTGCCCCAGCAGTGCCAGAAGCCTCGGCTTCCCCCAAACAG
81         T  S  E  S  S  G  S  A  P  A  V  P  E  A  S  A  S  P  K  Q

                  310       320       330       340       350       360
301       CGGCGCTCCATTATCCGTGACCGGGGACCTATGTATGATGACCCCACCTTGCCTGAAGGT
101        R  R  S  I  I  R  D  R  G  P  M  Y  D  D  P  T  L  P  E  G

                  370       380       390       400       410       420
361       TAGACACGAAAGCTTAAACAAAGGAAGTCTGGCCGATCTGCTGGAAAGTATGATGTATAT
121        *  T  R  K  L  K  Q  R  K  S  G  R  S  A  G  K  Y  D  V  Y
```

```
              430       440       450       460       470       480
421     TTGATCAATCCCCAGGGAAAAGCTTTTCGCTCTAAAGTAGAATTGATTGCATACTTTGAA
141      L  I  N  P  Q  G  K  A  F  R  S  K  V  E  L  I  A  Y  F  E

              490       500       510       520       530       540
481     AAGGTGGGAGACACCTCCTTGGACCCTAATGATTTTGACTTCACGGTAACTGGGAGAGGG
161      K  V  G  D  T  S  L  D  P  N  D  F  D  F  T  V  T  G  R  G

              550       560       570       580       590       600
541     AGCCCCTCCAGGAGAGAGCAGAAACCACCTAAGAAGCCCAAATCTCCCAAAGCTCCAGGA
181      S  P  S  R  R  E  Q  K  P  P  K  K  P  K  S  P  K  A  P  G

              610       620       630       640       650       660
601     ACTGGCAGGGGTCGGGGACGCCCCAAAGGGAGCGGCACTGGGAGACCAAAGGCAGCAGCA
201      T  G  R  G  R  G  R  P  K  G  S  G  T  G  R  P  K  A  A  A

              670       680       690       700       710       720
661     TCAGAAGGTGTTCAGGTGAAAAGGGTCCTGGAGAAGAGCCCTGGGAAACTTGTTGTCAAG
221      S  E  G  V  Q  V  K  R  V  L  E  K  S  P  G  K  L  V  V  K

              730       740       750       760       770       780
721     ATGCCTTTCCAAGCATCGCCTGGGGGTAAGGGTGAGGGAGGTGGGGCTACCACATCTGCC
241      M  P  F  Q  A  S  P  G  G  K  G  E  G  G  G  A  T  T  S  A

              790       800       810       820       830       840
781     CAGGTCATGGTGATCAAACGCCCTGGCAGAAAGCGAAAAGCTGAAGCTGACCCCCAGGCC
261      Q  V  M  V  I  K  R  P  G  R  K  R  K  A  E  A  D  P  Q  A

              850       860       870       880       890       900
841     ATTCCTAAGAAACGGGGTAGAAAGCCTGGGAGTGTGGTGGCAGCTGCTGCAGCTGAGGCC
281      I  P  K  K  R  G  R  K  P  G  S  V  V  A  A  A  A  A  E  A
```

```
                910       920       930       940       950       960
901      AAAAAGAAAGCCGTGAAGGAGTCTTCCATACGGTCTGTGCATGAGACTGTGCTCCCCATC
301       K  K  K  A  V  K  E  S  S  I  R  S  V  H  E  T  V  L  P  I

                970       980       990      1000      1010      1020
961      AAGAAGCGCAAGACCCGGGAGACGGTCAGCATCGAGGTCAAGGAAGTGGTGAAGCCCCTG
321       K  K  R  K  T  R  E  T  V  S  I  E  V  K  E  V  V  K  P  L

               1030      1040      1050      1060      1070      1080
1021     CTGGTGTCCACCCTTGGTGAGAAAAGCGGGAAGGGACTGAAGACCTGCAAGAGCCCTGGG
341       L  V  S  T  L  G  E  K  S  G  K  G  L  K  T  C  K  S  P  G

               1090      1100      1110      1120      1130      1140
1081     CGTAAAAGCAAGGAGAGCAGCCCCAAGGGGCGCAGCAGCAGTGCCTCCTCCCCACCTAAG
361       R  K  S  K  E  S  S  P  K  G  R  S  S  S  A  S  S  P  P  K

               1150      1160      1170      1180      1190      1200
1141     AAGGAGCACCATCATCACCACCATCACTCAGAGTCCACAAAGGCCCCCATGCCACTGCTC
381       K  E  H  H  H  H  H  H  H  S  E  S  T  K  A  P  M  P  L  L

               1210      1220      1230      1240      1250      1260
1201     CCATCCCCACCCCCACCTGAGCCTGAGAGCTCTGAGGACCCCATCAGCCCCCCTGAGCCT
401       P  S  P  P  P  P  E  P  E  S  S  E  D  P  I  S  P  P  E  P

               1270      1280      1290      1300      1310      1320
1261     CAGGACTTGAGCAGCAGCATCTGCAAAGAAGAGAAGATGCCCCGAGGAGGCTCACTGGAA
421       Q  D  L  S  S  S  I  C  K  E  E  K  M  P  R  G  G  S  L  E

               1330      1340      1350      1360      1370      1380
1321     AGCGATGGCTGCCCCAAGGAGCCAGCTAAGACTCAGCCTATGGTCGCCACCACTACCACA
441       S  D  G  C  P  K  E  P  A  K  T  Q  P  M  V  A  T  T  T  T
```

1390 1400 1410 1420 1430 1440

1381 GTTGCAGAAAAGTACAAACACCGAGGGGAGGGAGAGCGCAAAGACATTGTTTCATCTTCC

461 V A E K Y K H R G E G E R K D I V S S S

1450 1460 1470 1480 1490 1500

1441 ATGCCAAGGCCAAACAGAGAGGAGCCTGTGGACAGCCGGACGCCCGTGACCGAGAGAGTT

481 M P R P N R E E P V D S R T P V T E R V

1510 1520 1530 1540 1550 1560

1501 AGC**GGTACC***GCGGGCCCGGGATCCATCGCCACC***ATG**GTGAGCAAGGGCGAGGAGCTGTTC

KpnI ***Linker*** **start eGFP**

501 S G T A G P G S I A T M V S K G E E L F

1570 1580 1590 1600 1610 1620

1561 ACCGGGGTGGTGCCCATCCTGGTCGAGCTGGACGGCGACGTAAACGGCCACAAGTTCAGC

521 T G V V P I L V E L D G D V N G H K F S

1630 1640 1650 1660 1670 1680

1621 GTGTCCGGCGAGGGCGAGGGCGATGCCACCTACGGCAAGCTGACCCTGAAGTTCATCTGC

541 V S G E G E G D A T Y G K L T L K F I C

1690 1700 1710 1720 1730 1740

1681 ACCACCGGCAAGCTGCCCGTGCCCTGGCCCACCCTCGTGACCACCCTGACCTACGGCGTG

561 T T G K L P V P W P T L V T T L T Y G V

1750 1760 1770 1780 1790 1800

1741 CAGTGCTTCAGCCGCTACCCCGACCACATGAAGCAGCACGACTTCTTCAAGTCCGCCATG

581 Q C F S R Y P D H M K Q H D F F K S A M

1810 1820 1830 1840 1850 1860

1801 CCCGAAGGCTACGTCCAGGAGCGCACCATCTTCTTCAAGGACGACGGCAACTACAAGACC

```
601        P  E  G  Y  V  Q  E  R  T  I  F  F  K  D  D  G  N  Y  K  T

                1870      1880      1890      1900      1910      1920
1861       CGCGCCGAGGTGAAGTTCGAGGGCGACACCCTGGTGAACCGCATCGAGCTGAAGGGCATC
621        R  A  E  V  K  F  E  G  D  T  L  V  N  R  I  E  L  K  G  I

                1930      1940      1950      1960      1970      1980
1921       GACTTCAAGGAGGACGGCAACATCCTGGGGCACAAGCTGGAGTACAACTACAACAGCCAC
641        D  F  K  E  D  G  N  I  L  G  H  K  L  E  Y  N  Y  N  S  H

                1990      2000      2010      2020      2030      2040
1981       AACGTCTATATCATGGCCGACAAGCAGAAGAACGGCATCAAGGTGAACTTCAAGATCCGC
661        N  V  Y  I  M  A  D  K  Q  K  N  G  I  K  V  N  F  K  I  R

                2050      2060      2070      2080      2090      2100
2041       CACAACATCGAGGACGGCAGCGTGCAGCTCGCCGACCACTACCAGCAGAACACCCCCATC
681        H  N  I  E  D  G  S  V  Q  L  A  D  H  Y  Q  Q  N  T  P  I

                2110      2120      2130      2140      2150      2160
2101       GGCGACGGCCCCGTGCTGCTGCCCGACAACCACTACCTGAGCACCCAGTCCGCCCTGAGC
701        G  D  G  P  V  L  L  P  D  N  H  Y  L  S  T  Q  S  A  L  S

                2170      2180      2190      2200      2210      2220
2161       AAAGACCCCAACGAGAAGCGCGATCACATGGTCCTGCTGGAGTTCGTGACCGCCGCCGGG
721        K  D  P  N  E  K  R  D  H  M  V  L  L  E  F  V  T  A  A  G

                2230      2240      2250
2221       ATCACTCTCGGCATGGACGAGCTGTACAAGTAA
741        I  T  L  G  M  D  E  L  Y  K  *
```

ORF & amino acid sequence of the **human_PEX1_G843D** construct under control of the EF-1α promoter and the EF-1α polyA signal:

10 20 30 40 50 60

1 **ATG**TGGGGCAGCGATCGCCTGGCGGGTGCTGGGGGAGGCGGGGCGGCAGTGACTGTGGCC

1 M W G S D R L A G A G G G G A A V T V A

70 80 90 100 110 120

61 TTCACCAACGCTCGCGACTGCTTCCTCCACCTGCCGCGGCGTCTCGTGGCCCAGCTGCAT

21 F T N A R D C F L H L P R R L V A Q L H

130 140 150 160 170 180

121 CTGCTGCAGAATCAAGCTATAGAAGTGGTCTGGAGTCACCAGCCTGCATTCTTGAGCTGG

41 L L Q N Q A I E V V W S H Q P A F L S W

190 200 210 220 230 240

181 GTGGAAGGCAGGCATTTTAGTGATCAAGGTGAAAATGTGGCTGAAATTAACAGACAAGTT

61 V E G R H F S D Q G E N V A E I N R Q V

250 260 270 280 290 300

241 GGTCAAAAACTTGGACTCTCAAATGGGGGACAGGTATTTCTCAAGCCATGTTCCCATGTG

81 G Q K L G L S N G G Q V F L K P C S H V

310 320 330 340 350 360

301 GTATCTTGTCAACAAGTTGAGGTGGAACCCCTCTCAGCAGATGATTGGGAGATACTGGAG

101 V S C Q Q V E V E P L S A D D W E I L E

370 380 390 400 410 420

361 CTGCATGCTGTTTCCCTTGAACAACATCTTCTAGATCAAATTCGAATAGTTTTTCCAAAA

121 L H A V S L E Q H L L D Q I R I V F P K

430 440 450 460 470 480

```
421        GCCATTTTTCCTGTTTGGGTTGATCAACAAACGTACATATTTATCCAAATTGTTGCACTA
141         A  I  F  P  V  W  V  D  Q  Q  T  Y  I  F  I  Q  I  V  A  L

                  490       500       510       520       530       540
481        ATACCAGCTGCCTCTTATGGAAGGCTGGAAACTGACACCAAACTCCTTATTCAGCCAAAG
161         I  P  A  A  S  Y  G  R  L  E  T  D  T  K  L  L  I  Q  P  K

                  550       560       570       580       590       600
541        ACACGCCGAGCCAAAGAGAATACATTTTCAAAAGCTGATGCTGAATATAAAAAACTTCAT
181         T  R  R  A  K  E  N  T  F  S  K  A  D  A  E  Y  K  K  L  H

                  610       620       630       640       650       660
601        AGTTATGGAAGAGACCAGAAAGGAATGATGAAAGAACTTCAAACCAAGCAACTTCAGTCA
201         S  Y  G  R  D  Q  K  G  M  M  K  E  L  Q  T  K  Q  L  Q  S

                  670       680       690       700       710       720
661        AATACTGTGGGAATCACTGAATCTAATGAAAACGAGTCAGAGATTCCAGTTGACTCATCA
221         N  T  V  G  I  T  E  S  N  E  N  E  S  E  I  P  V  D  S  S

                  730       740       750       760       770       780
721        TCAGTAGCAAGTTTATGGACTATGATAGGAAGCATTTTTTCCTTTCAATCTGAGAAGAAA
241         S  V  A  S  L  W  T  M  I  G  S  I  F  S  F  Q  S  E  K  K

                  790       800       810       820       830       840
781        CAAGAGACATCTTGGGGTTTAACTGAAATCAATGCATTCAAAAATATGCAGTCAAAGGTT
261         Q  E  T  S  W  G  L  T  E  I  N  A  F  K  N  M  Q  S  K  V

                  850       860       870       880       890       900
841        GTTCCTCTAGACAATATTTTCAGAGTATGCAAATCTCAACCTCCTAGTATATATAACGCG
281         V  P  L  D  N  I  F  R  V  C  K  S  Q  P  P  S  I  Y  N  A

                  910       920       930       940       950       960
```

901 TCAGCAACCTCTGTTTTTCATAAACACTGTGCCATTCATGTATTTCCATGGGACCAGGAA

301 S A T S V F H K H C A I H V F P W D Q E

970 980 990 1000 1010 1020

961 TATTTTGATGTAGAGCCCAGCTTTACTGTGACATATGGAAAGCTAGTTAAGCTACTTTCT

321 Y F D V E P S F T V T Y G K L V K L L S

1030 1040 1050 1060 1070 1080

1021 CCAAAGCAACAGCAAAGTAAAACAAAACAAAATGTGTTATCACCTGAAAAAGAGAAGCAG

341 P K Q Q Q S K T K Q N V L S P E K E K Q

1090 1100 1110 1120 1130 1140

1081 ATGTCAGAGCCACTAGATCAAAAAAAAATTAGGTCAGATCATAATGAAGAAGATGAGAAG

361 M S E P L D Q K K I R S D H N E E D E K

1150 1160 1170 1180 1190 1200

1141 GCCTGTGTGCTACAAGTAGTCTGGAATGGACTTGAAGAATTGAACAATGCCATCAAATAT

381 A C V L Q V V W N G L E E L N N A I K Y

1210 1220 1230 1240 1250 1260

1201 ACCAAAAATGTAGAAGTTCTCCATCTTGGGAAAGTCTGGATTCCAGATGACCTGAGGAAG

401 T K N V E V L H L G K V W I P D D L R K

1270 1280 1290 1300 1310 1320

1261 AGACTAAATATAGAAATGCATGCCGTAGTCAGGATAACTCCAGTGGAAGTTACCCCTAAA

421 R L N I E M H A V V R I T P V E V T P K

1330 1340 1350 1360 1370 1380

1321 ATTCCAAGATCTCTAAAGTTACAACCTAGAGAGAATTTACCTAAAGACATAAGTGAAGAA

441 I P R S L K L Q P R E N L P K D I S E E

1390 1400 1410 1420 1430 1440

1381 GACATAAAAACTGTATTTTATTCATGGCTACAGCAGTCTACTACCACCATGCTTCCTTTG

461 D I K T V F Y S W L Q Q S T T T M L P L

1450 1460 1470 1480 1490 1500

1441 GTAATATCAGAGGAAGAATTTATTAAGCTGGAAACTAAAGATGGACTGAAGGAATTTTCT

481 V I S E E E F I K L E T K D G L K E F S

1510 1520 1530 1540 1550 1560

1501 CTGAGTATAGTTCATTCTTGGGAAAAAGAAAAAGATAAAAATATTTTTCTGTTGAGTCCC

501 L S I V H S W E K E K D K N I F L L S P

1570 1580 1590 1600 1610 1620

1561 AATTTGCTGCAGAAGACTACAATACAAGTCCTTCTAGATCCTATGGTAAAAGAAGAAAAC

521 N L L Q K T T I Q V L L D P M V K E E N

1630 1640 1650 1660 1670 1680

1621 AGTGAGGAAATTGACTTTATTCTTCCTTTTTTAAAGCTGAGCTCTTTGGGAGGAGTGAAT

541 S E E I D F I L P F L K L S S L G G V N

1690 1700 1710 1720 1730 1740

1681 TCCTTAGGCGTATCCTCCTTGGAGCACATCACTCACAGCCTCCTGGGACGCCCTTTGTCT

561 S L G V S S L E H I T H S L L G R P L S

1750 1760 1770 1780 1790 1800

1741 CGGCAGCTGATGTCTCTTGTTGCAGGACTTAGGAATGGAGCTCTTTTACTCACAGGAGGA

581 R Q L M S L V A G L R N G A L L L T G G

1810 1820 1830 1840 1850 1860

1801 AAGGGAAGTGGAAAATCAACTTTAGCCAAAGCAATCTGTAAAGAAGCATTTGACAAACTG

601 K G S G K S T L A K A I C K E A F D K L

1870 1880 1890 1900 1910 1920

1861 GATGCCCATGTGGAGAGAGTTGACTGTAAAGCTTTACGAGGAAAAAGGCTTGAAAACATA

621 D A H V E R V D C K A L R G K R L E N I

1930 1940 1950 1960 1970 1980

1921 CAAAAAACCCTAGAGGTGGCTTTCTCAGAGGCAGTGTGGATGCAGCCATCTGTTGTCCTG

641 Q K T L E V A F S E A V W M Q P S V V L

1990 2000 2010 2020 2030 2040

1981 CTGGATGACCTTGACCTCATTGCTGGACTGCCTGCTGTCCCGGAACATGAGCACAGTCCT

661 L D D L D L I A G L P A V P E H E H S P

2050 2060 2070 2080 2090 2100

2041 GATGCGGTGCAGAGCCAGCGGCTTGCTCATGCTTTGAATGATATGATAAAAGAGTTTATC

681 D A V Q S Q R L A H A L N D M I K E F I

2110 2120 2130 2140 2150 2160

2101 TCCATGGGAAGTTTGGTTGCACTGATTGCCACAAGTCAGTCTCAGCAATCTCTACATCCT

701 S M G S L V A L I A T S Q S Q Q S L H P

2170 2180 2190 2200 2210 2220

2161 TTACTTGTTTCTGCTCAAGGAGTTCACATATTTCAGTGCGTCCAACACATTCAGCCTCCT

721 L L V S A Q G V H I F Q C V Q H I Q P P

2230 2240 2250 2260 2270 2280

2221 AATCAGGAACAAAGATGTGAAATTCTGTGTAATGTAATAAAAAATAAATTGGACTGTGAT

741 N Q E Q R C E I L C N V I K N K L D C D

2290 2300 2310 2320 2330 2340

2281 ATAAACAAGTTCACCGATCTTGACCTGCAGCATGTAGCTAAAGAAACTGGAGGGTTTGTG

761 I N K F T D L D L Q H V A K E T G G F V

2350 2360 2370 2380 2390 2400

2341 GCTAGAGATTTTACAGTACTTGTGGATCGAGCCATACATTCTCGACTCTCTCGTCAGAGT

781 A R D F T V L V D R A I H S R L S R Q S

2410 2420 2430 2440 2450 2460

2401 ATATCCACCAGAGAAAAATTAGTTTTAACAACATTGGACTTCCAAAAGGCTCTCCGCGGA

801 I S T R E K L V L T T L D F Q K A L R G

2470 2480 2490 2500 2510 2520

2461 TTTCTTCCTGCGTCTTTGCGAAGTGTCAACCTGCATAAACCTAGAGACCTGGGTTGGGAC

821 F L P A S L R S V N L H K P R D L G W D

2530 2540 2550 2560 2570 2580

2521 AAGATT**GAT**GGGTTACATGAAGTTAGGCAGATACTCATGGATACTATCCAGTTACCTGCC

841 K I D G L H E V R Q I L M D T I Q L P A

2590 2600 2610 2620 2630 2640

2581 AAGTATCCAGAATTATTTGCAAACTTGCCCATACGACAAAGAACAGGAATACTGTTGTAT

861 K Y P E L F A N L P I R Q R T G I L L Y

2650 2660 2670 2680 2690 2700

2641 GGTCCGCCTGGAACAGGAAAAACCTTACTAGCTGGGGTAATTGCACGAGAGAGTAGAATG

881 G P P G T G K T L L A G V I A R E S R M

2710 2720 2730 2740 2750 2760

2701 AATTTTATAAGTGTCAAGGGGCCAGAGTTACTCAGCAAATACATTGGAGCAAGTGAACAA

901 N F I S V K G P E L L S K Y I G A S E Q

2770 2780 2790 2800 2810 2820

2761 GCTGTTCGGGATATTTTTATTAGAGCACAGGCTGCAAAGCCCTGCATTCTTTTCTTTGAT

921 A V R D I F I R A Q A A K P C I L F F D

2830 2840 2850 2860 2870 2880

```
2821    GAATTTGAATCCATTGCTCCTCGGCGGGGTCATGATAATACAGGAGTTACAGACCGAGTA
941      E  F  E  S  I  A  P  R  R  G  H  D  N  T  G  V  T  D  R  V

               2890      2900      2910      2920      2930      2940
2881    GTTAACCAGTTGCTGACTCAGTTGGATGGAGTAGAAGGCTTACAGGGTGTTTATGTATTG
961      V  N  Q  L  L  T  Q  L  D  G  V  E  G  L  Q  G  V  Y  V  L

               2950      2960      2970      2980      2990      3000
2941    GCTGCTACTAGTCGCCCTGACTTGATTGACCCTGCCCTGCTTAGGCCTGGTCGACTAGAT
981      A  A  T  S  R  P  D  L  I  D  P  A  L  L  R  P  G  R  L  D

               3010      3020      3030      3040      3050      3060
3001    AAATGTGTATACTGTCCTCCTCCTGATCAGGTGTCACGTCTTGAAATTTTAAATGTCCTC
1001     K  C  V  Y  C  P  P  P  D  Q  V  S  R  L  E  I  L  N  V  L

               3070      3080      3090      3100      3110      3120
3061    AGTGACTCTCTACCTCTGGCAGATGATGTTGACCTTCAGCATGTAGCATCAGTAACTGAC
1021     S  D  S  L  P  L  A  D  D  V  D  L  Q  H  V  A  S  V  T  D

               3130      3140      3150      3160      3170      3180
3121    TCCTTTACTGGAGCTGATCTGAAAGCTTTACTTTACAATGCCCAATTGGAGGCCTTACAT
1041     S  F  T  G  A  D  L  K  A  L  L  Y  N  A  Q  L  E  A  L  H

               3190      3200      3210      3220      3230      3240
3181    GGAATGCTGCTCTCGAGTGGACTCCAGGATGGAAGTTCCAGCTCTGATAGTGACCTAAGT
1061     G  M  L  L  S  S  G  L  Q  D  G  S  S  S  S  D  S  D  L  S

               3250      3260      3270      3280      3290      3300
3241    CTGTCTTCAATGGTCTTTCTTAACCATAGCAGTGGCTCTGACGATTCAGCTGGAGATGGA
1081     L  S  S  M  V  F  L  N  H  S  S  G  S  D  D  S  A  G  D  G

               3310      3320      3330      3340      3350      3360
```

```
3301    GAATGTGGCTTAGATCAGTCCCTTGTTTCTTTAGAGATGTCCGAGATCCTTCCAGATGAA
1101     E  C  G  L  D  Q  S  L  V  S  L  E  M  S  E  I  L  P  D  E

              3370      3380      3390      3400      3410      3420
3361    TCAAAATTCAATATGTACCGGCTCTACTTTGGAAGCTCTTATGAATCAGAACTTGGAAAT
1121     S  K  F  N  M  Y  R  L  Y  F  G  S  S  Y  E  S  E  L  G  N

              3430      3440      3450      3460      3470      3480
3421    GGAACCTCTTCTGATTTGAGCTCACAATGTCTCTCTGCACCAAGCTCCATGACTCAGGAT
1141     G  T  S  S  D  L  S  S  Q  C  L  S  A  P  S  S  M  T  Q  D

              3490      3500      3510      3520      3530      3540
3481    TTGCCTGGAGTTCCTGGGAAAGACCAGTTGTTTTCACAGCCTCCAGTGTTAAGGACAGCT
1161     L  P  G  V  P  G  K  D  Q  L  F  S  Q  P  P  V  L  R  T  A

              3550      3560      3570      3580      3590      3600
3541    TCACAAGAGGGTTGCCAAGAACTTACACAAGAACAAAGAGATCAACTGAGGGCAGATATC
1181     S  Q  E  G  C  Q  E  L  T  Q  E  Q  R  D  Q  L  R  A  D  I

              3610      3620      3630      3640      3650      3660
3601    AGTATTATCAAAGGCAGATACCGGAGCCAAAGTGGAGAGGACGAATCCATGAACCAACCA
1201     S  I  I  K  G  R  Y  R  S  Q  S  G  E  D  E  S  M  N  Q  P

              3670      3680      3690      3700      3710      3720
3661    GGACCAATCAAAACCAGACTGGCTATTAGTCAGTCACATTTAATGACTGCACTTGGTCAC
1221     G  P  I  K  T  R  L  A  I  S  Q  S  H  L  M  T  A  L  G  H

              3730      3740      3750      3760      3770      3780
3721    ACAAGACCATCCATTAGTGAAGATGACTGGAAGAATTTTGCTGAGCTATATGAAAGCTTT
1241     T  R  P  S  I  S  E  D  D  W  K  N  F  A  E  L  Y  E  S  F

              3790      3800      3810      3820      3830      3840
```

```
3781        CAAAATCCAAAGAGGAGAAAAAAATCAAAGTGGAACAATGTTTCGACCTGGACAGAAAGTA
1261         Q   N   P   K   R   R   K   N   Q   S   G   T   M   F   R   P   G   Q   K   V

                  3850       3860       3870       3880       3890
3841        ACTTTAGCAGAAAACCTGTATTTTCAGGGACACCATCACCATCACCATTAA
   1281     T   L   A   E   N   L   Y   F   Q   G   H   H   H   H   H   H   *
```

ORF & amino acid sequence of the **dPspCas13b_ADAR2DD_E488Q** construct under control of the CMV promoter and the bGH poly(A) signal:

```
                 10        20        30        40        50        60
1         ATGAACATCCCCGCTCTGGTGGAAAACCAGAAGAAGTACTTTGGCACCTACAGCGTGATG
1          M  N  I  P  A  L  V  E  N  Q  K  K  Y  F  G  T  Y  S  V  M

                 70        80        90       100       110       120
61        GCCATGCTGAACGCTCAGACCGTGCTGGACCACATCCAGAAGGTGGCCGATATTGAGGGC
21         A  M  L  N  A  Q  T  V  L  D  H  I  Q  K  V  A  D  I  E  G

                130       140       150       160       170       180
121       GAGCAGAACGAGAACAACGAGAATCTGTGGTTTCACCCCGTGATGAGCCACCTGTACAAC
41         E  Q  N  E  N  N  E  N  L  W  F  H  P  V  M  S  H  L  Y  N

                190       200       210       220       230       240
181       GCCAAGAACGGCTACGACAAGCAGCCCGAGAAAACCATGTTCATCATCGAGCGGCTGCAG
61         A  K  N  G  Y  D  K  Q  P  E  K  T  M  F  I  I  E  R  L  Q

                250       260       270       280       290       300
241       AGCTACTTCCCATTCCTGAAGATCATGGCCGAGAACCAGAGAGAGTACAGCAACGGCAAG
81         S  Y  F  P  F  L  K  I  M  A  E  N  Q  R  E  Y  S  N  G  K

                310       320       330       340       350       360
301       TACAAGCAGAACCGCGTGGAAGTGAACAGCAACGACATCTTCGAGGTGCTGAAGCGCGCC
101        Y  K  Q  N  R  V  E  V  N  S  N  D  I  F  E  V  L  K  R  A

                370       380       390       400       410       420
361       TTCGGCGTGCTGAAGATGTACAGGGACCTGACCAACGCATACAAGACCTACGAGGAAAAG
121        F  G  V  L  K  M  Y  R  D  L  T  N  A  Y  K  T  Y  E  E  K

                430       440       450       460       470       480
421       CTGAACGACGGCTGCGAGTTCCTGACCAGCACAGAGCAACCTCTGAGCGGCATGATCAAC
```

141 L N D G C E F L T S T E Q P L S G M I N

490 500 510 520 530 540

481 AACTACTACACAGTGGCCCTGCGGAACATGAACGAGAGATACGGCTACAAGACAGAGGAC

161 N Y Y T V A L R N M N E R Y G Y K T E D

550 560 570 580 590 600

541 CTGGCCTTCATCCAGGACAAGCGGTTCAAGTTCGTGAAGGACGCCTACGGCAAGAAAAAG

181 L A F I Q D K R F K F V K D A Y G K K K

610 620 630 640 650 660

601 TCCCAAGTGAATACCGGATTCTTCCTGAGCCTGCAGGACTACAACGGCGACACACAGAAG

201 S Q V N T G F F L S L Q D Y N G D T Q K

670 680 690 700 710 720

661 AAGCTGCACCTGAGCGGAGTGGGAATCGCCCTGCTGATCTGCCTGTTCCTGGACAAGCAG

221 K L H L S G V G I A L L I C L F L D K Q

730 740 750 760 770 780

721 TACATCAACATCTTTCTGAGCAGGCTGCCCATCTTCTCCAGCTACAATGCCCAGAGCGAG

241 Y I N I F L S R L P I F S S Y N A Q S E

790 800 810 820 830 840

781 GAACGGCGGATCATCATCAGATCCTTCGGCATCAACAGCATCAAGCTGCCCAAGGACCGG

261 E R R I I I R S F G I N S I K L P K D R

850 860 870 880 890 900

841 ATCCACAGCGAGAAGTCCAACAAGAGCGTGGCCATGGATATGCTCAACGAAGTGAAGCGG

281 I H S E K S N K S V A M D M L N E V K R

910 920 930 940 950 960

901 TGCCCCGACGAGCTGTTCACAACACTGTCTGCCGAGAAGCAGTCCCGGTTCAGAATCATC

301 C P D E L F T T L S A E K Q S R F R I I

970 980 990 1000 1010 1020

961 AGCGACGACCACAATGAAGTGCTGATGAAGCGGAGCAGCGACAGATTCGTGCCTCTGCTG

321 S D D H N E V L M K R S S D R F V P L L

1030 1040 1050 1060 1070 1080

1021 CTGCAGTATATCGATTACGGCAAGCTGTTCGACCACATCAGGTTCCACGTGAACATGGGC

341 L Q Y I D Y G K L F D H I R F H V N M G

1090 1100 1110 1120 1130 1140

1081 AAGCTGAGATACCTGCTGAAGGCCGACAAGACCTGCATCGACGGCCAGACCAGAGTCAGA

361 K L R Y L L K A D K T C I D G Q T R V R

1150 1160 1170 1180 1190 1200

1141 GTGATCGAGCAGCCCCTGAACGGCTTCGGCAGACTGGAAGAGGCCGAGACAATGCGGAAG

381 V I E Q P L N G F G R L E E A E T M R K

1210 1220 1230 1240 1250 1260

1201 CAAGAGAACGGCACCTTCGGCAACAGCGGCATCCGGATCAGAGACTTCGAGAACATGAAG

401 Q E N G T F G N S G I R I R D F E N M K

1270 1280 1290 1300 1310 1320

1261 CGGGACGACGCCAATCCTGCCAACTATCCCTACATCGTGGACACCTACACACACTACATC

421 R D D A N P A N Y P Y I V D T Y T H Y I

1330 1340 1350 1360 1370 1380

1321 CTGGAAAACAACAAGGTCGAGATGTTTATCAACGACAAAGAGGACAGCGCCCCACTGCTG

441 L E N N K V E M F I N D K E D S A P L L

1390 1400 1410 1420 1430 1440

1381 CCCGTGATCGAGGATGATAGATACGTGGTCAAGACAATCCCCAGCTGCCGGATGAGCACC

461 P V I E D D R Y V V K T I P S C R M S T

1450 1460 1470 1480 1490 1500

1441 CTGGAAATTCCAGCCATGGCCTTCCACATGTTTCTGTTCGGCAGCAAGAAAACCGAGAAG

481 L E I P A M A F H M F L F G S K K T E K

1510 1520 1530 1540 1550 1560

1501 CTGATCGTGGACGTGCACAACCGGTACAAGAGACTGTTCCAGGCCATGCAGAAAGAAGAA

501 L I V D V H N R Y K R L F Q A M Q K E E

1570 1580 1590 1600 1610 1620

1561 GTGACCGCCGAGAATATCGCCAGCTTCGGAATCGCCGAGAGCGACCTGCCTCAGAAGATC

521 V T A E N I A S F G I A E S D L P Q K I

1630 1640 1650 1660 1670 1680

1621 CTGGATCTGATCAGCGGCAATGCCCACGGCAAGGATGTGGACGCCTTCATCAGACTGACC

541 L D L I S G N A H G K D V D A F I R L T

1690 1700 1710 1720 1730 1740

1681 GTGGACGACATGCTGACCGACACCGAGCGGAGAATCAAGAGATTCAAGGACGACCGGAAG

561 V D D M L T D T E R R I K R F K D D R K

1750 1760 1770 1780 1790 1800

1741 TCCATTCGGAGCGCCGACAACAAGATGGGAAAGAGAGGCTTCAAGCAGATCTCCACAGGC

581 S I R S A D N K M G K R G F K Q I S T G

1810 1820 1830 1840 1850 1860

1801 AAGCTGGCCGACTTCCTGGCCAAGGACATCGTGCTGTTTCAGCCCAGCGTGAACGATGGC

601 K L A D F L A K D I V L F Q P S V N D G

1870 1880 1890 1900 1910 1920

1861 GAGAACAAGATCACCGGCCTGAACTACCGGATCATGCAGAGCGCCATTGCCGTGTACGAT

621 E N K I T G L N Y R I M Q S A I A V Y D

1930 1940 1950 1960 1970 1980

1921 AGCGGCGACGATTACGAGGCCAAGCAGCAGTTCAAGCTGATGTTCGAGAAGGCCCGGCTG

641 S G D D Y E A K Q Q F K L M F E K A R L

1990 2000 2010 2020 2030 2040

1981 ATCGGCAAGGGCACAACAGAGCCTCATCCATTTCTGTACAAGGTGTTCGCCCGCAGCATC

661 I G K G T T E P H P F L Y K V F A R S I

2050 2060 2070 2080 2090 2100

2041 CCCGCCAATGCCGTCGAGTTCTACGAGCGCTACCTGATCGAGCGGAAGTTCTACCTGACC

681 P A N A V E F Y E R Y L I E R K F Y L T

2110 2120 2130 2140 2150 2160

2101 GGCCTGTCCAACGAGATCAAGAAAGGCAACAGAGTGGATGTGCCCTTCATCCGGCGGGAC

701 G L S N E I K K G N R V D V P F I R R D

2170 2180 2190 2200 2210 2220

2161 CAGAACAAGTGGAAAACACCCGCCATGAAGACCCTGGGCAGAATCTACAGCGAGGATCTG

721 Q N K W K T P A M K T L G R I Y S E D L

2230 2240 2250 2260 2270 2280

2221 CCCGTGGAACTGCCCAGACAGATGTTCGACAATGAGATCAAGTCCCACCTGAAGTCCCTG

741 P V E L P R Q M F D N E I K S H L K S L

2290 2300 2310 2320 2330 2340

2281 CCACAGATGGAAGGCATCGACTTCAACAATGCCAACGTGACCTATCTGATCGCCGAGTAC

761 P Q M E G I D F N N A N V T Y L I A E Y

2350 2360 2370 2380 2390 2400

2341 ATGAAGAGAGTGCTGGACGACGACTTCCAGACCTTCTACCAGTGGAACCGCAACTACCGG

```
781         M  K  R  V  L  D  D  D  F  Q  T  F  Y  Q  W  N  R  N  Y  R

                2410      2420      2430      2440      2450      2460
2401        TACATGGACATGCTTAAGGGCGAGTACGACAGAAAGGGCTCCCTGCAGCACTGCTTCACC
801         Y  M  D  M  L  K  G  E  Y  D  R  K  G  S  L  Q  H  C  F  T

                2470      2480      2490      2500      2510      2520
2461        AGCGTGGAAGAGAGAGAAGGCCTCTGGAAAGAGCGGGCCTCCAGAACAGAGCGGTACAGA
821         S  V  E  E  R  E  G  L  W  K  E  R  A  S  R  T  E  R  Y  R

                2530      2540      2550      2560      2570      2580
2521        AAGCAGGCCAGCAACAAGATCCGCAGCAACCGGCAGATGAGAAACGCCAGCAGCGAAGAG
841         K  Q  A  S  N  K  I  R  S  N  R  Q  M  R  N  A  S  S  E  E

                2590      2600      2610      2620      2630      2640
2581        ATCGAGACAATCCTGGATAAGCGGCTGAGCAACAGCCGGAACGAGTACCAGAAAAGCGAG
861         I  E  T  I  L  D  K  R  L  S  N  S  R  N  E  Y  Q  K  S  E

                2650      2660      2670      2680      2690      2700
2641        AAAGTGATCCGGCGCTACAGAGTGCAGGATGCCCTGCTGTTTCTGCTGGCCAAAAAGACC
881         K  V  I  R  R  Y  R  V  Q  D  A  L  L  F  L  L  A  K  K  T

                2710      2720      2730      2740      2750      2760
2701        CTGACCGAACTGGCCGATTTCGACGGCGAGAGGTTCAAACTGAAAGAAATCATGCCCGAC
901         L  T  E  L  A  D  F  D  G  E  R  F  K  L  K  E  I  M  P  D

                2770      2780      2790      2800      2810      2820
2761        GCCGAGAAGGGAATCCTGAGCGAGATCATGCCCATGAGCTTCACCTTCGAGAAAGGCGGC
921         A  E  K  G  I  L  S  E  I  M  P  M  S  F  T  F  E  K  G  G

                2830      2840      2850      2860      2870      2880
2821        AAGAAGTACACCATCACCAGCGAGGGCATGAAGCTGAAGAACTACGGCGACTTCTTTGTG
```

941 K K Y T I T S E G M K L K N Y G D F F V

2890 2900 2910 2920 2930 2940
2881 CTGGCTAGCGACAAGAGGATCGGCAACCTGCTGGAACTCGTGGGCAGCGACATCGTGTCC
961 L A S D K R I G N L L E L V G S D I V S

2950 2960 2970 2980 2990 3000
2941 AAAGAGGATATCATGGAAGAGTTCAACAAATACGACCAGTGCAGGCCCGAGATCAGCTCC
981 K E D I M E E F N K Y D Q C R P E I S S

3010 3020 3030 3040 3050 3060
3001 ATCGTGTTCAACCTGGAAAAGTGGGCCTTCGACACATACCCCGAGCTGTCTGCCAGAGTG
1001 I V F N L E K W A F D T Y P E L S A R V

3070 3080 3090 3100 3110 3120
3061 GACCGGGAAGAGAAGGTGGACTTCAAGAGCATCCTGAAAATCCTGCTGAACAACAAGAAC
1021 D R E E K V D F K S I L K I L L N N K N

3130 3140 3150 3160 3170 3180
3121 ATCAACAAAGAGCAGAGCGACATCCTGCGGAAGATCCGGAACGCCTTCGATGCAAACAAT
1041 I N K E Q S D I L R K I R N A F D A N N

3190 3200 3210 3220 3230 3240
3181 TACCCCGACAAAGGCGTGGTGGAAATCAAGGCCCTGCCTGAGATCGCCATGAGCATCAAG
1061 Y P D K G V V E I K A L P E I A M S I K

3250 3260 3270 3280 3290 3300
3241 AAGGCCTTTGGGGAGTACGCCATCATGAAGGGAAGCCTGCAGCTGCCTCCACTTGAAAGA
1081 K A F G E Y A I M K G S L Q L P P L E R

3310 3320 3330 3340 3350 3360
3301 CTGACACTGGGATCCCAGCTGCATTTACCGCAGGTTTTAGCTGACGCTGTCTCACGCCTG

1101 L T L G S Q L H L P Q V L A D A V S R L

3370 3380 3390 3400 3410 3420

3361 GTCCTGGGTAAGTTTGGTGACCTGACCGACAACTTCTCCTCCCCTCACGCTCGCAGAAAA

1121 V L G K F G D L T D N F S S P H A R R K

3430 3440 3450 3460 3470 3480

3421 GTGCTGGCTGGAGTCGTCATGACAACAGGCACAGATGTTAAAGATGCCAAGGTGATAAGT

1141 V L A G V V M T T G T D V K D A K V I S

3490 3500 3510 3520 3530 3540

3481 GTTTCTACAGGAACAAAATGTATTAATGGTGAATACATGAGTGATCGTGGCCTTGCATTA

1161 V S T G T K C I N G E Y M S D R G L A L

3550 3560 3570 3580 3590 3600

3541 AATGACTGCCATGCAGAAATAATATCTCGGAGATCCTTGCTCAGATTTCTTTATACACAA

1181 N D C H A E I I S R R S L L R F L Y T Q

3610 3620 3630 3640 3650 3660

3601 CTTGAGCTTTACTTAAATAACAAAGATGATCAAAAAAGATCCATCTTTCAGAAATCAGAG

1201 L E L Y L N N K D D Q K R S I F Q K S E

3670 3680 3690 3700 3710 3720

3661 CGAGGGGGGTTTAGGCTGAAGGAGAATGTCCAGTTTCATCTGTACATCAGCACCTCTCCC

1221 R G G F R L K E N V Q F H L Y I S T S P

3730 3740 3750 3760 3770 3780

3721 TGTGGAGATGCCAGAATCTTCTCACCACATGAGCCAATCCTGGAAGAACCAGCAGATAGA

1241 C G D A R I F S P H E P I L E E P A D R

3790 3800 3810 3820 3830 3840

3781 CACCCAAATCGTAAAGCAAGAGGACAGCTACGGACCAAAATAGAGTCTGGTCAGGGGACG

```
1261      H  P  N  R  K  A  R  G  Q  L  R  T  K  I  E  S  G  Q  G  T

                3850      3860      3870      3880      3890      3900
3841      ATTCCAGTGCGCTCCAATGCGAGCATCCAAACGTGGGACGGGGTGCTGCAAGGGGAGCGG
1281      I  P  V  R  S  N  A  S  I  Q  T  W  D  G  V  L  Q  G  E  R

                3910      3920      3930      3940      3950      3960
3901      CTGCTCACCATGTCCTGCAGTGACAAGATTGCACGCTGGAACGTGGTGGGCATCCAGGGA
1301      L  L  T  M  S  C  S  D  K  I  A  R  W  N  V  V  G  I  Q  G

                3970      3980      3990      4000      4010      4020
3961      TCACTGCTCAGCATTTTCGTGGAGCCCATTTACTTCTCGAGCATCATCCTGGGCAGCCTT
1321      S  L  L  S  I  F  V  E  P  I  Y  F  S  S  I  I  L  G  S  L

                4030      4040      4050      4060      4070      4080
4021      TACCACGGGGACCACCTTTCCAGGGCCATGTACCAGCGGATCTCCAACATAGAGGACCTG
1341      Y  H  G  D  H  L  S  R  A  M  Y  Q  R  I  S  N  I  E  D  L

                4090      4100      4110      4120      4130      4140
4081      CCACCTCTCTACACCCTCAACAAGCCTTTGCTCAGTGGCATCAGCAATGCAGAAGCACGG
1361      P  P  L  Y  T  L  N  K  P  L  L  S  G  I  S  N  A  E  A  R

                4150      4160      4170      4180      4190      4200
4141      CAGCCAGGGAAGGCCCCCAACTTCAGTGTCAACTGGACGGTAGGCGACTCCGCTATTGAG
1381      Q  P  G  K  A  P  N  F  S  V  N  W  T  V  G  D  S  A  I  E

                4210      4220      4230      4240      4250      4260
4201      GTCATCAACGCCACGACTGGGAAGGATGAGCTGGGCCGCGCGTCCCGCCTGTGTAAGCAC
1401      V  I  N  A  T  T  G  K  D  E  L  G  R  A  S  R  L  C  K  H

                4270      4280      4290      4300      4310      4320
4261      GCGTTGTACTGTCGCTGGATGCGTGTGCACGGCAAGGTTCCCTCCCACTTACTACGCTCC
```

```
1421        A  L  Y  C  R  W  M  R  V  H  G  K  V  P  S  H  L  L  R  S

                4330      4340      4350      4360      4370      4380
4321        AAGATTACCAAGCCCAACGTGTACCATGAGTCCAAGCTGGCGGCAAAGGAGTACCAGGCC
1441        K  I  T  K  P  N  V  Y  H  E  S  K  L  A  A  K  E  Y  Q  A

                4390      4400      4410      4420      4430      4440
4381        GCCAAGGCGCGTCTGTTCACAGCCTTCATCAAGGCGGGGCTGGGGGCCTGGGTGGAGAAG
1461        A  K  A  R  L  F  T  A  F  I  K  A  G  L  G  A  W  V  E  K

                4450      4460      4470
4441        CCCACCGAGCAGGACCAGTTCTCACTCACGTAA
1481        P  T  E  Q  D  Q  F  S  L  T  *
```

ORF & amino acid sequence of the **4λN_ADAR2DD_E488Q** construct under control of the CMV promoter and the bGH poly(A) signal:

```
              10        20        30        40        50        60
1      ATGGACTACAAGGACGACGATGACAAACTAGTAAATGCCCGTACGCGCCGGCGCGAACGG
1       M  D  Y  K  D  D  D  D  K  L  V  N  A  R  T  R  R  R  E  R

              70        80        90       100       110       120
61     CGAGCAGAAAAGCAGGCACAGTGGAAGGCAGCTAATGGCGGAGGGGGAAGCGGGGGCGGT
21      R  A  E  K  Q  A  Q  W  K  A  A  N  G  G  G  G  S  G  G  G

             130       140       150       160       170       180
121    GGAAGTGGGGGTGGAGGCAGCAATGCGCGGACCCGCCGACGTGAACGGCGAGCAGAAAAA
41      G  S  G  G  G  G  S  N  A  R  T  R  R  R  E  R  R  A  E  K

             190       200       210       220       230       240
181    CAGGCGCAATGGAAAGCAGCCAACGGCGGCGGTGGAAGTGGAGGTGGTGGAAGCGGAGGC
61      Q  A  Q  W  K  A  A  N  G  G  G  G  S  G  G  G  G  S  G  G

             250       260       270       280       290       300
241    GGTGGTAGTAACGCCAGAACCCGACGCAGAGAGAGGAGGGCAGAGAAACAGGCCCAGTGG
81      G  G  S  N  A  R  T  R  R  R  E  R  R  A  E  K  Q  A  Q  W

             310       320       330       340       350       360
301    AAAGCCGCTAACGGAGGAGGGGGATCCGGTGGAGGCGGCAGTGGAGGCGGGGGATCTAAC
101     K  A  A  N  G  G  G  G  S  G  G  G  G  S  G  G  G  G  S  N

             370       380       390       400       410       420
361    GCACGAACACGACGACGTGAGCGTCGCGCTGAGAAACAAGCTCAATGGAAAGCTGCAAAC
121     A  R  T  R  R  R  E  R  R  A  E  K  Q  A  Q  W  K  A  A  N

             430       440       450       460       470       480
421    TTGCACTTGGATCAGACGCCATCTCGCCAGCCTATTCCCAGTGAGGGTCTTCAGCTGCAT
```

```
141         L  H  L  D  Q  T  P  S  R  Q  P  I  P  S  E  G  L  Q  L  H

                   490        500        510        520        530        540
481         TTACCGCAGGTTTTAGCTGACGCTGTCTCACGCCTGGTCCTGGGTAAGTTTGGTGACCTG
161         L  P  Q  V  L  A  D  A  V  S  R  L  V  L  G  K  F  G  D  L

                   550        560        570        580        590        600
541         ACCGACAACTTCTCCTCCCCTCACGCTCGCAGAAAAGTGCTGGCTGGAGTCGTCATGACA
181         T  D  N  F  S  S  P  H  A  R  R  K  V  L  A  G  V  V  M  T

                   610        620        630        640        650        660
601         ACAGGCACAGATGTTAAAGATGCCAAGGTGATAAGTGTTTCTACAGGAACAAAATGTATT
201         T  G  T  D  V  K  D  A  K  V  I  S  V  S  T  G  T  K  C  I

                   670        680        690        700        710        720
661         AATGGTGAATACATGAGTGATCGTGGCCTTGCATTAAATGACTGCCATGCAGAAATAATA
221         N  G  E  Y  M  S  D  R  G  L  A  L  N  D  C  H  A  E  I  I

                   730        740        750        760        770        780
721         TCTCGGAGATCCTTGCTCAGATTTCTTTATACACAACTTGAGCTTTACTTAAATAACAAA
241         S  R  R  S  L  L  R  F  L  Y  T  Q  L  E  L  Y  L  N  N  K

                   790        800        810        820        830        840
781         GATGATCAAAAAAGATCCATCTTTCAGAAATCAGAGCGAGGGGGGTTTAGGCTGAAGGAG
261         D  D  Q  K  R  S  I  F  Q  K  S  E  R  G  G  F  R  L  K  E

                   850        860        870        880        890        900
841         AATGTCCAGTTTCATCTGTACATCAGCACCTCTCCCTGTGGAGATGCCAGAATCTTCTCA
281         N  V  Q  F  H  L  Y  I  S  T  S  P  C  G  D  A  R  I  F  S

                   910        920        930        940        950        960
901         CCACATGAGCCAATCCTGGAAGAACCAGCAGATAGACACCCAAATCGTAAAGCAAGAGGA
```

```
301          P  H  E  P  I  L  E  E  P  A  D  R  H  P  N  R  K  A  R  G

                   970       980       990      1000      1010      1020
961         CAGCTACGGACCAAAATAGAGTCTGGTCAGGGGACGATTCCAGTGCGCTCCAATGCGAGC
321          Q  L  R  T  K  I  E  S  G  Q  G  T  I  P  V  R  S  N  A  S

                  1030      1040      1050      1060      1070      1080
1021        ATCCAAACGTGGGACGGGGTGCTGCAAGGGGAGCGGCTGCTCACCATGTCCTGCAGTGAC
341          I  Q  T  W  D  G  V  L  Q  G  E  R  L  L  T  M  S  C  S  D

                  1090      1100      1110      1120      1130      1140
1081        AAGATTGCACGCTGGAACGTGGTGGGCATCCAGGGATCCCTGCTCAGCATTTTCGTGGAG
361          K  I  A  R  W  N  V  V  G  I  Q  G  S  L  L  S  I  F  V  E

                  1150      1160      1170      1180      1190      1200
1141        CCCATTTACTTCTCGAGCATCATCCTGGGCAGCCTTTACCACGGGGACCACCTTTCCAGG
381          P  I  Y  F  S  S  I  I  L  G  S  L  Y  H  G  D  H  L  S  R

                  1210      1220      1230      1240      1250      1260
1201        GCCATGTACCAGCGGATCTCCAACATAGAGGACCTGCCACCTCTCTACACCCTCAACAAG
401          A  M  Y  Q  R  I  S  N  I  E  D  L  P  P  L  Y  T  L  N  K

                  1270      1280      1290      1300      1310      1320
1261        CCTTTGCTCAGTGGCATCAGCAATGCAGAAGCACGGCAGCCAGGGAAGGCCCCCAACTTC
421          P  L  L  S  G  I  S  N  A  E  A  R  Q  P  G  K  A  P  N  F

                  1330      1340      1350      1360      1370      1380
1321        AGTGTCAACTGGACGGTAGGCGACTCCGCTATTGAGGTCATCAACGCCACGACTGGGAAG
441          S  V  N  W  T  V  G  D  S  A  I  E  V  I  N  A  T  T  G  K

                  1390      1400      1410      1420      1430      1440
1381        GATGAGCTGGGCCGCGCGTCCCGCCTGTGTAAGCACGCGTTGTACTGTCGCTGGATGCGT
```

```
461        D  E  L  G  R  A  S  R  L  C  K  H  A  L  Y  C  R  W  M  R

                1450      1460      1470      1480      1490      1500
1441       GTGCACGGCAAGGTTCCCTCCCACTTACTACGCTCCAAGATTACCAAACCCAACGTGTAC
481        V  H  G  K  V  P  S  H  L  L  R  S  K  I  T  K  P  N  V  Y

                1510      1520      1530      1540      1550      1560
1501       CATGAGTCCAAGCTGGCGGCAAAGGAGTACCAGGCCGCCAAGGCGCGTCTGTTCACAGCC
501        H  E  S  K  L  A  A  K  E  Y  Q  A  A  K  A  R  L  F  T  A

                1570      1580      1590      1600      1610      1620
1561       TTCATCAAGGCGGGGCTGGGGGCCTGGGTGGAGAAGCCCACCGAGCAGGACCAGTTCTCA
521        F  I  K  A  G  L  G  A  W  V  E  K  P  T  E  Q  D  Q  F  S

                1630      1640      1650
1621       CTCACGCCACTAGTCCACCATCACCATCACCATTGA
541        L  T  P  L  V  H  H  H  H  H  H  *
```

5. Plasmid-maps & sequences:

Plasmid-map and sequence of the **pEGFP-N3 vector (Clontech):**

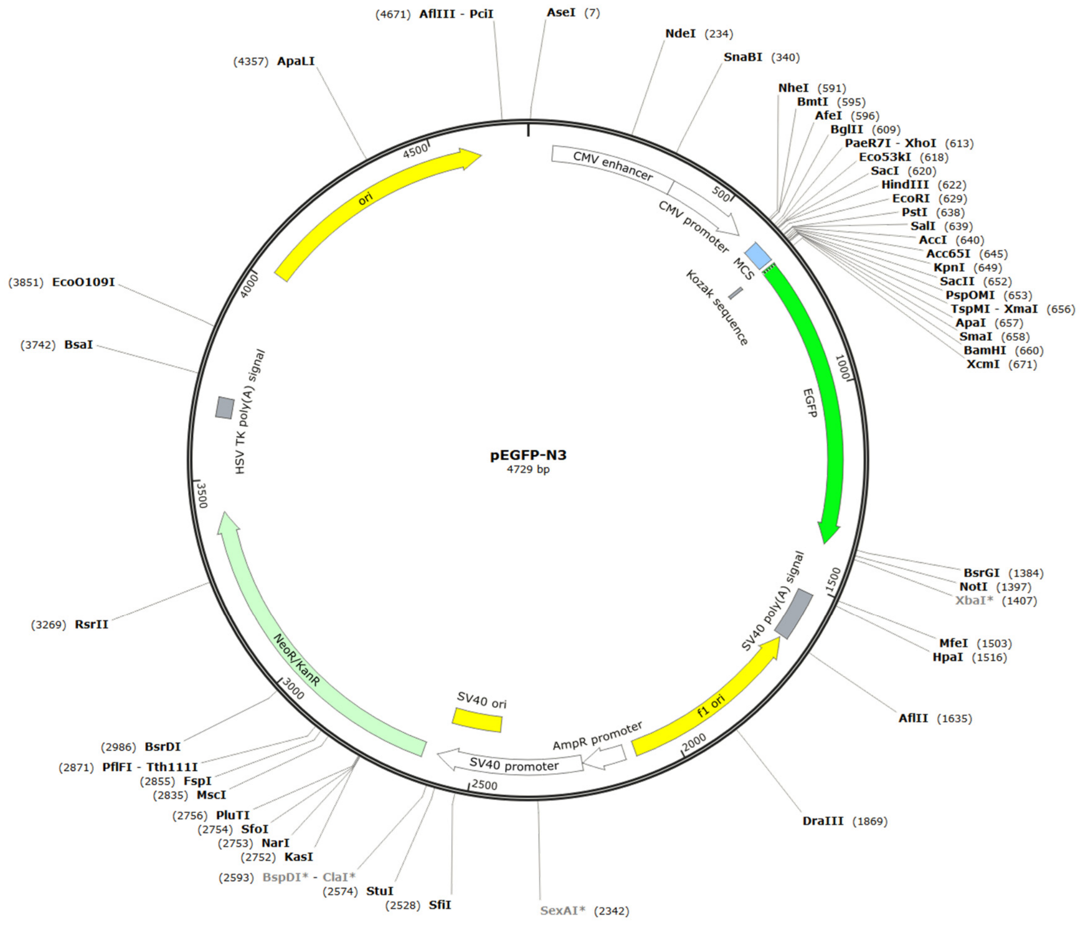


```
1     TAGTTATTAA TAGTAATCAA TTACGGGGTC ATTAGTTCAT AGCCCATATA TGGAGTTCCG
61    CGTTACATAA CTTACGGTAA ATGGCCCGCC TGGCTGACCG CCCAACGACC CCCGCCCATT
121   GACGTCAATA ATGACGTATG TTCCCATAGT AACGCCAATA GGGACTTTCC ATTGACGTCA
181   ATGGGTGGAG TATTTACGGT AAACTGCCCA CTTGGCAGTA CATCAAGTGT ATCATATGCC
241   AAGTACGCCC CCTATTGACG TCAATGACGG TAAATGGCCC GCCTGGCATT ATGCCCAGTA
301   CATGACCTTA TGGGACTTTC CTACTTGGCA GTACATCTAC GTATTAGTCA TCGCTATTAC
361   CATGGTGATG CGGTTTTGGC AGTACATCAA TGGGCGTGGA TAGCGGTTTG ACTCACGGGG
421   ATTTCCAAGT CTCCACCCCA TTGACGTCAA TGGGAGTTTG TTTTGGCACC AAAATCAACG
481   GGACTTTCCA AAATGTCGTA ACAACTCCGC CCCATTGACG CAAATGGGCG GTAGGCGTGT
541   ACGGTGGGAG GTCTATATAA GCAGAGCTGG TTTAGTGAAC CGTCAGATCC GCTAGCGCTA
601   CCGGACTCAG ATCTCGAGCT CAAGCTTCGA ATTCTGCAGT CGACGGTACC GCGGGCCCGG
661   GATCCATCGC CACCATGGTG AGCAAGGGCG AGGAGCTGTT CACCGGGGTG GTGCCCATCC
721   TGGTCGAGCT GGACGGCGAC GTAAACGGCC ACAAGTTCAG CGTGTCCGGC GAGGGCGAGG
781   GCGATGCCAC CTACGGCAAG CTGACCCTGA AGTTCATCTG CACCACCGGC AAGCTGCCCG
841   TGCCCTGGCC CACCCTCGTG ACCACCCTGA CCTACGGCGT GCAGTGCTTC AGCCGCTACC
901   CCGACCACAT GAAGCAGCAC GACTTCTTCA AGTCCGCCAT GCCCGAAGGC TACGTCCAGG
961   AGCGCACCAT CTTCTTCAAG GACGACGGCA ACTACAAGAC CCGCGCCGAG GTGAAGTTCG
```

1021 AGGGCGACAC CCTGGTGAAC CGCATCGAGC TGAAGGGCAT CGACTTCAAG GAGGACGGCA
1081 ACATCCTGGG GCACAAGCTG GAGTACAACT ACAACAGCCA CAACGTCTAT ATCATGGCCG
1141 ACAAGCAGAA GAACGGCATC AAGGTGAACT TCAAGATCCG CCACAACATC GAGGACGGCA
1201 GCGTGCAGCT CGCCGACCAC TACCAGCAGA ACACCCCCAT CGGCGACGGC CCCGTGCTGC
1261 TGCCCGACAA CCACTACCTG AGCACCCAGT CCGCCCTGAG CAAAGACCCC AACGAGAAGC
1321 GCGATCACAT GGTCCTGCTG GAGTTCGTGA CCGCCGCCGG GATCACTCTC GGCATGGACG
1381 AGCTGTACAA GTAAAGCGGC CGCGACTCTA GATCATAATC AGCCATACCA CATTTGTAGA
1441 GGTTTTACTT GCTTTAAAAA ACCTCCCACA CCTCCCCCTG AACCTGAAAC ATAAAATGAA
1501 TGCAATTGTT GTTGTTAACT TGTTTATTGC AGCTTATAAT GGTTACAAAT AAAGCAATAG
1561 CATCACAAAT TTCACAAATA AAGCATTTTT TTCACTGCAT TCTAGTTGTG GTTTGTCCAA
1621 ACTCATCAAT GTATCTTAAG GCGTAAATTG TAAGCGTTAA TATTTTGTTA AAATTCGCGT
1681 TAAATTTTTG TTAAATCAGC TCATTTTTTA ACCAATAGGC CGAAATCGGC AAAATCCCTT
1741 ATAAATCAAA AGAATAGACC GAGATAGGGT TGAGTGTTGT TCCAGTTTGG AACAAGAGTC
1801 CACTATTAAA GAACGTGGAC TCCAACGTCA AAGGGCGAAA AACCGTCTAT CAGGGCGATG
1861 GCCCACTACG TGAACCATCA CCCTAATCAA GTTTTTTGGG GTCGAGGTGC CGTAAAGCAC
1921 TAAATCGGAA CCCTAAAGGG AGCCCCCGAT TTAGAGCTTG ACGGGGAAAG CCGGCGAACG
1981 TGGCGAGAAA GGAAGGGAAG AAAGCGAAAG GAGCGGGCGC TAGGGCGCTG GCAAGTGTAG
2041 CGGTCACGCT GCGCGTAACC ACCACACCCG CCGCGCTTAA TGCGCCGCTA CAGGGCGCGT
2101 CAGGTGGCAC TTTTCGGGGA AATGTGCGCG GAACCCCTAT TTGTTTATTT TTCTAAATAC
2161 ATTCAAATAT GTATCCGCTC ATGAGACAAT AACCCTGATA AATGCTTCAA TAATATTGAA
2221 AAAGGAAGAG TCCTGAGGCG GAAAGAACCA GCTGTGGAAT GTGTGTCAGT TAGGGTGTGG
2281 AAAGTCCCCA GGCTCCCCAG CAGGCAGAAG TATGCAAAGC ATGCATCTCA ATTAGTCAGC
2341 AACCAGGTGT GGAAAGTCCC CAGGCTCCCC AGCAGGCAGA AGTATGCAAA GCATGCATCT
2401 CAATTAGTCA GCAACCATAG TCCCGCCCCT AACTCCGCCC ATCCCGCCCC TAACTCCGCC
2461 CAGTTCCGCC CATTCTCCGC CCCATGGCTG ACTAATTTTT TTTATTTATG CAGAGGCCGA
2521 GGCCGCCTCG GCCTCTGAGC TATTCCAGAA GTAGTGAGGA GGCTTTTTTG GAGGCCTAGG
2581 CTTTTGCAAA GATCGATCAA GAGACAGGAT GAGGATCGTT TCGCATGATT GAACAAGATG
2641 GATTGCACGC AGGTTCTCCG GCCGCTTGGG TGGAGAGGCT ATTCGGCTAT GACTGGGCAC
2701 AACAGACAAT CGGCTGCTCT GATGCCGCCG TGTTCCGGCT GTCAGCGCAG GGGCGCCCGG
2761 TTCTTTTTGT CAAGACCGAC CTGTCCGGTG CCCTGAATGA ACTGCAAGAC GAGGCAGCGC
2821 GGCTATCGTG GCTGGCCACG ACGGGCGTTC CTTGCGCAGC TGTGCTCGAC GTTGTCACTG
2881 AAGCGGGAAG GGACTGGCTG CTATTGGGCG AAGTGCCGGG GCAGGATCTC CTGTCATCTC
2941 ACCTTGCTCC TGCCGAGAAA GTATCCATCA TGGCTGATGC AATGCGGCGG CTGCATACGC
3001 TTGATCCGGC TACCTGCCCA TTCGACCACC AAGCGAAACA TCGCATCGAG CGAGCACGTA
3061 CTCGGATGGA AGCCGGTCTT GTCGATCAGG ATGATCTGGA CGAAGAGCAT CAGGGGCTCG
3121 CGCCAGCCGA ACTGTTCGCC AGGCTCAAGG CGAGCATGCC CGACGGCGAG GATCTCGTCG
3181 TGACCCATGG CGATGCCTGC TTGCCGAATA TCATGGTGGA AAATGGCCGC TTTTCTGGAT
3241 TCATCGACTG TGGCCGGCTG GGTGTGGCGG ACCGCTATCA GGACATAGCG TTGGCTACCC
3301 GTGATATTGC TGAAGAGCTT GGCGGCGAAT GGGCTGACCG CTTCCTCGTG CTTTACGGTA
3361 TCGCCGCTCC CGATTCGCAG CGCATCGCCT TCTATCGCCT TCTTGACGAG TTCTTCTGAG
3421 CGGGACTCTG GGGTTCGAAA TGACCGACCA AGCGACGCCC AACCTGCCAT CACGAGATTT
3481 CGATTCCACC GCCGCCTTCT ATGAAAGGTT GGGCTTCGGA ATCGTTTTCC GGGACGCCGG
3541 CTGGATGATC CTCCAGCGCG GGGATCTCAT GCTGGAGTTC TTCGCCCACC CTAGGGGGAG
3601 GCTAACTGAA ACACGGAAGG AGACAATACC GGAAGGAACC CGCGCTATGA CGGCAATAAA
3661 AAGACAGAAT AAAACGCACG GTGTTGGGTC GTTTGTTCAT AAACGCGGGG TTCGGTCCCA
3721 GGGCTGGCAC TCTGTCGATA CCCCACCGAG ACCCCATTGG GGCCAATACG CCCGCGTTTC
3781 TTCCTTTTCC CCACCCCACC CCCCAAGTTC GGGTGAAGGC CCAGGGCTCG CAGCCAACGT
3841 CGGGGCGGCA GGCCCTGCCA TAGCCTCAGG TTACTCATAT ATACTTTAGA TTGATTTAAA
3901 ACTTCATTTT TAATTTAAAA GGATCTAGGT GAAGATCCTT TTTGATAATC TCATGACCAA
3961 AATCCCTTAA CGTGAGTTTT CGTTCCACTG AGCGTCAGAC CCCGTAGAAA AGATCAAAGG
4021 ATCTTCTTGA GATCCTTTTT TTCTGCGCGT AATCTGCTGC TTGCAAACAA AAAAACCACC
4081 GCTACCAGCG GTGGTTTGTT TGCCGGATCA AGAGCTACCA ACTCTTTTTC CGAAGGTAAC
4141 TGGCTTCAGC AGAGCGCAGA TACCAAATAC TGTCCTTCTA GTGTAGCCGT AGTTAGGCCA
4201 CCACTTCAAG AACTCTGTAG CACCGCCTAC ATACCTCGCT CTGCTAATCC TGTTACCAGT
4261 GGCTGCTGCC AGTGGCGATA AGTCGTGTCT TACCGGGTTG GACTCAAGAC GATAGTTACC
4321 GGATAAGGCG CAGCGGTCGG GCTGAACGGG GGGTTCGTGC ACACAGCCCA GCTTGGAGCG
4381 AACGACCTAC ACCGAACTGA GATACCTACA GCGTGAGCTA TGAGAAAGCG CCACGCTTCC

4441 CGAAGGGAGA AAGGCGGACA GGTATCCGGT AAGCGGCAGG GTCGGAACAG GAGAGCGCAC
4501 GAGGGAGCTT CCAGGGGGAA ACGCCTGGTA TCTTTATAGT CCTGTCGGGT TTCGCCACCT
4561 CTGACTTGAG CGTCGATTTT TGTGATGCTC GTCAGGGGGG CGGAGCCTAT GGAAAAACGC
4621 CAGCAACGCG GCCTTTTTAC GGTTCCTGGC CTTTTGCTGG CCTTTTGCTC ACATGTTCTT
4681 TCCTGCGTTA TCCCCTGATT CTGTGGATAA CCGTATTACC GCCATGCAT

Plasmid-map and sequence of the **Tornado OHA vector U6 (pTS1541):**

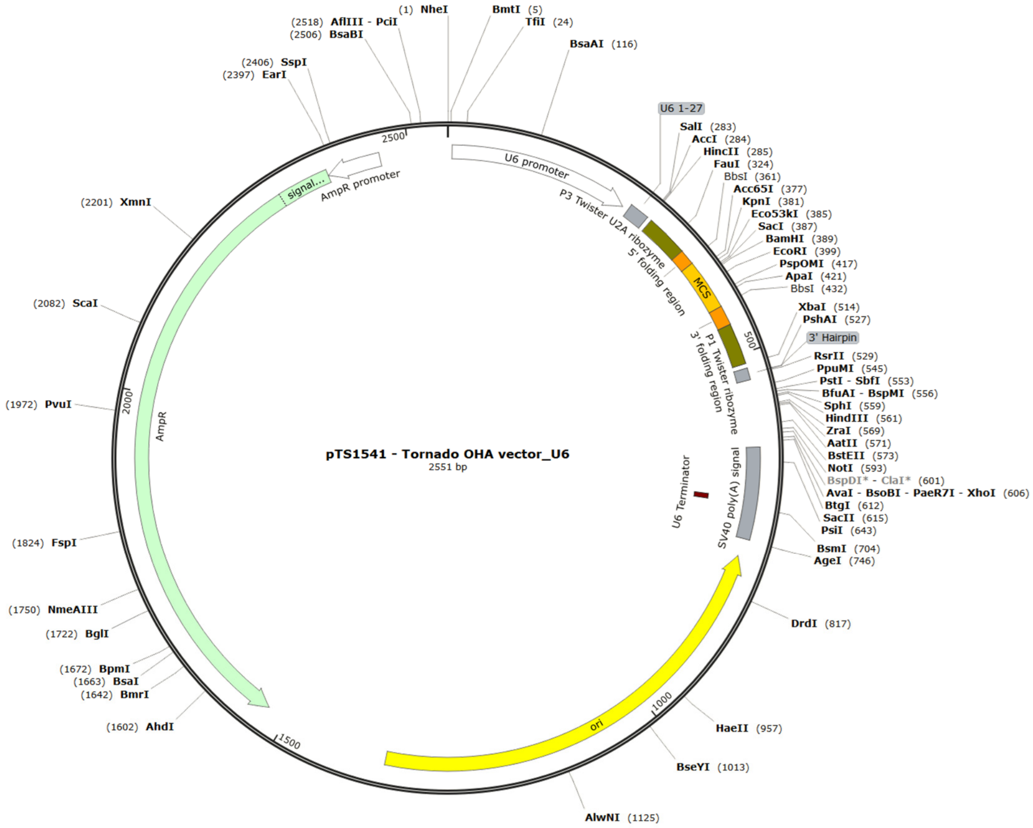

1 GCTAGCGAGG GCCTATTTCC CATGATTCCT TCATATTTGC ATATACGATA CAAGGCTGTT
61 AGAGAGATAA TTAGAATTAA TTTGACTGTA AACACAAAGA TATTAGTACA AAATACGTGA
121 CGTAGAAAGT AATAATTTCT TGGGTAGTTT GCAGTTTTAA AATTATGTTT TAAAATGGAC
181 TATCATATGC TTACCGTAAC TTGAAAGTAT TTCGATTTCT TGGCTTTATA TATCTTGTGG
241 AAAGGACGAA ACACCGTGCT CGCTTCGGCA GCACATATAC TAGTCGACGC CATCAGTCGC
301 CGGTCCCAAG CCCGGATAAA ATGGGAGGGG GCGGGAAACC GCCTAACCAT GCCGACTGAT
361 GGCAGTAGTC TTCACTGGTA CCGAGCTCGG ATCCACTGGA ATTCGCCATG CATCTAGGGC
421 CCTAGAAGAC TACTGCCATC AGTCGGCGTG GACTGTAGAA CACTGCCAAT GCCGGTCCCA
481 AGCCCGGATA AAAGTGGAGG GTACAGTCCA CGCTCTAGAG CGGACTTCGG TCCGCTTTTT
541 ACTAGGACCT GCAGGCATGC AAGCTTGACG TCGGTTACCG ATATCCATAT GGCGGCCGCA
601 TCGATCTCGA GCCGCGGACT AGTAACTTGT TTATTGCAGC TTATAATGGT TACAAATAAA
661 GCAATAGCAT CACAAATTTC ACAAATAAAG CATTTTTTTC ACTGCATTCT AGTTGTGGTT
721 TGTCCAAACT CATCAATGTA TCTTAACCGG TGGCCGCGTT GCTGGCGTTT TTCCATAGGC
781 TCCGCCCCCC TGACGAGCAT CACAAAAATC GACGCTCAAG TCAGAGGTGG CGAAACCCGA
841 CAGGACTATA AAGATACCAG GCGTTTCCCC CTGGAAGCTC CCTCGTGCGC TCTCCTGTTC
901 CGACCCTGCC GCTTACCGGA TACCTGTCCG CCTTTCTCCC TTCGGGAAGC GTGGCGCTTT
961 CTCATAGCTC ACGCTGTAGG TATCTCAGTT CGGTGTAGGT CGTTCGCTCC AAGCTGGGCT
1021 GTGTGCACGA ACCCCCCGTT CAGCCCGACC GCTGCGCCTT ATCCGGTAAC TATCGTCTTG
1081 AGTCCAACCC GGTAAGACAC GACTTATCGC CACTGGCAGC AGCCACTGGT AACAGGATTA
1141 GCAGAGCGAG GTATGTAGGC GGTGCTACAG AGTTCTTGAA GTGGTGGCCT AACTACGGCT
1201 ACACTAGAAG AACAGTATTT GGTATCTGCG CTCTGCTGAA GCCAGTTACC TTCGGAAAAA
1261 GAGTTGGTAG CTCTTGATCC GGCAAACAAA CCACCGCTGG TAGCGGTGGT TTTTTTGTTT

1321 GCAAGCAGCA GATTACGCGC AGAAAAAAAG GATCTCAAGA AGATCCTTTG ATCTTTTCTA
1381 CGGGGTCTGA CGCTCAGTGG AACGAAAACT CACGTTAAGG GATTTTGGTC ATGAGATTAT
1441 CAAAAAGGAT CTTCACCTAG ATCCTTTTAA ATTAAAAATG AAGTTTTAAA TCAATCTAAA
1501 GTATATATGA GTAAACTTGG TCTGACAGTT ACCAATGCTT AATCAGTGAG GCACCTATCT
1561 CAGCGATCTG TCTATTTCGT TCATCCATAG TTGCCTGACT CCCCGTCGTG TAGATAACTA
1621 CGATACGGGA GGGCTTACCA TCTGGCCCCA GTGCTGCAAT GATACCGCGA GACCCACGCT
1681 CACCGGCTCC AGATTTATCA GCAATAAACC AGCCAGCCGG AAGGGCCGAG CGCAGAAGTG
1741 GTCCTGCAAC TTTATCCGCC TCCATCCAGT CTATTAATTG TTGCCGGGAA GCTAGAGTAA
1801 GTAGTTCGCC AGTTAATAGT TTGCGCAACG TTGTTGCCAT TGCTACAGGC ATCGTGGTGT
1861 CACGCTCGTC GTTTGGTATG GCTTCATTCA GCTCCGGTTC CCAACGATCA AGGCGAGTTA
1921 CATGATCCCC CATGTTGTGC AAAAAAGCGG TTAGCTCCTT CGGTCCTCCG ATCGTTGTCA
1981 GAAGTAAGTT GGCCGCAGTG TTATCACTCA TGGTTATGGC AGCACTGCAT AATTCTCTTA
2041 CTGTCATGCC ATCCGTAAGA TGCTTTTCTG TGACTGGTGA GTACTCAACC AAGTCATTCT
2101 GAGAATAGTG TATGCGGCGA CCGAGTTGCT CTTGCCCGGC GTCAATACGG GATAATACCG
2161 CGCCACATAG CAGAACTTTA AAAGTGCTCA TCATTGGAAA ACGTTCTTCG GGGCGAAAAC
2221 TCTCAAGGAT CTTACCGCTG TTGAGATCCA GTTCGATGTA ACCCACTCGT GCACCCAACT
2281 GATCTTCAGC ATCTTTTACT TTCACCAGCG TTTCTGGGTG AGCAAAAACA GGAAGGCAAA
2341 ATGCCGCAAA AAAGGGAATA AGGGCGACAC GGAAATGTTG AATACTCATA CTCTTCCTTT
2401 TTCAATATTA TTGAAGCATT TATCAGGGTT ATTGTCTCAT GAGCGGATAC ATATTTGAAC
2461 GGCCGGATTT CTTGTCACGC TTTGATGCAT CGTTGGTGGT TGATGGATAT CTGACGAACA
2521 TGTGCACAGT GGTACAAGGT ATTCCTGGTT G

Plasmid-map and sequence of the **pAAV-GFP vector (Cell Biolabs):**

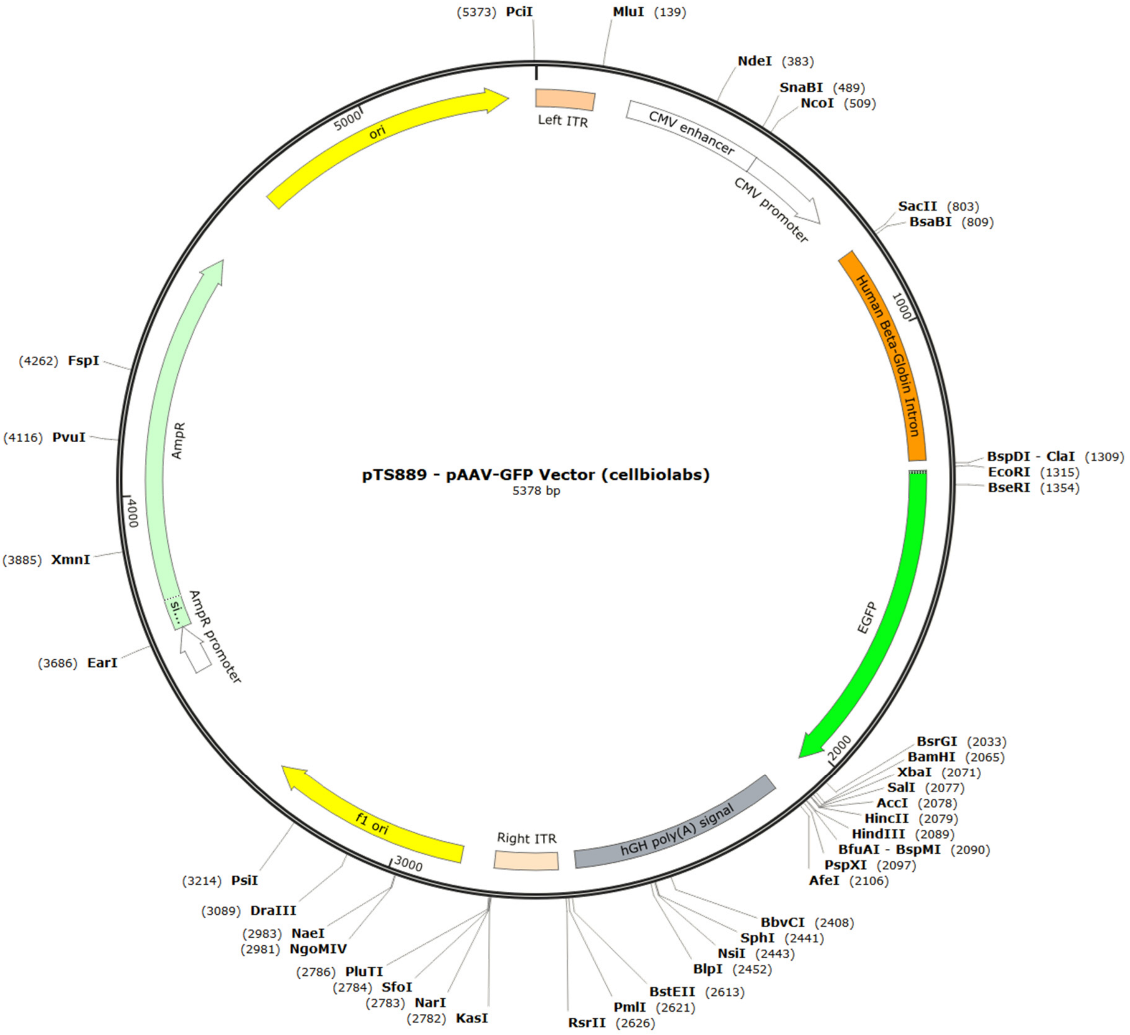

1	CCTGCAGGCA	GCTGCGCGCT	CGCTCGCTCA	CTGAGGCCGC	CCGGGCGTCG	GGCGACCTTT
61	GGTCGCCCGG	CCTCAGTGAG	CGAGCGAGCG	CGCAGAGAGG	GAGTGGCCAA	CTCCATCACT
121	AGGGGTTCCT	GCGGCCGCAC	GCGTGGAGCT	AGTTATTAAT	AGTAATCAAT	TACGGGGTCA
181	TTAGTTCATA	GCCCATATAT	GGAGTTCCGC	GTTACATAAC	TTACGGTAAA	TGGCCCGCCT
241	GGCTGACCGC	CCAACGACCC	CCGCCCATTG	ACGTCAATAA	TGACGTATGT	TCCCATAGTA
301	ACGTCAATAG	GGACTTTCCA	TTGACGTCAA	TGGGTGGAGT	ATTTACGGTA	AACTGCCCAC
361	TTGGCAGTAC	ATCAAGTGTA	TCATATGCCA	AGTACGCCCC	CTATTGACGT	CAATGACGGT
421	AAATGGCCCG	CCTGGCATTA	TGCCCAGTAC	ATGACCTTAT	GGGACTTTCC	TACTTGGCAG
481	TACATCTACG	TATTAGTCAT	CGCTATTACC	ATGGTGATGC	GGTTTTGGCA	GTACATCAAT
541	GGGCGTGGAT	AGCGGTTTGA	CTCACGGGGA	TTTCCAAGTC	TCCACCCCAT	TGACGTCAAT
601	GGGAGTTTGT	TTTGCACCAA	AATCAACGGG	ACTTTCCAAA	ATGTCGTAAC	AACTCCGCCC
661	CATTGACGCA	AATGGGCGGT	AGGCGTGTAC	GGTGGGAGGT	CTATATAAGC	AGAGCTCGTT
721	TAGTGAACCG	TCAGATCGCC	TGGAGACGCC	ATCCACGCTG	TTTTGACCTC	CATAGAAGAC
781	ACCGGGACCG	ATCCAGCCTC	CGCGGATTCG	AATCCCGGCC	GGGAACGGTG	CATTGGAACG
841	CGGATTCCCC	GTGCCAAGAG	TGACGTAAGT	ACCGCCTATA	GAGTCTATAG	GCCCACAAAA
901	AATGCTTTCT	TCTTTTAATA	TACTTTTTTG	TTTATCTTAT	TTCTAATACT	TTCCCTAATC
961	TCTTTCTTTC	AGGGCAATAA	TGATACAATG	TATCATGCCT	CTTTGCACCA	TTCTAAAGAA
1021	TAACAGTGAT	AATTTCTGGG	TTAAGGCAAT	AGCAATATTT	CTGCATATAA	ATATTTCTGC

1081 ATATAAATTG TAACTGATGT AAGAGGTTTC ATATTGCTAA TAGCAGCTAC AATCCAGCTA
1141 CCATTCTGCT TTTATTTTAT GGTTGGGATA AGGCTGGATT ATTCTGAGTC CAAGCTAGGC
1201 CCTTTTGCTA ATCATGTTCA TACCTCTTAT CTTCCTCCCA CAGCTCCTGG GCAACGTGCT
1261 GGTCTGTGTG CTGGCCCATC ACTTTGGCAA AGAATTGGGA TTCGAACATC GATTGAATTC
1321 TGAATGGTGA GCAAGGGCGA GGAGCTGTTC ACCGGGGTGG TGCCCATCCT GGTCGAGCTG
1381 GACGGCGACG TAAACGGCCA CAAGTTCAGC GTGTCCGGCG AGGGCGAGGG CGATGCCACC
1441 TACGGCAAGC TGACCCTGAA GTTCATCTGC ACCACCGGCA AGCTGCCCGT GCCCTGGCCC
1501 ACCCTCGTGA CCACCCTGAC CTACGGCGTG CAGTGCTTCA GCCGCTACCC CGACCACATG
1561 AAGCAGCACG ACTTCTTCAA GTCCGCCATG CCCGAAGGCT ACGTCCAGGA GCGCACCATC
1621 TTCTTCAAGG ACGACGGCAA CTACAAGACC CGCGCCGAGG TGAAGTTCGA GGGCGACACC
1681 CTGGTGAACC GCATCGAGCT GAAGGGCATC GACTTCAAGG AGGACGGCAA CATCCTGGGG
1741 CACAAGCTGG AGTACAACTA CAACAGCCAC AACGTCTATA TCATGGCCGA CAAGCAGAAG
1801 AACGGCATCA AGGTGAACTT CAAGATCCGC CACAACATCG AGGACGGCAG CGTGCAGCTC
1861 GCCGACCACT ACCAGCAGAA CACCCCCATC GGCGACGGCC CCGTGCTGCT GCCCGACAAC
1921 CACTACCTGA GCACCCAGTC CGCCCTGAGC AAAGACCCCA ACGAGAAGCG CGATCACATG
1981 GTCCTGCTGG AGTTCGTGAC CGCCGCCGGG ATCACTCTCG GCATGGACGA GCTGTACAAG
2041 TACTCAGATC TCGAGCTCAA GTAGGGATCC TCTAGAGTCG ACCTGCAGAA GCTTGCCTCG
2101 AGCAGCGCTG CTCGAGAGAT CTACGGGTGG CATCCCTGTG ACCCCTCCCC AGTGCCTCTC
2161 CTGGCCCTGG AAGTTGCCAC TCCAGTGCCC ACCAGCCTTG TCCTAATAAA ATTAAGTTGC
2221 ATCATTTTGT CTGACTAGGT GTCCTTCTAT AATATTATGG GGTGGAGGGG GGTGGTATGG
2281 AGCAAGGGGC AAGTTGGGAA GACAACCTGT AGGGCCTGCG GGGTCTATTG GGAACCAAGC
2341 TGGAGTGCAG TGGCACAATC TTGGCTCACT GCAATCTCCG CCTCCTGGGT TCAAGCGATT
2401 CTCCTGCCTC AGCCTCCCGA GTTGTTGGGA TTCCAGGCAT GCATGACCAG GCTCAGCTAA
2461 TTTTTGTTTT TTTGGTAGAG ACGGGGTTTC ACCATATTGG CCAGGCTGGT CTCCAACTCC
2521 TAATCTCAGG TGATCTACCC ACCTTGGCCT CCCAAATTGC TGGGATTACA GGCGTGAACC
2581 ACTGCTCCCT TCCCTGTCCT TCTGATTTTG TAGGTAACCA CGTGCGGACC GAGCGGCCGC
2641 AGGAACCCCT AGTGATGGAG TTGGCCACTC CCTCTCTGCG CGCTCGCTCG CTCACTGAGG
2701 CCGGGCGACC AAAGGTCGCC CGACGCCCGG GCTTTGCCCG GGCGGCCTCA GTGAGCGAGC
2761 GAGCGCGCAG CTGCCTGCAG GGGCGCCTGA TGCGGTATTT TCTCCTTACG CATCTGTGCG
2821 GTATTTCACA CCGCATACGT CAAAGCAACC ATAGTACGCG CCCTGTAGCG GCGCATTAAG
2881 CGCGGCGGGT GTGGTGGTTA CGCGCAGCGT GACCGCTACA CTTGCCAGCG CCCTAGCGCC
2941 CGCTCCTTTC GCTTTCTTCC CTTCCTTTCT CGCCACGTTC GCCGGCTTTC CCCGTCAAGC
3001 TCTAAATCGG GGGCTCCCTT TAGGGTTCCG ATTTAGTGCT TTACGGCACC TCGACCCCAA
3061 AAAACTTGAT TTGGGTGATG GTTCACGTAG TGGGCCATCG CCCTGATAGA CGGTTTTTCG
3121 CCCTTTGACG TTGGAGTCCA CGTTCTTTAA TAGTGGACTC TTGTTCCAAA CTGGAACAAC
3181 ACTCAACCCT ATCTCGGGCT ATTCTTTTGA TTTATAAGGG ATTTTGCCGA TTTCGGCCTA
3241 TTGGTTAAAA AATGAGCTGA TTTAACAAAA ATTTAACGCG AATTTTAACA AAATATTAAC
3301 GTTTACAATT TTATGGTGCA CTCTCAGTAC AATCTGCTCT GATGCCGCAT AGTTAAGCCA
3361 GCCCCGACAC CCGCCAACAC CCGCTGACGC GCCCTGACGG GCTTGTCTGC TCCCGGCATC
3421 CGCTTACAGA CAAGCTGTGA CCGTCTCCGG GAGCTGCATG TGTCAGAGGT TTTCACCGTC
3481 ATCACCGAAA CGCGCGAGAC GAAAGGGCCT CGTGATACGC CTATTTTTAT AGGTTAATGT
3541 CATGATAATA ATGGTTTCTT AGACGTCAGG TGGCACTTTT CGGGGAAATG TGCGCGGAAC
3601 CCCTATTTGT TTATTTTTCT AAATACATTC AAATATGTAT CCGCTCATGA GACAATAACC
3661 CTGATAAATG CTTCAATAAT ATTGAAAAAG GAAGAGTATG AGTATTCAAC ATTTCCGTGT
3721 CGCCCTTATT CCCTTTTTTG CGGCATTTTG CCTTCCTGTT TTTGCTCACC CAGAAACGCT
3781 GGTGAAAGTA AAAGATGCTG AAGATCAGTT GGGTGCACGA GTGGGTTACA TCGAACTGGA
3841 TCTCAACAGC GGTAAGATCC TTGAGAGTTT TCGCCCCGAA GAACGTTTTC CAATGATGAG
3901 CACTTTTAAA GTTCTGCTAT GTGGCGCGGT ATTATCCCGT ATTGACGCCG GGCAAGAGCA
3961 ACTCGGTCGC CGCATACACT ATTCTCAGAA TGACTTGGTT GAGTACTCAC CAGTCACAGA
4021 AAAGCATCTT ACGGATGGCA TGACAGTAAG AGAATTATGC AGTGCTGCCA TAACCATGAG
4081 TGATAACACT GCGGCCAACT TACTTCTGAC AACGATCGGA GGACCGAAGG AGCTAACCGC
4141 TTTTTTGCAC AACATGGGGG ATCATGTAAC TCGCCTTGAT CGTTGGGAAC CGGAGCTGAA
4201 TGAAGCCATA CCAAACGACG AGCGTGACAC CACGATGCCT GTAGCAATGG CAACAACGTT
4261 GCGCAAACTA TTAACTGGCG AACTACTTAC TCTAGCTTCC CGGCAACAAT TAATAGACTG
4321 GATGGAGGCG GATAAAGTTG CAGGACCACT TCTGCGCTCG GCCCTTCCGG CTGGCTGGTT
4381 TATTGCTGAT AAATCTGGAG CCGGTGAGCG TGGGTCTCGC GGTATCATTG CAGCACTGGG
4441 GCCAGATGGT AAGCCCTCCC GTATCGTAGT TATCTACACG ACGGGGAGTC AGGCAACTAT

4501 GGATGAACGA AATAGACAGA TCGCTGAGAT AGGTGCCTCA CTGATTAAGC ATTGGTAACT
4561 GTCAGACCAA GTTTACTCAT ATATACTTTA GATTGATTTA AAACTTCATT TTTAATTTAA
4621 AAGGATCTAG GTGAAGATCC TTTTTGATAA TCTCATGACC AAAATCCCTT AACGTGAGTT
4681 TTCGTTCCAC TGAGCGTCAG ACCCCGTAGA AAAGATCAAA GGATCTTCTT GAGATCCTTT
4741 TTTTCTGCGC GTAATCTGCT GCTTGCAAAC AAAAAAACCA CCGCTACCAG CGGTGGTTTG
4801 TTTGCCGGAT CAAGAGCTAC CAACTCTTTT TCCGAAGGTA ACTGGCTTCA GCAGAGCGCA
4861 GATACCAAAT ACTGTCCTTC TAGTGTAGCC GTAGTTAGGC CACCACTTCA AGAACTCTGT
4921 AGCACCGCCT ACATACCTCG CTCTGCTAAT CCTGTTACCA GTGGCTGCTG CCAGTGGCGA
4981 TAAGTCGTGT CTTACCGGGT TGGACTCAAG ACGATAGTTA CCGGATAAGG CGCAGCGGTC
5041 GGGCTGAACG GGGGGTTCGT GCACACAGCC CAGCTTGGAG CGAACGACCT ACACCGAACT
5101 GAGATACCTA CAGCGTGAGC TATGAGAAAG CGCCACGCTT CCCGAAGGGA GAAAGGCGGA
5161 CAGGTATCCG GTAAGCGGCA GGGTCGGAAC AGGAGAGCGC ACGAGGGAGC TTCCAGGGGG
5221 AAACGCCTGG TATCTTTATA GTCCTGTCGG GTTTCGCCAC CTCTGACTTG AGCGTCGATT
5281 TTTGTGATGC TCGTCAGGGG GGCGGAGCCT ATGGAAAAAC GCCAGCAACG CGGCCTTTTT
5341 ACGGTTCCTG GCCTTTTGCT GGCCTTTTGC TCACATGT

Plasmid-map and sequence of **circ-mMecp2-V27.2.4_gRNA_pEdit_no-stuffer (pTS2108):**

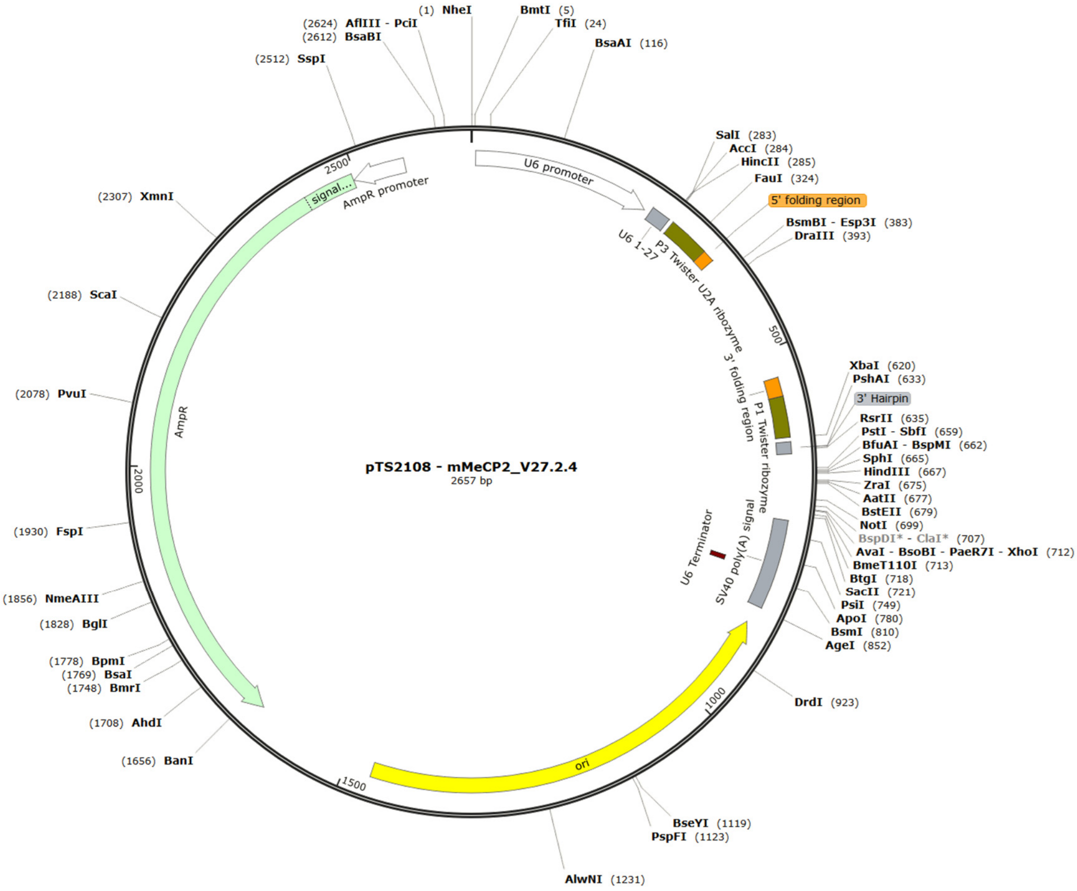

1 GCTAGCGAGG GCCTATTTCC CATGATTCCT TCATATTTGC ATATACGATA CAAGGCTGTT
61 AGAGAGATAA TTAGAATTAA TTTGACTGTA AACACAAAGA TATTAGTACA AAATACGTGA
121 CGTAGAAAGT AATAATTTCT TGGGTAGTTT GCAGTTTTAA AATTATGTTT TAAAATGGAC
181 TATCATATGC TTACCGTAAC TTGAAAGTAT TTCGATTTCT TGGCTTTATA TATCTTGTGG
241 AAAGGACGAA ACACCGTGCT CGCTTCGGCA GCACATATAC TAGTCGACGC CATCAGTCGC
301 CGGTCCCAAG CCCGGATAAA ATGGGAGGGG GCGGGAAACC GCCTAACCAT GCCGACTGAT
361 GGCAGCATGT TGTTCTCGTC TCCTCGACAC CTCGTGTCCA ACCTTCAGGC AAAAAGTCAT
421 CATGCATAGG TCCCCAAACA CGGATGATGG AGCGCCGCAA AGATCAAATA TACATCGTAC
481 TAAATCCAGC AGATCGGCCA GACTAAAAAG GTGTCGAGAA GAGGAGAACA ATATCTTTCT
541 GCCATCAGTC GGCGTGGACT GTAGAACACT GCCAATGCCG GTCCCAAGCC CGGATAAAAG
601 TGGAGGGTAC AGTCCACGCT CTAGAGCGGA CTTCGGTCCG CTTTTTACTA GGACCTGCAG
661 GCATGCAAGC TTGACGTCGG TTACCGATAT CCATATGGCG GCCGCATCGA TCTCGAGCCG
721 CGGACTAGTA ACTTGTTTAT TGCAGCTTAT AATGGTTACA AATAAAGCAA TAGCATCACA
781 AATTTCACAA ATAAAGCATT TTTTTCACTG CATTCTAGTT GTGGTTTGTC CAAACTCATC
841 AATGTATCTT AACCGGTGGC CGCGTTGCTG GCGTTTTTCC ATAGGCTCCG CCCCCCTGAC
901 GAGCATCACA AAAATCGACG CTCAAGTCAG AGGTGGCGAA ACCCGACAGG ACTATAAAGA
961 TACCAGGCGT TTCCCCCTGG AAGCTCCCTC GTGCGCTCTC CTGTTCCGAC CCTGCCGCTT
1021 ACCGGATACC TGTCCGCCTT TCTCCCTTCG GGAAGCGTGG CGCTTTCTCA TAGCTCACGC
1081 TGTAGGTATC TCAGTTCGGT GTAGGTCGTT CGCTCCAAGC TGGGCTGTGT GCACGAACCC
1141 CCCGTTCAGC CCGACCGCTG CGCCTTATCC GGTAACTATC GTCTTGAGTC CAACCCGGTA
1201 AGACACGACT TATCGCCACT GGCAGCAGCC ACTGGTAACA GGATTAGCAG AGCGAGGTAT
1261 GTAGGCGGTG CTACAGAGTT CTTGAAGTGG TGGCCTAACT ACGGCTACAC TAGAAGAACA
1321 GTATTTGGTA TCTGCGCTCT GCTGAAGCCA GTTACCTTCG GAAAAAGAGT TGGTAGCTCT

1381 TGATCCGGCA AACAAACCAC CGCTGGTAGC GGTGGTTTTT TTGTTTGCAA GCAGCAGATT
1441 ACGCGCAGAA AAAAAGGATC TCAAGAAGAT CCTTTGATCT TTTCTACGGG GTCTGACGCT
1501 CAGTGGAACG AAAACTCACG TTAAGGGATT TTGGTCATGA GATTATCAAA AAGGATCTTC
1561 ACCTAGATCC TTTTAAATTA AAAATGAAGT TTTAAATCAA TCTAAAGTAT ATATGAGTAA
1621 ACTTGGTCTG ACAGTTACCA ATGCTTAATC AGTGAGGCAC CTATCTCAGC GATCTGTCTA
1681 TTTCGTTCAT CCATAGTTGC CTGACTCCCC GTCGTGTAGA TAACTACGAT ACGGGAGGGC
1741 TTACCATCTG GCCCCAGTGC TGCAATGATA CCGCGAGACC CACGCTCACC GGCTCCAGAT
1801 TTATCAGCAA TAAACCAGCC AGCCGGAAGG GCCGAGCGCA GAAGTGGTCC TGCAACTTTA
1861 TCCGCCTCCA TCCAGTCTAT TAATTGTTGC CGGGAAGCTA GAGTAAGTAG TTCGCCAGTT
1921 AATAGTTTGC GCAACGTTGT TGCCATTGCT ACAGGCATCG TGGTGTCACG CTCGTCGTTT
1981 GGTATGGCTT CATTCAGCTC CGGTTCCCAA CGATCAAGGC GAGTTACATG ATCCCCCATG
2041 TTGTGCAAAA AAGCGGTTAG CTCCTTCGGT CCTCCGATCG TTGTCAGAAG TAAGTTGGCC
2101 GCAGTGTTAT CACTCATGGT TATGGCAGCA CTGCATAATT CTCTTACTGT CATGCCATCC
2161 GTAAGATGCT TTTCTGTGAC TGGTGAGTAC TCAACCAAGT CATTCTGAGA ATAGTGTATG
2221 CGGCGACCGA GTTGCTCTTG CCCGGCGTCA ATACGGGATA ATACCGCGCC ACATAGCAGA
2281 ACTTTAAAAG TGCTCATCAT TGGAAAACGT TCTTCGGGGC GAAAACTCTC AAGGATCTTA
2341 CCGCTGTTGA GATCCAGTTC GATGTAACCC ACTCGTGCAC CCAACTGATC TTCAGCATCT
2401 TTTACTTTCA CCAGCGTTTC TGGGTGAGCA AAAACAGGAA GGCAAAATGC CGCAAAAAAG
2461 GGAATAAGGG CGACACGGAA ATGTTGAATA CTCATACTCT TCCTTTTTCA ATATTATTGA
2521 AGCATTTATC AGGGTTATTG TCTCATGAGC GGATACATAT TTGAACGGCC GGATTTCTTG
2581 TCACGCTTTG ATGCATCGTT GGTGGTTGAT GGATATCTGA CGAACATGTG CACAGTGGTA
2641 CAAGGTATTC CTGGTTG

Plasmid-map and sequence of **circ-mMecp2-V27.2.4_gRNA_pAAV_no-stuffer (pTS2123):**

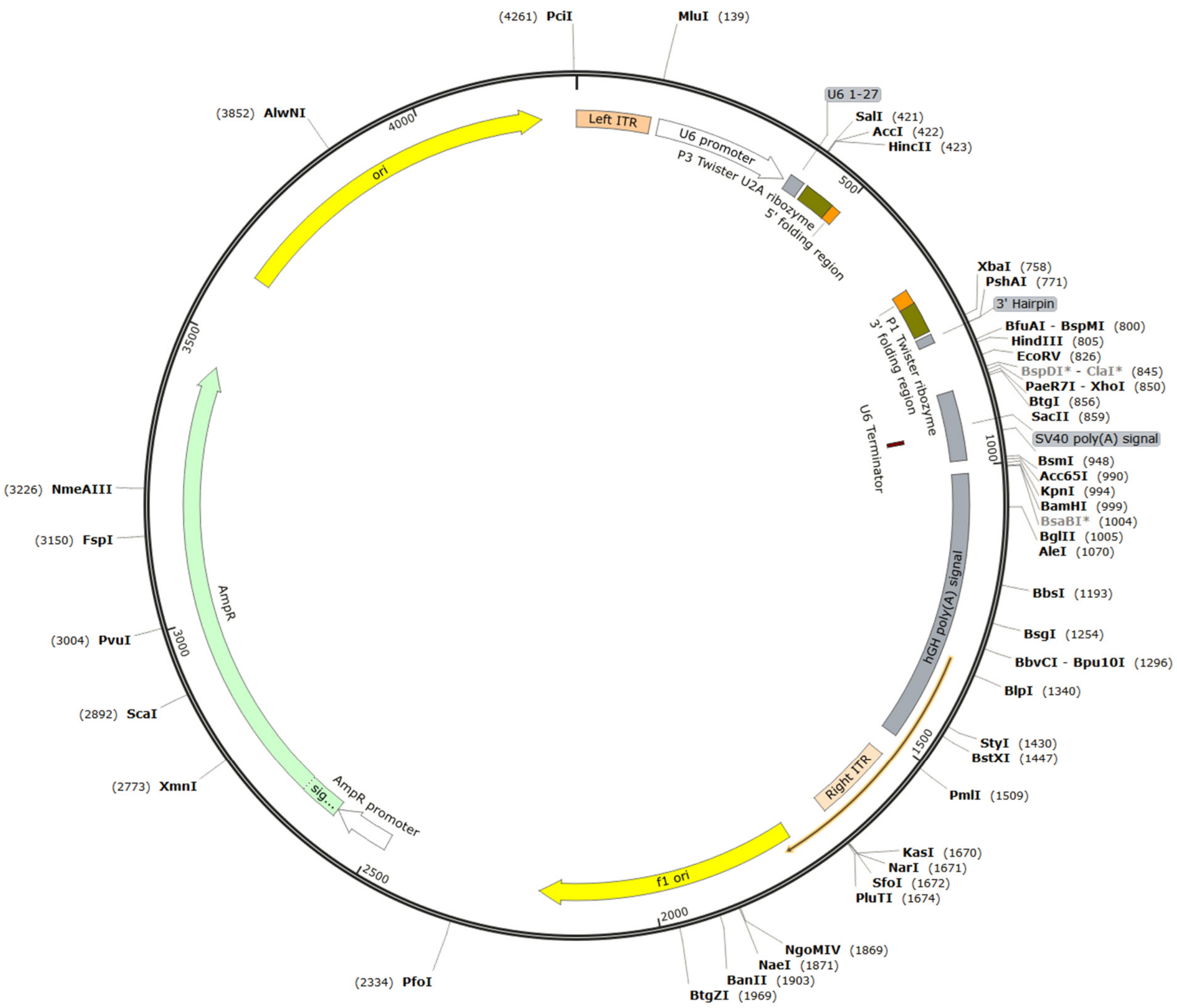

1 CCTGCAGGCA GCTGCGCGCT CGCTCGCTCA CTGAGGCCGC CCGGGCGTCG GGCGACCTTT
61 GGTCGCCCGG CCTCAGTGAG CGAGCGAGCG CGCAGAGAGG GAGTGGCCAA CTCCATCACT
121 AGGGGTTCCT GCGGCCGCAC GCGTGAGGGC CTATTTCCCA TGATTCCTTC ATATTTGCAT
181 ATACGATACA AGGCTGTTAG AGAGATAATT AGAATTAATT TGACTGTAAA CACAAAGATA
241 TTAGTACAAA ATACGTGACG TAGAAAGTAA TAATTTCTTG GGTAGTTTGC AGTTTTAAAA
301 TTATGTTTTA AAATGGACTA TCATATGCTT ACCGTAACTT GAAAGTATTT CGATTTCTTG
361 GCTTTATATA TCTTGTGGAA AGGACGAAAC ACCGTGCTCG CTTCGGCAGC ACATATACTA
421 GTCGACGCCA TCAGTCGCCG GTCCCAAGCC CGGATAAAAT GGGAGGGGGC GGGAAACCGC
481 CTAACCATGC CGACTGATGG CAGCATGTTG TTCTCGTCTC CTCGACACCT CGTGTCCAAC
541 CTTCAGGCAA AAAGTCATCA TGCATAGGTC CCCAAACACG GATGATGGAG CGCCGCAAAG
601 ATCAAATATA CATCGTACTA AATCCAGCAG ATCGGCCAGA CTAAAAAGGT GTCGAGAAGA
661 GGAGAACAAT ATCTTTCTGC CATCAGTCGG CGTGGACTGT AGAACACTGC CAATGCCGGT
721 CCCAAGCCCG GATAAAAGTG GAGGGTACAG TCCACGCTCT AGAGCGGACT TCGGTCCGCT
781 TTTTACTAGG ACCTGCAGGC ATGCAAGCTT GACGTCGGTT ACCGATATCC ATATGGCGGC
841 CGCATCGATC TCGAGCCGCG GACTAGTAAC TTGTTTATTG CAGCTTATAA TGGTTACAAA
901 TAAAGCAATA GCATCACAAA TTTCACAAAT AAAGCATTTT TTTCACTGCA TTCTAGTTGT
961 GGTTTGTCCA AACTCATCAA TGTATCTTAG GTACCTTTGG ATCCAGATCT ACGGGTGGCA
1021 TCCCTGTGAC CCCTCCCCAG TGCCTCTCCT GGCCCTGGAA GTTGCCACTC CAGTGCCCAC
1081 CAGCCTTGTC CTAATAAAAT TAAGTTGCAT CATTTTGTCT GACTAGGTGT CCTTCTATAA

1141 TATTATGGGG TGGAGGGGGG TGGTATGGAG CAAGGGGCAA GTTGGGAAGA CAACCTGTAG
1201 GGCCTGCGGG GTCTATTGGG AACCAAGCTG GAGTGCAGTG GCACAATCTT GGCTCACTGC
1261 AATCTCCGCC TCCTGGGTTC AAGCGATTCT CCTGCCTCAG CCTCCCGAGT TGTTGGGATT
1321 CCAGGCATGC ATGACCAGGC TCAGCTAATT TTTGTTTTTT TGGTAGAGAC GGGGTTTCAC
1381 CATATTGGCC AGGCTGGTCT CCAACTCCTA ATCTCAGGTG ATCTACCCAC CTTGGCCTCC
1441 CAAATTGCTG GGATTACAGG CGTGAACCAC TGCTCCCTTC CCTGTCCTTC TGATTTTGTA
1501 GGTAACCACG TGCGGACCGA GCGGCCGCAG GAACCCCTAG TGATGGAGTT GGCCACTCCC
1561 TCTCTGCGCG CTCGCTCGCT CACTGAGGCC GGGCGACCAA AGGTCGCCCG ACGCCCGGGC
1621 TTTGCCCGGG CGGCCTCAGT GAGCGAGCGA GCGCGCAGCT GCCTGCAGGG GCGCCTGATG
1681 CGGTATTTTC TCCTTACGCA TCTGTGCGGT ATTTCACACC GCATACGTCA AAGCAACCAT
1741 AGTACGCGCC CTGTAGCGGC GCATTAAGCG CGGCGGGTGT GGTGGTTACG CGCAGCGTGA
1801 CCGCTACACT TGCCAGCGCC CTAGCGCCCG CTCCTTTCGC TTTCTTCCCT TCCTTTCTCG
1861 CCACGTTCGC CGGCTTTCCC CGTCAAGCTC TAAATCGGGG GCTCCCTTTA GGGTTCCGAT
1921 TTAGTGCTTT ACGGCACCTC GACCCCAAAA AACTTGATTT GGGTGATGGT TCACGTAGTG
1981 GGCCATCGCC CTGATAGACG GTTTTTCGCC CTTTGACGTT GGAGTCCACG TTCTTTAATA
2041 GTGGACTCTT GTTCCAAACT GGAACAACAC TCAACCCTAT CTCGGGCTAT TCTTTTGATT
2101 TATAAGGGAT TTTGCCGATT TCGGCCTATT GGTTAAAAAA TGAGCTGATT TAACAAAAAT
2161 TTAACGCGAA TTTTAACAAA ATATTAACGT TTACAATTTT ATGGTGCACT CTCAGTACAA
2221 TCTGCTCTGA TGCCGCATAG TTAAGCCAGC CCCGACACCC GCCAACACCC GCTGACGCGC
2281 CCTGACGGGC TTGTCTGCTC CCGGCATCCG CTTACAGACA AGCTGTGACC GTCTCCGGGA
2341 GCTGCATGTG TCAGAGGTTT TCACCGTCAT CACCGAAACG CGCGAGACGA AAGGGCCTCG
2401 TGATACGCCT ATTTTTATAG GTTAATGTCA TGATAATAAT GGTTTCTTAG ACGTCAGGTG
2461 GCACTTTTCG GGGAAATGTG CGCGGAACCC CTATTTGTTT ATTTTTCTAA ATACATTCAA
2521 ATATGTATCC GCTCATGAGA CAATAACCCT GATAAATGCT TCAATAATAT TGAAAAAGGA
2581 AGAGTATGAG TATTCAACAT TTCCGTGTCG CCCTTATTCC CTTTTTTGCG GCATTTTGCC
2641 TTCCTGTTTT TGCTCACCCA GAAACGCTGG TGAAAGTAAA AGATGCTGAA GATCAGTTGG
2701 GTGCACGAGT GGGTTACATC GAACTGGATC TCAACAGCGG TAAGATCCTT GAGAGTTTTC
2761 GCCCCGAAGA ACGTTTTCCA ATGATGAGCA CTTTTAAAGT TCTGCTATGT GGCGCGGTAT
2821 TATCCCGTAT TGACGCCGGG CAAGAGCAAC TCGGTCGCCG CATACACTAT TCTCAGAATG
2881 ACTTGGTTGA GTACTCACCA GTCACAGAAA AGCATCTTAC GGATGGCATG ACAGTAAGAG
2941 AATTATGCAG TGCTGCCATA ACCATGAGTG ATAACACTGC GGCCAACTTA CTTCTGACAA
3001 CGATCGGAGG ACCGAAGGAG CTAACCGCTT TTTTGCACAA CATGGGGGAT CATGTAACTC
3061 GCCTTGATCG TTGGGAACCG GAGCTGAATG AAGCCATACC AAACGACGAG CGTGACACCA
3121 CGATGCCTGT AGCAATGGCA ACAACGTTGC GCAAACTATT AACTGGCGAA CTACTTACTC
3181 TAGCTTCCCG GCAACAATTA ATAGACTGGA TGGAGGCGGA TAAAGTTGCA GGACCACTTC
3241 TGCGCTCGGC CCTTCCGGCT GGCTGGTTTA TTGCTGATAA ATCTGGAGCC GGTGAGCGTG
3301 GGTCTCGCGG TATCATTGCA GCACTGGGGC CAGATGGTAA GCCCTCCCGT ATCGTAGTTA
3361 TCTACACGAC GGGGAGTCAG GCAACTATGG ATGAACGAAA TAGACAGATC GCTGAGATAG
3421 GTGCCTCACT GATTAAGCAT TGGTAACTGT CAGACCAAGT TTACTCATAT ATACTTTAGA
3481 TTGATTTAAA ACTTCATTTT TAATTTAAAA GGATCTAGGT GAAGATCCTT TTTGATAATC
3541 TCATGACCAA AATCCCTTAA CGTGAGTTTT CGTTCCACTG AGCGTCAGAC CCCGTAGAAA
3601 AGATCAAAGG ATCTTCTTGA GATCCTTTTT TTCTGCGCGT AATCTGCTGC TTGCAAACAA
3661 AAAAACCACC GCTACCAGCG GTGGTTTGTT TGCCGGATCA AGAGCTACCA ACTCTTTTTC
3721 CGAAGGTAAC TGGCTTCAGC AGAGCGCAGA TACCAAATAC TGTCCTTCTA GTGTAGCCGT
3781 AGTTAGGCCA CCACTTCAAG AACTCTGTAG CACCGCCTAC ATACCTCGCT CTGCTAATCC
3841 TGTTACCAGT GGCTGCTGCC AGTGGCGATA AGTCGTGTCT TACCGGGTTG GACTCAAGAC
3901 GATAGTTACC GGATAAGGCG CAGCGGTCGG GCTGAACGGG GGGTTCGTGC ACACAGCCCA
3961 GCTTGGAGCG AACGACCTAC ACCGAACTGA GATACCTACA GCGTGAGCTA TGAGAAAGCG
4021 CCACGCTTCC CGAAGGGAGA AAGGCGGACA GGTATCCGGT AAGCGGCAGG GTCGGAACAG
4081 GAGAGCGCAC GAGGGAGCTT CCAGGGGGAA ACGCCTGGTA TCTTTATAGT CCTGTCGGGT
4141 TTCGCCACCT CTGACTTGAG CGTCGATTTT TGTGATGCTC GTCAGGGGGG CGGAGCCTAT
4201 GGAAAAACGC CAGCAACGCG GCCTTTTTAC GGTTCCTGGC CTTTTGCTGG CCTTTTGCTC
4261 ACATGT

Plasmid-map and sequence of **BMPR2_W298X_cDNA_pcDNA3.1 (pTS1160):**

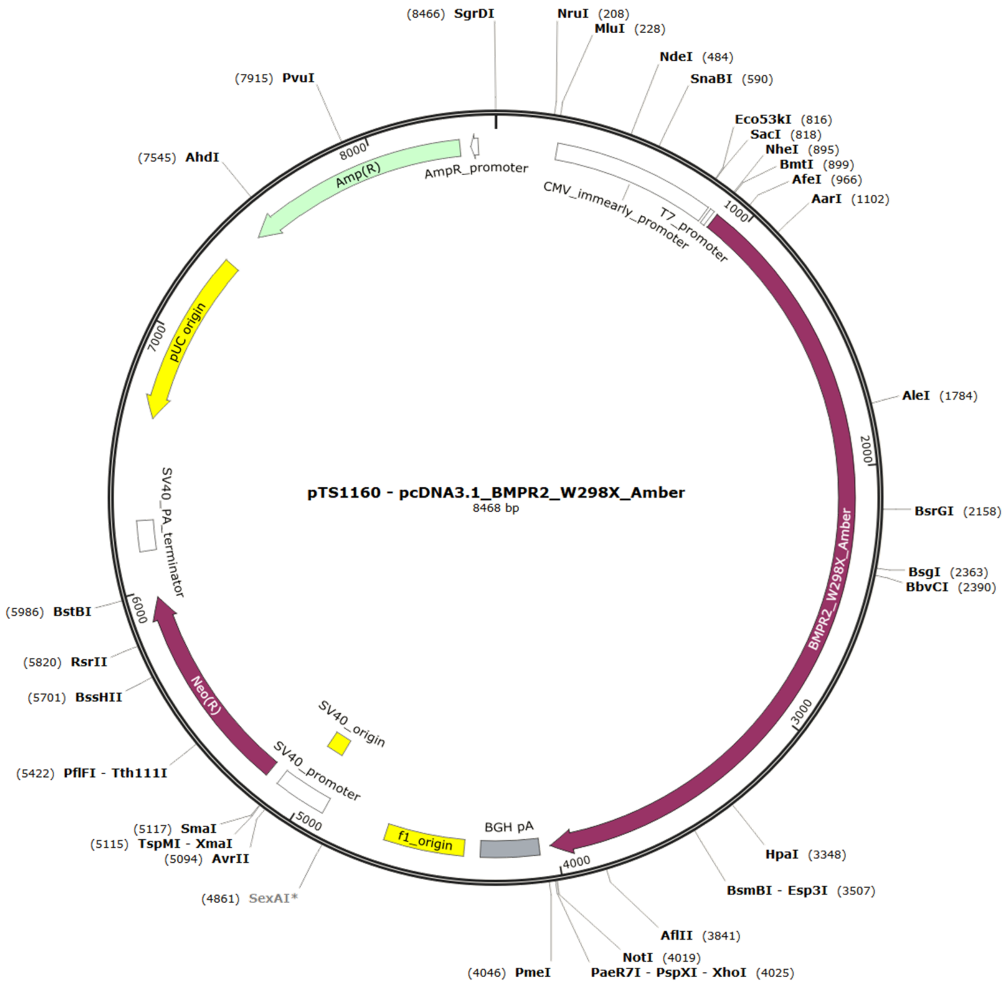


```
1     GACGGATCGG GAGATCTCCC GATCCCCTAT GGTGCACTCT CAGTACAATC TGCTCTGATG
61    CCGCATAGTT AAGCCAGTAT CTGCTCCCTG CTTGTGTGTT GGAGGTCGCT GAGTAGTGCG
121   CGAGCAAAAT TTAAGCTACA ACAAGGCAAG GCTTGACCGA CAATTGCATG AAGAATCTGC
181   TTAGGGTTAG GCGTTTTGCG CTGCTTCGCG ATGTACGGGC CAGATATACG CGTTGACATT
241   GATTATTGAC TAGTTATTAA TAGTAATCAA TTACGGGGTC ATTAGTTCAT AGCCCATATA
301   TGGAGTTCCG CGTTACATAA CTTACGGTAA ATGGCCCGCC TGGCTGACCG CCCAACGACC
361   CCCGCCCATT GACGTCAATA ATGACGTATG TTCCCATAGT AACGCCAATA GGGACTTTCC
421   ATTGACGTCA ATGGGTGGAG TATTTACGGT AAACTGCCCA CTTGGCAGTA CATCAAGTGT
481   ATCATATGCC AAGTACGCCC CCTATTGACG TCAATGACGG TAAATGGCCC GCCTGGCATT
541   ATGCCCAGTA CATGACCTTA TGGGACTTTC CTACTTGGCA GTACATCTAC GTATTAGTCA
601   TCGCTATTAC CATGGTGATG CGGTTTTGGC AGTACATCAA TGGGCGTGGA TAGCGGTTTG
661   ACTCACGGGG ATTTCCAAGT CTCCACCCCA TTGACGTCAA TGGGAGTTTG TTTTGGCACC
721   AAAATCAACG GGACTTTCCA AAATGTCGTA ACAACTCCGC CCCATTGACG CAAATGGGCG
781   GTAGGCGTGT ACGGTGGGAG GTCTATATAA GCAGAGCTCT CTGGCTAACT AGAGAACCCA
841   CTGCTTACTG GCTTATCGAA ATTAATACGA CTCACTATAG GGAGACCCAA GCTGGCTAGC
901   ATGACTTCCT CGCTGCAGCG GCCCTGGCGG GTGCCCTGGC TACCATGGAC CATCCTGCTG
961   GTCAGCGCTG CGGCTGCTTC GCAGAATCAA GAACGGCTAT GTGCGTTTAA AGATCCGTAT
1021  CAGCAAGACC TTGGGATAGG TGAGAGTAGA ATCTCTCATG AAAATGGGAC AATATTATGC
1081  TCGAAAGGTA GCACCTGCTA TGGCCTTTGG GAGAAATCAA AAGGGGACAT AAATCTTGTA
```

1141 AAACAAGGAT GTTGGTCTCA CATTGGAGAT CCCCAAGAGT GTCACTATGA AGAATGTGTA
1201 GTAACTACCA CTCCTCCCTC AATTCAGAAT GGAACATACC GTTTCTGCTG TTGTAGCACA
1261 GATTTATGTA ATGTCAACTT TACTGAGAAT TTTCCACCTC CTGACACAAC ACCACTCAGT
1321 CCACCTCATT CATTTAACCG AGATGAGACA ATAATCATTG CTTTGGCATC AGTCTCTGTA
1381 TTAGCTGTTT TGATAGTTGC CTTATGCTTT GGATACAGAA TGTTGACAGG AGACCGTAAA
1441 CAAGGTCTTC ACAGTATGAA CATGATGGAG GCAGCAGCAT CCGAACCCTC TCTTGATCTA
1501 GATAATCTGA AACTGTTGGA GCTGATTGGC CGAGGTCGAT ATGGAGCAGT ATATAAAGGC
1561 TCCTTGGATG AGCGTCCAGT TGCTGTAAAA GTGTTTTCCT TTGCAAACCG TCAGAATTTT
1621 ATCAACGAAA AGAACATTTA CAGAGTGCCT TTGATGGAAC ATGACAACAT TGCCCGCTTT
1681 ATAGTTGGAG ATGAGAGAGT CACTGCAGAT GGACGCATGG AATATTTGCT TGTGATGGAG
1741 TACTATCCCA ATGGATCTTT ATGCAAGTAT TTAAGTCTCC ACACAAGTGA CTAGGTAAGC
1801 TCTTGCCGTC TTGCTCATTC TGTTACTAGA GGACTGGCTT ATCTTCACAC AGAATTACCA
1861 CGAGGAGATC ATTATAAACC TGCAATTTCC CATCGAGATT TAAACAGCAG AAATGTCCTA
1921 GTGAAAAATG ATGGAACCTG TGTTATTAGT GACTTTGGAC TGTCCATGAG GCTGACTGGA
1981 AATAGACTGG TGCGCCCAGG GGAGGAAGAT AATGCAGCCA TAAGCGAGGT TGGCACTATC
2041 AGATATATGG CACCAGAAGT GCTAGAAGGA GCTGTGAACT TGAGGGACTG TGAATCAGCT
2101 TTGAAACAAG TAGACATGTA TGCTCTTGGA CTAATCTATT GGGAGATATT TATGAGATGT
2161 ACAGACCTCT TCCCAGGGGA ATCCGTACCA GAGTACCAGA TGGCTTTTCA GACAGAGGTT
2221 GGAAACCATC CCACTTTTGA GGATATGCAG GTTCTCGTGT CTAGGGAAAA ACAGAGACCC
2281 AAGTTCCCAG AAGCCTGGAA AGAAAATAGC CTGGCAGTGA GGTCACTCAA GGAGACAATC
2341 GAAGACTGTT GGGACCAGGA TGCAGAGGCT CGGCTTACTG CACAGTGTGC TGAGGAAAGG
2401 ATGGCTGAAC TTATGATGAT TTGGGAAAGA AACAAATCTG TGAGCCCAAC AGTCAATCCA
2461 ATGTCTACTG CTATGCAGAA TGAACGCAAC CTGTCACATA ATAGGCGTGT GCCAAAAATT
2521 GGTCCTTATC CAGATTATTC TTCCTCCTCA TACATTGAAG ACTCTATCCA TCATACTGAC
2581 AGCATCGTGA AGAATATTTC CTCTGAGCAT TCTATGTCCA GCACACCTTT GACTATAGGG
2641 GAAAAAAACC GAAATTCAAT TAACTATGAA CGACAGCAAG CACAAGCTCG AATCCCCAGC
2701 CCTGAAACAA GTGTCACCAG CCTCTCCACC AACACAACAA CCACAAACAC CACAGGACTC
2761 ACGCCAAGTA CTGGCATGAC TACTATATCT GAGATGCCAT ACCCAGATGA AACAAATCTG
2821 CATACCACAA ATGTTGCACA GTCAATTGGG CCAACCCCTG TCTGCTTACA GCTGACAGAA
2881 GAAGACTTGG AAACCAACAA GCTAGACCCA AAAGAAGTTG ATAAGAACCT CAAGGAAAGC
2941 TCTGATGAGA ATCTCATGGA GCACTCTCTT AAACAGTTCA GTGGCCCAGA CCCACTGAGC
3001 AGTACTAGTT CTAGCTTGCT TTACCCACTC ATAAAACTTG CAGTAGAAGC AACTGGACAG
3061 CAGGACTTCA CACAGACTGC AAATGGCCAA GCATGTTTGA TTCCTGATGT TCTGCCTACT
3121 CAGATCTATC CTCTCCCCAA GCAGCAGAAC CTTCCCAAGA GACCTACTAG TTTGCCTTTG
3181 AACACCAAAA ATTCAACAAA AGAGCCCCGG CTAAAATTTG GCAGCAAGCA CAAATCAAAC
3241 TTGAAACAAG TCGAAACTGG AGTTGCCAAG ATGAATACAA TCAATGCAGC AGAACCTCAT
3301 GTGGTGACAG TCACCATGAA TGGTGTGGCA GGTAGAAACC ACAGTGTTAA CTCCCATGCT
3361 GCCACAACCC AATATGCCAA TGGGACAGTA CTATCTGGCC AAACAACCAA CATAGTGACA
3421 CATAGGGCCC AAGAAATGTT GCAGAATCAG TTTATTGGTG AGGACACCCG GCTGAATATT
3481 AATTCCAGTC CTGATGAGCA TGAGCCTTTA CTGAGACGAG AGCAACAAGC TGGCCATGAT
3541 GAAGGTGTTC TGGATCGTCT TGTGGACAGG AGGGAACGGC CACTAGAAGG TGGCCGAACT
3601 AATTCCAATA ACAACAACAG CAATCCATGT TCAGAACAAG ATGTTCTTGC ACAGGGTGTT
3661 CCAAGCACAG CAGCAGATCC TGGGCCATCA AAGCCCAGAA GAGCACAGAG GCCTAATTCT
3721 CTGGATCTTT CAGCCACAAA TGTCCTGGAT GGCAGCAGTA TACAGATAGG TGAGTCAACA
3781 CAAGATGGCA AATCAGGATC AGGTGAAAAG ATCAAGAAAC GTGTGAAAAC TCCCTATTCT
3841 CTTAAGCGGT GGCGCCCCTC CACCTGGGTC ATCTCCACTG AATCGCTGGA CTGTGAAGTC
3901 AACAATAATG GCAGTAACAG GGCAGTTCAT TCCAAATCCA GCACTGCTGT TTACCTTGCA
3961 GAAGGAGGCA CTGCTACAAC CATGGTGTCT AAAGATATAG GAATGAACTG TCTGTGAGCG
4021 GCCGCTCGAG TCTAGAGGGC CCGTTTAAAC CCGCTGATCA GCCTCGACTG TGCCTTCTAG
4081 TTGCCAGCCA TCTGTTGTTT GCCCCTCCCC CGTGCCTTCC TTGACCCTGG AAGGTGCCAC
4141 TCCCACTGTC CTTTCCTAAT AAAATGAGGA AATTGCATCG CATTGTCTGA GTAGGTGTCA
4201 TTCTATTCTG GGGGGTGGGG TGGGGCAGGA CAGCAAGGGG GAGGATTGGG AAGACAATAG
4261 CAGGCATGCT GGGGATGCGG TGGGCTCTAT GGCTTCTGAG GCGGAAAGAA CCAGCTGGGG
4321 CTCTAGGGGG TATCCCCACG CGCCCTGTAG CGGCGCATTA AGCGCGGCGG GTGTGGTGGT
4381 TACGCGCAGC GTGACCGCTA CACTTGCCAG CGCCCTAGCG CCCGCTCCTT TCGCTTTCTT
4441 CCCTTCCTTT CTCGCCACGT TCGCCGGCTT TCCCCGTCAA GCTCTAAATC GGGGGCTCCC
4501 TTTAGGGTTC CGATTTAGTG CTTTACGGCA CCTCGACCCC AAAAAACTTG ATTAGGGTGA

4561 TGGTTCACGT AGTGGGCCAT CGCCCTGATA GACGGTTTTT CGCCCTTTGA CGTTGGAGTC
4621 CACGTTCTTT AATAGTGGAC TCTTGTTCCA AACTGGAACA ACACTCAACC CTATCTCGGT
4681 CTATTCTTTT GATTTATAAG GGATTTTGCC GATTTCGGCC TATTGGTTAA AAAATGAGCT
4741 GATTTAACAA AAATTTAACG CGAATTAATT CTGTGGAATG TGTGTCAGTT AGGGTGTGGA
4801 AAGTCCCCAG GCTCCCCAGC AGGCAGAAGT ATGCAAAGCA TGCATCTCAA TTAGTCAGCA
4861 ACCAGGTGTG GAAAGTCCCC AGGCTCCCCA GCAGGCAGAA GTATGCAAAG CATGCATCTC
4921 AATTAGTCAG CAACCATAGT CCCGCCCCTA ACTCCGCCCA TCCCGCCCCT AACTCCGCCC
4981 AGTTCCGCCC ATTCTCCGCC CCATGGCTGA CTAATTTTTT TTATTTATGC AGAGGCCGAG
5041 GCCGCCTCTG CCTCTGAGCT ATTCCAGAAG TAGTGAGGAG GCTTTTTTGG AGGCCTAGGC
5101 TTTTGCAAAA AGCTCCCGGG AGCTTGTATA TCCATTTTCG GATCTGATCA AGAGACAGGA
5161 TGAGGATCGT TTCGCATGAT TGAACAAGAT GGATTGCACG CAGGTTCTCC GGCCGCTTGG
5221 GTGGAGAGGC TATTCGGCTA TGACTGGGCA CAACAGACAA TCGGCTGCTC TGATGCCGCC
5281 GTGTTCCGGC TGTCAGCGCA GGGGCGCCCG GTTCTTTTTG TCAAGACCGA CCTGTCCGGT
5341 GCCCTGAATG AACTGCAGGA CGAGGCAGCG CGGCTATCGT GGCTGGCCAC GACGGGCGTT
5401 CCTTGCGCAG CTGTGCTCGA CGTTGTCACT GAAGCGGGAA GGGACTGGCT GCTATTGGGC
5461 GAAGTGCCGG GGCAGGATCT CCTGTCATCT CACCTTGCTC CTGCCGAGAA AGTATCCATC
5521 ATGGCTGATG CAATGCGGCG GCTGCATACG CTTGATCCGG CTACCTGCCC ATTCGACCAC
5581 CAAGCGAAAC ATCGCATCGA GCGAGCACGT ACTCGGATGG AAGCCGGTCT TGTCGATCAG
5641 GATGATCTGG ACGAAGAGCA TCAGGGGCTC GCGCCAGCCG AACTGTTCGC CAGGCTCAAG
5701 GCGCGCATGC CCGACGGCGA GGATCTCGTC GTGACCCATG GCGATGCCTG CTTGCCGAAT
5761 ATCATGGTGG AAAATGGCCG CTTTTCTGGA TTCATCGACT GTGGCCGGCT GGGTGTGGCG
5821 GACCGCTATC AGGACATAGC GTTGGCTACC CGTGATATTG CTGAAGAGCT TGGCGGCGAA
5881 TGGGCTGACC GCTTCCTCGT GCTTTACGGT ATCGCCGCTC CCGATTCGCA GCGCATCGCC
5941 TTCTATCGCC TTCTTGACGA GTTCTTCTGA GCGGGACTCT GGGGTTCGAA ATGACCGACC
6001 AAGCGACGCC CAACCTGCCA TCACGAGATT TCGATTCCAC CGCCGCCTTC TATGAAAGGT
6061 TGGGCTTCGG AATCGTTTTC CGGGACGCCG GCTGGATGAT CCTCCAGCGC GGGGATCTCA
6121 TGCTGGAGTT CTTCGCCCAC CCCAACTTGT TTATTGCAGC TTATAATGGT TACAAATAAA
6181 GCAATAGCAT CACAAATTTC ACAAATAAAG CATTTTTTTC ACTGCATTCT AGTTGTGGTT
6241 TGTCCAAACT CATCAATGTA TCTTATCATG TCTGTATACC GTCGACCTCT AGCTAGAGCT
6301 TGGCGTAATC ATGGTCATAG CTGTTTCCTG TGTGAAATTG TTATCCGCTC ACAATTCCAC
6361 ACAACATACG AGCCGGAAGC ATAAAGTGTA AAGCCTGGGG TGCCTAATGA GTGAGCTAAC
6421 TCACATTAAT TGCGTTGCGC TCACTGCCCG CTTTCCAGTC GGGAAACCTG TCGTGCCAGC
6481 TGCATTAATG AATCGGCCAA CGCGCGGGGA GAGGCGGTTT GCGTATTGGG CGCTCTTCCG
6541 CTTCCTCGCT CACTGACTCG CTGCGCTCGG TCGTTCGGCT GCGGCGAGCG GTATCAGCTC
6601 ACTCAAAGGC GGTAATACGG TTATCCACAG AATCAGGGGA TAACGCAGGA AAGAACATGT
6661 GAGCAAAAGG CCAGCAAAAG GCCAGGAACC GTAAAAAGGC CGCGTTGCTG GCGTTTTTCC
6721 ATAGGCTCCG CCCCCCTGAC GAGCATCACA AAAATCGACG CTCAAGTCAG AGGTGGCGAA
6781 ACCCGACAGG ACTATAAAGA TACCAGGCGT TTCCCCCTGG AAGCTCCCTC GTGCGCTCTC
6841 CTGTTCCGAC CCTGCCGCTT ACCGGATACC TGTCCGCCTT TCTCCCTTCG GGAAGCGTGG
6901 CGCTTTCTCA TAGCTCACGC TGTAGGTATC TCAGTTCGGT GTAGGTCGTT CGCTCCAAGC
6961 TGGGCTGTGT GCACGAACCC CCCGTTCAGC CCGACCGCTG CGCCTTATCC GGTAACTATC
7021 GTCTTGAGTC CAACCCGGTA AGACACGACT TATCGCCACT GGCAGCAGCC ACTGGTAACA
7081 GGATTAGCAG AGCGAGGTAT GTAGGCGGTG CTACAGAGTT CTTGAAGTGG TGGCCTAACT
7141 ACGGCTACAC TAGAAGAACA GTATTTGGTA TCTGCGCTCT GCTGAAGCCA GTTACCTTCG
7201 GAAAAAGAGT TGGTAGCTCT TGATCCGGCA AACAAACCAC CGCTGGTAGC GGTTTTTTTG
7261 TTTGCAAGCA GCAGATTACG CGCAGAAAAA AAGGATCTCA AGAAGATCCT TTGATCTTTT
7321 CTACGGGGTC TGACGCTCAG TGGAACGAAA ACTCACGTTA AGGGATTTTG GTCATGAGAT
7381 TATCAAAAAG GATCTTCACC TAGATCCTTT TAAATTAAAA ATGAAGTTTT AAATCAATCT
7441 AAAGTATATA TGAGTAAACT TGGTCTGACA GTTACCAATG CTTAATCAGT GAGGCACCTA
7501 TCTCAGCGAT CTGTCTATTT CGTTCATCCA TAGTTGCCTG ACTCCCCGTC GTGTAGATAA
7561 CTACGATACG GGAGGGCTTA CCATCTGGCC CCAGTGCTGC AATGATACCG CGAGACCCAC
7621 GCTCACCGGC TCCAGATTTA TCAGCAATAA ACCAGCCAGC CGGAAGGGCC GAGCGCAGAA
7681 GTGGTCCTGC AACTTTATCC GCCTCCATCC AGTCTATTAA TTGTTGCCGG GAAGCTAGAG
7741 TAAGTAGTTC GCCAGTTAAT AGTTTGCGCA ACGTTGTTGC CATTGCTACA GGCATCGTGG
7801 TGTCACGCTC GTCGTTTGGT ATGGCTTCAT TCAGCTCCGG TTCCCAACGA TCAAGGCGAG
7861 TTACATGATC CCCCATGTTG TGCAAAAAAG CGGTTAGCTC CTTCGGTCCT CCGATCGTTG
7921 TCAGAAGTAA GTTGGCCGCA GTGTTATCAC TCATGGTTAT GGCAGCACTG CATAATTCTC

7981 TTACTGTCAT GCCATCCGTA AGATGCTTTT CTGTGACTGG TGAGTACTCA ACCAAGTCAT
8041 TCTGAGAATA GTGTATGCGG CGACCGAGTT GCTCTTGCCC GGCGTCAATA CGGGATAATA
8101 CCGCGCCACA TAGCAGAACT TTAAAAGTGC TCATCATTGG AAAACGTTCT TCGGGGCGAA
8161 AACTCTCAAG GATCTTACCG CTGTTGAGAT CCAGTTCGAT GTAACCCACT CGTGCACCCA
8221 ACTGATCTTC AGCATCTTTT ACTTTCACCA GCGTTTCTGG GTGAGCAAAA ACAGGAAGGC
8281 AAAATGCCGC AAAAAAGGGA ATAAGGGCGA CACGGAAATG TTGAATACTC ATACTCTTCC
8341 TTTTTCAATA TTATTGAAGC ATTTATCAGG GTTATTGTCT CATGAGCGGA TACATATTTG
8401 AATGTATTTA GAAAAATAAA CAAATAGGGG TTCCGCGCAC ATTTCCCCGA AAAGTGCCAC
8461 CTGACGTC

Plasmid-map and sequence of **circ-mMecp2-V27.2.4_gRNA_pAAV_stuffer (pTS2154):**

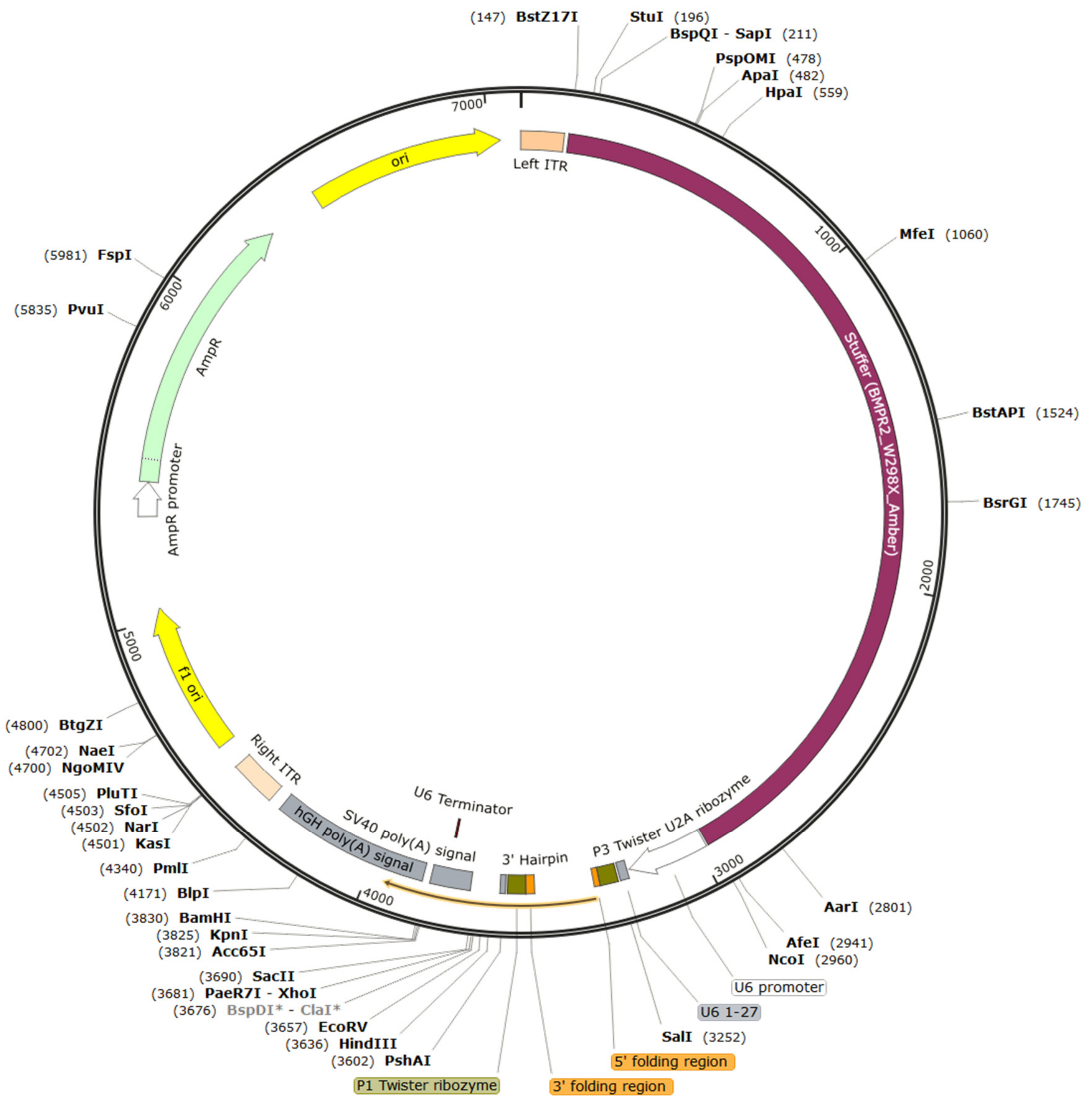


```
1         CCTGCAGGCA GCTGCGCGCT CGCTCGCTCA CTGAGGCCGC CCGGGCGTCG GGCGACCTTT
61        GGTCGCCCGG CCTCAGTGAG CGAGCGAGCG CGCAGAGAGG GAGTGGCCAA CTCCATCACT
121       AGGGGTTCCT GCGGCCGCAC GCGTGTATAC TGCTGCCATC CAGGACATTT GTGGCTGAAA
181       GATCCAGAGA ATTAGGCCTC TGTGCTCTTC TGGGCTTTGA TGGCCCAGGA TCTGCTGCTG
241       TGCTTGGAAC ACCCTGTGCA AGAACATCTT GTTCTGAACA TGGATTGCTG TTGTTGTTAT
301       TGGAATTAGT TCGGCCACCT TCTAGTGGCC GTTCCCTCCT GTCCACAAGA CGATCCAGAA
361       CACCTTCATC ATGGCCAGCT TGTTGCTCTC GTCTCAGTAA AGGCTCATGC TCATCAGGAC
421       TGGAATTAAT ATTCAGCCGG GTGTCCTCAC CAATAAACTG ATTCTGCAAC ATTTCTTGGG
481       CCCTATGTGT CACTATGTTG GTTGTTTGGC CAGATAGTAC TGTCCCATTG GCATATTGGG
541       TTGTGGCAGC ATGGGAGTTA ACACTGTGGT TTCTACCTGC CACACCATTC ATGGTGACTG
601       TCACCACATG AGGTTCTGCT GCATTGATTG TATTCATCTT GGCAACTCCA GTTTCGACTT
661       GTTTCAAGTT TGATTTGTGC TTGCTGCCAA ATTTTAGCCG GGGCTCTTTT GTTGAATTTT
721       TGGTGTTCAA AGGCAAACTA GTAGGTCTCT TGGGAAGGTT CTGCTGCTTG GGGAGAGGAT
781       AGATCTGAGT AGGCAGAACA TCAGGAATCA AACATGCTTG GCCATTTGCA GTCTGTGTGA
841       AGTCCTGCTG TCCAGTTGCT TCTACTGCAA GTTTTATGAG TGGGTAAAGC AAGCTAGAAC
```

901 TAGTACTGCT CAGTGGGTCT GGGCCACTGA ACTGTTTAAG AGAGTGCTCC ATGAGATTCT
961 CATCAGAGCT TTCCTTGAGG TTCTTATCAA CTTCTTTTGG GTCTAGCTTG TTGGTTTCCA
1021 AGTCTTCTTC TGTCAGCTGT AAGCAGACAG GGGTTGGCCC AATTGACTGT GCAACATTTG
1081 TGGTATGCAG ATTTGTTTCA TCTGGGTATG GCATCTCAGA TATAGTAGTC ATGCCAGTAC
1141 TTGGCGTGAG TCCTGTGGTG TTTGTGGTTG TTGTGTTGGT GGAGAGGCTG GTGACACTTG
1201 TTTCAGGGCT GGGGATTCGA GCTTGTGCTT GCTGTCGTTC ATAGTTAATT GAATTTCGGT
1261 TTTTTTCCCC TATAGTCAAA GGTGTGCTGG ACATAGAATG CTCAGAGGAA ATATTCTTCA
1321 CGATGCTGTC AGTATGATGG ATAGAGTCTT CAATGTATGA GGAGGAAGAA TAATCTGGAT
1381 AAGGACCAAT TTTTGGCACA CGCCTATTAT GTGACAGGTT GCGTTCATTC TGCATAGCAG
1441 TAGACATTGG ATTGACTGTT GGGCTCACAG ATTTGTTTCT TTCCCAAATC ATCATAAGTT
1501 CAGCCATCCT TTCCTCAGCA CACTGTGCAG TAAGCCGAGC CTCTGCATCC TGGTCCCAAC
1561 AGTCTTCGAT TGTCTCCTTG AGTGACCTCA CTGCCAGGCT ATTTTCTTTC CAGGCTTCTG
1621 GGAACTTGGG TCTCTGTTTT TCCCTAGACA CGAGAACCTG CATATCCTCA AAAGTGGGAT
1681 GGTTTCCAAC CTCTGTCTGA AAAGCCATCT GGTACTCTGG TACGGATTCC CCTGGGAAGA
1741 GGTCTGTACA TCTCATAAAT ATCTCCCAAT AGATTAGTCC AAGAGCATAC ATGTCTACTT
1801 GTTTCAAAGC TGATTCACAG TCCCTCAAGT TCACAGCTCC TTCTAGCACT TCTGGTGCCA
1861 TATATCTGAT AGTGCCAACC TCGCTTATGG CTGCATTATC TTCCTCCCCT GGGCGCACCA
1921 GTCTATTTCC AGTCAGCCTC ATGGACAGTC CAAAGTCACT AATAACACAG GTTCCATCAT
1981 TTTTCACTAG GACATTTCTG CTGTTTAAAT CTCGATGGGA AATTGCAGGT TTATAATGAT
2041 CTCCTCGTGG TAATTCTGTG TGAAGATAAG CCAGTCCTCT AGTAACAGAA TGAGCAAGAC
2101 GGCAAGAGCT TACCTAGTCA CTTGTGTGGA GACTTAAATA CTTGCATAAA GATCCATTGG
2161 GATAGTACTC CATCACAAGC AAATATTCCA TGCGTCCATC TGCAGTGACT CTCTCATCTC
2221 CAACTATAAA GCGGGCAATG TTGTCATGTT CCATCAAAGG CACTCTGTAA ATGTTCTTTT
2281 CGTTGATAAA ATTCTGACGG TTTGCAAAGG AAAACACTTT TACAGCAACT GGACGCTCAT
2341 CCAAGGAGCC TTTATATACT GCTCCATATC GACCTCGGCC AATCAGCTCC AACAGTTTCA
2401 GATTATCTAG ATCAAGAGAG GGTTCGGATG CTGCTGCCTC CATCATGTTC ATACTGTGAA
2461 GACCTTGTTT ACGGTCTCCT GTCAACATTC TGTATCCAAA GCATAAGGCA ACTATCAAAA
2521 CAGCTAATAC AGAGACTGAT GCCAAAGCAA TGATTATTGT CTCATCTCGG TTAAATGAAT
2581 GAGGTGGACT GAGTGGTGTT GTGTCAGGAG GTGGAAAATT CTCAGTAAAG TTGACATTAC
2641 ATAAATCTGT GCTACAACAG CAGAAACGGT ATGTTCCATT CTGAATTGAG GGAGGAGTGG
2701 TAGTTACTAC ACATTCTTCA TAGTGACACT CTTGGGGATC TCCAATGTGA GACCAACATC
2761 CTTGTTTTAC AAGATTTATG TCCCCTTTTG ATTTCTCCCA AAGGCCATAG CAGGTGCTAC
2821 CTTTCGAGCA TAATATTGTC CCATTTTCAT GAGAGATTCT ACTCTCACCT ATCCCAAGGT
2881 CTTGCTGATA CGGATCTTTA AACGCACATA GCCGTTCTTG ATTCTGCGAA GCAGCCGCAG
2941 CGCTGACCAG CAGGATGGTC CATGGTAGCA CGCGTGAGGG CCTATTTCCC ATGATTCCTT
3001 CATATTTGCA TATACGATAC AAGGCTGTTA GAGAGATAAT TAGAATTAAT TTGACTGTAA
3061 ACACAAAGAT ATTAGTACAA AATACGTGAC GTAGAAAGTA ATAATTTCTT GGGTAGTTTG
3121 CAGTTTTAAA ATTATGTTTT AAAATGGACT ATCATATGCT TACCGTAACT TGAAAGTATT
3181 TCGATTTCTT GGCTTTATAT ATCTTGTGGA AAGGACGAAA CACCGTGCTC GCTTCGGCAG
3241 CACATATACT AGTCGACGCC ATCAGTCGCC GGTCCCAAGC CCGGATAAAA TGGGAGGGGG
3301 CGGGAAACCG CCTAACCATG CCGACTGATG GCAGCATGTT GTTCTCGTCT CCTCGACACC
3361 TCGTGTCCAA CCTTCAGGCA AAAAGTCATC ATGCATAGGT CCCCAAACAC GGATGATGGA
3421 GCGCCGCAAA GATCAAATAT ACATCGTACT AAATCCAGCA GATCGGCCAG ACTAAAAAGG
3481 TGTCGAGAAG AGGAGAACAA TATCTTTCTG CCATCAGTCG GCGTGGACTG TAGAACACTG
3541 CCAATGCCGG TCCCAAGCCC GGATAAAAGT GGAGGGTACA GTCCACGCTC TAGAGCGGAC
3601 TTCGGTCCGC TTTTTACTAG GACCTGCAGG CATGCAAGCT TGACGTCGGT TACCGATATC
3661 CATATGGCGG CCGCATCGAT CTCGAGCCGC GGACTAGTAA CTTGTTTATT GCAGCTTATA
3721 ATGGTTACAA ATAAAGCAAT AGCATCACAA ATTTCACAAA TAAAGCATTT TTTTCACTGC
3781 ATTCTAGTTG TGGTTTGTCC AAACTCATCA ATGTATCTTA GGTACCTTTG GATCCAGATC
3841 TACGGGTGGC ATCCCTGTGA CCCCTCCCCA GTGCCTCTCC TGGCCCTGGA AGTTGCCACT
3901 CCAGTGCCCA CCAGCCTTGT CCTAATAAAA TTAAGTTGCA TCATTTTGTC TGACTAGGTG
3961 TCCTTCTATA ATATTATGGG GTGGAGGGGG GTGGTATGGA GCAAGGGGCA AGTTGGGAAG
4021 ACAACCTGTA GGGCCTGCGG GGTCTATTGG GAACCAAGCT GGAGTGCAGT GGCACAATCT
4081 TGGCTCACTG CAATCTCCGC CTCCTGGGTT CAAGCGATTC TCCTGCCTCA GCCTCCCGAG
4141 TTGTTGGGAT TCCAGGCATG CATGACCAGG CTCAGCTAAT TTTGTTTTTT TTGGTAGAGA
4201 CGGGGTTTCA CCATATTGGC CAGGCTGGTC TCCAACTCCT AATCTCAGGT GATCTACCCA
4261 CCTTGGCCTC CCAAATTGCT GGGATTACAG GCGTGAACCA CTGCTCCCTT CCCTGTCCTT

4321 CTGATTTTGT AGGTAACCAC GTGCGGACCG AGCGGCCGCA GGAACCCCTA GTGATGGAGT
4381 TGGCCACTCC CTCTCTGCGC GCTCGCTCGC TCACTGAGGC CGGGCGACCA AAGGTCGCCC
4441 GACGCCCGGG CTTTGCCCGG GCGGCCTCAG TGAGCGAGCG AGCGCGCAGC TGCCTGCAGG
4501 GGCGCCTGAT GCGGTATTTT CTCCTTACGC ATCTGTGCGG TATTTCACAC CGCATACGTC
4561 AAAGCAACCA TAGTACGCGC CCTGTAGCGG CGCATTAAGC GCGGCGGGTG TGGTGGTTAC
4621 GCGCAGCGTG ACCGCTACAC TTGCCAGCGC CCTAGCGCCC GCTCCTTTCG CTTTCTTCCC
4681 TTCCTTTCTC GCCACGTTCG CCGGCTTTCC CCGTCAAGCT CTAAATCGGG GGCTCCCTTT
4741 AGGGTTCCGA TTTAGTGCTT TACGGCACCT CGACCCCAAA AAACTTGATT TGGGTGATGG
4801 TTCACGTAGT GGGCCATCGC CCTGATAGAC GGTTTTTCGC CCTTTGACGT TGGAGTCCAC
4861 GTTCTTTAAT AGTGGACTCT TGTTCCAAAC TGGAACAACA CTCAACCCTA TCTCGGGCTA
4921 TTCTTTTGAT TTATAAGGGA TTTTGCCGAT TTCGGCCTAT TGGTTAAAAA ATGAGCTGAT
4981 TTAACAAAAA TTTAACGCGA ATTTTAACAA AATATTAACG TTTACAATTT TATGGTGCAC
5041 TCTCAGTACA ATCTGCTCTG ATGCCGCATA GTTAAGCCAG CCCCGACACC CGCCAACACC
5101 CGCTGACGCG CCCTGACGGG CTTGTCTGCT CCCGGCATCC GCTTACAGAC AAGCTGTGAC
5161 CGTCTCCGGG AGCTGCATGT GTCAGAGGTT TTCACCGTCA TCACCGAAAC GCGCGAGACG
5221 AAAGGGCCTC GTGATACGCC TATTTTTATA GGTTAATGTC ATGATAATAA TGGTTTCTTA
5281 GACGTCAGGT GGCACTTTTC GGGGAAATGT GCGCGGAACC CCTATTTGTT TATTTTTCTA
5341 AATACATTCA AATATGTATC CGCTCATGAG ACAATAACCC TGATAAATGC TTCAATAATA
5401 TTGAAAAAGG AAGAGTATGA GTATTCAACA TTTCCGTGTC GCCCTTATTC CCTTTTTTGC
5461 GGCATTTTGC CTTCCTGTTT TTGCTCACCC AGAAACGCTG GTGAAAGTAA AAGATGCTGA
5521 AGATCAGTTG GGTGCACGAG TGGGTTACAT CGAACTGGAT CTCAACAGCG GTAAGATCCT
5581 TGAGAGTTTT CGCCCCGAAG AACGTTTTCC AATGATGAGC ACTTTTAAAG TTCTGCTATG
5641 TGGCGCGGTA TTATCCCGTA TTGACGCCGG GCAAGAGCAA CTCGGTCGCC GCATACACTA
5701 TTCTCAGAAT GACTTGGTTG AGTACTCACC AGTCACAGAA AAGCATCTTA CGGATGGCAT
5761 GACAGTAAGA GAATTATGCA GTGCTGCCAT AACCATGAGT GATAACACTG CGGCCAACTT
5821 ACTTCTGACA ACGATCGGAG GACCGAAGGA GCTAACCGCT TTTTTGCACA ACATGGGGGA
5881 TCATGTAACT CGCCTTGATC GTTGGGAACC GGAGCTGAAT GAAGCCATAC CAAACGACGA
5941 GCGTGACACC ACGATGCCTG TAGCAATGGC AACAACGTTG CGCAAACTAT TAACTGGCGA
6001 ACTACTTACT CTAGCTTCCC GGCAACAATT AATAGACTGG ATGGAGGCGG ATAAAGTTGC
6061 AGGACCACTT CTGCGCTCGG CCCTTCCGGC TGGCTGGTTT ATTGCTGATA AATCTGGAGC
6121 CGGTGAGCGT GGGTCTCGCG GTATCATTGC AGCACTGGGG CCAGATGGTA AGCCCTCCCG
6181 TATCGTAGTT ATCTACACGA CGGGGAGTCA GGCAACTATG GATGAACGAA ATAGACAGAT
6241 CGCTGAGATA GGTGCCTCAC TGATTAAGCA TTGGTAACTG TCAGACCAAG TTTACTCATA
6301 TATACTTTAG ATTGATTTAA AACTTCATTT TTAATTTAAA AGGATCTAGG TGAAGATCCT
6361 TTTTGATAAT CTCATGACCA AAATCCCTTA ACGTGAGTTT TCGTTCCACT GAGCGTCAGA
6421 CCCCGTAGAA AAGATCAAAG GATCTTCTTG AGATCCTTTT TTTCTGCGCG TAATCTGCTG
6481 CTTGCAAACA AAAAAACCAC CGCTACCAGC GGTGGTTTGT TTGCCGGATC AAGAGCTACC
6541 AACTCTTTTT CCGAAGGTAA CTGGCTTCAG CAGAGCGCAG ATACCAAATA CTGTCCTTCT
6601 AGTGTAGCCG TAGTTAGGCC ACCACTTCAA GAACTCTGTA GCACCGCCTA CATACCTCGC
6661 TCTGCTAATC CTGTTACCAG TGGCTGCTGC CAGTGGCGAT AAGTCGTGTC TTACCGGGTT
6721 GGACTCAAGA CGATAGTTAC CGGATAAGGC GCAGCGGTCG GGCTGAACGG GGGGTTCGTG
6781 CACACAGCCC AGCTTGGAGC GAACGACCTA CACCGAACTG AGATACCTAC AGCGTGAGCT
6841 ATGAGAAAGC GCCACGCTTC CCGAAGGGAG AAAGGCGGAC AGGTATCCGG TAAGCGGCAG
6901 GGTCGGAACA GGAGAGCGCA CGAGGGAGCT TCCAGGGGGA AACGCCTGGT ATCTTTATAG
6961 TCCTGTCGGG TTTCGCCACC TCTGACTTGA GCGTCGATTT TTGTGATGCT CGTCAGGGGG
7021 GCGGAGCCTA TGGAAAAACG CCAGCAACGC GGCCTTTTTA CGGTTCCTGG CCTTTTGCTG
7081 GCCTTTTGCT CACATGT

Plasmid-map and sequence of the **pcDNA3.1 eGFP vector (pTS58):**

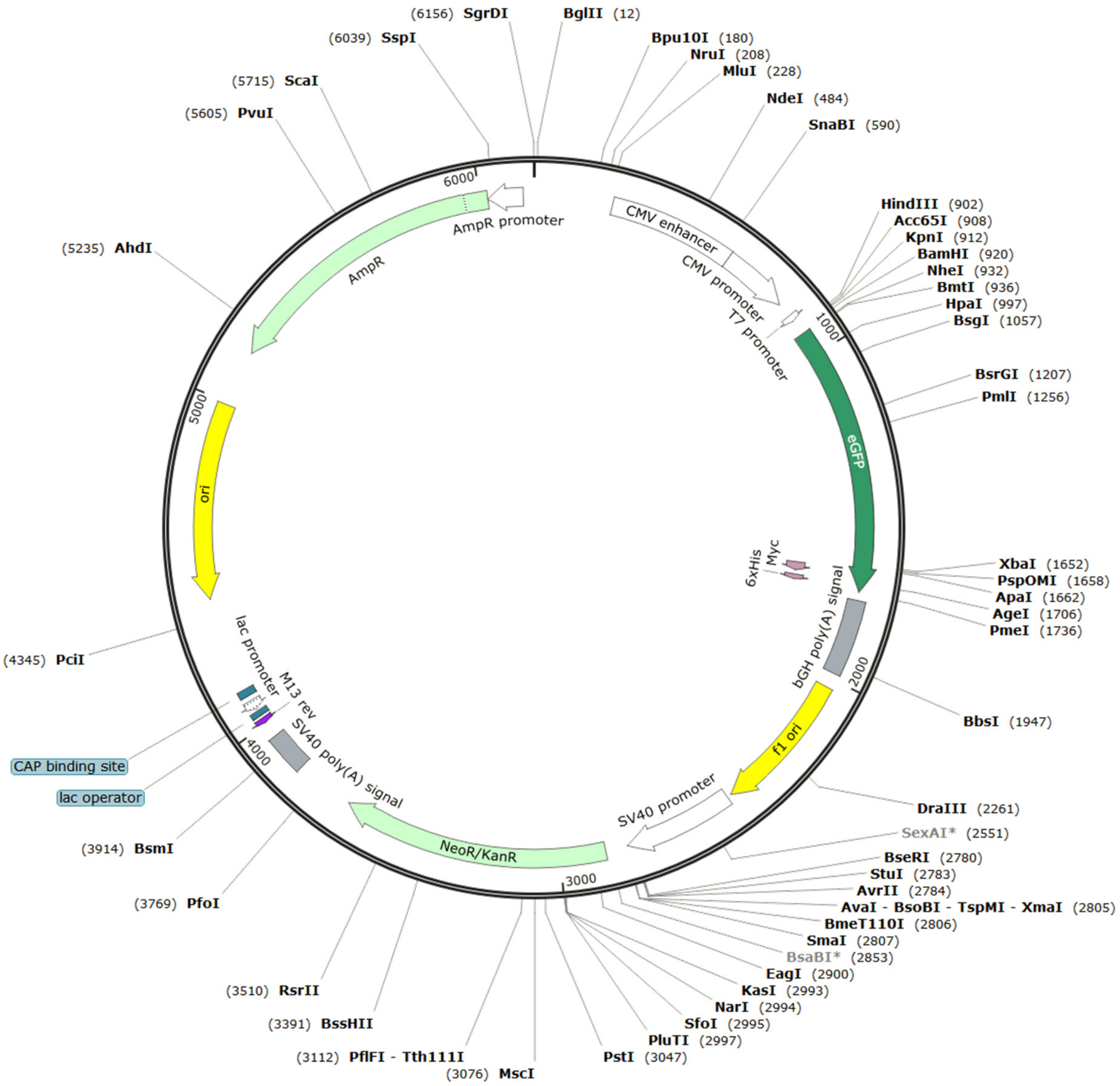

1 GACGGATCGG GAGATCTCCC GATCCCCTAT GGTGCACTCT CAGTACAATC TGCTCTGATG
61 CCGCATAGTT AAGCCAGTAT CTGCTCCCTG CTTGTGTGTT GGAGGTCGCT GAGTAGTGCG
121 CGAGCAAAAT TTAAGCTACA ACAAGGCAAG GCTTGACCGA CAATTGCATG AAGAATCTGC
181 TTAGGGTTAG GCGTTTTGCG CTGCTTCGCG ATGTACGGGC CAGATATACG CGTTGACATT
241 GATTATTGAC TAGTTATTAA TAGTAATCAA TTACGGGGTC ATTAGTTCAT AGCCCATATA
301 TGGAGTTCCG CGTTACATAA CTTACGGTAA ATGGCCCGCC TGGCTGACCG CCCAACGACC
361 CCCGCCCATT GACGTCAATA ATGACGTATG TTCCCATAGT AACGCCAATA GGGACTTTCC
421 ATTGACGTCA ATGGGTGGAG TATTTACGGT AAACTGCCCA CTTGGCAGTA CATCAAGTGT
481 ATCATATGCC AAGTACGCCC CCTATTGACG TCAATGACGG TAAATGGCCC GCCTGGCATT
541 ATGCCCAGTA CATGACCTTA TGGGACTTTC CTACTTGGCA GTACATCTAC GTATTAGTCA
601 TCGCTATTAC CATGGTGATG CGGTTTTGGC AGTACATCAA TGGGCGTGGA TAGCGGTTTG
661 ACTCACGGGG ATTTCCAAGT CTCCACCCCA TTGACGTCAA TGGGAGTTTG TTTTGGCACC
721 AAAATCAACG GGACTTTCCA AAATGTCGTA ACAACTCCGC CCCATTGACG CAAATGGGCG

781 GTAGGCGTGT ACGGTGGGAG GTCTATATAA GCAGAGCTCT CTGGCTAACT AGAGAACCCA
841 CTGCTTACTG GCTTATCGAA ATTAATACGA CTCACTATAG GGAGACCCAA GCTGGCTAGT
901 TAAGCTTGGT ACCGAGCTCG GATCCACCAT GGCTAGCAAA GGAGAAGAAC TCTTCACTGG
961 AGTTGTCCCA ATTCTTGTTG AATTAGATGG TGATGTTAAC GGCCACAAGT TCTCTGTCAG
1021 TGGAGAGGGT GAAGGTGATG CAACATACGG AAAACTTACC CTGAAGTTCA TCTGCACTAC
1081 TGGCAAACTG CCTGTTCCGT GGCCGACACT AGTGACGACG CTCTGCTATG GCGTCCAGTG
1141 CTTTTCAAGA TACCCGGATC ACATGAAACG GCATGACTTT TTCAAGAGTG CCATGCCCGA
1201 AGGTTATGTA CAGGAAAGGA CCATCTTCTT CAAAGATGAC GGCAACTACA AGACACGTGC
1261 TGAAGTCAAG TTTGAAGGTG ATACCCTTGT TAATAGAATC GAGTTAAAAG GTATTGACTT
1321 CAAGGAAGAT GGCAACATTC TGGGACACAA ATTGGAATAC AACTATAACT CACACAATGT
1381 ATACATCATG GCAGACAAAC AAAAGAATGG AATCAAAGTG AACTTCAAGA CCCGCCACAA
1441 CATTGAAGAT GGAAGCGTTC AACTAGCAGA CCATTATCAA CAAAATACTC CAATTGGCGA
1501 TGGCCCTGTC CTTTTACCAG ACAACCATTA CCTGTCCACA CAATCTGCCC TTTCGAAAGA
1561 TCCCAACGAA AAGAGAGACC ACATGGTCCT TCTTGAGTTT GTAACAGCTG CTGGGATTAC
1621 ACATGGCATG GATGAACTAT ACAAATCCGG CTCTAGAGGG CCCTTCGAAC AAAAACTCAT
1681 CTCAGAAGAG GATCTGAATA TGCATACCGG TCATCATCAC CATCACCATT GAGTTTAAAC
1741 CCGCTGATCA GCCTCGACTG TGCCTTCTAG TTGCCAGCCA TCTGTTGTTT GCCCCTCCCC
1801 CGTGCCTTCC TTGACCCTGG AAGGTGCCAC TCCCACTGTC CTTTCCTAAT AAAATGAGGA
1861 AATTGCATCG CATTGTCTGA GTAGGTGTCA TTCTATTCTG GGGGGTGGGG TGGGGCAGGA
1921 CAGCAAGGGG GAGGATTGGG AAGACAATAG CAGGCATGCT GGGGATGCGG TGGGCTCTAT
1981 GGCTTCTGAG GCGGAAAGAA CCAGCTGGGG CTCTAGGGGG TATCCCCACG CGCCCTGTAG
2041 CGGCGCATTA AGCGCGGCGG GTGTGGTGGT TACGCGCAGC GTGACCGCTA CACTTGCCAG
2101 CGCCCTAGCG CCCGCTCCTT TCGCTTTCTT CCCTTCCTTT CTCGCCACGT TCGCCGGCTT
2161 TCCCCGTCAA GCTCTAAATC GGGGGCTCCC TTTAGGGTTC CGATTTAGTG CTTTACGGCA
2221 CCTCGACCCC AAAAAACTTG ATTAGGGTGA TGGTTCACGT AGTGGGCCAT CGCCCTGATA
2281 GACGGTTTTT CGCCCTTTGA CGTTGGAGTC CACGTTCTTT AATAGTGGAC TCTTGTTCCA
2341 AACTGGAACA ACACTCAACC CTATCTCGGT CTATTCTTTT GATTTATAAG GGATTTTGCC
2401 GATTTCGGCC TATTGGTTAA AAAATGAGCT GATTTAACAA AAATTTAACG CGAATTAATT
2461 CTGTGGAATG TGTGTCAGTT AGGGTGTGGA AAGTCCCCAG GCTCCCCAGC AGGCAGAAGT
2521 ATGCAAAGCA TGCATCTCAA TTAGTCAGCA ACCAGGTGTG GAAAGTCCCC AGGCTCCCCA
2581 GCAGGCAGAA GTATGCAAAG CATGCATCTC AATTAGTCAG CAACCATAGT CCCGCCCCTA
2641 ACTCCGCCCA TCCCGCCCCT AACTCCGCCC AGTTCCGCCC ATTCTCCGCC CCATGGCTGA
2701 CTAATTTTTT TTATTTATGC AGAGGCCGAG GCCGCCTCTG CCTCTGAGCT ATTCCAGAAG
2761 TAGTGAGGAG GCTTTTTTGG AGGCCTAGGC TTTTGCAAAA AGCTCCCGGG AGCTTGTATA
2821 TCCATTTTCG GATCTGATCA AGAGACAGGA TGAGGATCGT TTCGCATGAT TGAACAAGAT
2881 GGATTGCACG CAGGTTCTCC GGCCGCTTGG GTGGAGAGGC TATTCGGCTA TGACTGGGCA
2941 CAACAGACAA TCGGCTGCTC TGATGCCGCC GTGTTCCGGC TGTCAGCGCA GGGGCGCCCG
3001 GTTCTTTTTG TCAAGACCGA CCTGTCCGGT GCCCTGAATG AACTGCAGGA CGAGGCAGCG
3061 CGGCTATCGT GGCTGGCCAC GACGGGCGTT CCTTGCGCAG CTGTGCTCGA CGTTGTCACT
3121 GAAGCGGGAA GGGACTGGCT GCTATTGGGC GAAGTGCCGG GGCAGGATCT CCTGTCATCT
3181 CACCTTGCTC CTGCCGAGAA AGTATCCATC ATGGCTGATG CAATGCGGCG GCTGCATACG
3241 CTTGATCCGG CTACCTGCCC ATTCGACCAC CAAGCGAAAC ATCGCATCGA GCGAGCACGT
3301 ACTCGGATGG AAGCCGGTCT TGTCGATCAG GATGATCTGG ACGAAGAGCA TCAGGGGCTC
3361 GCGCCAGCCG AACTGTTCGC CAGGCTCAAG GCGCGCATGC CCGACGGCGA GGATCTCGTC
3421 GTGACCCATG GCGATGCCTG CTTGCCGAAT ATCATGGTGG AAAATGGCCG CTTTTCTGGA
3481 TTCATCGACT GTGGCCGGCT GGGTGTGGCG GACCGCTATC AGGACATAGC GTTGGCTACC
3541 CGTGATATTG CTGAAGAGCT TGGCGGCGAA TGGGCTGACC GCTTCCTCGT GCTTTACGGT
3601 ATCGCCGCTC CCGATTCGCA GCGCATCGCC TTCTATCGCC TTCTTGACGA GTTCTTCTGA
3661 GCGGGACTCT GGGGTTCGAA ATGACCGACC AAGCGACGCC CAACCTGCCA TCACGAGATT
3721 TCGATTCCAC CGCCGCCTTC TATGAAAGGT TGGGCTTCGG AATCGTTTTC CGGGACGCCG
3781 GCTGGATGAT CCTCCAGCGC GGGGATCTCA TGCTGGAGTT CTTCGCCCAC CCCAACTTGT
3841 TTATTGCAGC TTATAATGGT TACAAATAAA GCAATAGCAT CACAAATTTC ACAAATAAAG
3901 CATTTTTTTC ACTGCATTCT AGTTGTGGTT TGTCCAAACT CATCAATGTA TCTTATCATG
3961 TCTGTATACC GTCGACCTCT AGCTAGAGCT TGGCGTAATC ATGGTCATAG CTGTTTCCTG
4021 TGTGAAATTG TTATCCGCTC ACAATTCCAC ACAACATACG AGCCGGAAGC ATAAAGTGTA
4081 AAGCCTGGGG TGCCTAATGA GTGAGCTAAC TCACATTAAT TGCGTTGCGC TCACTGCCCG
4141 CTTTCCAGTC GGGAAACCTG TCGTGCCAGC TGCATTAATG AATCGGCCAA CGCGCGGGGA

4201 GAGGCGGTTT GCGTATTGGG CGCTCTTCCG CTTCCTCGCT CACTGACTCG CTGCGCTCGG
4261 TCGTTCGGCT GCGGCGAGCG GTATCAGCTC ACTCAAAGGC GGTAATACGG TTATCCACAG
4321 AATCAGGGGA TAACGCAGGA AAGAACATGT GAGCAAAAGG CCAGCAAAAG GCCAGGAACC
4381 GTAAAAAGGC CGCGTTGCTG GCGTTTTTCC ATAGGCTCCG CCCCCCTGAC GAGCATCACA
4441 AAAATCGACG CTCAAGTCAG AGGTGGCGAA ACCCGACAGG ACTATAAAGA TACCAGGCGT
4501 TTCCCCCTGG AAGCTCCCTC GTGCGCTCTC CTGTTCCGAC CCTGCCGCTT ACCGGATACC
4561 TGTCCGCCTT TCTCCCTTCG GGAAGCGTGG CGCTTTCTCA TAGCTCACGC TGTAGGTATC
4621 TCAGTTCGGT GTAGGTCGTT CGCTCCAAGC TGGGCTGTGT GCACGAACCC CCCGTTCAGC
4681 CCGACCGCTG CGCCTTATCC GGTAACTATC GTCTTGAGTC CAACCCGGTA AGACACGACT
4741 TATCGCCACT GGCAGCAGCC ACTGGTAACA GGATTAGCAG AGCGAGGTAT GTAGGCGGTG
4801 CTACAGAGTT CTTGAAGTGG TGGCCTAACT ACGGCTACAC TAGAAGAACA GTATTTGGTA
4861 TCTGCGCTCT GCTGAAGCCA GTTACCTTCG GAAAAAGAGT TGGTAGCTCT TGATCCGGCA
4921 AACAAACCAC CGCTGGTAGC GGTTTTTTTG TTTGCAAGCA GCAGATTACG CGCAGAAAAA
4981 AAGGATCTCA AGAAGATCCT TTGATCTTTT CTACGGGGTC TGACGCTCAG TGGAACGAAA
5041 ACTCACGTTA AGGGATTTTG GTCATGAGAT TATCAAAAAG GATCTTCACC TAGATCCTTT
5101 TAAATTAAAA ATGAAGTTTT AAATCAATCT AAAGTATATA TGAGTAAACT TGGTCTGACA
5161 GTTACCAATG CTTAATCAGT GAGGCACCTA TCTCAGCGAT CTGTCTATTT CGTTCATCCA
5221 TAGTTGCCTG ACTCCCCGTC GTGTAGATAA CTACGATACG GGAGGGCTTA CCATCTGGCC
5281 CCAGTGCTGC AATGATACCG CGAGACCCAC GCTCACCGGC TCCAGATTTA TCAGCAATAA
5341 ACCAGCCAGC CGGAAGGGCC GAGCGCAGAA GTGGTCCTGC AACTTTATCC GCCTCCATCC
5401 AGTCTATTAA TTGTTGCCGG GAAGCTAGAG TAAGTAGTTC GCCAGTTAAT AGTTTGCGCA
5461 ACGTTGTTGC CATTGCTACA GGCATCGTGG TGTCACGCTC GTCGTTTGGT ATGGCTTCAT
5521 TCAGCTCCGG TTCCCAACGA TCAAGGCGAG TTACATGATC CCCCATGTTG TGCAAAAAAG
5581 CGGTTAGCTC CTTCGGTCCT CCGATCGTTG TCAGAAGTAA GTTGGCCGCA GTGTTATCAC
5641 TCATGGTTAT GGCAGCACTG CATAATTCTC TTACTGTCAT GCCATCCGTA AGATGCTTTT
5701 CTGTGACTGG TGAGTACTCA ACCAAGTCAT TCTGAGAATA GTGTATGCGG CGACCGAGTT
5761 GCTCTTGCCC GGCGTCAATA CGGGATAATA CCGCGCCACA TAGCAGAACT TTAAAAGTGC
5821 TCATCATTGG AAAACGTTCT TCGGGGCGAA AACTCTCAAG GATCTTACCG CTGTTGAGAT
5881 CCAGTTCGAT GTAACCCACT CGTGCACCCA ACTGATCTTC AGCATCTTTT ACTTTCACCA
5941 GCGTTTCTGG GTGAGCAAAA ACAGGAAGGC AAAATGCCGC AAAAAAGGGA ATAAGGGCGA
6001 CACGGAAATG TTGAATACTC ATACTCTTCC TTTTTCAATA TTATTGAAGC ATTTATCAGG
6061 GTTATTGTCT CATGAGCGGA TACATATTTG AATGTATTTA GAAAAATAAA CAAATAGGGG
6121 TTCCGCGCAC ATTTCCCCGA AAAGTGCCAC CTGACGTC

Plasmid-map and sequence of the **Tornado OHA vector EF1α (pTS1593):**

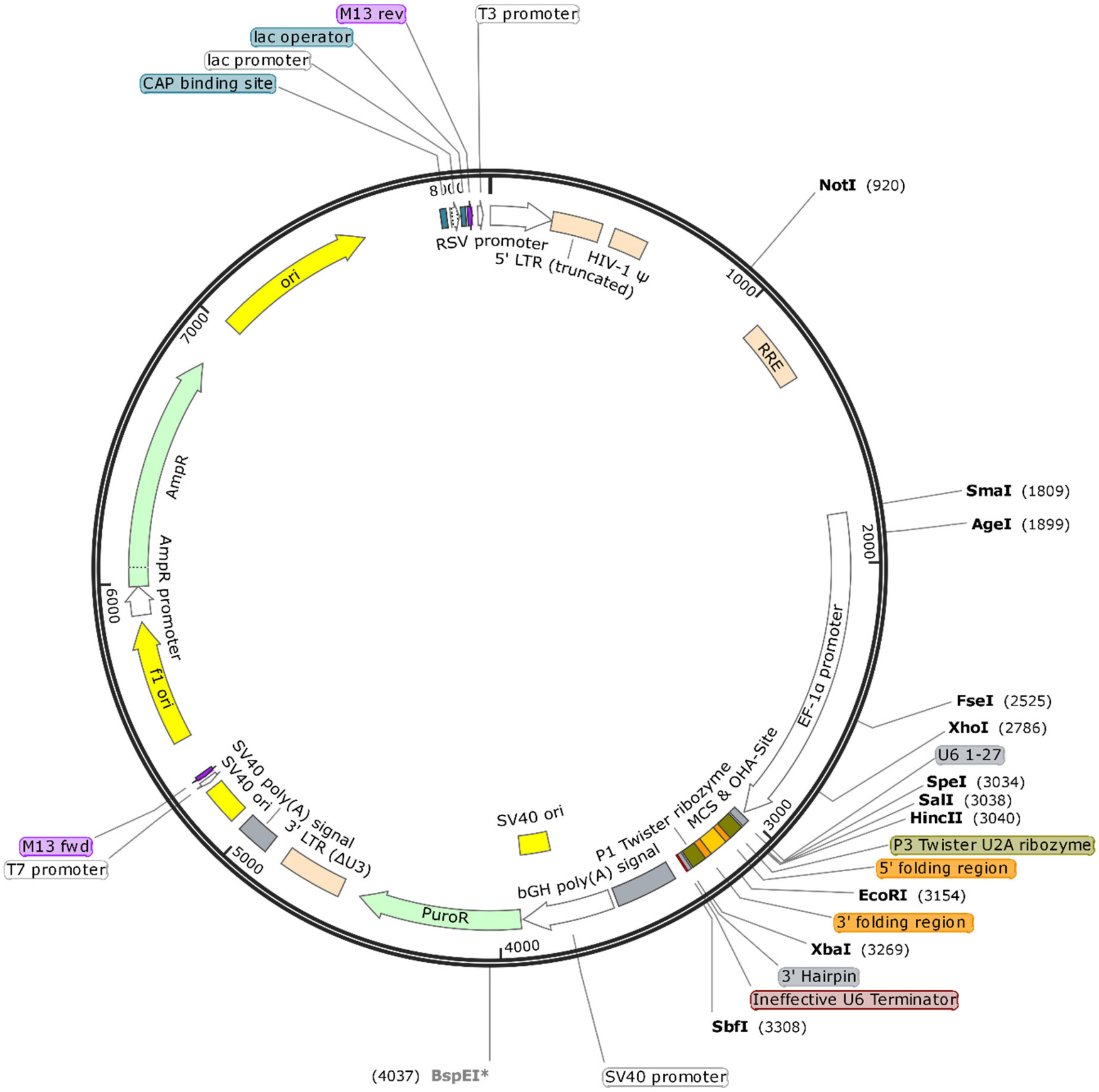


```
1          AATGTAGTCT TATGCAATAC TCTTGTAGTC TTGCAACATG GTAACGATGA GTTAGCAACA
61         TGCCTTACAA GGAGAGAAAA AGCACCGTGC ATGCCGATTG GTGGAAGTAA GGTGGTACGA
121        TCGTGCCTTA TTAGGAAGGC AACAGACGGG TCTGACATGG ATTGGACGAA CCACTGAATT
181        GCCGCATTGC AGAGATATTG TATTTAAGTG CCTAGCTCGA TACATAAACG GGTCTCTCTG
241        GTTAGACCAG ATCTGAGCCT GGGAGCTCTC TGGCTAACTA GGGAACCCAC TGCTTAAGCC
301        TCAATAAAGC TTGCCTTGAG TGCTTCAAGT AGTGTGTGCC CGTCTGTTGT GTGACTCTGG
361        TAACTAGAGA TCCCTCAGAC CCTTTTAGTC AGTGTGGAAA ATCTCTAGCA GTGGCGCCCG
421        AACAGGGACT TGAAAGCGAA AGGGAAACCA GAGGAGCTCT CTCGACGCAG GACTCGGCTT
481        GCTGAAGCGC GCACGGCAAG AGGCGAGGGG CGGCGACTGG TGAGTACGCC AAAAATTTTG
541        ACTAGCGGAG GCTAGAAGGA GAGAGATGGG TGCGAGAGCG TCAGTATTAA GCGGGGGAGA
601        ATTAGATCGC GATGGGAAAA AATTCGGTTA AGGCCAGGGG GAAAGAAAAA ATATAAATTA
661        AAACATATAG TATGGGCAAG CAGGGAGCTA GAACGATTCG CAGTTAATCC TGGCCTGTTA
```

721 GAAACATCAG AAGGCTGTAG ACAAATACTG GGACAGCTAC AACCATCCCT TCAGACAGGA
781 TCAGAAGAAC TTAGATCATT ATATAATACA GTAGCAACCC TCTATTGTGT GCATCAAAGG
841 ATAGAGATAA AAGACACCAA GGAAGCTTTA GACAAGATAG AGGAAGAGCA AAACAAAAGT
901 AAGACCACCG CACAGCAAGC GGCCGCTGAT CTTCAGACCT GGAGGAGGAG ATATGAGGGA
961 CAATTGGAGA AGTGAATTAT ATAAATATAA AGTAGTAAAA ATTGAACCAT TAGGAGTAGC
1021 ACCCACCAAG GCAAAGAGAA GAGTGGTGCA GAGAGAAAAA AGAGCAGTGG GAATAGGAGC
1081 TTTGTTCCTT GGGTTCTTGG GAGCAGCAGG AAGCACTATG GGCGCAGCGT CAATGACGCT
1141 GACGGTACAG GCCAGACAAT TATTGTCTGG TATAGTGCAG CAGCAGAACA ATTTGCTGAG
1201 GGCTATTGAG GCGCAACAGC ATCTGTTGCA ACTCACAGTC TGGGGCATCA AGCAGCTCCA
1261 GGCAAGAATC CTGGCTGTGG AAAGATACCT AAAGGATCAA CAGCTCCTGG GGATTTGGGG
1321 TTGCTCTGGA AAACTCATTT GCACCACTGC TGTGCCTTGG AATGCTAGTT GGAGTAATAA
1381 ATCTCTGGAA CAGATTTGGA ATCACACGAC CTGGATGGAG TGGGACAGAG AAATTAACAA
1441 TTACACAAGC TTAATACACT CCTTAATTGA AGAATCGCAA AACCAGCAAG AAAAGAATGA
1501 ACAAGAATTA TTGGAATTAG ATAAATGGGC AAGTTTGTGG AATTGGTTTA ACATAACAAA
1561 TTGGCTGTGG TATATAAAAT TATTCATAAT GATAGTAGGA GGCTTGGTAG GTTTAAGAAT
1621 AGTTTTTGCT GTACTTTCTA TAGTGAATAG AGTTAGGCAG GGATATTCAC CATTATCGTT
1681 TCAGACCCAC CTCCCAACCC CGAGGGGACC CGACAGGCCC GAAGGAATAG AAGAAGAAGG
1741 TGGAGAGAGA GACAGAGACA GATCCATTCG ATTAGTGAAC GGATCTCGAC GGTATCGCTA
1801 GTGGATCCCG GGATCCGTGA GGCTCCGGTG CCCGTCAGTG GGCAGAGCGC ACATCGCCCA
1861 CAGTCCCCGA GAAGTTGGGG GGAGGGGTCG GCAATTGAAC CGGTGCCTAG AGAAGGTGGC
1921 GCGGGGTAAA CTGGGAAAGT GATGTCGTGT ACTGGCTCCG CCTTTTTCCC GAGGGTGGGG
1981 GAGAACCGTA TATAAGTGCA GTAGTCGCCG TGAACGTTCT TTTTCGCAAC GGGTTTGCCG
2041 CCAGAACACA GGTAAGTGCC GTGTGTGGTT CCCGCGGGCC TGGCCTCTTT ACGGGTTATG
2101 GCCCTTGCGT GCCTTGAATT ACTTCCACCT GGCTGCAGTA CGTGATTCTT GATCCCGAGC
2161 TTCGGGTTGG AAGTGGGTGG GAGAGTTCGA GGCCTTGCGC TTAAGGAGCC CCTTCGCCTC
2221 GTGCTTGAGT TGAGGCCTGG CCTGGGCGCT GGGGCCGCCG CGTGCGAATC TGGTGGCACC
2281 TTCGCGCCTG TCTCGCTGCT TTCGATAAGT CTCTAGCCAT TTAAAATTTT TGATGACCTG
2341 CTGCGACGCT TTTTTTCTGG CAAGATAGTC TTGTAAATGC GGGCCAAGAT CTGCACACTG
2401 GTATTTCGGT TTTTGGGGCC GCGGGCGGCG ACGGGGCCCG TGCGTCCCAG CGCACATGTT
2461 CGGCGAGGCG GGGCCTGCGA GCGCGGCCAC CGAGAATCGG ACGGGGGTAG TCTCAAGCTG
2521 GCCGGCCTGC TCTGGTGCCT GGCCTCGCGC CGCCGTGTAT CGCCCCGCCC TGGGCGGCAA
2581 GGCTGGCCCG GTCGGCACCA GTTGCGTGAG CGGAAAGATG GCCGCTTCCC GGCCCTGCTG
2641 CAGGGAGCTC AAAATGGAGG ACGCGGCGCT CGGGAGAGCG GGCGGGTGAG TCACCCACAC
2701 AAAGGAAAAG GGCCTTTCCG TCCTCAGCCG TCGCTTCATG TGACTCCACG GAGTACCGGG
2761 CGCCGTCCAG GCACCTCGAT TAGTTCTCGA GCTTTTGGAG TACGTCGTCT TTAGGTTGGG
2821 GGGAGGGGTT TTATGCGATG GAGTTTCCCC ACACTGAGTG GGTGGAGACT GAAGTTAGGC
2881 CAGCTTGGCA CTTGATGTAA TTCTCCTTGG AATTTGCCCT TTTTGAGTTT GGATCTTGGT
2941 TCATTCTCAA GCCTCAGACA GTGGTTCAAA GTTTTTTTCT TCCATTTCAG GTGTCGTGAG
3001 GTCTAGCACC GTGCTCGCTT CGGCAGCACA TATACTAGTC GACGCCATCA GTCGCCGGTC
3061 CCAAGCCCGG ATAAAATGGG AGGGGGCGGG AAACCGCCTA ACCATGCCGA CTGATGGCAG
3121 TAGTCTTCAC TGGTACCGAG CTCGGATCCA CTGGAATTCG CCATGCATCT AGGGCCCTAG
3181 AAGACTACTG CCATCAGTCG GCGTGGACTG TAGAACACTG CCAATGCCGG TCCCAAGCCC
3241 GGATAAAAGT GGAGGGTACA GTCCACGCTC TAGAGCGGAC TTCGGTCCGC TTTTTACTAG
3301 GACCTGCAGG CATGCTCGAC AGAGCTCGCT GATCAGCCTC GACTGTGCCT TCTAGTTGCC
3361 AGCCATCTGT TGTTTGCCCC TCCCCCGTGC CTTCCTTGAC CCTGGAAGGT GCCACTCCCA
3421 CTGTCCTTTC CTAATAAAAT GAGGAAATTG CATCGCATTG TCTGAGTAGG TGTCATTCTA
3481 TTCTGGGGGG TGGGGTGGGG CAGGACAGCA AGGGGGAGGA TTGGGAAGAT AATAGCAGGC
3541 ATGCTGGGGA TGCGGTGGGC TCTATGGTTT CGACCCTGTG GAATGTGTGT CAGTTAGGGT
3601 GTGGAAAGTC CCCAGGCTCC CCAGCAGGCA GAAGTATGCA AAGCATGCAT CTCAATTAGT
3661 CAGCAACCAG GTGTGGAAAG TCCCCAGGCT CCCCAGCAGG CAGAAGTATG CAAAGCATGC
3721 ATCTCAATTA GTCAGCAACC ATAGTCCCGC CCCTAACTCC GCCCATCCCG CCCCTAACTC
3781 CGCCCAGTTC CGCCCATTCT CCGCCCCATG GCTGACTAAT TTTTTTTATT TATGCAGAGG
3841 CCGAGGCCGC CTCGGCCTCT GAGCTATTCC AGAAGTAGTG AGGAGGCTTT TTTGGAGGCC
3901 TAGGCTTTTG CAAAAAGCTT ACCATGACCG AGTACAAGCC CACGGTGCGC CTCGCCACCC
3961 GCGACGACGT CCCCAGGGCC GTACGCACCC TCGCCGCCGC GTTCGCCGAC TACCCCGCCA
4021 CGCGCCACAC CGTCGATCCG GACCGCCACA TCGAGCGGGT CACCGAGCTG CAAGAACTCT
4081 TCCTCACGCG CGTCGGGCTC GACATCGGCA AGGTGTGGGT CGCGGACGAC GGCGCCGCGG

4141 TGGCGGTCTG GACCACGCCG GAGAGCGTCG AAGCGGGGGC GGTGTTCGCC GAGATCGGCC
4201 CGCGCATGGC CGAGTTGAGC GGTTCCCGGC TGGCCGCGCA GCAACAGATG GAAGGCCTCC
4261 TGGCGCCGCA CCGGCCCAAG GAGCCCGCGT GGTTCCTGGC CACCGTCGGC GTCTCGCCCG
4321 ACCACCAGGG CAAGGGTCTG GGCAGCGCCG TCGTGCTCCC CGGAGTGGAG GCGGCCGAGC
4381 GCGCCGGGGT GCCCGCCTTC CTGGAGACCT CCGCGCCCCG CAACCTCCCC TTCTACGAGC
4441 GGCTCGGCTT CACCGTCACC GCCGACGTCG AGGTGCCCGA AGGACCGCGC ACCTGGTGCA
4501 TGACCCGCAA GCCCGGTGCC TGAGGTACCT TTAAGACCAA TGACTTACAA GGCAGCTGTA
4561 GATCTTAGCC ACTTTTTAAA AGAAAAGGGG GGACTGGAAG GGCTAATTCA CTCCCAACGA
4621 AAACAAGATC TGCTTTTTGC TTGTACTGGG TCTCTCTGGT TAGACCAGAT CTGAGCCTGG
4681 GAGCTCTCTG GCTAACTAGG GAACCCACTG CTTAAGCCTC AATAAAGCTT GCCTTGAGTG
4741 CTTCAAGTAG TGTGTGCCCG TCTGTTGTGT GACTCTGGTA ACTAGAGATC CCTCAGACCC
4801 TTTTAGTCAG TGTGGAAAAT CTCTAGCAGT AGTAGTTCAT GTCATCTTAT TATTCAGTAT
4861 TTATAACTTG CAAAGAAATG AATATCAGAG AGTGAGAGGA ACTTGTTTAT TGCAGCTTAT
4921 AATGGTTACA AATAAAGCAA TAGCATCACA AATTTCACAA ATAAAGCATT TTTTTCACTG
4981 CATTCTAGTT GTGGTTTGTC CAAACTCATC AATGTATCTT ATCATGTCTG GCTCTAGCTA
5041 TCCCGCCCCT AACTCCGCCC ATCCCGCCCC TAACTCCGCC CAGTTCCGCC CATTCTCCGC
5101 CCCATGGCTG ACTAATTTTT TTTATTTATG CAGAGGCCGA GGCCGCCTCG GCCTCTGAGC
5161 TATTCCAGAA GTAGTGAGGA GGCTTTTTTG GAGGCCTAGG GACGTACCCA ATTCGCCCTA
5221 TAGTGAGTCG TATTACGCGC GCTCACTGGC CGTCGTTTTA CAACGTCGTG ACTGGGAAAA
5281 CCCTGGCGTT ACCCAACTTA ATCGCCTTGC AGCACATCCC CCTTTCGCCA GCTGGCGTAA
5341 TAGCGAAGAG GCCCGCACCG ATCGCCCTTC CCAACAGTTG CGCAGCCTGA ATGGCGAATG
5401 GGACGCGCCC TGTAGCGGCG CATTAAGCGC GGCGGGTGTG GTGGTTACGC GCAGCGTGAC
5461 CGCTACACTT GCCAGCGCCC TAGCGCCCGC TCCTTTCGCT TTCTTCCCTT CCTTTCTCGC
5521 CACGTTCGCC GGCTTTCCCC GTCAAGCTCT AAATCGGGGG CTCCCTTTAG GGTTCCGATT
5581 TAGTGCTTTA CGGCACCTCG ACCCCAAAAA ACTTGATTAG GGTGATGGTT CACGTAGTGG
5641 GCCATCGCCC TGATAGACGG TTTTTCGCCC TTTGACGTTG GAGTCCACGT TCTTTAATAG
5701 TGGACTCTTG TTCCAAACTG GAACAACACT CAACCCTATC TCGGTCTATT CTTTTGATTT
5761 ATAAGGGATT TTGCCGATTT CGGCCTATTG GTTAAAAAAT GAGCTGATTT AACAAAAATT
5821 TAACGCGAAT TTTAACAAAA TATTAACGCT TACAATTTAG GTGGCACTTT TCGGGGAAAT
5881 GTGCGCGGAA CCCCTATTTG TTTATTTTTC TAAATACATT CAAATATGTA TCCGCTCATG
5941 AGACAATAAC CCTGATAAAT GCTTCAATAA TATTGAAAAA GGAAGAGTAT GAGTATTCAA
6001 CATTTCCGTG TCGCCCTTAT TCCCTTTTTT GCGGCATTTT GCCTTCCTGT TTTTGCTCAC
6061 CCAGAAACGC TGGTGAAAGT AAAAGATGCT GAAGATCAGT TGGGTGCACG AGTGGGTTAC
6121 ATCGAACTGG ATCTCAACAG CGGTAAGATC CTTGAGAGTT TTCGCCCCGA AGAACGTTTT
6181 CCAATGATGA GCACTTTTAA AGTTCTGCTA TGTGGCGCGG TATTATCCCG TATTGACGCC
6241 GGGCAAGAGC AACTCGGTCG CCGCATACAC TATTCTCAGA ATGACTTGGT TGAGTACTCA
6301 CCAGTCACAG AAAAGCATCT TACGGATGGC ATGACAGTAA GAGAATTATG CAGTGCTGCC
6361 ATAACCATGA GTGATAACAC TGCGGCCAAC TTACTTCTGA CAACGATCGG AGGACCGAAG
6421 GAGCTAACCG CTTTTTTGCA CAACATGGGG GATCATGTAA CTCGCCTTGA TCGTTGGGAA
6481 CCGGAGCTGA ATGAAGCCAT ACCAAACGAC GAGCGTGACA CCACGATGCC TGTAGCAATG
6541 GCAACAACGT TGCGCAAACT ATTAACTGGC GAACTACTTA CTCTAGCTTC CCGGCAACAA
6601 TTAATAGACT GGATGGAGGC GGATAAAGTT GCAGGACCAC TTCTGCGCTC GGCCCTTCCG
6661 GCTGGCTGGT TTATTGCTGA TAAATCTGGA GCCGGTGAGC GTGGGTCTCG CGGTATCATT
6721 GCAGCACTGG GGCCAGATGG TAAGCCCTCC CGTATCGTAG TTATCTACAC GACGGGGAGT
6781 CAGGCAACTA TGGATGAACG AAATAGACAG ATCGCTGAGA TAGGTGCCTC ACTGATTAAG
6841 CATTGGTAAC TGTCAGACCA AGTTTACTCA TATATACTTT AGATTGATTT AAAACTTCAT
6901 TTTTAATTTA AAAGGATCTA GGTGAAGATC CTTTTTGATA ATCTCATGAC CAAAATCCCT
6961 TAACGTGAGT TTTCGTTCCA CTGAGCGTCA GACCCCGTAG AAAAGATCAA AGGATCTTCT
7021 TGAGATCCTT TTTTTCTGCG CGTAATCTGC TGCTTGCAAA CAAAAAAACC ACCGCTACCA
7081 GCGGTGGTTT GTTTGCCGGA TCAAGAGCTA CCAACTCTTT TTCCGAAGGT AACTGGCTTC
7141 AGCAGAGCGC AGATACCAAA TACTGTTCTT CTAGTGTAGC CGTAGTTAGG CCACCACTTC
7201 AAGAACTCTG TAGCACCGCC TACATACCTC GCTCTGCTAA TCCTGTTACC AGTGGCTGCT
7261 GCCAGTGGCG ATAAGTCGTG TCTTACCGGG TTGGACTCAA GACGATAGTT ACCGGATAAG
7321 GCGCAGCGGT CGGGCTGAAC GGGGGGTTCG TGCACACAGC CCAGCTTGGA GCGAACGACC
7381 TACACCGAAC TGAGATACCT ACAGCGTGAG CTATGAGAAA GCGCCACGCT TCCCGAAGGG
7441 AGAAAGGCGG ACAGGTATCC GGTAAGCGGC AGGGTCGGAA CAGGAGAGCG CACGAGGGAG
7501 CTTCCAGGGG GAAACGCCTG GTATCTTTAT AGTCCTGTCG GGTTTCGCCA CCTCTGACTT

7561 GAGCGTCGAT TTTTGTGATG CTCGTCAGGG GGGCGGAGCC TATGGAAAAA CGCCAGCAAC
7621 GCGGCCTTTT TACGGTTCCT GGCCTTTTGC TGGCCTTTTG CTCACATGTT CTTTCCTGCG
7681 TTATCCCCTG ATTCTGTGGA TAACCGTATT ACCGCCTTTG AGTGAGCTGA TACCGCTCGC
7741 CGCAGCCGAA CGACCGAGCG CAGCGAGTCA GTGAGCGAGG AAGCGGAAGA GCGCCCAATA
7801 CGCAAACCGC CTCTCCCCGC GCGTTGGCCG ATTCATTAAT GCAGCTGGCA CGACAGGTTT
7861 CCCGACTGGA AAGCGGGCAG TGAGCGCAAC GCAATTAATG TGAGTTAGCT CACTCATTAG
7921 GCACCCCAGG CTTTACACTT TATGCTTCCG GCTCGTATGT TGTGTGGAAT TGTGAGCGGA
7981 TAACAATTTC ACACAGGAAA CAGCTATGAC CATGATTACG CCAAGCGCGC AATTAACCCT
8041 CACTAAAGGG AACAAAAGCT GGACTGCAAG CTT

Plasmid-map and sequence of the **Tornado OHA vector U6 (pTS1790):**

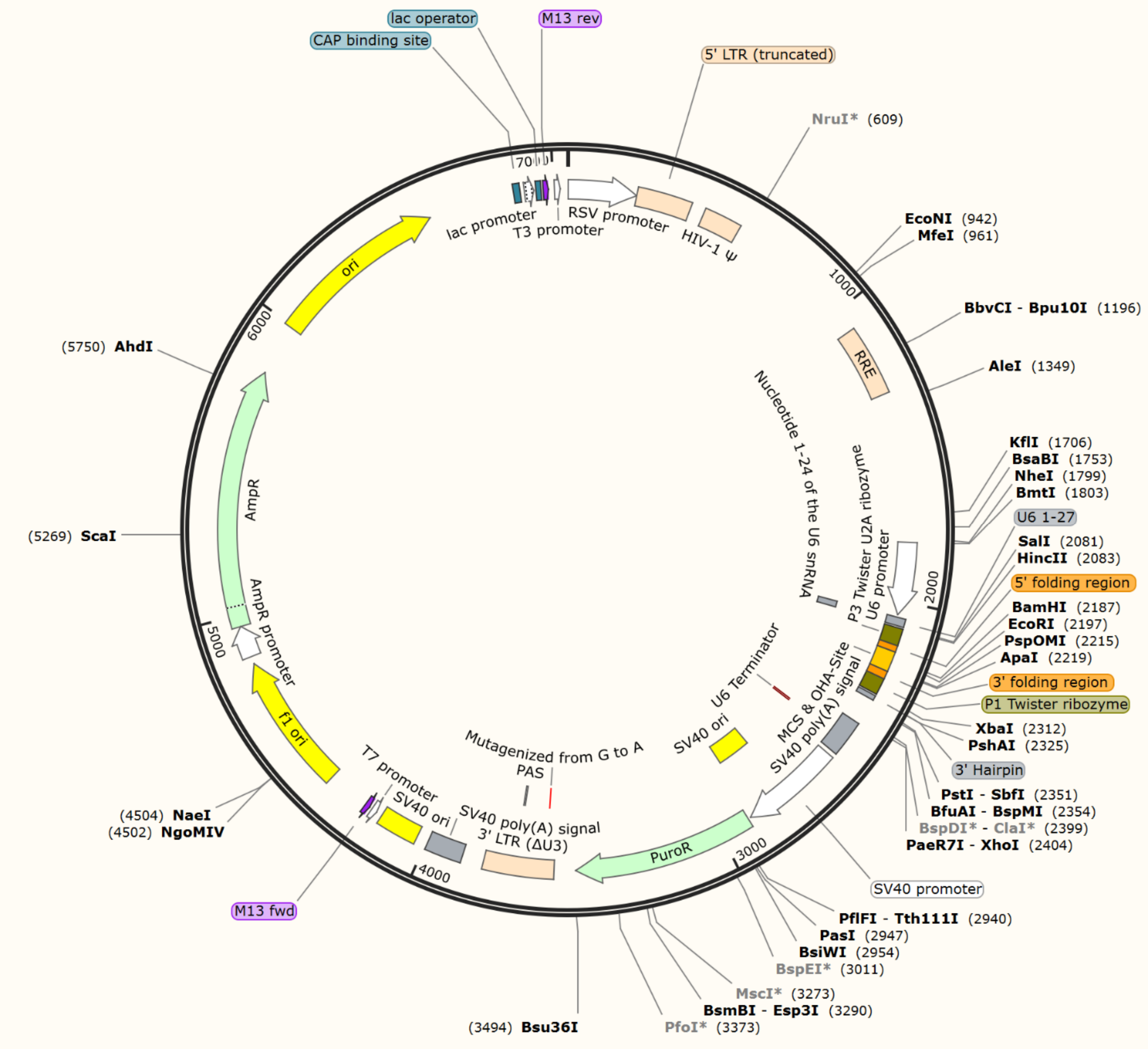

1 AATGTAGTCT TATGCAATAC TCTTGTAGTC TTGCAACATG GTAACGATGA GTTAGCAACA
61 TGCCTTACAA GGAGAGAAAA AGCACCGTGC ATGCCGATTG GTGGAAGTAA GGTGGTACGA
121 TCGTGCCTTA TTAGGAAGGC AACAGACGGG TCTGACATGG ATTGGACGAA CCACTGAATT
181 GCCGCATTGC AGAGATATTG TATTTAAGTG CCTAGCTCGA TACATAAACG GGTCTCTCTG
241 GTTAGACCAG ATCTGAGCCT GGGAGCTCTC TGGCTAACTA GGGAACCCAC TGCTTAAGCC
301 TCAATAAAGC TTGCCTTGAG TGCTTCAAGT AGTGTGTGCC CGTCTGTTGT GTGACTCTGG
361 TAACTAGAGA TCCCTCAGAC CCTTTTAGTC AGTGTGGAAA ATCTCTAGCA GTGGCGCCCG
421 AACAGGGACT TGAAAGCGAA AGGGAAACCA GAGGAGCTCT CTCGACGCAG GACTCGGCTT
481 GCTGAAGCGC GCACGGCAAG AGGCGAGGGG CGGCGACTGG TGAGTACGCC AAAAATTTTG
541 ACTAGCGGAG GCTAGAAGGA GAGAGATGGG TGCGAGAGCG TCAGTATTAA GCGGGGGAGA
601 ATTAGATCGC GATGGGAAAA AATTCGGTTA AGGCCAGGGG GAAAGAAAAA ATATAAATTA
661 AAACATATAG TATGGGCAAG CAGGGAGCTA GAACGATTCG CAGTTAATCC TGGCCTGTTA
721 GAAACATCAG AAGGCTGTAG ACAAATACTG GGACAGCTAC AACCATCCCT TCAGACAGGA
781 TCAGAAGAAC TTAGATCATT ATATAATACA GTAGCAACCC TCTATTGTGT GCATCAAAGG
841 ATAGAGATAA AAGACACCAA GGAAGCTTTA GACAAGATAG AGGAAGAGCA AAACAAAAGT

901 AAGACCACCG CACAGCAAGC GGCCGCTGAT CTTCAGACCT GGAGGAGGAG ATATGAGGGA
961 CAATTGGAGA AGTGAATTAT ATAAATATAA AGTAGTAAAA ATTGAACCAT TAGGAGTAGC
1021 ACCCACCAAG GCAAAGAGAA GAGTGGTGCA GAGAGAAAAA AGAGCAGTGG GAATAGGAGC
1081 TTTGTTCCTT GGGTTCTTGG GAGCAGCAGG AAGCACTATG GGCGCAGCGT CAATGACGCT
1141 GACGGTACAG GCCAGACAAT TATTGTCTGG TATAGTGCAG CAGCAGAACA ATTTGCTGAG
1201 GGCTATTGAG GCGCAACAGC ATCTGTTGCA ACTCACAGTC TGGGGCATCA AGCAGCTCCA
1261 GGCAAGAATC CTGGCTGTGG AAAGATACCT AAAGGATCAA CAGCTCCTGG GGATTTGGGG
1321 TTGCTCTGGA AAACTCATTT GCACCACTGC TGTGCCTTGG AATGCTAGTT GGAGTAATAA
1381 ATCTCTGGAA CAGATTTGGA ATCACACGAC CTGGATGGAG TGGGACAGAG AAATTAACAA
1441 TTACACAAGC TTAATACACT CCTTAATTGA AGAATCGCAA AACCAGCAAG AAAAGAATGA
1501 ACAAGAATTA TTGGAATTAG ATAAATGGGC AAGTTTGTGG AATTGGTTTA ACATAACAAA
1561 TTGGCTGTGG TATATAAAAT TATTCATAAT GATAGTAGGA GGCTTGGTAG GTTTAAGAAT
1621 AGTTTTTGCT GTACTTTCTA TAGTGAATAG AGTTAGGCAG GGATATTCAC CATTATCGTT
1681 TCAGACCCAC CTCCCAACCC CGAGGGGACC CGGATTTCTT GTCACGCTTT GATGCATCGT
1741 TGGTGGTTGA TGGATATCTG ACGAACATGT GCACAGTGGT ACAAGGTATT CCTGGTTGGC
1801 TAGCGAGGGC CTATTTCCCA TGATTCCTTC ATATTTGCAT ATACGATACA AGGCTGTTAG
1861 AGAGATAATT AGAATTAATT TGACTGTAAA CACAAAGATA TTAGTACAAA ATACGTGACG
1921 TAGAAAGTAA TAATTTCTTG GGTAGTTTGC AGTTTTAAAA TTATGTTTTA AAATGGACTA
1981 TCATATGCTT ACCGTAACTT GAAAGTATTT CGATTTCTTG GCTTTATATA TCTTGTGGAA
2041 AGGACGAAAC ACCGTGCTCG CTTCGGCAGC ACATATACTA GTCGACGCCA TCAGTCGCCG
2101 GTCCCAAGCC CGGATAAAAT GGGAGGGGGC GGGAAACCGC CTAACCATGC CGACTGATGG
2161 CAGTAGTCTT CACTGGTACC GAGCTCGGAT CCACTGGAAT TCGCCATGCA TCTAGGGCCC
2221 TAGAAGACTA CTGCCATCAG TCGGCGTGGA CTGTAGAACA CTGCCAATGC CGGTCCCAAG
2281 CCCGGATAAA AGTGGAGGGT ACAGTCCACG CTCTAGAGCG GACTTCGGTC CGCTTTTTAC
2341 TAGGACCTGC AGGCATGCAA GCTTGACGTC GGTTACCGAT ATCCATATGG CGGCCGCATC
2401 GATCTCGAGC CGCGGACTAG TAACTTGTTT ATTGCAGCTT ATAATGGTTA CAAATAAAGC
2461 AATAGCATCA CAAATTTCAC AAATAAAGCA TTTTTTTCAC TGCATTCTAG TTGTGGTTTG
2521 TCCAAACTCA TCAATGTATC TTATCGACCC TGTGGAATGT GTGTCAGTTA GGGTGTGGAA
2581 AGTCCCCAGG CTCCCCAGCA GGCAGAAGTA TGCAAAGCAT GCATCTCAAT TAGTCAGCAA
2641 CCAGGTGTGG AAAGTCCCCA GGCTCCCCAG CAGGCAGAAG TATGCAAAGC ATGCATCTCA
2701 ATTAGTCAGC AACCATAGTC CCGCCCCTAA CTCCGCCCAT CCCGCCCCTA ACTCCGCCCA
2761 GTTCCGCCCA TTCTCCGCCC CATGGCTGAC TAATTTTTTT TATTTATGCA GAGGCCGAGG
2821 CCGCCTCGGC CTCTGAGCTA TTCCAGAAGT AGTGAGGAGG CTTTTTTGGA GGCCTAGGCT
2881 TTTGCAAAAA GCTTACCATG ACCGAGTACA AGCCCACGGT GCGCCTCGCC ACCCGCGACG
2941 ACGTCCCCAG GGCCGTACGC ACCCTCGCCG CCGCGTTCGC CGACTACCCC GCCACGCGCC
3001 ACACCGTCGA TCCGGACCGC CACATCGAGC GGGTCACCGA GCTGCAAGAA CTCTTCCTCA
3061 CGCGCGTCGG GCTCGACATC GGCAAGGTGT GGGTCGCGGA CGACGGCGCC GCGGTGGCGG
3121 TCTGGACCAC GCCGGAGAGC GTCGAAGCGG GGGCGGTGTT CGCCGAGATC GGCCCGCGCA
3181 TGGCCGAGTT GAGCGGTTCC CGGCTGGCCG CGCAGCAACA GATGGAAGGC CTCCTGGCGC
3241 CGCACCGGCC CAAGGAGCCC GCGTGGTTCC TGGCCACCGT CGGCGTCTCG CCCGACCACC
3301 AGGGCAAGGG TCTGGGCAGC GCCGTCGTGC TCCCCGGAGT GGAGGCGGCC GAGCGCGCCG
3361 GGGTGCCCGC CTTCCTGGAG ACCTCCGCGC CCCGCAACCT CCCCTTCTAC GAGCGGCTCG
3421 GCTTCACCGT CACCGCCGAC GTCGAGGTGC CCGAAGGACC GCGCACCTGG TGCATGACCC
3481 GCAAGCCCGG TGCCTGAGGT ACCTTTAAGA CCAATGACTT ACAAGGCAGC TGTAGATCTT
3541 AGCCACTTTT TAAAAGAAAA GGGGGGACTG GAAGGGCTAA TTCACTCCCA ACGAAAACAA
3601 GATCTGCTTT TTGCTTGTAC TGGGTCTCTC TGGTTAGACC AGATCTGAGC CTGGGAGCTC
3661 TCTGGCTAAC TAGGGAACCC ACTGCTTAAG CCTCAATAAA GCTTGCCTTG AGTGCTTCAA
3721 GTAGTGTGTG CCCGTCTGTT GTGTGACTCT GGTAACTAGA GATCCCTCAG ACCCTTTTAG
3781 TCAGTGTGGA AAATCTCTAG CAGTAGTAGT TCATGTCATC TTATTATTCA GTATTTATAA
3841 CTTGCAAAGA AATGAATATC AGAGAGTGAG AGGAACTTGT TTATTGCAGC TTATAATGGT
3901 TACAAATAAA GCAATAGCAT CACAAATTTC ACAAATAAAG CATTTTTTTC ACTGCATTCT
3961 AGTTGTGGTT TGTCCAAACT CATCAATGTA TCTTATCATG TCTGGCTCTA GCTATCCCGC
4021 CCCTAACTCC GCCCATCCCG CCCCTAACTC CGCCCAGTTC CGCCCATTCT CCGCCCCATG
4081 GCTGACTAAT TTTTTTTATT TATGCAGAGG CCGAGGCCGC CTCGGCCTCT GAGCTATTCC
4141 AGAAGTAGTG AGGAGGCTTT TTTGGAGGCC TAGGGACGTA CCCAATTCGC CCTATAGTGA
4201 GTCGTATTAC GCGCGCTCAC TGGCCGTCGT TTTACAACGT CGTGACTGGG AAAACCCTGG
4261 CGTTACCCAA CTTAATCGCC TTGCAGCACA TCCCCCTTTC GCCAGCTGGC GTAATAGCGA

4321 AGAGGCCCGC ACCGATCGCC CTTCCCAACA GTTGCGCAGC CTGAATGGCG AATGGGACGC
4381 GCCCTGTAGC GGCGCATTAA GCGCGGCGGG TGTGGTGGTT ACGCGCAGCG TGACCGCTAC
4441 ACTTGCCAGC GCCCTAGCGC CCGCTCCTTT CGCTTTCTTC CCTTCCTTTC TCGCCACGTT
4501 CGCCGGCTTT CCCCGTCAAG CTCTAAATCG GGGGCTCCCT TTAGGGTTCC GATTTAGTGC
4561 TTTACGGCAC CTCGACCCCA AAAAACTTGA TTAGGGTGAT GGTTCACGTA GTGGGCCATC
4621 GCCCTGATAG ACGGTTTTTC GCCCTTTGAC GTTGGAGTCC ACGTTCTTTA ATAGTGGACT
4681 CTTGTTCCAA ACTGGAACAA CACTCAACCC TATCTCGGTC TATTCTTTTG ATTTATAAGG
4741 GATTTTGCCG ATTTCGGCCT ATTGGTTAAA AAATGAGCTG ATTTAACAAA AATTTAACGC
4801 GAATTTTAAC AAAATATTAA CGCTTACAAT TTAGGTGGCA CTTTTCGGGG AAATGTGCGC
4861 GGAACCCCTA TTTGTTTATT TTTCTAAATA CATTCAAATA TGTATCCGCT CATGAGACAA
4921 TAACCCTGAT AAATGCTTCA ATAATATTGA AAAAGGAAGA GTATGAGTAT TCAACATTTC
4981 CGTGTCGCCC TTATTCCCTT TTTTGCGGCA TTTTGCCTTC CTGTTTTTGC TCACCCAGAA
5041 ACGCTGGTGA AAGTAAAAGA TGCTGAAGAT CAGTTGGGTG CACGAGTGGG TTACATCGAA
5101 CTGGATCTCA ACAGCGGTAA GATCCTTGAG AGTTTTCGCC CCGAAGAACG TTTTCCAATG
5161 ATGAGCACTT TTAAAGTTCT GCTATGTGGC GCGGTATTAT CCCGTATTGA CGCCGGGCAA
5221 GAGCAACTCG GTCGCCGCAT ACACTATTCT CAGAATGACT TGGTTGAGTA CTCACCAGTC
5281 ACAGAAAAGC ATCTTACGGA TGGCATGACA GTAAGAGAAT TATGCAGTGC TGCCATAACC
5341 ATGAGTGATA ACACTGCGGC CAACTTACTT CTGACAACGA TCGGAGGACC GAAGGAGCTA
5401 ACCGCTTTTT TGCACAACAT GGGGGATCAT GTAACTCGCC TTGATCGTTG GGAACCGGAG
5461 CTGAATGAAG CCATACCAAA CGACGAGCGT GACACCACGA TGCCTGTAGC AATGGCAACA
5521 ACGTTGCGCA AACTATTAAC TGGCGAACTA CTTACTCTAG CTTCCCGGCA ACAATTAATA
5581 GACTGGATGG AGGCGGATAA AGTTGCAGGA CCACTTCTGC GCTCGGCCCT TCCGGCTGGC
5641 TGGTTTATTG CTGATAAATC TGGAGCCGGT GAGCGTGGGT CTCGCGGTAT CATTGCAGCA
5701 CTGGGGCCAG ATGGTAAGCC CTCCCGTATC GTAGTTATCT ACACGACGGG GAGTCAGGCA
5761 ACTATGGATG AACGAAATAG ACAGATCGCT GAGATAGGTG CCTCACTGAT TAAGCATTGG
5821 TAACTGTCAG ACCAAGTTTA CTCATATATA CTTTAGATTG ATTTAAAACT TCATTTTTAA
5881 TTTAAAAGGA TCTAGGTGAA GATCCTTTTT GATAATCTCA TGACCAAAAT CCCTTAACGT
5941 GAGTTTTCGT TCCACTGAGC GTCAGACCCC GTAGAAAAGA TCAAAGGATC TTCTTGAGAT
6001 CCTTTTTTTC TGCGCGTAAT CTGCTGCTTG CAAACAAAAA AACCACCGCT ACCAGCGGTG
6061 GTTTGTTTGC CGGATCAAGA GCTACCAACT CTTTTTCCGA AGGTAACTGG CTTCAGCAGA
6121 GCGCAGATAC CAAATACTGT TCTTCTAGTG TAGCCGTAGT TAGGCCACCA CTTCAAGAAC
6181 TCTGTAGCAC CGCCTACATA CCTCGCTCTG CTAATCCTGT TACCAGTGGC TGCTGCCAGT
6241 GGCGATAAGT CGTGTCTTAC CGGGTTGGAC TCAAGACGAT AGTTACCGGA TAAGGCGCAG
6301 CGGTCGGGCT GAACGGGGGG TTCGTGCACA CAGCCCAGCT TGGAGCGAAC GACCTACACC
6361 GAACTGAGAT ACCTACAGCG TGAGCTATGA GAAAGCGCCA CGCTTCCCGA AGGGAGAAAG
6421 GCGGACAGGT ATCCGGTAAG CGGCAGGGTC GGAACAGGAG AGCGCACGAG GGAGCTTCCA
6481 GGGGGAAACG CCTGGTATCT TTATAGTCCT GTCGGGTTTC GCCACCTCTG ACTTGAGCGT
6541 CGATTTTTGT GATGCTCGTC AGGGGGGCGG AGCCTATGGA AAAACGCCAG CAACGCGGCC
6601 TTTTTACGGT TCCTGGCCTT TTGCTGGCCT TTTGCTCACA TGTTCTTTCC TGCGTTATCC
6661 CCTGATTCTG TGGATAACCG TATTACCGCC TTTGAGTGAG CTGATACCGC TCGCCGCAGC
6721 CGAACGACCG AGCGCAGCGA GTCAGTGAGC GAGGAAGCGG AAGAGCGCCC AATACGCAAA
6781 CCGCCTCTCC CCGCGCGTTG GCCGATTCAT TAATGCAGCT GGCACGACAG GTTTCCCGAC
6841 TGGAAAGCGG GCAGTGAGCG CAACGCAATT AATGTGAGTT AGCTCACTCA TTAGGCACCC
6901 CAGGCTTTAC ACTTTATGCT TCCGGCTCGT ATGTTGTGTG GAATTGTGAG CGGATAACAA
6961 TTTCACACAG GAAACAGCTA TGACCATGAT TACGCCAAGC GCGCAATTAA CCCTCACTAA
7021 AGGGAACAAA AGCTGGACTG CAAGCTT

Plasmid-map and sequence of the **Transposon U6 circ RAB7A LEAPER gRNA vector (pTS1896):**

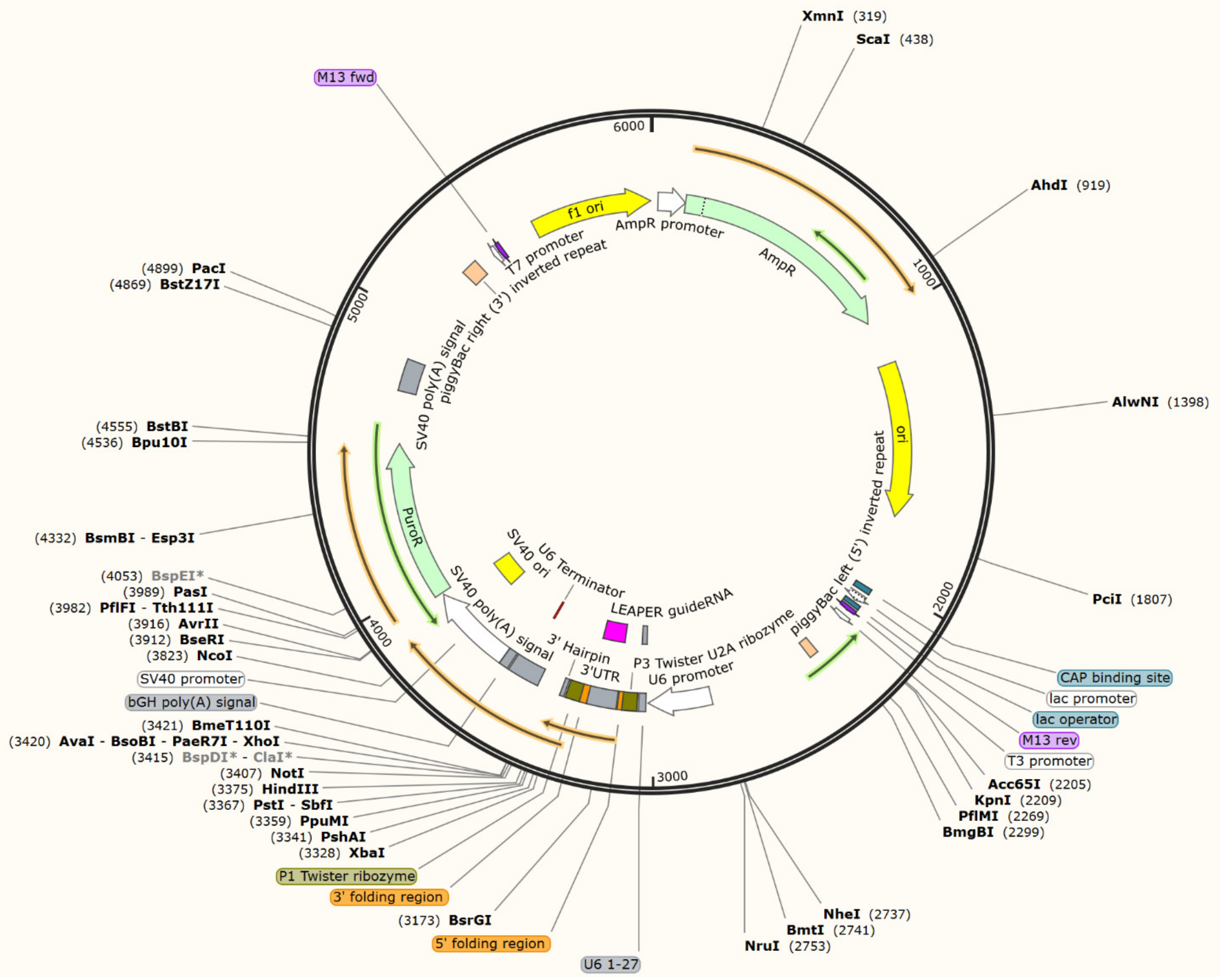

1 CAGGTGGCAC TTTTCGGGGA AATGTGCGCG GAACCCCTAT TTGTTTATTT TTCTAAATAC
61 ATTCAAATAT GTATCCGCTC ATGAGACAAT AACCCTGATA AATGCTTCAA TAATATTGAA
121 AAAGGAAGAG TATGAGTATT CAACATTTCC GTGTCGCCCT TATTCCCTTT TTTGCGGCAT
181 TTTGCCTTCC TGTTTTTGCT CACCCAGAAA CGCTGGTGAA AGTAAAAGAT GCTGAAGATC
241 AGTTGGGTGC ACGAGTGGGT TACATCGAAC TGGATCTCAA CAGCGGTAAG ATCCTTGAGA
301 GTTTTCGCCC CGAAGAACGT TTTCCAATGA TGAGCACTTT TAAAGTTCTG CTATGTGGCG
361 CGGTATTATC CCGTATTGAC GCCGGGCAAG AGCAACTCGG TCGCCGCATA CACTATTCTC
421 AGAATGACTT GGTTGAGTAC TCACCAGTCA CAGAAAAGCA TCTTACGGAT GGCATGACAG
481 TAAGAGAATT ATGCAGTGCT GCCATAACCA TGAGTGATAA CACTGCGGCC AACTTACTTC
541 TGACAACGAT CGGAGGACCG AAGGAGCTAA CCGCTTTTTT GCACAACATG GGGGATCATG
601 TAACTCGCCT TGATCGTTGG GAACCGGAGC TGAATGAAGC CATACCAAAC GACGAGCGTG
661 ACACCACGAT GCCTGTAGCA ATGGCAACAA CGTTGCGCAA ACTATTAACT GGCGAACTAC
721 TTACTCTAGC TTCCCGGCAA CAATTAATAG ACTGGATGGA GGCGGATAAA GTTGCAGGAC
781 CACTTCTGCG CTCGGCCCTT CCGGCTGGCT GGTTTATTGC TGATAAATCT GGAGCCGGTG
841 AGCGTGGGTC TCGCGGTATC ATTGCAGCAC TGGGGCCAGA TGGTAAGCCC TCCCGTATCG
901 TAGTTATCTA CACGACGGGG AGTCAGGCAA CTATGGATGA ACGAAATAGA CAGATCGCTG
961 AGATAGGTGC CTCACTGATT AAGCATTGGT AACTGTCAGA CCAAGTTTAC TCATATATAC
1021 TTTAGATTGA TTTAAAACTT CATTTTTAAT TTAAAAGGAT CTAGGTGAAG ATCCTTTTTG

1081 ATAATCTCAT GACCAAAATC CCTTAACGTG AGTTTTCGTT CCACTGAGCG TCAGACCCCG
1141 TAGAAAAGAT CAAAGGATCT TCTTGAGATC CTTTTTTTCT GCGCGTAATC TGCTGCTTGC
1201 AAACAAAAAA ACCACCGCTA CCAGCGGTGG TTTGTTTGCC GGATCAAGAG CTACCAACTC
1261 TTTTTCCGAA GGTAACTGGC TTCAGCAGAG CGCAGATACC AAATACTGTC CTTCTAGTGT
1321 AGCCGTAGTT AGGCCACCAC TTCAAGAACT CTGTAGCACC GCCTACATAC CTCGCTCTGC
1381 TAATCCTGTT ACCAGTGGCT GCTGCCAGTG GCGATAAGTC GTGTCTTACC GGGTTGGACT
1441 CAAGACGATA GTTACCGGAT AAGGCGCAGC GGTCGGGCTG AACGGGGGGT TCGTGCACAC
1501 AGCCCAGCTT GGAGCGAACG ACCTACACCG AACTGAGATA CCTACAGCGT GAGCTATGAG
1561 AAAGCGCCAC GCTTCCCGAA GGGAGAAAGG CGGACAGGTA TCCGGTAAGC GGCAGGGTCG
1621 GAACAGGAGA GCGCACGAGG GAGCTTCCAG GGGGAAACGC CTGGTATCTT TATAGTCCTG
1681 TCGGGTTTCG CCACCTCTGA CTTGAGCGTC GATTTTTGTG ATGCTCGTCA GGGGGGCGGA
1741 GCCTATGGAA AAACGCCAGC AACGCGGCCT TTTTACGGTT CCTGGCCTTT TGCTGGCCTT
1801 TTGCTCACAT GTTCTTTCCT GCGTTATCCC CTGATTCTGT GGATAACCGT ATTACCGCCT
1861 TTGAGTGAGC TGATACCGCT CGCCGCAGCC GAACGACCGA GCGCAGCGAG TCAGTGAGCG
1921 AGGAAGCGGA AGAGCGCCCA ATACGCAAAC CGCCTCTCCC CGCGCGTTGG CCGATTCATT
1981 AATGCAGCTG GCACGACAGG TTTCCCGACT GGAAAGCGGG CAGTGAGCGC AACGCAATTA
2041 ATGTGAGTTA GCTCACTCAT TAGGCACCCC AGGCTTTACA CTTTATGCTT CCGGCTCGTA
2101 TGTTGTGTGG AATTGTGAGC GGATAACAAT TTCACACAGG AAACAGCTAT GACCATGATT
2161 ACGCCAAGCT CGGAATTAAC CCTCACTAAA GGGAACAAAA GCTGGGTACC TCGCGCGACT
2221 TGGTTTGCCA TTCTTTAGCG CGCGTCGCGT CACACAGCTT GGCCACAATG TGGTTTTTGT
2281 CAAACGAAGA TTCTATGACG TGTTTAAAGT TTAGGTCGAG TAAAGCGCAA ATCTTTTTTA
2341 ACCCTAGAAA GATAGTCTGC GTAAAATTGA CGCATGCATT CTTGAAATAT TGCTCTCTCT
2401 TTCTAAATAG CGCGAATCCG TCGCTGTGCA TTTAGGACAT CTCAGTCGCC GCTTGGAGCT
2461 CCCGTGAGGC GTGCTTGTCA ATGCGGTAAG TGTCACTGAT TTTGAACTAT AACGACCGCG
2521 TGAGTCAAAA TGACGCATGA TTATCTTTTA CGTGACTTTT AAGATTTAAC TCATACGATA
2581 ATTATATTGT TATTTCATGT TCTACTTACG TGATAACTTA TTATATATAT ATTTTCTTGT
2641 TATAGATATC GTGACTAATA TATAATAAAA TGGGTAGTTC TTTAGACGAT GAGCATATCC
2701 TCTCTGCTCT TCTGCAAAGC GATGACGAGC TTGTTGGCTA GCGCGCTGCT TCGCGATGTA
2761 CGGGCCAGAT ATACGCGAGG GCCTATTTCC CATGATTCCT TCATATTTGC ATATACGATA
2821 CAAGGCTGTT AGAGAGATAA TTAGAATTAA TTTGACTGTA AACACAAAGA TATTAGTACA
2881 AAATACGTGA CGTAGAAAGT AATAATTTCT TGGGTAGTTT GCAGTTTTAA AATTATGTTT
2941 TAAAATGGAC TATCATATGC TTACCGTAAC TTGAAAGTAT TTCGATTTCT TGGCTTTATA
3001 TATCTTGTGG AAAGGACGAA ACACCGTGCT CGCTTCGGCA GCACATATAC TAGTCGACGC
3061 CATCAGTCGC CGGTCCCAAG CCCGGATAAA ATGGGAGGGG GCGGGAAACC GCCTAACCAT
3121 GCCGACTGAT GGCAGGTCTT TGATAAAAGG CGTACATAAT TCTTGTGTCT ACTGTACAGA
3181 ATACTGCCGC CAGCTGGATT TCCCAATTCT GAGTAACACT CTGCAATCCA AACAGGGTTC
3241 AACCCTCTGC CATCAGTCGG CGTGGACTGT AGAACACTGC CAATGCCGGT CCCAAGCCCG
3301 GATAAAAGTG GAGGGTACAG TCCACGCTCT AGAGCGGACT TCGGTCCGCT TTTTACTAGG
3361 ACCTGCAGGC ATGCAAGCTT GACGTCGGTT ACCGATATCC ATATGGCGGC CGCATCGATC
3421 TCGAGCCGCG GACTAGTAAC TTGTTTATTG CAGCTTATAA TGGTTACAAA TAAAGCAATA
3481 GCATCACAAA TTTCACAAAT AAAGCATTTT TTTCACTGCA TTCTAGTTGT GGTTTGTCCA
3541 AACTCATCAA TGTATCTTAT CGACCTAGCA GGCATGCTGG GGATGCGGTG GGCTCTATGG
3601 CGTGTGTCAG TTAGGGTGTG GAAAGTCCCC AGGCTCCCCA GCAGGCAGAA GTATGCAAAG
3661 CATGCATCTC AATTAGTCAG CAACCAGGTG TGGAAAGTCC CCAGGCTCCC CAGCAGGCAG
3721 AAGTATGCAA AGCATGCATC TCAATTAGTC AGCAACCATA GTCCCGCCCC TAACTCCGCC
3781 CATCCCGCCC CTAACTCCGC CCAGTTCCGC CCATTCTCCG CCCCATGGCT GACTAATTTT
3841 TTTTATTTAT GCAGAGGCCG AGGCCGCCTC TGCCTCTGAG CTATTCCAGA AGTAGTGAGG
3901 AGGCTTTTTT GGAGGCCTAG GCTTTTGCAA AAAGCTCCCA TGACCGAGTA CAAGCCCACG
3961 GTGCGCCTCG CCACCCGCGA CGACGTCCCC AGGGCCGTAC GCACCCTCGC CGCCGCGTTC
4021 GCCGACTACC CCGCCACGCG CCACACCGTC GATCCGGACC GCCACATCGA GCGGGTCACC
4081 GAGCTGCAAG AACTCTTCCT CACGCGCGTC GGGCTCGACA TCGGCAAGGT GTGGGTCGCG
4141 GACGACGGCG CCGCGGTGGC GGTCTGGACC ACGCCGGAGA GCGTCGAAGC GGGGGCGGTG
4201 TTCGCCGAGA TCGGCCCGCG CATGGCCGAG TTGAGCGGTT CCCGGCTGGC CGCGCAGCAA
4261 CAGATGGAAG GCCTCCTGGC GCCGCACCGG CCCAAGGAGC CCGCGTGGTT CCTGGCCACC
4321 GTCGGCGTCT CGCCCGACCA CCAGGGCAAG GGTCTGGGCA GCGCCGTCGT GCTCCCCGGA
4381 GTGGAGGCGG CCGAGCGCGC CGGGGTGCCC GCCTTCCTGG AGACCTCCGC GCCCCGCAAC
4441 CTCCCCTTCT ACGAGCGGCT CGGCTTCACC GTCACCGCCG ACGTCGAGGT GCCCGAAGGA

4501 CCGCGCACCT GGTGCATGAC CCGCAAGCCC GGTGCCTGAG CGGGACTCTG GGGTTCGAAA
4561 TGACCGACCA AGCGACGCCC GAAATGACCG ACCAAGCGAC GCCCAACCTG CCATCACGAG
4621 ATTTCGATTC CACCGCCGCC TTCTATGAAA GGTTGGGCTT CGGAATCGTT TTCCGGGACG
4681 CCGGCTGGAT GATCCTCCAG CGCGGGGATC TCATGCTGGA GTTCTTCGCC CACCCCAACT
4741 TGTTTATTGC AGCTTATAAT GGTTACAAAT AAAGCAATAG CATCACAAAT TTCACAAATA
4801 AAGCATTTTT TTCACTGCAT TCTAGTTGTG GTTTGTCCAA ACTCATCAAT GTATCTTATC
4861 ATGTCTGTAT ACCGTCGACC TCTAGCTAGT CGAGTTAATT AACGAGAGCA TAATATTGAT
4921 ATGTGCCAAA GTTGTTTCTG ACTGACTAAT AAGTATAATT TGTTTCTATT ATGTATAGGT
4981 TAAGCTAATT ACTTATTTTA TAATACAACA TGACTGTTTT TAAAGTACAA AATAAGTTTA
5041 TTTTTGTAAA AGAGAGAATG TTTAAAAGTT TTGTTACTTT ATAGAAGAAA TTTTGAGTTT
5101 TTGTTTTTTT TTAATAAATA AATAAACATA AATAAATTGT TTGTTGAATT TATTATTAGT
5161 ATGTAAGTGT AAATATAATA AAACTTAATA TCTATTCAAA TTAATAAATA AACCTCGATA
5221 TACAGACCGA TAAAACACAT GCGTCAATTT TACGCATGAT TATCTTTAAC GTACGTCACA
5281 ATATGATTAT CTTTCTAGGG TTAAATAATA GTTTCTAATT TTTTTATTAT TCAGCCTGCT
5341 GTCGTGAATA CCGAGCTCCA ATTCGCCCTA TAGTGAGTCG TATTACAATT CACTGGCCGT
5401 CGTTTTACAA CGTCGTGACT GGGAAAACCC TGGCGTTACC CAACTTAATC GCCTTGCAGC
5461 ACATCCCCCT TTCGCCAGCT GGCGTAATAG CGAAGAGGCC CGCACCGATC GCCCTTCCCA
5521 ACAGTTGCGC AGCCTGAATG GCGAATGGCG CGACGCGCCC TGTAGCGGCG CATTAAGCGC
5581 GGCGGGTGTG GTGGTTACGC GCAGCGTGAC CGCTACACTT GCCAGCGCCC TAGCGCCCGC
5641 TCCTTTCGCT TTCTTCCCTT CCTTTCTCGC CACGTTCGCC GGCTTTCCCC GTCAAGCTCT
5701 AAATCGGGGG CTCCCTTTAG GGTTCCGATT TAGTGCTTTA CGGCACCTCG ACCCCAAAAA
5761 ACTTGATTAG GGTGATGGTT CACGTAGTGG GCCATCGCCC TGATAGACGG TTTTTCGCCC
5821 TTTGACGTTG GAGTCCACGT TCTTTAATAG TGGACTCTTG TTCCAAACTG GAACAACACT
5881 CAACCCTATC TCGGTCTATT CTTTTGATTT ATAAGGGATT TTGCCGATTT CGGCCTATTG
5941 GTTAAAAAAT GAGCTGATTT AACAAAAATT TAACGCGAAT TTTAACAAAA TATTAACGTT
6001 TACAATTTCC

Plasmid-map and sequence of the **Transposon U6 linear RAB7A LEAPER gRNA vector (pTS1897):**

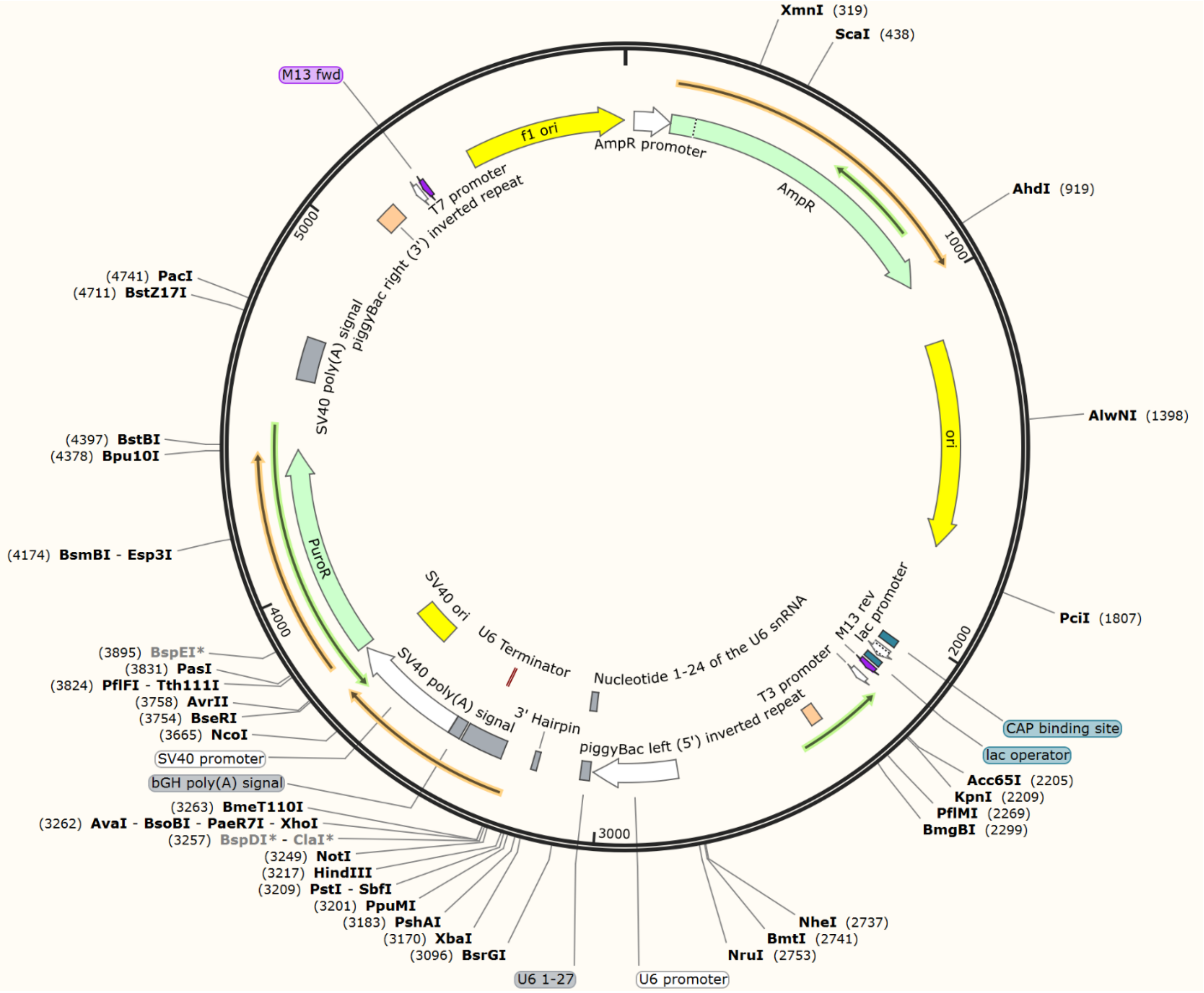

1 CAGGTGGCAC TTTTCGGGGA AATGTGCGCG GAACCCCTAT TTGTTTATTT TTCTAAATAC
61 ATTCAAATAT GTATCCGCTC ATGAGACAAT AACCCTGATA AATGCTTCAA TAATATTGAA
121 AAAGGAAGAG TATGAGTATT CAACATTTCC GTGTCGCCCT TATTCCCTTT TTTGCGGCAT
181 TTTGCCTTCC TGTTTTTGCT CACCCAGAAA CGCTGGTGAA AGTAAAAGAT GCTGAAGATC
241 AGTTGGGTGC ACGAGTGGGT TACATCGAAC TGGATCTCAA CAGCGGTAAG ATCCTTGAGA
301 GTTTTCGCCC CGAAGAACGT TTTCCAATGA TGAGCACTTT TAAAGTTCTG CTATGTGGCG
361 CGGTATTATC CCGTATTGAC GCCGGGCAAG AGCAACTCGG TCGCCGCATA CACTATTCTC
421 AGAATGACTT GGTTGAGTAC TCACCAGTCA CAGAAAAGCA TCTTACGGAT GGCATGACAG
481 TAAGAGAATT ATGCAGTGCT GCCATAACCA TGAGTGATAA CACTGCGGCC AACTTACTTC
541 TGACAACGAT CGGAGGACCG AAGGAGCTAA CCGCTTTTTT GCACAACATG GGGGATCATG
601 TAACTCGCCT TGATCGTTGG GAACCGGAGC TGAATGAAGC CATACCAAAC GACGAGCGTG
661 ACACCACGAT GCCTGTAGCA ATGGCAACAA CGTTGCGCAA ACTATTAACT GGCGAACTAC
721 TTACTCTAGC TTCCCGGCAA CAATTAATAG ACTGGATGGA GGCGGATAAA GTTGCAGGAC
781 CACTTCTGCG CTCGGCCCTT CCGGCTGGCT GGTTTATTGC TGATAAATCT GGAGCCGGTG
841 AGCGTGGGTC TCGCGGTATC ATTGCAGCAC TGGGGCCAGA TGGTAAGCCC TCCCGTATCG
901 TAGTTATCTA CACGACGGGG AGTCAGGCAA CTATGGATGA ACGAAATAGA CAGATCGCTG
961 AGATAGGTGC CTCACTGATT AAGCATTGGT AACTGTCAGA CCAAGTTTAC TCATATATAC
1021 TTTAGATTGA TTTAAAACTT CATTTTTAAT TTAAAAGGAT CTAGGTGAAG ATCCTTTTTG

1081 ATAATCTCAT GACCAAAATC CCTTAACGTG AGTTTTCGTT CCACTGAGCG TCAGACCCCG
1141 TAGAAAAGAT CAAAGGATCT TCTTGAGATC CTTTTTTTCT GCGCGTAATC TGCTGCTTGC
1201 AAACAAAAAA ACCACCGCTA CCAGCGGTGG TTTGTTTGCC GGATCAAGAG CTACCAACTC
1261 TTTTTCCGAA GGTAACTGGC TTCAGCAGAG CGCAGATACC AAATACTGTC CTTCTAGTGT
1321 AGCCGTAGTT AGGCCACCAC TTCAAGAACT CTGTAGCACC GCCTACATAC CTCGCTCTGC
1381 TAATCCTGTT ACCAGTGGCT GCTGCCAGTG GCGATAAGTC GTGTCTTACC GGGTTGGACT
1441 CAAGACGATA GTTACCGGAT AAGGCGCAGC GGTCGGGCTG AACGGGGGGT TCGTGCACAC
1501 AGCCCAGCTT GGAGCGAACG ACCTACACCG AACTGAGATA CCTACAGCGT GAGCTATGAG
1561 AAAGCGCCAC GCTTCCCGAA GGGAGAAAGG CGGACAGGTA TCCGGTAAGC GGCAGGGTCG
1621 GAACAGGAGA GCGCACGAGG GAGCTTCCAG GGGGAAACGC CTGGTATCTT TATAGTCCTG
1681 TCGGGTTTCG CCACCTCTGA CTTGAGCGTC GATTTTTGTG ATGCTCGTCA GGGGGGCGGA
1741 GCCTATGGAA AAACGCCAGC AACGCGGCCT TTTTACGGTT CCTGGCCTTT TGCTGGCCTT
1801 TTGCTCACAT GTTCTTTCCT GCGTTATCCC CTGATTCTGT GGATAACCGT ATTACCGCCT
1861 TTGAGTGAGC TGATACCGCT CGCCGCAGCC GAACGACCGA GCGCAGCGAG TCAGTGAGCG
1921 AGGAAGCGGA AGAGCGCCCA ATACGCAAAC CGCCTCTCCC CGCGCGTTGG CCGATTCATT
1981 AATGCAGCTG GCACGACAGG TTTCCCGACT GGAAAGCGGG CAGTGAGCGC AACGCAATTA
2041 ATGTGAGTTA GCTCACTCAT TAGGCACCCC AGGCTTTACA CTTTATGCTT CCGGCTCGTA
2101 TGTTGTGTGG AATTGTGAGC GGATAACAAT TTCACACAGG AAACAGCTAT GACCATGATT
2161 ACGCCAAGCT CGGAATTAAC CCTCACTAAA GGGAACAAAA GCTGGGTACC TCGCGCGACT
2221 TGGTTTGCCA TTCTTTAGCG CGCGTCGCGT CACACAGCTT GGCCACAATG TGGTTTTTGT
2281 CAAACGAAGA TTCTATGACG TGTTTAAAGT TTAGGTCGAG TAAAGCGCAA ATCTTTTTTA
2341 ACCCTAGAAA GATAGTCTGC GTAAAATTGA CGCATGCATT CTTGAAATAT TGCTCTCTCT
2401 TTCTAAATAG CGCGAATCCG TCGCTGTGCA TTTAGGACAT CTCAGTCGCC GCTTGGAGCT
2461 CCCGTGAGGC GTGCTTGTCA ATGCGGTAAG TGTCACTGAT TTTGAACTAT AACGACCGCG
2521 TGAGTCAAAA TGACGCATGA TTATCTTTTA CGTGACTTTT AAGATTTAAC TCATACGATA
2581 ATTATATTGT TATTTCATGT TCTACTTACG TGATAACTTA TTATATATAT ATTTTCTTGT
2641 TATAGATATC GTGACTAATA TATAATAAAA TGGGTAGTTC TTTAGACGAT GAGCATATCC
2701 TCTCTGCTCT TCTGCAAAGC GATGACGAGC TTGTTGGCTA GCGCGCTGCT TCGCGATGTA
2761 CGGGCCAGAT ATACGCGAGG GCCTATTTCC CATGATTCCT TCATATTTGC ATATACGATA
2821 CAAGGCTGTT AGAGAGATAA TTAGAATTAA TTTGACTGTA AACACAAAGA TATTAGTACA
2881 AAATACGTGA CGTAGAAAGT AATAATTTCT TGGGTAGTTT GCAGTTTTAA AATTATGTTT
2941 TAAAATGGAC TATCATATGC TTACCGTAAC TTGAAAGTAT TTCGATTTCT TGGCTTTATA
3001 TATCTTGTGG AAAGGACGAA ACACCGTGCT CGCTTCGGCA GCACATATAC TAGTCGACGT
3061 CTTTGATAAA AGGCGTACAT AATTCTTGTG TCTACTGTAC AGAATACTGC CGCCAGCTGG
3121 ATTTCCCAAT TCTGAGTAAC ACTCTGCAAT CCAAACAGGG TTCAACCCTT CTAGAGCGGA
3181 CTTCGGTCCG CTTTTTACTA GGACCTGCAG GCATGCAAGC TTGACGTCGG TTACCGATAT
3241 CCATATGGCG GCCGCATCGA TCTCGAGCCG CGGACTAGTA ACTTGTTTAT TGCAGCTTAT
3301 AATGGTTACA AATAAAGCAA TAGCATCACA AATTTCACAA ATAAAGCATT TTTTTCACTG
3361 CATTCTAGTT GTGGTTTGTC CAAACTCATC AATGTATCTT ATCGACCTAG CAGGCATGCT
3421 GGGGATGCGG TGGGCTCTAT GGCGTGTGTC AGTTAGGGTG TGGAAAGTCC CCAGGCTCCC
3481 CAGCAGGCAG AAGTATGCAA AGCATGCATC TCAATTAGTC AGCAACCAGG TGTGGAAAGT
3541 CCCCAGGCTC CCCAGCAGGC AGAAGTATGC AAAGCATGCA TCTCAATTAG TCAGCAACCA
3601 TAGTCCCGCC CCTAACTCCG CCCATCCCGC CCCTAACTCC GCCCAGTTCC GCCCATTCTC
3661 CGCCCCATGG CTGACTAATT TTTTTTATTT ATGCAGAGGC CGAGGCCGCC TCTGCCTCTG
3721 AGCTATTCCA GAAGTAGTGA GGAGGCTTTT TTGGAGGCCT AGGCTTTTGC AAAAAGCTCC
3781 CATGACCGAG TACAAGCCCA CGGTGCGCCT CGCCACCCGC GACGACGTCC CCAGGGCCGT
3841 ACGCACCCTC GCCGCCGCGT TCGCCGACTA CCCCGCCACG CGCCACACCG TCGATCCGGA
3901 CCGCCACATC GAGCGGGTCA CCGAGCTGCA AGAACTCTTC CTCACGCGCG TCGGGCTCGA
3961 CATCGGCAAG GTGTGGGTCG CGGACGACGG CGCCGCGGTG GCGGTCTGGA CCACGCCGGA
4021 GAGCGTCGAA GCGGGGGCGG TGTTCGCCGA GATCGGCCCG CGCATGGCCG AGTTGAGCGG
4081 TTCCCGGCTG GCCGCGCAGC AACAGATGGA AGGCCTCCTG GCGCCGCACC GGCCCAAGGA
4141 GCCCGCGTGG TTCCTGGCCA CCGTCGGCGT CTCGCCCGAC CACCAGGGCA AGGGTCTGGG
4201 CAGCGCCGTC GTGCTCCCCG GAGTGGAGGC GGCCGAGCGC GCCGGGGTGC CCGCCTTCCT
4261 GGAGACCTCC GCGCCCCGCA ACCTCCCCTT CTACGAGCGG CTCGGCTTCA CCGTCACCGC
4321 CGACGTCGAG GTGCCCGAAG GACCGCGCAC CTGGTGCATG ACCCGCAAGC CCGGTGCCTG
4381 AGCGGGACTC TGGGGTTCGA AATGACCGAC CAAGCGACGC CCGAAATGAC CGACCAAGCG
4441 ACGCCCAACC TGCCATCACG AGATTTCGAT TCCACCGCCG CCTTCTATGA AAGGTTGGGC

4501 TTCGGAATCG TTTTCCGGGA CGCCGGCTGG ATGATCCTCC AGCGCGGGGA TCTCATGCTG
4561 GAGTTCTTCG CCCACCCCAA CTTGTTTATT GCAGCTTATA ATGGTTACAA ATAAAGCAAT
4621 AGCATCACAA ATTTCACAAA TAAAGCATTT TTTTCACTGC ATTCTAGTTG TGGTTTGTCC
4681 AAACTCATCA ATGTATCTTA TCATGTCTGT ATACCGTCGA CCTCTAGCTA GTCGAGTTAA
4741 TTAACGAGAG CATAATATTG ATATGTGCCA AAGTTGTTTC TGACTGACTA ATAAGTATAA
4801 TTTGTTTCTA TTATGTATAG GTTAAGCTAA TTACTTATTT TATAATACAA CATGACTGTT
4861 TTTAAAGTAC AAAATAAGTT TATTTTTGTA AAAGAGAGAA TGTTTAAAAG TTTTGTTACT
4921 TTATAGAAGA AATTTTGAGT TTTTGTTTTT TTTTAATAAA TAAATAAACA TAAATAAATT
4981 GTTTGTTGAA TTTATTATTA GTATGTAAGT GTAAATATAA TAAAACTTAA TATCTATTCA
5041 AATTAATAAA TAAACCTCGA TATACAGACC GATAAAACAC ATGCGTCAAT TTTACGCATG
5101 ATTATCTTTA ACGTACGTCA CAATATGATT ATCTTTCTAG GGTTAAATAA TAGTTTCTAA
5161 TTTTTTTATT ATTCAGCCTG CTGTCGTGAA TACCGAGCTC CAATTCGCCC TATAGTGAGT
5221 CGTATTACAA TTCACTGGCC GTCGTTTTAC AACGTCGTGA CTGGGAAAAC CCTGGCGTTA
5281 CCCAACTTAA TCGCCTTGCA GCACATCCCC CTTTCGCCAG CTGGCGTAAT AGCGAAGAGG
5341 CCCGCACCGA TCGCCCTTCC CAACAGTTGC GCAGCCTGAA TGGCGAATGG CGCGACGCGC
5401 CCTGTAGCGG CGCATTAAGC GCGGCGGGTG TGGTGGTTAC GCGCAGCGTG ACCGCTACAC
5461 TTGCCAGCGC CCTAGCGCCC GCTCCTTTCG CTTTCTTCCC TTCCTTTCTC GCCACGTTCG
5521 CCGGCTTTCC CCGTCAAGCT CTAAATCGGG GGCTCCCTTT AGGGTTCCGA TTTAGTGCTT
5581 TACGGCACCT CGACCCCAAA AAACTTGATT AGGGTGATGG TTCACGTAGT GGGCCATCGC
5641 CCTGATAGAC GGTTTTTCGC CCTTTGACGT TGGAGTCCAC GTTCTTTAAT AGTGGACTCT
5701 TGTTCCAAAC TGGAACAACA CTCAACCCTA TCTCGGTCTA TTCTTTTGAT TTATAAGGGA
5761 TTTTGCCGAT TTCGGCCTAT TGGTTAAAAA ATGAGCTGAT TTAACAAAAA TTTAACGCGA
5821 ATTTTAACAA AATATTAACG TTTACAATTT CC

Plasmid-map and sequence of the **Transposon EF1α circ RAB7A LEAPER gRNA vector (pTS1899):**

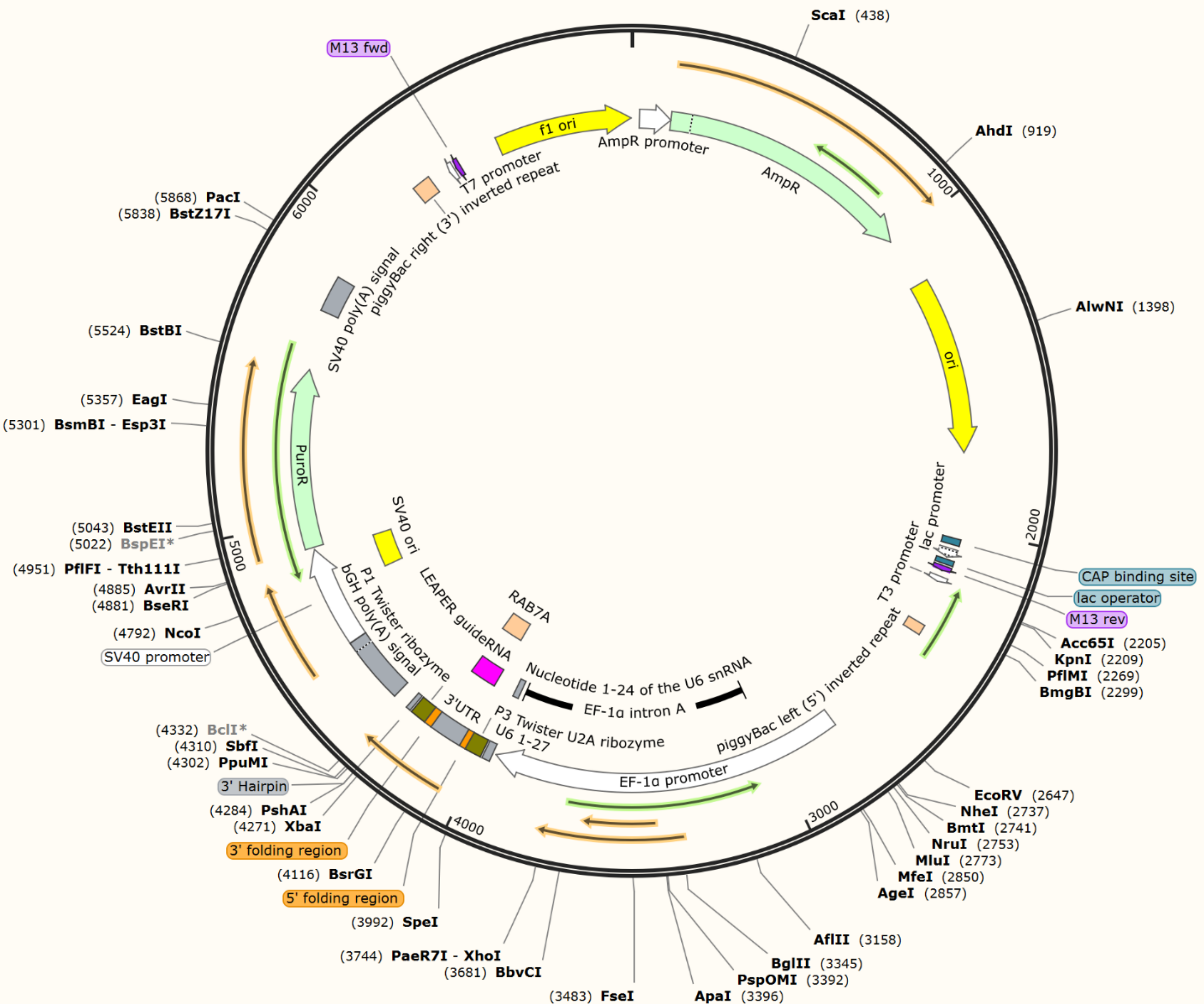

1	CAGGTGGCAC	TTTTCGGGGA	AATGTGCGCG	GAACCCCTAT	TTGTTTATTT	TTCTAAATAC
61	ATTCAAATAT	GTATCCGCTC	ATGAGACAAT	AACCCTGATA	AATGCTTCAA	TAATATTGAA
121	AAAGGAAGAG	TATGAGTATT	CAACATTTCC	GTGTCGCCCT	TATTCCCTTT	TTTGCGGCAT
181	TTTGCCTTCC	TGTTTTTGCT	CACCCAGAAA	CGCTGGTGAA	AGTAAAAGAT	GCTGAAGATC
241	AGTTGGGTGC	ACGAGTGGGT	TACATCGAAC	TGGATCTCAA	CAGCGGTAAG	ATCCTTGAGA
301	GTTTTCGCCC	CGAAGAACGT	TTTCCAATGA	TGAGCACTTT	TAAAGTTCTG	CTATGTGGCG
361	CGGTATTATC	CCGTATTGAC	GCCGGGCAAG	AGCAACTCGG	TCGCCGCATA	CACTATTCTC
421	AGAATGACTT	GGTTGAGTAC	TCACCAGTCA	CAGAAAAGCA	TCTTACGGAT	GGCATGACAG
481	TAAGAGAATT	ATGCAGTGCT	GCCATAACCA	TGAGTGATAA	CACTGCGGCC	AACTTACTTC
541	TGACAACGAT	CGGAGGACCG	AAGGAGCTAA	CCGCTTTTTT	GCACAACATG	GGGGATCATG
601	TAACTCGCCT	TGATCGTTGG	GAACCGGAGC	TGAATGAAGC	CATACCAAAC	GACGAGCGTG
661	ACACCACGAT	GCCTGTAGCA	ATGGCAACAA	CGTTGCGCAA	ACTATTAACT	GGCGAACTAC
721	TTACTCTAGC	TTCCCGGCAA	CAATTAATAG	ACTGGATGGA	GGCGGATAAA	GTTGCAGGAC
781	CACTTCTGCG	CTCGGCCCTT	CCGGCTGGCT	GGTTTATTGC	TGATAAATCT	GGAGCCGGTG
841	AGCGTGGGTC	TCGCGGTATC	ATTGCAGCAC	TGGGGCCAGA	TGGTAAGCCC	TCCCGTATCG
901	TAGTTATCTA	CACGACGGGG	AGTCAGGCAA	CTATGGATGA	ACGAAATAGA	CAGATCGCTG
961	AGATAGGTGC	CTCACTGATT	AAGCATTGGT	AACTGTCAGA	CCAAGTTTAC	TCATATATAC

1021 TTTAGATTGA TTTAAAACTT CATTTTTAAT TTAAAAGGAT CTAGGTGAAG ATCCTTTTTG
1081 ATAATCTCAT GACCAAAATC CCTTAACGTG AGTTTTCGTT CCACTGAGCG TCAGACCCCG
1141 TAGAAAAGAT CAAAGGATCT TCTTGAGATC CTTTTTTTCT GCGCGTAATC TGCTGCTTGC
1201 AAACAAAAAA ACCACCGCTA CCAGCGGTGG TTTGTTTGCC GGATCAAGAG CTACCAACTC
1261 TTTTTCCGAA GGTAACTGGC TTCAGCAGAG CGCAGATACC AAATACTGTC CTTCTAGTGT
1321 AGCCGTAGTT AGGCCACCAC TTCAAGAACT CTGTAGCACC GCCTACATAC CTCGCTCTGC
1381 TAATCCTGTT ACCAGTGGCT GCTGCCAGTG GCGATAAGTC GTGTCTTACC GGGTTGGACT
1441 CAAGACGATA GTTACCGGAT AAGGCGCAGC GGTCGGGCTG AACGGGGGGT TCGTGCACAC
1501 AGCCCAGCTT GGAGCGAACG ACCTACACCG AACTGAGATA CCTACAGCGT GAGCTATGAG
1561 AAAGCGCCAC GCTTCCCGAA GGGAGAAAGG CGGACAGGTA TCCGGTAAGC GGCAGGGTCG
1621 GAACAGGAGA GCGCACGAGG GAGCTTCCAG GGGGAAACGC CTGGTATCTT TATAGTCCTG
1681 TCGGGTTTCG CCACCTCTGA CTTGAGCGTC GATTTTTGTG ATGCTCGTCA GGGGGGCGGA
1741 GCCTATGGAA AAACGCCAGC AACGCGGCCT TTTTACGGTT CCTGGCCTTT TGCTGGCCTT
1801 TTGCTCACAT GTTCTTTCCT GCGTTATCCC CTGATTCTGT GGATAACCGT ATTACCGCCT
1861 TTGAGTGAGC TGATACCGCT CGCCGCAGCC GAACGACCGA GCGCAGCGAG TCAGTGAGCG
1921 AGGAAGCGGA AGAGCGCCCA ATACGCAAAC CGCCTCTCCC CGCGCGTTGG CCGATTCATT
1981 AATGCAGCTG GCACGACAGG TTTCCCGACT GGAAAGCGGG CAGTGAGCGC AACGCAATTA
2041 ATGTGAGTTA GCTCACTCAT TAGGCACCCC AGGCTTTACA CTTTATGCTT CCGGCTCGTA
2101 TGTTGTGTGG AATTGTGAGC GGATAACAAT TTCACACAGG AAACAGCTAT GACCATGATT
2161 ACGCCAAGCT CGGAATTAAC CCTCACTAAA GGGAACAAAA GCTGGGTACC TCGCGCGACT
2221 TGGTTTGCCA TTCTTTAGCG CGCGTCGCGT CACACAGCTT GGCCACAATG TGGTTTTTGT
2281 CAAACGAAGA TTCTATGACG TGTTTAAAGT TTAGGTCGAG TAAAGCGCAA ATCTTTTTTA
2341 ACCCTAGAAA GATAGTCTGC GTAAAATTGA CGCATGCATT CTTGAAATAT TGCTCTCTCT
2401 TTCTAAATAG CGCGAATCCG TCGCTGTGCA TTTAGGACAT CTCAGTCGCC GCTTGGAGCT
2461 CCCGTGAGGC GTGCTTGTCA ATGCGGTAAG TGTCACTGAT TTTGAACTAT AACGACCGCG
2521 TGAGTCAAAA TGACGCATGA TTATCTTTTA CGTGACTTTT AAGATTTAAC TCATACGATA
2581 ATTATATTGT TATTTCATGT TCTACTTACG TGATAACTTA TTATATATAT ATTTTCTTGT
2641 TATAGATATC GTGACTAATA TATAATAAAA TGGGTAGTTC TTTAGACGAT GAGCATATCC
2701 TCTCTGCTCT TCTGCAAAGC GATGACGAGC TTGTTGGCTA GCGCGCTGCT TCGCGATGTA
2761 CGGGCCAGAT ATACGCGTGG CTCCGGTGCC CGTCAGTGGG CAGAGCGCAC ATCGCCCACA
2821 GTCCCCGAGA AGTTGGGGGG AGGGGTCGGC AATTGAACCG GTGCCTAGAG AAGGTGGCGC
2881 GGGGTAAACT GGGAAAGTGA TGTCGTGTAC TGGCTCCGCC TTTTTCCCGA GGGTGGGGGA
2941 GAACCGTATA TAAGTGCAGT AGTCGCCGTG AACGTTCTTT TTCGCAACGG GTTTGCCGCC
3001 AGAACACAGG TAAGTGCCGT GTGTGGTTCC CGCGGGCCTG GCCTCTTTAC GGGTTATGGC
3061 CCTTGCGTGC CTTGAATTAC TTCCACCTGG CTGCAGTACG TGATTCTTGA TCCCGAGCTT
3121 CGGGTTGGAA GTGGGTGGGA GAGTTCGAGG CCTTGCGCTT AAGGAGCCCC TTCGCCTCGT
3181 GCTTGAGTTG AGGCCTGGCC TGGGCGCTGG GGCCGCCGCG TGCGAATCTG GTGGCACCTT
3241 CGCGCCTGTC TCGCTGCTTT CGATAAGTCT CTAGCCATTT AAAATTTTTG ATGACCTGCT
3301 GCGACGCTTT TTTTCTGGCA AGATAGTCTT GTAAATGCGG GCCAAGATCT GCACACTGGT
3361 ATTTCGGTTT TTGGGGCCGC GGGCGGCGAC GGGGCCCGTG CGTCCCAGCG CACATGTTCG
3421 GCGAGGCGGG GCCTGCGAGC GCGGCCACCG AGAATCGGAC GGGGGTAGTC TCAAGCTGGC
3481 CGGCCTGCTC TGGTGCCTGG CCTCGCGCCG CCGTGTATCG CCCCGCCCTG GGCGGCAAGG
3541 CTGGCCCGGT CGGCACCAGT TGCGTGAGCG GAAAGATGGC CGCTTCCCGG CCCTGCTGCA
3601 GGGAGCTCAA AATGGAGGAC GCGGCGCTCG GGAGAGCGGG CGGGTGAGTC ACCCACACAA
3661 AGGAAAAGGG CCTTTCCGTC CTCAGCCGTC GCTTCATGTG ACTCCACGGA GTACCGGGCG
3721 CCGTCCAGGC ACCTCGATTA GTTCTCGAGC TTTTGGAGTA CGTCGTCTTT AGGTTGGGGG
3781 GAGGGGTTTT ATGCGATGGA GTTTCCCCAC ACTGAGTGGG TGGAGACTGA AGTTAGGCCA
3841 GCTTGGCACT TGATGTAATT CTCCTTGGAA TTTGCCCTTT TTGAGTTTGG ATCTTGGTTC
3901 ATTCTCAAGC CTCAGACAGT GGTTCAAAGT TTTTTTCTTC CATTTCAGGT GTCGTGAGGT
3961 CTAGCACCGT GCTCGCTTCG GCAGCACATA TACTAGTCGA CGCCATCAGT CGCCGGTCCC
4021 AAGCCCGGAT AAAATGGGAG GGGGCGGGAA ACCGCCTAAC CATGCCGACT GATGGCAGGT
4081 CTTTGATAAA AGGCGTACAT AATTCTTGTG TCTACTGTAC AGAATACTGC CGCCAGCTGG
4141 ATTTCCCAAT TCTGAGTAAC ACTCTGCAAT CCAAACAGGG TTCAACCCTC TGCCATCAGT
4201 CGGCGTGGAC TGTAGAACAC TGCCAATGCC GGTCCCAAGC CCGGATAAAA GTGGAGGGTA
4261 CAGTCCACGC TCTAGAGCGG ACTTCGGTCC GCTTTTTACT AGGACCTGCA GGCATGCTCG
4321 ACAGAGCTCG CTGATCAGCC TCGACTGTGC CTTCTAGTTG CCAGCCATCT GTTGTTTGCC
4381 CCTCCCCCGT GCCTTCCTTG ACCCTGGAAG GTGCCACTCC CACTGTCCTT TCCTAATAAA

4441 ATGAGGAAAT TGCATCGCAT TGTCTGAGTA GGTGTCATTC TATTCTGGGG GGTGGGGTGG
4501 GGCAGGACAG CAAGGGGGAG GATTGGGAAG ATAATAGCAG GCATGCTGGG GATGCGGTGG
4561 GCTCTATGGC GTGTGTCAGT TAGGGTGTGG AAAGTCCCCA GGCTCCCCAG CAGGCAGAAG
4621 TATGCAAAGC ATGCATCTCA ATTAGTCAGC AACCAGGTGT GGAAAGTCCC CAGGCTCCCC
4681 AGCAGGCAGA AGTATGCAAA GCATGCATCT CAATTAGTCA GCAACCATAG TCCCGCCCCT
4741 AACTCCGCCC ATCCCGCCCC TAACTCCGCC CAGTTCCGCC CATTCTCCGC CCCATGGCTG
4801 ACTAATTTTT TTTATTTATG CAGAGGCCGA GGCCGCCTCT GCCTCTGAGC TATTCCAGAA
4861 GTAGTGAGGA GGCTTTTTTG GAGGCCTAGG CTTTTGCAAA AAGCTCCCAT GACCGAGTAC
4921 AAGCCCACGG TGCGCCTCGC CACCCGCGAC GACGTCCCCA GGGCCGTACG CACCCTCGCC
4981 GCCGCGTTCG CCGACTACCC CGCCACGCGC CACACCGTCG ATCCGGACCG CCACATCGAG
5041 CGGGTCACCG AGCTGCAAGA ACTCTTCCTC ACGCGCGTCG GGCTCGACAT CGGCAAGGTG
5101 TGGGTCGCGG ACGACGGCGC CGCGGTGGCG GTCTGGACCA CGCCGGAGAG CGTCGAAGCG
5161 GGGGCGGTGT TCGCCGAGAT CGGCCCGCGC ATGGCCGAGT TGAGCGGTTC CCGGCTGGCC
5221 GCGCAGCAAC AGATGGAAGG CCTCCTGGCG CCGCACCGGC CCAAGGAGCC CGCGTGGTTC
5281 CTGGCCACCG TCGGCGTCTC GCCCGACCAC CAGGGCAAGG GTCTGGGCAG CGCCGTCGTG
5341 CTCCCCGGAG TGGAGGCGGC CGAGCGCGCC GGGGTGCCCG CCTTCCTGGA GACCTCCGCG
5401 CCCCGCAACC TCCCCTTCTA CGAGCGGCTC GGCTTCACCG TCACCGCCGA CGTCGAGGTG
5461 CCCGAAGGAC CGCGCACCTG GTGCATGACC CGCAAGCCCG GTGCCTGAGC GGGACTCTGG
5521 GGTTCGAAAT GACCGACCAA GCGACGCCCG AAATGACCGA CCAAGCGACG CCCAACCTGC
5581 CATCACGAGA TTTCGATTCC ACCGCCGCCT TCTATGAAAG GTTGGGCTTC GGAATCGTTT
5641 TCCGGGACGC CGGCTGGATG ATCCTCCAGC GCGGGGATCT CATGCTGGAG TTCTTCGCCC
5701 ACCCCAACTT GTTTATTGCA GCTTATAATG GTTACAAATA AAGCAATAGC ATCACAAATT
5761 TCACAAATAA AGCATTTTTT TCACTGCATT CTAGTTGTGG TTTGTCCAAA CTCATCAATG
5821 TATCTTATCA TGTCTGTATA CCGTCGACCT CTAGCTAGTC GAGTTAATTA ACGAGAGCAT
5881 AATATTGATA TGTGCCAAAG TTGTTTCTGA CTGACTAATA AGTATAATTT GTTTCTATTA
5941 TGTATAGGTT AAGCTAATTA CTTATTTTAT AATACAACAT GACTGTTTTT AAAGTACAAA
6001 ATAAGTTTAT TTTTGTAAAA GAGAGAATGT TTAAAAGTTT TGTTACTTTA TAGAAGAAAT
6061 TTTGAGTTTT TGTTTTTTTT TAATAAATAA ATAAACATAA ATAAATTGTT TGTTGAATTT
6121 ATTATTAGTA TGTAAGTGTA AATATAATAA AACTTAATAT CTATTCAAAT TAATAAATAA
6181 ACCTCGATAT ACAGACCGAT AAAACACATG CGTCAATTTT ACGCATGATT ATCTTTAACG
6241 TACGTCACAA TATGATTATC TTTCTAGGGT TAAATAATAG TTTCTAATTT TTTTATTATT
6301 CAGCCTGCTG TCGTGAATAC CGAGCTCCAA TTCGCCCTAT AGTGAGTCGT ATTACAATTC
6361 ACTGGCCGTC GTTTTACAAC GTCGTGACTG GGAAAACCCT GGCGTTACCC AACTTAATCG
6421 CCTTGCAGCA CATCCCCCTT TCGCCAGCTG GCGTAATAGC GAAGAGGCCC GCACCGATCG
6481 CCCTTCCCAA CAGTTGCGCA GCCTGAATGG CGAATGGCGC GACGCGCCCT GTAGCGGCGC
6541 ATTAAGCGCG GCGGGTGTGG TGGTTACGCG CAGCGTGACC GCTACACTTG CCAGCGCCCT
6601 AGCGCCCGCT CCTTTCGCTT TCTTCCCTTC CTTTCTCGCC ACGTTCGCCG GCTTTCCCCG
6661 TCAAGCTCTA AATCGGGGGC TCCCTTTAGG GTTCCGATTT AGTGCTTTAC GGCACCTCGA
6721 CCCCAAAAAA CTTGATTAGG GTGATGGTTC ACGTAGTGGG CCATCGCCCT GATAGACGGT
6781 TTTTCGCCCT TTGACGTTGG AGTCCACGTT CTTTAATAGT GGACTCTTGT TCCAAACTGG
6841 AACAACACTC AACCCTATCT CGGTCTATTC TTTTGATTTA TAAGGGATTT TGCCGATTTC
6901 GGCCTATTGG TTAAAAAATG AGCTGATTTA ACAAAAATTT AACGCGAATT TTAACAAAAT
6961 ATTAACGTTT ACAATTTCC

Plasmid-map and sequence of the **Transposon EF1α linear RAB7A LEAPER gRNA vector (pTS1900):**

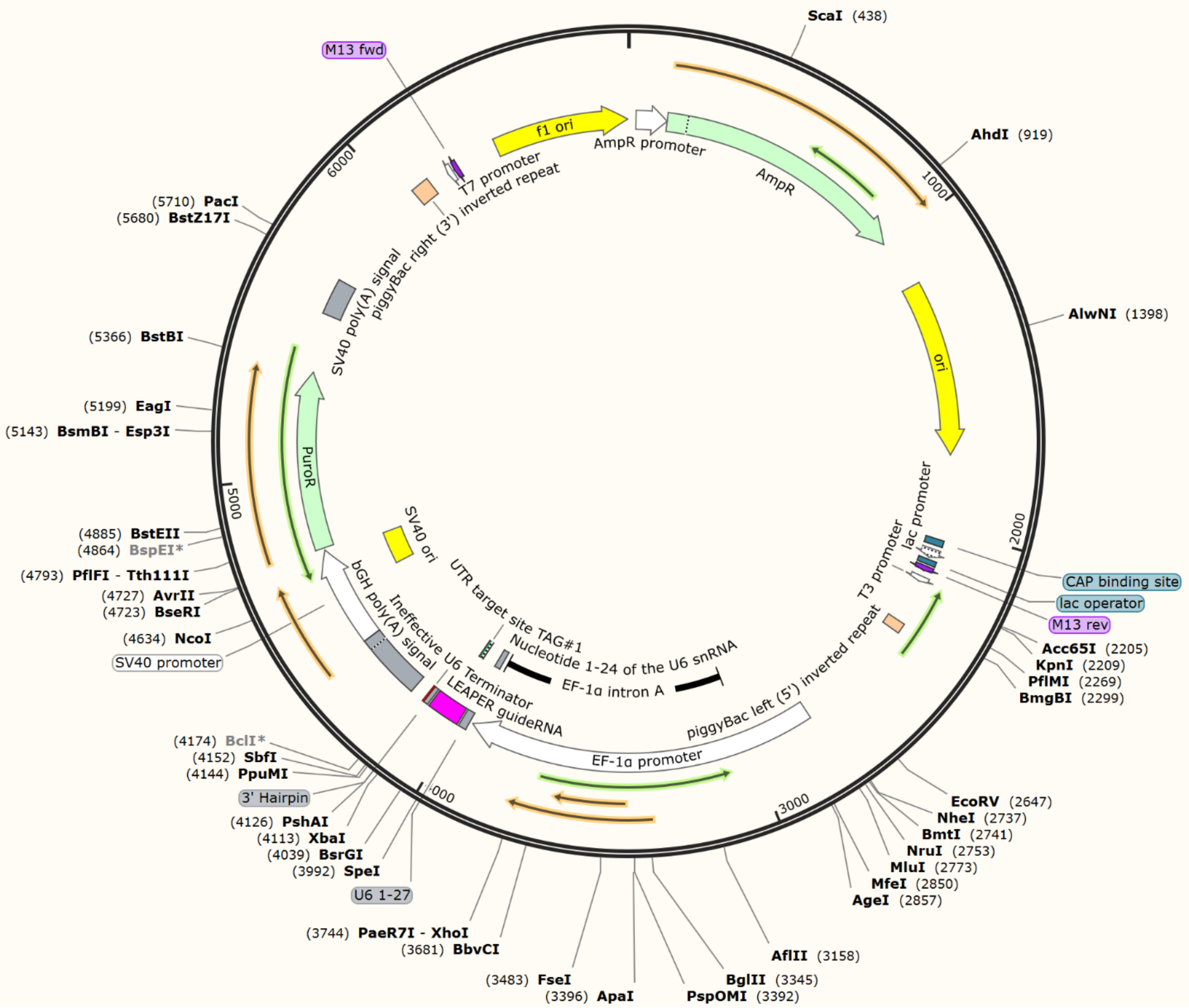

1 CAGGTGGCAC TTTTCGGGGA AATGTGCGCG GAACCCCTAT TTGTTTATTT TTCTAAATAC
61 ATTCAAATAT GTATCCGCTC ATGAGACAAT AACCCTGATA AATGCTTCAA TAATATTGAA
121 AAAGGAAGAG TATGAGTATT CAACATTTCC GTGTCGCCCT TATTCCCTTT TTTGCGGCAT
181 TTTGCCTTCC TGTTTTTGCT CACCCAGAAA CGCTGGTGAA AGTAAAAGAT GCTGAAGATC
241 AGTTGGGTGC ACGAGTGGGT TACATCGAAC TGGATCTCAA CAGCGGTAAG ATCCTTGAGA
301 GTTTTCGCCC CGAAGAACGT TTTCCAATGA TGAGCACTTT TAAAGTTCTG CTATGTGGCG
361 CGGTATTATC CCGTATTGAC GCCGGGCAAG AGCAACTCGG TCGCCGCATA CACTATTCTC
421 AGAATGACTT GGTTGAGTAC TCACCAGTCA CAGAAAAGCA TCTTACGGAT GGCATGACAG
481 TAAGAGAATT ATGCAGTGCT GCCATAACCA TGAGTGATAA CACTGCGGCC AACTTACTTC
541 TGACAACGAT CGGAGGACCG AAGGAGCTAA CCGCTTTTTT GCACAACATG GGGGATCATG
601 TAACTCGCCT TGATCGTTGG GAACCGGAGC TGAATGAAGC CATACCAAAC GACGAGCGTG
661 ACACCACGAT GCCTGTAGCA ATGGCAACAA CGTTGCGCAA ACTATTAACT GGCGAACTAC
721 TTACTCTAGC TTCCCGGCAA CAATTAATAG ACTGGATGGA GGCGGATAAA GTTGCAGGAC
781 CACTTCTGCG CTCGGCCCTT CCGGCTGGCT GGTTTATTGC TGATAAATCT GGAGCCGGTG
841 AGCGTGGGTC TCGCGGTATC ATTGCAGCAC TGGGGCCAGA TGGTAAGCCC TCCCGTATCG
901 TAGTTATCTA CACGACGGGG AGTCAGGCAA CTATGGATGA ACGAAATAGA CAGATCGCTG
961 AGATAGGTGC CTCACTGATT AAGCATTGGT AACTGTCAGA CCAAGTTTAC TCATATATAC

1021 TTTAGATTGA TTTAAAACTT CATTTTTAAT TTAAAAGGAT CTAGGTGAAG ATCCTTTTTG
1081 ATAATCTCAT GACCAAAATC CCTTAACGTG AGTTTTCGTT CCACTGAGCG TCAGACCCCG
1141 TAGAAAAGAT CAAAGGATCT TCTTGAGATC CTTTTTTTCT GCGCGTAATC TGCTGCTTGC
1201 AAACAAAAAA ACCACCGCTA CCAGCGGTGG TTTGTTTGCC GGATCAAGAG CTACCAACTC
1261 TTTTTCCGAA GGTAACTGGC TTCAGCAGAG CGCAGATACC AAATACTGTC CTTCTAGTGT
1321 AGCCGTAGTT AGGCCACCAC TTCAAGAACT CTGTAGCACC GCCTACATAC CTCGCTCTGC
1381 TAATCCTGTT ACCAGTGGCT GCTGCCAGTG GCGATAAGTC GTGTCTTACC GGGTTGGACT
1441 CAAGACGATA GTTACCGGAT AAGGCGCAGC GGTCGGGCTG AACGGGGGGT TCGTGCACAC
1501 AGCCCAGCTT GGAGCGAACG ACCTACACCG AACTGAGATA CCTACAGCGT GAGCTATGAG
1561 AAAGCGCCAC GCTTCCCGAA GGGAGAAAGG CGGACAGGTA TCCGGTAAGC GGCAGGGTCG
1621 GAACAGGAGA GCGCACGAGG GAGCTTCCAG GGGGAAACGC CTGGTATCTT TATAGTCCTG
1681 TCGGGTTTCG CCACCTCTGA CTTGAGCGTC GATTTTTGTG ATGCTCGTCA GGGGGGCGGA
1741 GCCTATGGAA AAACGCCAGC AACGCGGCCT TTTTACGGTT CCTGGCCTTT TGCTGGCCTT
1801 TTGCTCACAT GTTCTTTCCT GCGTTATCCC CTGATTCTGT GGATAACCGT ATTACCGCCT
1861 TTGAGTGAGC TGATACCGCT CGCCGCAGCC GAACGACCGA GCGCAGCGAG TCAGTGAGCG
1921 AGGAAGCGGA AGAGCGCCCA ATACGCAAAC CGCCTCTCCC CGCGCGTTGG CCGATTCATT
1981 AATGCAGCTG GCACGACAGG TTTCCCGACT GGAAAGCGGG CAGTGAGCGC AACGCAATTA
2041 ATGTGAGTTA GCTCACTCAT TAGGCACCCC AGGCTTTACA CTTTATGCTT CCGGCTCGTA
2101 TGTTGTGTGG AATTGTGAGC GGATAACAAT TTCACACAGG AAACAGCTAT GACCATGATT
2161 ACGCCAAGCT CGGAATTAAC CCTCACTAAA GGGAACAAAA GCTGGGTACC TCGCGCGACT
2221 TGGTTTGCCA TTCTTTAGCG CGCGTCGCGT CACACAGCTT GGCCACAATG TGGTTTTTGT
2281 CAAACGAAGA TTCTATGACG TGTTTAAAGT TTAGGTCGAG TAAAGCGCAA ATCTTTTTTA
2341 ACCCTAGAAA GATAGTCTGC GTAAAATTGA CGCATGCATT CTTGAAATAT TGCTCTCTCT
2401 TTCTAAATAG CGCGAATCCG TCGCTGTGCA TTTAGGACAT CTCAGTCGCC GCTTGGAGCT
2461 CCCGTGAGGC GTGCTTGTCA ATGCGGTAAG TGTCACTGAT TTTGAACTAT AACGACCGCG
2521 TGAGTCAAAA TGACGCATGA TTATCTTTTA CGTGACTTTT AAGATTTAAC TCATACGATA
2581 ATTATATTGT TATTTCATGT TCTACTTACG TGATAACTTA TTATATATAT ATTTTCTTGT
2641 TATAGATATC GTGACTAATA TATAATAAAA TGGGTAGTTC TTTAGACGAT GAGCATATCC
2701 TCTCTGCTCT TCTGCAAAGC GATGACGAGC TTGTTGGCTA GCGCGCTGCT TCGCGATGTA
2761 CGGGCCAGAT ATACGCGTGG CTCCGGTGCC CGTCAGTGGG CAGAGCGCAC ATCGCCCACA
2821 GTCCCCGAGA AGTTGGGGGG AGGGGTCGGC AATTGAACCG GTGCCTAGAG AAGGTGGCGC
2881 GGGGTAAACT GGGAAAGTGA TGTCGTGTAC TGGCTCCGCC TTTTTCCCGA GGGTGGGGGA
2941 GAACCGTATA TAAGTGCAGT AGTCGCCGTG AACGTTCTTT TTCGCAACGG GTTTGCCGCC
3001 AGAACACAGG TAAGTGCCGT GTGTGGTTCC CGCGGGCCTG GCCTCTTTAC GGGTTATGGC
3061 CCTTGCGTGC CTTGAATTAC TTCCACCTGG CTGCAGTACG TGATTCTTGA TCCCGAGCTT
3121 CGGGTTGGAA GTGGGTGGGA GAGTTCGAGG CCTTGCGCTT AAGGAGCCCC TTCGCCTCGT
3181 GCTTGAGTTG AGGCCTGGCC TGGGCGCTGG GGCCGCCGCG TGCGAATCTG GTGGCACCTT
3241 CGCGCCTGTC TCGCTGCTTT CGATAAGTCT CTAGCCATTT AAAATTTTTG ATGACCTGCT
3301 GCGACGCTTT TTTTCTGGCA AGATAGTCTT GTAAATGCGG GCCAAGATCT GCACACTGGT
3361 ATTTCGGTTT TTGGGGCCGC GGGCGGCGAC GGGGCCCGTG CGTCCCAGCG CACATGTTCG
3421 GCGAGGCGGG GCCTGCGAGC GCGGCCACCG AGAATCGGAC GGGGGTAGTC TCAAGCTGGC
3481 CGGCCTGCTC TGGTGCCTGG CCTCGCGCCG CCGTGTATCG CCCCGCCCTG GGCGGCAAGG
3541 CTGGCCCGGT CGGCACCAGT TGCGTGAGCG GAAAGATGGC CGCTTCCCGG CCCTGCTGCA
3601 GGGAGCTCAA AATGGAGGAC GCGGCGCTCG GGAGAGCGGG CGGGTGAGTC ACCCACACAA
3661 AGGAAAAGGG CCTTTCCGTC CTCAGCCGTC GCTTCATGTG ACTCCACGGA GTACCGGGCG
3721 CCGTCCAGGC ACCTCGATTA GTTCTCGAGC TTTTGGAGTA CGTCGTCTTT AGGTTGGGGG
3781 GAGGGGTTTT ATGCGATGGA GTTTCCCCAC ACTGAGTGGG TGGAGACTGA AGTTAGGCCA
3841 GCTTGGCACT TGATGTAATT CTCCTTGGAA TTTGCCCTTT TTGAGTTTGG ATCTTGGTTC
3901 ATTCTCAAGC CTCAGACAGT GGTTCAAAGT TTTTTTCTTC CATTTCAGGT GTCGTGAGGT
3961 CTAGCACCGT GCTCGCTTCG GCAGCACATA TACTAGTCGA CGTCTTTGAT AAAAGGCGTA
4021 CATAATTCTT GTGTCTACTG TACAGAATAC TGCCGCCAGC TGGATTTCCC AATTCTGAGT
4081 AACACTCTGC AATCCAAACA GGGTTCAACC CTTCTAGAGC GGACTTCGGT CCGCTTTTTA
4141 CTAGGACCTG CAGGCATGCT CGACAGAGCT CGCTGATCAG CCTCGACTGT GCCTTCTAGT
4201 TGCCAGCCAT CTGTTGTTTG CCCCTCCCCC GTGCCTTCCT TGACCCTGGA AGGTGCCACT
4261 CCCACTGTCC TTTCCTAATA AAATGAGGAA ATTGCATCGC ATTGTCTGAG TAGGTGTCAT
4321 TCTATTCTGG GGGGTGGGGT GGGGCAGGAC AGCAAGGGGG AGGATTGGGA AGATAATAGC
4381 AGGCATGCTG GGGATGCGGT GGGCTCTATG GCGTGTGTCA GTTAGGGTGT GGAAAGTCCC

4441 CAGGCTCCCC AGCAGGCAGA AGTATGCAAA GCATGCATCT CAATTAGTCA GCAACCAGGT
4501 GTGGAAAGTC CCCAGGCTCC CCAGCAGGCA GAAGTATGCA AAGCATGCAT CTCAATTAGT
4561 CAGCAACCAT AGTCCCGCCC CTAACTCCGC CCATCCCGCC CCTAACTCCG CCCAGTTCCG
4621 CCCATTCTCC GCCCCATGGC TGACTAATTT TTTTTATTTA TGCAGAGGCC GAGGCCGCCT
4681 CTGCCTCTGA GCTATTCCAG AAGTAGTGAG GAGGCTTTTT TGGAGGCCTA GGCTTTTGCA
4741 AAAAGCTCCC ATGACCGAGT ACAAGCCCAC GGTGCGCCTC GCCACCCGCG ACGACGTCCC
4801 CAGGGCCGTA CGCACCCTCG CCGCCGCGTT CGCCGACTAC CCCGCCACGC GCCACACCGT
4861 CGATCCGGAC CGCCACATCG AGCGGGTCAC CGAGCTGCAA GAACTCTTCC TCACGCGCGT
4921 CGGGCTCGAC ATCGGCAAGG TGTGGGTCGC GGACGACGGC GCCGCGGTGG CGGTCTGGAC
4981 CACGCCGGAG AGCGTCGAAG CGGGGGCGGT GTTCGCCGAG ATCGGCCCGC GCATGGCCGA
5041 GTTGAGCGGT TCCCGGCTGG CCGCGCAGCA ACAGATGGAA GGCCTCCTGG CGCCGCACCG
5101 GCCCAAGGAG CCCGCGTGGT TCCTGGCCAC CGTCGGCGTC TCGCCCGACC ACCAGGGCAA
5161 GGGTCTGGGC AGCGCCGTCG TGCTCCCCGG AGTGGAGGCG GCCGAGCGCG CCGGGGTGCC
5221 CGCCTTCCTG GAGACCTCCG CGCCCCGCAA CCTCCCCTTC TACGAGCGGC TCGGCTTCAC
5281 CGTCACCGCC GACGTCGAGG TGCCCGAAGG ACCGCGCACC TGGTGCATGA CCCGCAAGCC
5341 CGGTGCCTGA GCGGGACTCT GGGGTTCGAA ATGACCGACC AAGCGACGCC CGAAATGACC
5401 GACCAAGCGA CGCCCAACCT GCCATCACGA GATTTCGATT CCACCGCCGC CTTCTATGAA
5461 AGGTTGGGCT TCGGAATCGT TTTCCGGGAC GCCGGCTGGA TGATCCTCCA GCGCGGGGAT
5521 CTCATGCTGG AGTTCTTCGC CCACCCCAAC TTGTTTATTG CAGCTTATAA TGGTTACAAA
5581 TAAAGCAATA GCATCACAAA TTTCACAAAT AAAGCATTTT TTTCACTGCA TTCTAGTTGT
5641 GGTTTGTCCA AACTCATCAA TGTATCTTAT CATGTCTGTA TACCGTCGAC CTCTAGCTAG
5701 TCGAGTTAAT TAACGAGAGC ATAATATTGA TATGTGCCAA AGTTGTTTCT GACTGACTAA
5761 TAAGTATAAT TTGTTTCTAT TATGTATAGG TTAAGCTAAT TACTTATTTT ATAATACAAC
5821 ATGACTGTTT TTAAAGTACA AAATAAGTTT ATTTTTGTAA AAGAGAGAAT GTTTAAAAGT
5881 TTTGTTACTT TATAGAAGAA ATTTTGAGTT TTTGTTTTTT TTTAATAAAT AAATAAACAT
5941 AAATAAATTG TTTGTTGAAT TTATTATTAG TATGTAAGTG TAAATATAAT AAAACTTAAT
6001 ATCTATTCAA ATTAATAAAT AAACCTCGAT ATACAGACCG ATAAAACACA TGCGTCAATT
6061 TTACGCATGA TTATCTTTAA CGTACGTCAC AATATGATTA TCTTTCTAGG GTTAAATAAT
6121 AGTTTCTAAT TTTTTTATTA TTCAGCCTGC TGTCGTGAAT ACCGAGCTCC AATTCGCCCT
6181 ATAGTGAGTC GTATTACAAT TCACTGGCCG TCGTTTTACA ACGTCGTGAC TGGGAAAACC
6241 CTGGCGTTAC CCAACTTAAT CGCCTTGCAG CACATCCCCC TTTCGCCAGC TGGCGTAATA
6301 GCGAAGAGGC CCGCACCGAT CGCCCTTCCC AACAGTTGCG CAGCCTGAAT GGCGAATGGC
6361 GCGACGCGCC CTGTAGCGGC GCATTAAGCG CGGCGGGTGT GGTGGTTACG CGCAGCGTGA
6421 CCGCTACACT TGCCAGCGCC CTAGCGCCCG CTCCTTTCGC TTTCTTCCCT TCCTTTCTCG
6481 CCACGTTCGC CGGCTTTCCC CGTCAAGCTC TAAATCGGGG GCTCCCTTTA GGGTTCCGAT
6541 TTAGTGCTTT ACGGCACCTC GACCCCAAAA AACTTGATTA GGGTGATGGT TCACGTAGTG
6601 GGCCATCGCC CTGATAGACG GTTTTTCGCC CTTTGACGTT GGAGTCCACG TTCTTTAATA
6661 GTGGACTCTT GTTCCAAACT GGAACAACAC TCAACCCTAT CTCGGTCTAT TCTTTTGATT
6721 TATAAGGGAT TTTGCCGATT TCGGCCTATT GGTTAAAAAA TGAGCTGATT TAACAAAAAT
6781 TTAACGCGAA TTTTAACAAA ATATTAACGT TTACAATTTC C

Plasmid-map and sequence of the **CircLEAPER AC50 cloning vector (pTS2508):**

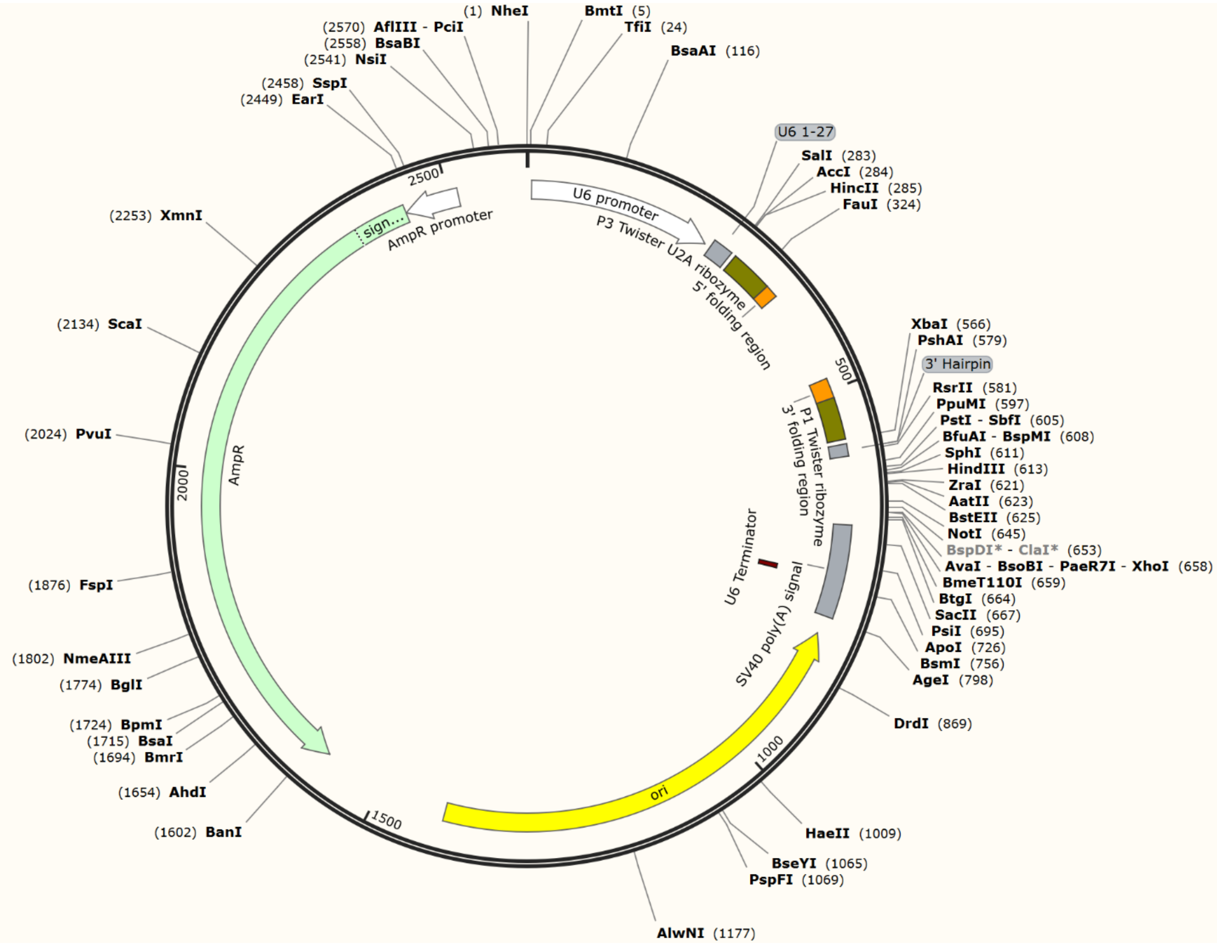

1 GCTAGCGAGG GCCTATTTCC CATGATTCCT TCATATTTGC ATATACGATA CAAGGCTGTT
61 AGAGAGATAA TTAGAATTAA TTTGACTGTA AACACAAAGA TATTAGTACA AAATACGTGA
121 CGTAGAAAGT AATAATTTCT TGGGTAGTTT GCAGTTTTAA AATTATGTTT TAAAATGGAC
181 TATCATATGC TTACCGTAAC TTGAAAGTAT TTCGATTTCT TGGCTTTATA TATCTTGTGG
241 AAAGGACGAA ACACCGTGCT CGCTTCGGCA GCACATATAC TAGTCGACGC CATCAGTCGC
301 CGGTCCCAAG CCCGGATAAA ATGGGAGGGG GCGGGAAACC GCCTAACCAT GCCGACTGAT
361 GGCAGAAAAA CAAAAAACAA AAAAAACAAA AAAAAAACCA AAAAAACAAA ACACATGGTC
421 TTCGTGGAAG ACTTAAAAAC AAAAAACAAA AAAAACAAAA AAAAAACCAA AAAAACAAAA
481 CACACTGCCA TCAGTCGGCG TGGACTGTAG AACACTGCCA ATGCCGGTCC CAAGCCCGGA
541 TAAAAGTGGA GGGTACAGTC CACGCTCTAG AGCGGACTTC GGTCCGCTTT TTACTAGGAC
601 CTGCAGGCAT GCAAGCTTGA CGTCGGTTAC CGATATCCAT ATGGCGGCCG CATCGATCTC
661 GAGCCGCGGA CTAGTAACTT GTTTATTGCA GCTTATAATG GTTACAAATA AAGCAATAGC
721 ATCACAAATT TCACAAATAA AGCATTTTTT TCACTGCATT CTAGTTGTGG TTTGTCCAAA
781 CTCATCAATG TATCTTAACC GGTGGCCGCG TTGCTGGCGT TTTTCCATAG GCTCCGCCCC
841 CCTGACGAGC ATCACAAAAA TCGACGCTCA AGTCAGAGGT GGCGAAACCC GACAGGACTA
901 TAAAGATACC AGGCGTTTCC CCCTGGAAGC TCCCTCGTGC GCTCTCCTGT TCCGACCCTG
961 CCGCTTACCG GATACCTGTC CGCCTTTCTC CCTTCGGGAA GCGTGGCGCT TTCTCATAGC
1021 TCACGCTGTA GGTATCTCAG TTCGGTGTAG GTCGTTCGCT CCAAGCTGGG CTGTGTGCAC
1081 GAACCCCCCG TTCAGCCCGA CCGCTGCGCC TTATCCGGTA ACTATCGTCT TGAGTCCAAC
1141 CCGGTAAGAC ACGACTTATC GCCACTGGCA GCAGCCACTG GTAACAGGAT TAGCAGAGCG
1201 AGGTATGTAG GCGGTGCTAC AGAGTTCTTG AAGTGGTGGC CTAACTACGG CTACACTAGA

1261 AGAACAGTAT TTGGTATCTG CGCTCTGCTG AAGCCAGTTA CCTTCGGAAA AAGAGTTGGT
1321 AGCTCTTGAT CCGGCAAACA AACCACCGCT GGTAGCGGTG GTTTTTTTGT TTGCAAGCAG
1381 CAGATTACGC GCAGAAAAAA AGGATCTCAA GAAGATCCTT TGATCTTTTC TACGGGGTCT
1441 GACGCTCAGT GGAACGAAAA CTCACGTTAA GGGATTTTGG TCATGAGATT ATCAAAAAGG
1501 ATCTTCACCT AGATCCTTTT AAATTAAAAA TGAAGTTTTA AATCAATCTA AAGTATATAT
1561 GAGTAAACTT GGTCTGACAG TTACCAATGC TTAATCAGTG AGGCACCTAT CTCAGCGATC
1621 TGTCTATTTC GTTCATCCAT AGTTGCCTGA CTCCCCGTCG TGTAGATAAC TACGATACGG
1681 GAGGGCTTAC CATCTGGCCC CAGTGCTGCA ATGATACCGC GAGACCCACG CTCACCGGCT
1741 CCAGATTTAT CAGCAATAAA CCAGCCAGCC GGAAGGGCCG AGCGCAGAAG TGGTCCTGCA
1801 ACTTTATCCG CCTCCATCCA GTCTATTAAT TGTTGCCGGG AAGCTAGAGT AAGTAGTTCG
1861 CCAGTTAATA GTTTGCGCAA CGTTGTTGCC ATTGCTACAG GCATCGTGGT GTCACGCTCG
1921 TCGTTTGGTA TGGCTTCATT CAGCTCCGGT TCCCAACGAT CAAGGCGAGT TACATGATCC
1981 CCCATGTTGT GCAAAAAAGC GGTTAGCTCC TTCGGTCCTC CGATCGTTGT CAGAAGTAAG
2041 TTGGCCGCAG TGTTATCACT CATGGTTATG GCAGCACTGC ATAATTCTCT TACTGTCATG
2101 CCATCCGTAA GATGCTTTTC TGTGACTGGT GAGTACTCAA CCAAGTCATT CTGAGAATAG
2161 TGTATGCGGC GACCGAGTTG CTCTTGCCCG GCGTCAATAC GGGATAATAC CGCGCCACAT
2221 AGCAGAACTT TAAAAGTGCT CATCATTGGA AAACGTTCTT CGGGGCGAAA ACTCTCAAGG
2281 ATCTTACCGC TGTTGAGATC CAGTTCGATG TAACCCACTC GTGCACCCAA CTGATCTTCA
2341 GCATCTTTTA CTTTCACCAG CGTTTCTGGG TGAGCAAAAA CAGGAAGGCA AAATGCCGCA
2401 AAAAAGGGAA TAAGGGCGAC ACGGAAATGT TGAATACTCA TACTCTTCCT TTTTCAATAT
2461 TATTGAAGCA TTTATCAGGG TTATTGTCTC ATGAGCGGAT ACATATTTGA ACGGCCGGAT
2521 TTCTTGTCAC GCTTTGATGC ATCGTTGGTG GTTGATGGAT ATCTGACGAA CATGTGCACA
2581 GTGGTACAAG GTATTCCTGG TTG

References:

1. Reautschnig, P., et al., *CLUSTER guide RNAs enable precise and efficient RNA editing with endogenous ADAR enzymes in vivo.* Nat Biotechnol, 2022. **40**(5): p. 759-768.
2. Wettengel, J., et al., *Harnessing human ADAR2 for RNA repair - Recoding a PINK1 mutation rescues mitophagy.* Nucleic Acids Res, 2017. **45**(5): p. 2797-2808.

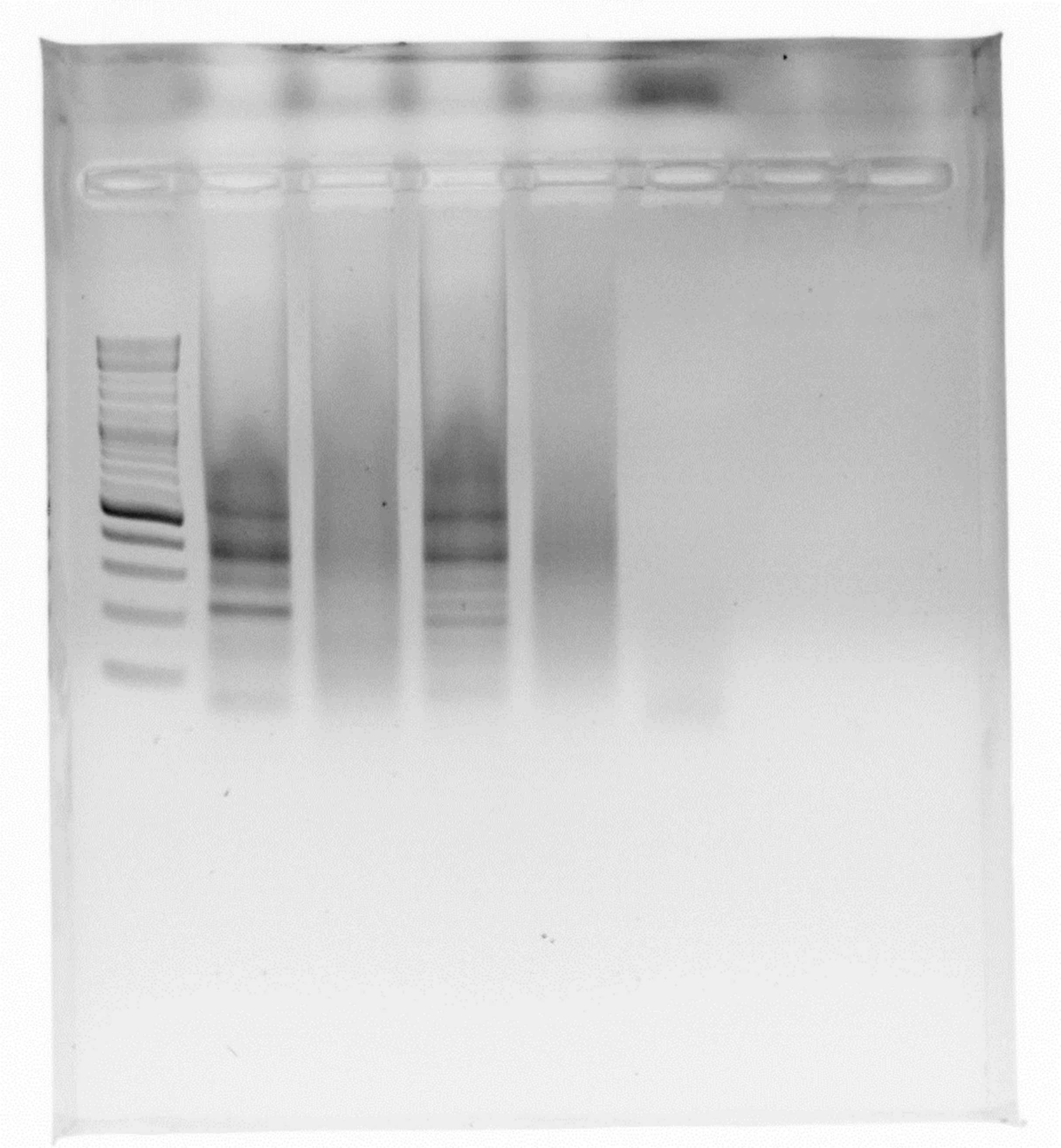

Uncropped blot used for Figure S6b. The ladder used in the left most lane is the 1 kb Plus DNA Ladder (#N3200, NEB).
