# Supplementary material for: Precise in vivo RNA base editing with a wobble-enhanced circular CLUSTER guide RNA
Source: Nat Biotechnol. 2024 Jul 12;43(4):545–57. doi: 10.1038/s41587-024-02313-0 (PMC11994451; doi:10.1038/s41587-024-02313-0)
Supplement: Supplementary file 2 — Reporting Summary [file 41587_2024_2313_MOESM2_ESM.pdf]

nature portfolio

Corresponding author(s): Thorsten Stafforst

Last updated by author(s): May 1, 2024

Reporting Summary

Nature Portfolio wishes to improve the reproducibility of the work that we publish. This form provides structure for consistency and transparency in reporting. For further information on Nature Portfolio policies, see our Editorial Policies and the Editorial Policy Checklist.

Statistics

For all statistical analyses, confirm that the following items are present in the figure legend, table legend, main text, or Methods section.

n/a	Confirmed	
☐	☒	The exact sample size (*n*) for each experimental group/condition, given as a discrete number and unit of measurement
☐	☒	A statement on whether measurements were taken from distinct samples or whether the same sample was measured repeatedly
☐	☒	The statistical test(s) used AND whether they are one- or two-sided<br>*Only common tests should be described solely by name; describe more complex techniques in the Methods section.*
☐	☒	A description of all covariates tested
☒	☐	A description of any assumptions or corrections, such as tests of normality and adjustment for multiple comparisons
☐	☒	A full description of the statistical parameters including central tendency (e.g. means) or other basic estimates (e.g. regression coefficient) AND variation (e.g. standard deviation) or associated estimates of uncertainty (e.g. confidence intervals)
☐	☒	For null hypothesis testing, the test statistic (e.g. *F*, *t*, *r*) with confidence intervals, effect sizes, degrees of freedom and *P* value noted<br>*Give P values as exact values whenever suitable.*
☒	☐	For Bayesian analysis, information on the choice of priors and Markov chain Monte Carlo settings
☒	☐	For hierarchical and complex designs, identification of the appropriate level for tests and full reporting of outcomes
☒	☐	Estimates of effect sizes (e.g. Cohen's *d*, Pearson's *r*), indicating how they were calculated

Our web collection on statistics for biologists contains articles on many of the points above.

Software and code

Policy information about availability of computer code

Data collection	The full code of the GuideRNA-Forge tool is available upon request. The compiled tool will be available online prior to publication under: https://github.com/recruitment-cluster-finder/GuideRNA-FORGE
Data analysis	Data were analyzed using Excel 2016 and GraphPad Prism 8,<br>figures were created with CorelDraw 2017,<br>the manuscript was written with Word 2016,<br>the custom "GuideRNA Forge" tool was written in python (version 3.9) and used the Vienna RNA package (version 2.0) (https://www.tbi.univie.ac.at/RNA/),<br>gRNA folds were created using the RNAfold web server (University of Vienna, http://rna.tbi.univie.ac.at/cgi-bin/RNAWebSuite/RNAfold.cgi),<br>qPCR analysis was performed using the 7500 data analysis software version 1.1 (animal experiment) and version 2.3 (rest of the study),<br>structural analysis was performed using the open-source PyMOL™ Molecular Graphics System (version 2.5.0),<br>Sanger sequence traces were analyzed using SNAP-Gene (version 4.2.11),<br>microscopy images were acquired using ZEN 2.3 SPI FPS Black (version 14.0.0.0) and pseudo colored using ImageJ (version 1.54f),<br>RNA-seq. reads were demultiplexed using Illumina bcl2fastq (version 2.20),<br>RNA-seq. adapters were trimmed with Skewer (version 0.2.2),<br>RNA-seq. FASTQ file quality was assessed with FastQC (version 0.11.8),<br>RNA-seq. reads were aligned to the GRCm38/mm10 reference genome using STAR (version 2.7.3a),<br>Deep amplicon-seq. reads were demultiplexed using BCL Convert (version 2.4.0),<br>Deep amplicon-seq. adapters were trimmed with FastQ (version 1.0.0),<br>Deep amplicon-seq. FASTQ file quality was assessed with FastQC (version 0.11.9),<br>Deep amplicon-seq. reads were quality trimmed and their low quality bases masked using Seqtk (version 1.3-r106),

Deep amplicon-seq. reads were aligned to the GRCm38/mm10 reference genome using BWA-MEM (version 0.7.17-r1188),
Deep amplicon-seq. alignments were analyzed using the Integrative Genomics Viewer (version 2.16.2).

For manuscripts utilizing custom algorithms or software that are central to the research but not yet described in published literature, software must be made available to editors and reviewers. We strongly encourage code deposition in a community repository (e.g. GitHub). See the Nature Portfolio guidelines for submitting code & software for further information.

Data

Policy information about availability of data

All manuscripts must include a data availability statement. This statement should provide the following information, where applicable:

- Accession codes, unique identifiers, or web links for publicly available datasets
- A description of any restrictions on data availability
- For clinical datasets or third party data, please ensure that the statement adheres to our policy

Transcriptome-wide RNA-seq data are accessible via the NCBI GEO database with accession code GSE265898. Deep amplicon sequencing data are accessible via the NCBI Sequence Read Archive (SRA) database with accession code PRJNA1100948. Transcriptome-wide RNA-seq data from Sinnamon et al. are accessible via the NCBI SRA database with accession code PRJNA849938. Structure data from Thuy-Boun et al. and Stefl et al. are available with Protein Data Bank ID 5HP2 and 2L2J, respectively.

Research involving human participants, their data, or biological material

Policy information about studies with human participants or human data. See also policy information about sex, gender (identity/presentation), and sexual orientation and race, ethnicity and racism.

Reporting on sex and gender	No research involving human participants.
Reporting on race, ethnicity, or other socially relevant groupings	No research involving human participants.
Population characteristics	No research involving human participants.
Recruitment	No research involving human participants.
Ethics oversight	No research involving human participants.

Note that full information on the approval of the study protocol must also be provided in the manuscript.

Field-specific reporting

Please select the one below that is the best fit for your research. If you are not sure, read the appropriate sections before making your selection.

☒ Life sciences ☐ Behavioural & social sciences ☐ Ecological, evolutionary & environmental sciences

For a reference copy of the document with all sections, see nature.com/documents/nr-reporting-summary-flat.pdf

Life sciences study design

All studies must disclose on these points even when the disclosure is negative.

Sample size

Experiments for evaluating editing yields via Sanger sequencing were mostly done in n=3 biological replicates in rare cases in n=4 or n=5. When possible (in bar diagrams or box plots) data points are displayed individually. In case of heat maps (in-vitro experiments) the mean ± s.d. was displayed.
The evaluation of the editing yields via Sanger sequencing for the animal experiment was done with n=3-5 mice per group, with each indicated brain region being measured individually as one technical replicate.
Amplicon-seq. and RNA-seq. for the animal experiment were done with n=2 mice per group. In case of heat maps (in-vivo experiment) the median ± 95% confidence interval was displayed.
qPCR quantifications for the animal experiment were done with n=2 mice and n=3 technical replicates per tissue per mouse.
The evaluation of the fold change via the dual-luciferase assay for cell culture experiments was done with n=5 biological replicates with one technical measurement each.
No sample size calculation was performed. The sample sizes for cell culture experiments were selected based on the prior knowledge of variation. The group sizes for animal experiments were selected based on "Sinnamon, J. R., et al. (2022). "Targeted RNA editing in brainstem alleviates respiratory dysfunction in a mouse model of Rett syndrome." Proc Natl Acad Sci U S A 119(33): e2206053119."

Data exclusions

One of the non-targeting CLUSTER gRNA samples from the next-generation RNA-sequencing experiment failed quality control (RIN value 1) and thus had to be removed from the analysis.

Replication

All experiments could be reproduced, as shown in the manuscript, the number of replications and nature of replicates is always given in the figure caption.

Randomization	All samples were treated according to the same protocols side-by-side with the respective controls and thus, there was no requirement for randomization.
Blinding	No blinding was performed due to the involvement of several experimentators and several laboratories.

Reporting for specific materials, systems and methods

We require information from authors about some types of materials, experimental systems and methods used in many studies. Here, indicate whether each material, system or method listed is relevant to your study. If you are not sure if a list item applies to your research, read the appropriate section before selecting a response.

Materials & experimental systems

n/a	Involved in the study
☐	☒ Antibodies
☐	☒ Eukaryotic cell lines
☒	☐ Palaeontology and archaeology
☐	☒ Animals and other organisms
☒	☐ Clinical data
☒	☐ Dual use research of concern
☒	☐ Plants

Methods

n/a	Involved in the study
☒	☐ ChIP-seq
☒	☐ Flow cytometry
☒	☐ MRI-based neuroimaging

Antibodies

Antibodies used	MeCP2 (D4F3) XP Rabbit mAb (Monoclonal IgG, Cell Signaling Technology, #3456S), Donkey α Rabbit AlexaFluor 488 (Polyclonal IgG, Invitrogen, A21206)
Validation	MeCP2 (D4F3) XP Rabbit mAb (Commonly used commercial antibody validated in KO mouse tissue sections previously in our lab. RRID: AB_2143849, PMID: 35939700)<br><br>Donkey α Rabbit AlexaFluor 488 (Commonly used commercial antibody with 135 IHC-IF citations as of Jan. 2024. RRID: AB_2535792 Recent publications: PMID: 36865524, PMID: 36321664, PMID: 38017073)

Eukaryotic cell lines

Policy information about cell lines and Sex and Gender in Research

Cell line source(s)	HeLa (cat. no. CCL-2, ATCC)<br>HEK-Flp-In T-Rex-A1p110, A1p150, A2 (cat. no. R78007, Thermo Fisher scientific, stably transfected with ADAR1 p110 vector in our lab)<br>293FT (cat. no. R70007, Thermo Fisher scientific)
Authentication	Authentication via STR profiling was performed by the commercial suppliers before purchase of the material. Cell lines were not additionally authenticated by us.
Mycoplasma contamination	The HeLa cell line has been tested as mycoplasma-free by the commercial suppliers and in house. 293FT and HEK-Flp-In T-Rex (A1p110, A1p150, A2) cell lines were purchased mycoplasma-free, but not tested for mycoplasma in-house.
Commonly misidentified lines (See ICLAC register)	None were used.

Animals and other research organisms

Policy information about studies involving animals; ARRIVE guidelines recommended for reporting animal research, and Sex and Gender in Research

Laboratory animals	Species: mouse, strain: C57BL/6, disease: Rett syndrome caused by Mecp2 311G>A mutation.<br>All groups consisted of hemizygous male mice.<br>The animals were between P30 and P34 at the time of AAV treatment.
Wild animals	No wild animals were used in this study.
Reporting on sex	The findings in this study apply to male mice. Their sex was identified via PCR (see methods section). Female homozygous Rett mice (Mecp2-/-) are hard to obtain due to the inability to use hemizygous Rett males (Mecp2-/y) as breeders as they have a decreased life span and Rett-like symptoms that begin to emerge early in life. Homozygous females are also not a model for the human disease because the human Rett females are heterozygous. The hemizygous males are the optimal choice for our experimentation because

	we are able to accurately study the Mecp2 RNA editing efficiency and protein recovery without worrying about the mosaicism that Mecp2+/- females demonstrate.
Field-collected samples	No field-collected samples were used in this study.
Ethics oversight	All animal procedures were approved by the Institutional Animal Care and Use Committees of Oregon Health and Science University (IACUC protocol number: IP00000284).

Note that full information on the approval of the study protocol must also be provided in the manuscript.

Plants

Seed stocks	No plants were used in this study.
Novel plant genotypes	No plants were used in this study.
Authentication	No plants were used in this study.
